# High BMI-attributable female-specific cancers: a comprehensive analysis of the global disease burden and trends from 1990 to 2021 and projections to 2040

**DOI:** 10.3389/fonc.2025.1704299

**Published:** 2025-10-29

**Authors:** Guangming Sun, Junmei Tang, Hao Chen, Yue Zhu, Pan Ren, Hanyue Gan, Wenbin Wu

**Affiliations:** Department of Geriatrics, Hospital of Chengdu University of Traditional Chinese Medicine, Chengdu, China

**Keywords:** high body mass index, breast cancer, ovarian cancer, uterine cancer, Global Burden of Disease

## Abstract

**Background:**

High body mass index (BMI) is a key modifiable risk factor for breast, ovarian, and uterine cancer. Despite the ongoing global obesity epidemic, a systematic assessment of the long-term burden trends and disparities of these cancers attributable to high BMI is lacking, particularly across regions with different sociodemographic development. This study quantifies these trends and disparities, providing an evidence base to inform equitable global cancer prevention strategies.

**Methods:**

We extracted data on deaths and disability-adjusted life years (DALYs) for breast, ovarian, and uterine cancer attributable to high BMI from 1990 to 2021 across 204 countries from the Global Burden of Disease 2021 study. We used Joinpoint regression to analyze temporal trends in age-standardized rates (ASRs), employed efficiency frontier analysis to assess burden control, and quantified inequalities using the Slope Index of Inequality (SII) and Concentration Index (CI). Finally, an Autoregressive Integrated Moving Average (ARIMA) model was used to project burden trends to 2040.

**Results:**

Globally, from 1990 to 2021, annual deaths attributable to high BMI for these cancers more than doubled from 36,000 to 88,000, while DALYs rose from 0.83 million to 2.13 million. Burden trajectories showed marked divergence by Sociodemographic Index (SDI). In high-SDI regions, the burdens of breast and ovarian cancer declined, while the uterine cancer burden increased (AAPC in ASDALYs = 0.86%). Conversely, in low- and middle-SDI regions, the burdens of three cancers increased. The most pronounced rise occurred in ovarian cancer in low-SDI regions (ASDR AAPC of 4.49%). Inequality analysis revealed a widening absolute gap in burden, with the SII for ovarian cancer increasing by 95.3%. Projections indicate that DALY burdens for breast and ovarian cancer will continue to increase by 2040.

**Conclusion:**

The rising global burden of female-specific cancers attributable to high BMI is shifting disproportionately to low-SDI regions, exacerbating absolute health inequalities. This highlights an urgent need to integrate weight management and nutritional interventions into equitable, context-specific prevention strategies to address this growing global health challenge.

## 1 Introduction

Breast, ovarian, and uterine cancers represent a primary global public health challenge. The scale of this challenge is highlighted by 2022 global statistics, wherein breast cancer accounted for 23.8% of all new cancer diagnoses in women, while uterine and ovarian cancers comprised 4.3% and 3.4%, respectively (1). Collectively, these malignancies impose a substantial physical, psychological, and socioeconomic burden on patients, families, and healthcare systems (2). Therefore, a comprehensive analysis of their epidemiological trends is essential for developing targeted public health strategies and optimizing resource allocation.

A high body mass index (BMI) is a well-established modifiable risk factor for breast, ovarian, and uterine cancers (3, 4). The link is underpinned by several biological mechanisms, including the induction of chronic inflammation, the supply of excess energy to malignant cells, and, most critically, the disruption of endogenous hormonal balance through altered estrogen and insulin-like growth factor levels (5–8). This hormonal dysregulation is a key driver in the pathogenesis of these malignancies. For breast cancer, increased body fat elevates aromatase activity, which converts cholesterol into estradiol. The resulting rise in local estrogen activates estrogen receptors, driving tumor development (9, 10); furthermore, the cholesterol metabolite 27-hydroxycholesterol can independently promote cancer cell growth through similar estrogen-like mechanisms (11). In uterine cancer, obesity-induced hyperestrogenism, combined with insulin resistance and a disrupted estrogen-progesterone balance, significantly increases disease risk (12, 13). Meanwhile, in ovarian cancer, a high-fat diet provides substrates for estrogen synthesis and elevates gonadotropins like luteinizing hormone, which collectively stimulate the abnormal proliferation of ovarian epithelial cells (14, 15).

The Global Burden of Disease (GBD) study represents a foundational resource for global health assessment. The GBD 2021, for instance, provides a comprehensive epidemiological assessment of 371 diseases and 88 risk factors across 204 countries and territories (16). Utilizing a standardized framework that integrates extensive data sources with advanced statistical modeling, the GBD generates comparable, granular estimates of metrics such as incidence, prevalence, death, and disability-adjusted life years (DALYs). This rigorous methodology ensures the high reliability and cross-national comparability of its findings (17). While previous GBD reports have documented the overarching epidemiological trends of female cancers and identified high BMI as a key attributable risk factor (2), a granular, forward-looking analysis of the evolving burden and future trends specifically attributable to high BMI remains largely unexplored. Moreover, a systematic analysis of how this burden is distributed across regions of varying socioeconomic development and how these disparities have evolved over the past three decades is notably absent from the literature. To address this knowledge gap, our study leverages data from GBD 2021 to systematically analyze the burden attributable to high BMI for breast, ovarian, and uterine cancers, which represent all female-specific malignancies with attributable risk data for this factor in the GBD 2021 study, from 1990 to 2021 at global, regional, and Socio-Demographic Index (SDI) levels. We then employ health inequality analyses to precisely quantify the magnitude of these socioeconomic disparities and map their temporal evolution. Ultimately, this study aims to provide refined evidence on the attributable burden, offering a scientific basis for developing more targeted and equitable cancer prevention and control policies worldwide.

## 2 Materials and methods

### 2.1 Data sources

This study utilized data extracted from the GBD 2021. The GBD 2021 provides comprehensive and systematic health estimates for 204 countries and territories from 1990 to 2021, stratified by key demographic and socioeconomic factors (18, 19). We analyzed the burden of female breast, ovarian, and uterine cancers attributable to high BMI at global, regional (21 GBD super-regions), and national (204 countries and territories) levels. These three cancers were selected as they represent the complete set of female-specific malignancies for which the GBD 2021 provides attributable burden estimates linked to high BMI. For detailed data collection methods, refer to the GBD technical appendix (https://www.healthdata.org/gbd/methods-appendices-2021). All source data are publicly available from the Global Health Data Exchange (GHDx) via the GBD Results Tool (https://vizhub.healthdata.org/gbd-results/).

The primary outcomes were deaths and DALYs attributable to high BMI for the three specified cancers. We extracted the absolute number of deaths and DALYs, alongside their corresponding age-standardized rates (ASDR and ASDALYR). All estimates are presented with 95% uncertainty intervals (UIs), derived from the 2.5th and 97.5th percentiles of 1,000 posterior draws from the GBD model. Following GBD protocol, statistical significance was determined based on these UIs. A change was considered significant if its 95% UI did not include zero, and a difference between two estimates was considered significant if their 95% UIs did not overlap.

In this analysis, high BMI was defined as a BMI greater than 25 kg/m² in adults (aged 20 years and older), consistent with the definition of this specific risk factor in the GBD 2021 study (20). Disability-adjusted life years (DALYs), a composite measure of disease burden, are calculated as the sum of years of life lost (YLLs) due to premature death and years lived with disability (YLDs). One DALY represents the loss of one year of healthy life. Age-standardized rates (ASRs), including ASDR and ASDALYR, were calculated by standardizing age-specific rates to the GBD global standard population structure. ASRs are designed to eliminate the influence of differences in population age structures across groups or over time on disease rate comparisons, thereby enabling more accurate cross-regional and temporal comparisons (18). The SDI is a composite measure of national or regional development level in the GBD study, calculated as the geometric mean of total fertility rate, mean years of education in populations aged 15 years and older, and per capita income (21). The SDI ranges from 0 to 1. Based on SDI values, the GBD study categorizes countries and regions into five levels: low SDI, low-middle SDI, middle SDI, high-middle SDI, and high SDI.

This study adhered to the Guidelines for Accurate and Transparent Health Estimates Reporting (GATHER) (22). As the GBD data are publicly available and de-identified aggregate data, this study did not require additional ethical approval or informed consent.

### 2.2 Statistical analysis

This study performed a systematic analysis of the burden of disease data on female breast cancer, ovarian cancer, and uterine cancer attributable to high BMI, extracted from the GBD 2021 database. In order to ensure meaningful comparisons across different time periods, regions, and populations, all death and DALY rates were calculated using age-standardized methods, specifically ASDR and ASDALYR.

Using descriptive analysis, we conducted a comprehensive assessment of the disease burden status at two specific time points, 1990 and 2021, for the world, 21 GBD regions, 204 countries and territories, and the five SDI quintile regions (high SDI, upper-middle SDI, middle SDI, lower-middle SDI, and low SDI). The analysis encompasses the absolute numbers of deaths and DALYs attributable to high BMI for the three cancers, along with the corresponding ASDR and ASDALYR. Additionally, the global burden trends were disaggregated by year and age groups. To quantify long-term trends in disease burden from 1990 to 2021, we employed joinpoint regression analysis (Joinpoint Regression Program). This statistical method identifies significant inflection points within a time series, connecting them to construct a series of continuous linear segments that best fit the data. The analytical procedure began with a zero-joinpoint model, representing a single linear trend. Subsequently, the model sequentially incorporated additional joinpoints, to test for improved model fit. At each step, a Monte Carlo permutation test was used to determine whether the addition of a joinpoint resulted in a statistically significant improvement. This validation ensures that any identified change in trend represents a genuine shift rather than random fluctuation (23). For each trend segment, we computed the Annual Percentage Change (APC). Building on this, we computed the Average Annual Percentage Change (AAPC) over the entire study period, along with its 95% confidence interval (CI), and employed it as the key indicator for assessing the overall trend. Each linear segment in the Joinpoint regression model is based on the relationship between the natural logarithm (ln) of ASR and calendar year (x): ln(ASR) = α + βx + ϵ, where y is ln(ASR), x is the calendar year, and ϵ represents the error term (24). When the 95% CI for APC or AAPC does not include zero, we conclude that the trend change is statistically significant (25). To investigate in more detail the driving factors of changes in the absolute disease burden, we conducted a Das Gupta decomposition method. This method allows us to quantify the relative contributions of three main factors: population growth, changes in age structure, and age-specific attribution rates to changes in total deaths and DALYs (26). In the analysis of disparities and inequalities, we first examined the age-specific patterns of ASDR and ASDALYR to identify risk profiles across different life stages. Next, we illustrated the spatial distribution of disease burden on global maps and assessed the strength of the correlation between ASDR, ASDALYR, and the SDI across regions using Spearman’s rank correlation method. To further assess health inequality, we calculated the SII and the Concentration Index (CI), and analyzed their changes over time to examine the absolute and relative inequalities in health outcomes across the socioeconomic gradient (27). For benchmark comparison and future forecasting, we employed the efficiency frontier analysis to identify countries with best practices in controlling the attributable burden, based on their SDI levels, and offer these as reference benchmarks for other countries (16). To forecast trends in the ASDR and ASDALYR through 2040, we employed the Autoregressive Integrated Moving Average (ARIMA) model. This method is adept at handling non-stationary epidemiological time-series, as it captures complex temporal patterns to generate more robust predictions than simple linear extrapolation (28). Optimal model selection followed a systematic, three-stage procedure: determining the differencing order (d) through stationarity testing; identifying the autoregressive (p) and moving average (q) orders by minimizing the Akaike (AIC) and Bayesian (BIC) Information Criteria. Model validation was twofold: the coefficient of determination (R-squared) assessed goodness-of-fit by quantifying the explained historical variance, while the Ljung-Box Q test confirmed model adequacy by ensuring the independent distribution of residuals (29–31). All statistical analyses and data visualizations were conducted with the Joinpoint Regression Program (version 5.1.0) and R software (version 4.5.1), with data processing and graphical plotting carried out using relevant packages, including ggplot2, dplyr, ggpubr, and forecast. All statistical tests were performed as two-tailed tests, with a *p*-value of less than 0.05 regarded as statistically significant.

## 3 Results

### 3.1 The overall burden and temporal trends of female cancers attributed to high BMI

#### 3.1.1 A global overview and temporal trends of the disease burden of female cancers attributed to high BMI

**Figure 1:**
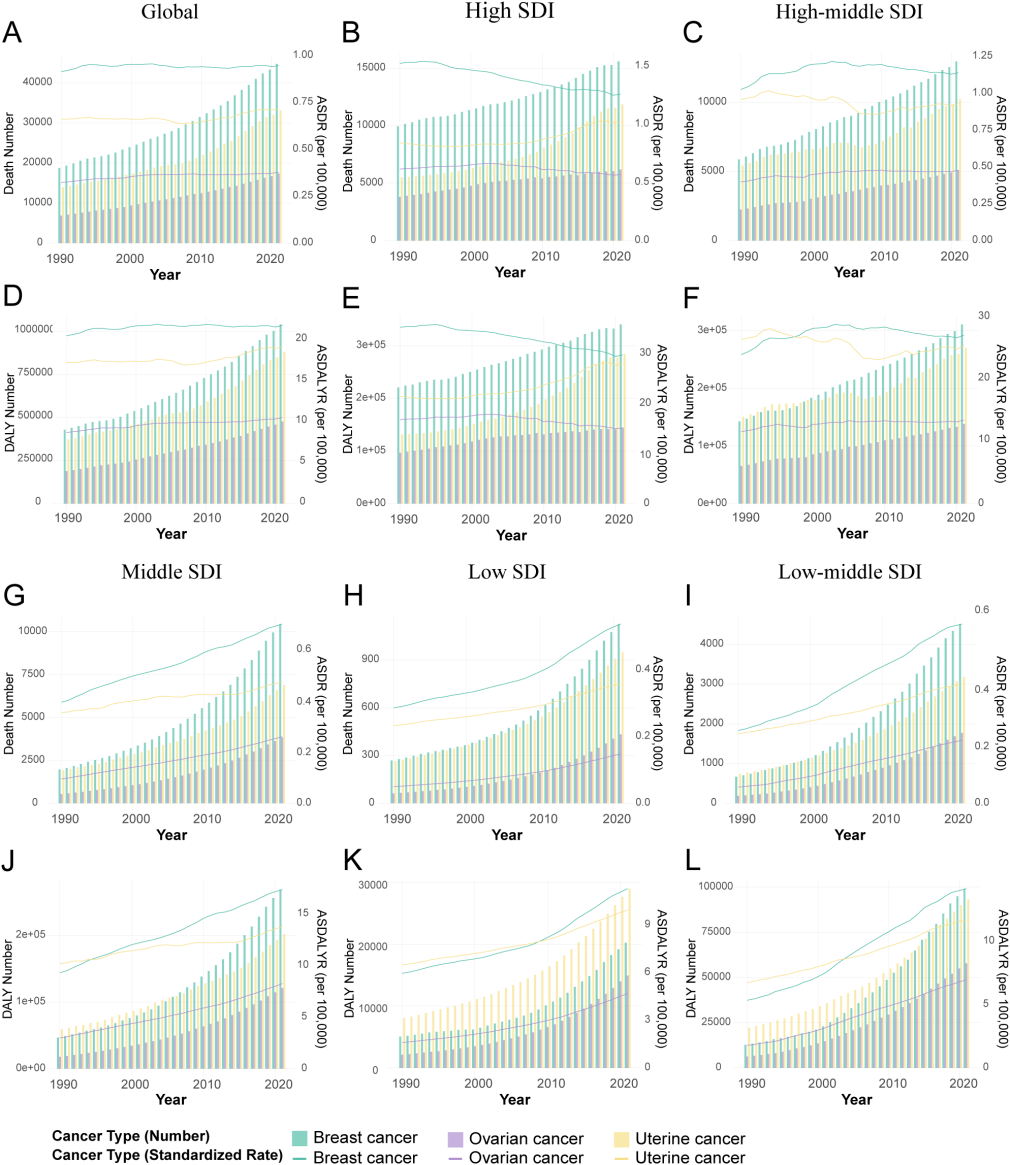
Temporal trends in the burden of female breast, ovarian, and uterine cancers attributable to high BMI by Socio-demographic Index (SDI) quintiles, 1990-2021. **(A)** Attributable deaths and ASDR for global. **(B)** Attributable deaths and ASDR for high SDI regions. **(C)** Attributable deaths and ASDR for high-middle SDI regions. **(D)** Attributable DALYs and ASDALYR for global. **(E)** Attributable DALYs and ASDALYR for high SDI regions. **(F)** Attributable DALYs and ASDALYR for high-middle SDI regions. **(G)** Attributable deaths and ASDR for middle SDI regions. **(H)** Attributable deaths and ASDR for low SDI regions. **(I)** Attributable deaths and ASDR for low-middle SDI regions. **(J)** Attributable DALYs and ASDALYR for middle SDI regions. **(K)** Attributable DALYs and ASDALYR for low SDI regions. **(L)** Attributable DALYs and ASDALYR for low SDI regions low-middle SDI regions.

**Figure 2:**
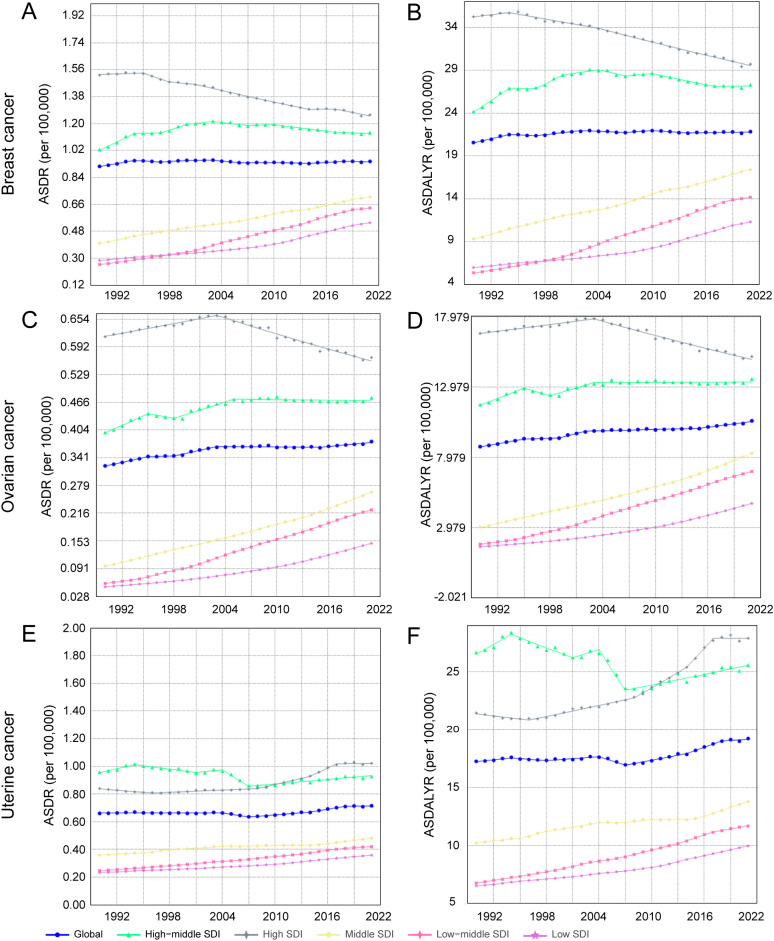
Joinpoint regression analysis of annual percent change (APC) in age-standardized rates of female breast, ovarian, and uterine cancers attributable to high body mass index (BMI), 1990-2021. **(A)** ASDR for breast cancer. **(B)** ASDALYR for breast cancer. **(C)** ASDR for ovarian cancer. **(D)** ASDALYR for ovarian cancer. **(E)** ASDR for uterine cancer. **(F)** ASDALYR for uterine cancer.

From 1990 to 2021, the global burden of breast, ovarian, and uterine cancers attributable to high BMI rose substantially, a trend observed in both age-standardized rates and absolute numbers (**Figure 1**, **Table 1**). The scale of this increase is stark: annual deaths more than doubled from approximately 36,000 to over 88,000, while associated DALYs surged by 157%, climbing from 830,000 to 2.13 million (**Supplementary Table S1**). This escalation underscores a significant and worsening global health challenge. Joinpoint regression analysis further elucidated the distinct growth trajectories and inflection points for each cancer (**Figure 2**, **Supplementary Tables S2**, **S3**). This analysis identified a high degree of trend volatility, particularly for breast cancer, which presented eight joinpoints in its ASDR trend. In comparison, the ASDR trends for ovarian and uterine cancer each exhibited four joinpoints. Ovarian cancer demonstrated the most consistent and rapid increase, with a significant AAPC in both age-standardized death rates (ASDR: 0.50%, 95% CI: 0.36–0.63) and DALY rates (ASDALYR: 0.61%, 95% CI: 0.50–0.72).

**Table 1:** The ASDR and ASDALYR attributable to high BMI in 1990 and 2021, and the AAPC from 1990 to 2021.

	1990		2021		1990-2021			
Location	ASDR (per100,000) No. (95%UI)	ASRDALY (per100,000) No. (95%UI)	ASDR (per100,000) No. (95%UI)	ASRDALY (per100,000) No. (95%UI)	ASDR (per100,000) No. (95%UI)	ASRDALY (per100,000) No. (95%UI)	ASDR AAPC No. (95%CI)	ASRDALY AAPC No. (95%CI)
Breast cancer								
Global	0.91 (-0.03,1.87)	20.55 (-0.86,41.7)	0.95 (-0.03,1.89)	21.83 (-1.01,42.51)	0.04 (-0.02,0.1)	0.06 (-0.01,0.13)	0.11 (-0.04, 0.26)	0.19 (0.05, 0.34)
High SDI	1.52 (-0.05,3.11)	35.25 (-1.46,70.82)	1.26 (-0.04,2.48)	29.71 (-1.11,58)	-0.17 (-0.22,-0.12)	-0.16 (-0.21,-0.09)	-0.64 (-0.84, -0.45)	-0.57 (-0.64, -0.49)
High-middle SDI	1.03 (-0.03,2.08)	24.17 (-1.07,48.35)	1.14 (-0.04,2.28)	27.31 (-0.97,52.79)	0.11 (0.01,0.23)	0.13 (0,0.3)	0.33 (0.22, 0.45)	0.38 (0.03, 0.74)
Middle SDI	0.4 (-0.01,0.79)	9.3 (-0.49,18.44)	0.71 (-0.02,1.45)	17.35 (-0.84,34.36)	0.77 (0.56,0.97)	0.87 (0.62,1.15)	1.88 (1.77, 1.99)	2.05 (1.94, 2.15)
Low-middle SDI	0.26 (-0.01,0.54)	5.34 (-0.63,11.2)	0.64 (-0.03,1.3)	14.15 (-1.24,28.39)	1.46 (1.14,1.86)	1.65 (1.17,2.26)	2.96 (2.74, 3.19)	3.22 (3.10, 3.34)
Low SDI	0.29 (-0.01,0.59)	5.96 (-0.76,12.51)	0.54 (-0.02,1.11)	11.29 (-1.16,22.86)	0.88 (0.58,1.28)	0.89 (0.52,1.41)	2.07 (1.99, 2.15)	2.09 (1.99, 2.19)
High-income Asia Pacific	0.24 (-0.01,0.48)	7.37 (-0.2,14.63)	0.42 (-0.01,0.85)	12.47 (-0.34,24.79)	0.76 (0.57,0.93)	0.69 (0.52,0.86)	1.82 (1.66, 1.98)	1.69 (1.53, 1.86)
High-income North America	2.03 (-0.06,4.03)	49.86 (-2.12,97.48)	1.61 (-0.06,3.12)	37.87 (-1.77,72.88)	-0.21 (-0.27,-0.14)	-0.24 (-0.3,-0.17)	-0.79 (-0.96, -0.62)	-0.91 (-1.07, -0.74)
Western Europe	1.74 (-0.06,3.54)	38.31 (-2.25,76)	1.43 (-0.04,2.94)	31.7 (-1.21,63.26)	-0.17 (-0.24,-0.11)	-0.17 (-0.26,-0.07)	-0.63 (-0.78, -0.48)	-0.64 (-0.81, -0.47)
Australasia	1.65 (-0.05,3.36)	38.36 (-1.96,76.57)	1.39 (-0.04,2.73)	32.15 (-1.35,62.68)	-0.16 (-0.27,-0.01)	-0.16 (-0.28,-0.01)	-0.58 (-1.02, -0.13)	-0.62 (-1.12, -0.12)
Andean Latin America	0.58 (-0.03,1.2)	12.07 (-1.29,25.61)	0.95 (-0.03,2.01)	21.45 (-1.64,45.43)	0.65 (0.24,1.23)	0.78 (0.24,1.72)	1.61 (1.04, 2.19)	1.94 (1.37, 2.51)
Tropical Latin America	0.92 (-0.03,1.85)	19.9 (-1.51,39.74)	1.19 (-0.04,2.38)	25.61 (-1.91,50.39)	0.29 (0.15,0.45)	0.29 (0.11,0.48)	0.87 (0.75, 0.99)	0.83 (0.70, 0.95)
Central Latin America	0.72 (-0.03,1.4)	14.75 (-1.25,29.13)	1.14 (-0.04,2.26)	26.15 (-2.05,51.79)	0.6 (0.38,0.85)	0.77 (0.48,1.19)	1.53 (1.24, 1.82)	1.83 (1.59, 2.07)
Southern Latin America	2.03 (-0.07,4.08)	43.83 (-2.36,85.49)	1.95 (-0.06,3.83)	41.99 (-1.84,80.97)	-0.04 (-0.15,0.1)	-0.04 (-0.18,0.14)	-0.10 (-0.41, 0.21)	-0.12 (-0.45, 0.22)
Caribbean	0.94 (-0.03,1.85)	20.1 (-1.72,39.77)	1.31 (-0.05,2.66)	28.16 (-2.45,55.69)	0.39 (0.2,0.61)	0.4 (0.17,0.72)	1.09 (0.64, 1.54)	1.11 (0.68, 1.54)
Central Europe	1.46 (-0.05,2.94)	32.23 (-1.84,63.57)	1.9 (-0.06,3.79)	42.71 (-1.54,84.25)	0.3 (0.17,0.46)	0.33 (0.14,0.54)	0.83 (0.67, 0.98)	0.89 (0.74, 1.03)
Eastern Europe	1.15 (-0.05,2.24)	27.01 (-2.06,52.27)	1.72 (-0.06,3.33)	41.12 (-1.59,79.47)	0.5 (0.27,0.75)	0.52 (0.21,0.93)	1.24 (0.69, 1.79)	1.37 (0.80, 1.95)
Central Asia	1.16 (-0.04,2.28)	26.86 (-2.22,52.37)	1.15 (-0.04,2.24)	26.92 (-1.54,51.56)	-0.02 (-0.14,0.13)	0 (-0.17,0.23)	-0.00 (-0.49, 0.49)	0.04 (-0.48, 0.57)
North Africa and Middle East	0.41 (-0.02,0.79)	9 (-0.99,17.64)	1.12 (-0.04,2.16)	25.49 (-1.97,49.92)	1.72 (1.32,2.19)	1.83 (1.26,2.82)	3.24 (2.99, 3.49)	3.35 (3.08, 3.62)
South Asia	0.13 (-0.01,0.28)	2.32 (-0.72,5.36)	0.38 (-0.02,0.77)	7.93 (-1.09,16.68)	1.93 (1.03,3.15)	2.41 (0.69,7.39)	3.55 (3.30, 3.80)	4.11 (3.82, 4.40)
Southeast Asia	0.33 (-0.01,0.67)	9.39 (-0.23,19.54)	0.8 (-0.02,1.7)	22.91 (-0.6,48.61)	1.47 (1.02,1.92)	1.44 (0.97,1.92)	2.96 (2.86, 3.05)	2.93 (2.82, 3.04)
East Asia	0.26 (-0.01,0.53)	7.15 (-0.19,14.9)	0.5 (-0.02,1.08)	14.54 (-0.47,31.06)	0.96 (0.4,1.65)	1.03 (0.44,1.8)	2.24 (2.12, 2.36)	2.36 (2.16, 2.56)
Oceania	1.21 (-0.03,2.54)	32.91 (-0.91,69.51)	1.67 (-0.05,3.42)	45.29 (-1.48,92.57)	0.39 (0.15,0.74)	0.38 (0.12,0.75)	1.03 (0.90, 1.16)	1.00 (0.86, 1.15)
Western Sub-Saharan Africa	0.62 (-0.02,1.22)	13.11 (-0.91,26.15)	1.38 (-0.04,2.71)	30.29 (-1.6,60.37)	1.22 (0.67,2.02)	1.31 (0.64,2.31)	2.61 (2.54, 2.67)	2.74 (2.66, 2.82)
Eastern Sub-Saharan Africa	0.35 (-0.01,0.72)	6.93 (-1.02,14.86)	0.73 (-0.03,1.51)	14.97 (-1.44,30.49)	1.1 (0.64,1.68)	1.16 (0.58,2.08)	2.42 (2.32, 2.52)	2.52 (2.38, 2.66)
Central Sub-Saharan Africa	0.36 (-0.01,0.8)	7.99 (-0.72,18.18)	0.83 (-0.02,1.86)	18.72 (-1.28,42.33)	1.29 (0.62,2.13)	1.34 (0.56,2.46)	2.71 (2.62, 2.79)	2.79 (2.71, 2.87)
Southern Sub-Saharan Africa	1.02 (-0.05,1.95)	19.51 (-1.88,39.25)	2.19 (-0.08,4.37)	46.54 (-3.18,90.11)	1.15 (0.69,1.94)	1.39 (0.72,2.76)	2.56 (2.14, 2.98)	2.82 (2.26, 3.39)
Ovarian cancer								
Global	0.32 (0.07,0.61)	8.72 (1.78,16.41)	0.38 (0.09,0.67)	10.56 (2.5,18.57)	0.17 (0.06,0.42)	0.21 (0.09,0.48)	0.50 (0.36, 0.63)	0.61 (0.50, 0.72)
High SDI	0.61 (0.13,1.15)	16.78 (3.57,31.36)	0.57 (0.14,1.01)	15.13 (3.79,26.82)	-0.08 (-0.15,0.08)	-0.1 (-0.17,0.07)	-0.31 (-0.36, -0.25)	-0.38 (-0.44, -0.32)
High-middle SDI	0.4 (0.09,0.75)	11.71 (2.49,21.84)	0.48 (0.12,0.85)	13.54 (3.26,24.13)	0.2 (0.06,0.41)	0.16 (0.02,0.38)	0.54 (0.26, 0.81)	0.42 (0.16, 0.69)
Middle SDI	0.1 (0.01,0.19)	2.99 (0.36,5.9)	0.26 (0.06,0.48)	8.25 (1.95,15.09)	1.73 (1.21,3.56)	1.76 (1.21,3.69)	3.31 (3.22, 3.40)	3.34 (3.25, 3.43)
Low-middle SDI	0.06 (0,0.12)	1.78 (0.13,3.74)	0.22 (0.04,0.41)	6.96 (1.43,12.58)	2.89 (1.59,6.45)	2.91 (1.62,6.27)	4.49 (4.31, 4.66)	4.49 (4.36, 4.63)
Low SDI	0.05 (0,0.11)	1.6 (0.01,3.56)	0.15 (0.02,0.29)	4.68 (0.74,9.04)	1.97 (-0.91,5.28)	1.93 (-0.38,4.8)	3.58 (3.53, 3.62)	3.54 (3.49, 3.59)
High-income Asia Pacific	0.07 (-0.01,0.17)	2.29 (-0.28,5.19)	0.12 (0.01,0.24)	3.5 (0.38,6.99)	0.58 (-1.26,2.47)	0.53 (-2.23,2.49)	1.50 (1.33, 1.68)	1.39 (1.17, 1.61)
High-income North America	0.83 (0.2,1.52)	23.36 (5.74,42.27)	0.78 (0.21,1.34)	20.45 (5.66,35.38)	-0.07 (-0.16,0.09)	-0.12 (-0.2,0.02)	-0.29 (-0.38, -0.20)	-0.52 (-0.68, -0.35)
Western Europe	0.63 (0.13,1.2)	17.18 (3.53,32.53)	0.58 (0.14,1.06)	14.7 (3.42,26.9)	-0.09 (-0.17,0.09)	-0.14 (-0.22,0.03)	-0.31 (-0.53, -0.08)	-0.52 (-0.78, -0.26)
Australasia	0.85 (0.18,1.58)	23.68 (5,43.97)	0.62 (0.16,1.1)	15.67 (4.12,27.86)	-0.28 (-0.38,-0.1)	-0.34 (-0.43,-0.17)	-0.94 (-1.84, -0.04)	-1.27 (-2.09, -0.45)
Andean Latin America	0.16 (0.03,0.33)	5.2 (0.93,10.8)	0.5 (0.12,0.97)	15.55 (3.86,29.85)	2.08 (1.2,3.9)	1.99 (1.13,3.74)	3.71 (3.35, 4.07)	3.61 (3.24, 3.98)
Tropical Latin America	0.28 (0.06,0.55)	8.52 (1.78,16.56)	0.46 (0.11,0.84)	13.52 (3.18,24.66)	0.6 (0.41,0.97)	0.59 (0.4,0.92)	1.53 (1.37, 1.70)	1.48 (1.29, 1.67)
Central Latin America	0.32 (0.07,0.59)	9.51 (2.09,17.81)	0.67 (0.19,1.21)	20.87 (5.99,37.44)	1.12 (0.77,1.67)	1.19 (0.84,1.73)	2.42 (2.10, 2.75)	2.55 (2.20, 2.90)
Southern Latin America	0.59 (0.14,1.12)	16.81 (3.87,31.61)	0.68 (0.18,1.2)	19.48 (5.21,34.22)	0.15 (-0.02,0.43)	0.16 (-0.01,0.44)	0.52 (0.25, 0.79)	0.48 (0.12, 0.84)
Caribbean	0.22 (0.05,0.42)	6.81 (1.44,12.82)	0.46 (0.11,0.84)	14.23 (3.43,26.1)	1.1 (0.8,1.8)	1.09 (0.77,1.74)	2.46 (2.11, 2.81)	2.45 (2.14, 2.76)
Central Europe	0.7 (0.16,1.3)	20.59 (4.65,38.13)	0.94 (0.24,1.71)	25.65 (6.53,46.23)	0.34 (0.2,0.51)	0.25 (0.12,0.43)	0.96 (0.88, 1.04)	0.73 (0.65, 0.81)
Eastern Europe	0.68 (0.16,1.21)	21.36 (4.99,37.8)	0.89 (0.24,1.54)	26.48 (6.87,45.59)	0.31 (0.14,0.56)	0.24 (0.06,0.49)	0.80 (0.23, 1.37)	0.62 (0.06, 1.19)
Central Asia	0.29 (0.06,0.54)	9.08 (2,16.79)	0.53 (0.13,0.95)	15.77 (3.76,28.49)	0.79 (0.54,1.17)	0.74 (0.49,1.11)	1.93 (1.61, 2.25)	1.84 (1.54, 2.15)
North Africa and Middle East	0.24 (0.05,0.49)	7.27 (1.49,14.33)	0.51 (0.15,0.89)	14.62 (4.22,25.6)	1.09 (0.37,1.93)	1.01 (0.3,1.8)	2.40 (2.33, 2.47)	2.26 (2.20, 2.32)
South Asia	0.03 (-0.01,0.07)	0.98 (-0.16,2.23)	0.16 (0.03,0.3)	5.11 (0.82,9.43)	4.6 (-7.6,20.7)	4.23 (-3.35,20.19)	5.75 (5.63, 5.87)	5.48 (5.40, 5.56)
Southeast Asia	0.05 (0,0.1)	1.67 (-0.06,3.64)	0.2 (0.04,0.38)	6.82 (1.27,12.97)	3.36 (-3.66,12.6)	3.09 (-1.88,10.62)	4.86 (4.79, 4.94)	4.65 (4.55, 4.76)
East Asia	0.03 (-0.02,0.08)	0.97 (-0.47,2.63)	0.16 (0.03,0.33)	4.86 (0.96,9.99)	4.14 (-17.23,26.5)	4 (-18.85,26.22)	5.44 (5.25, 5.63)	5.34 (5.16, 5.52)
Oceania	0.11 (0.02,0.22)	3.41 (0.66,6.95)	0.19 (0.05,0.34)	5.93 (1.54,11.06)	0.76 (0.34,1.39)	0.74 (0.31,1.34)	1.89 (1.77, 2.00)	1.85 (1.74, 1.96)
Western Sub-Saharan Africa	0.07 (0.01,0.14)	2.18 (0.34,4.04)	0.21 (0.05,0.41)	6.12 (1.3,11.8)	1.89 (0.76,3.71)	1.8 (0.79,3.49)	3.46 (3.36, 3.57)	3.37 (3.25, 3.48)
Eastern Sub-Saharan Africa	0.08 (0,0.17)	2.48 (0.02,5.26)	0.25 (0.04,0.49)	7.84 (1.32,15.4)	2.24 (-1.51,7.17)	2.16 (0.55,6.67)	3.87 (3.80, 3.94)	3.78 (3.72, 3.85)
Central Sub-Saharan Africa	0.05 (0,0.1)	1.35 (0.01,3.08)	0.18 (0.03,0.37)	5.55 (0.92,11.34)	3.07 (-0.68,8.61)	3.12 (0.71,8.79)	4.63 (4.59, 4.67)	4.67 (4.62, 4.71)
Southern Sub-Saharan Africa	0.36 (0.09,0.68)	10.58 (2.68,19.98)	0.81 (0.22,1.43)	23.2 (6.41,41.25)	1.28 (0.61,2)	1.19 (0.56,1.85)	2.67 (2.49, 2.84)	2.53 (2.34, 2.72)
Uterine cancer								
Global	0.66 (0.47,0.89)	17.26 (12.25,23.16)	0.72 (0.52,0.94)	19.23 (13.8,25.38)	0.08 (0.02,0.15)	0.11 (0.04,0.19)	0.23 (0.07, 0.40)	0.34 (0.16, 0.52)
High SDI	0.84 (0.6,1.14)	21.45 (15.47,28.69)	1.02 (0.74,1.34)	27.91 (20.5,36.15)	0.22 (0.15,0.29)	0.3 (0.22,0.38)	0.64 (0.49, 0.79)	0.86 (0.70, 1.03)
High-middle SDI	0.96 (0.68,1.28)	26.67 (18.82,35.51)	0.93 (0.66,1.22)	25.57 (18.19,33.72)	-0.03 (-0.11,0.06)	-0.04 (-0.12,0.05)	-0.07 (-0.46, 0.32)	-0.12 (-0.52, 0.28)
Middle SDI	0.36 (0.24,0.49)	10.18 (6.8,14.03)	0.48 (0.34,0.66)	13.78 (9.65,18.81)	0.34 (0.19,0.53)	0.35 (0.19,0.56)	0.95 (0.78, 1.12)	0.97 (0.81, 1.14)
Low-middle SDI	0.25 (0.17,0.34)	6.72 (4.63,9.14)	0.42 (0.29,0.57)	11.67 (7.97,15.63)	0.7 (0.5,0.95)	0.74 (0.51,1.02)	1.74 (1.59, 1.90)	1.80 (1.69, 1.91)
Low SDI	0.23 (0.15,0.33)	6.49 (4.28,9.15)	0.36 (0.23,0.51)	9.94 (6.52,14.4)	0.54 (0.28,0.9)	0.53 (0.25,0.93)	1.41 (1.34, 1.48)	1.40 (1.32, 1.49)
High-income Asia Pacific	0.26 (0.19,0.35)	6.9 (4.99,9.28)	0.31 (0.22,0.42)	9.07 (6.39,12.08)	0.19 (0.06,0.35)	0.32 (0.16,0.5)	0.52 (0.34, 0.71)	0.87 (0.68, 1.06)
High-income North America	1.05 (0.74,1.42)	28.14 (20.15,37.38)	1.57 (1.12,2)	44.48 (32.54,56.04)	0.49 (0.39,0.59)	0.58 (0.47,0.69)	1.30 (1.01, 1.59)	1.50 (1.20, 1.80)
Western Europe	0.8 (0.57,1.08)	19.99 (14.25,26.73)	0.92 (0.65,1.23)	23.28 (16.87,30.96)	0.14 (0.06,0.22)	0.16 (0.09,0.24)	0.41 (0.17, 0.64)	0.47 (0.27, 0.67)
Australasia	0.65 (0.46,0.88)	16.82 (11.69,23.01)	0.88 (0.63,1.16)	22.97 (16.43,30.23)	0.35 (0.17,0.58)	0.37 (0.18,0.59)	1.03 (0.50, 1.56)	1.07 (0.53, 1.61)
Andean Latin America	1.18 (0.78,1.65)	33.39 (22.12,47.15)	1.21 (0.78,1.81)	33.29 (21.33,49.61)	0.03 (-0.22,0.32)	0 (-0.25,0.3)	0.19 (-0.56, 0.96)	0.11 (-0.70, 0.93)
Tropical Latin America	0.84 (0.58,1.14)	21.4 (15,29.29)	0.92 (0.64,1.24)	24.1 (16.93,32.02)	0.1 (-0.01,0.21)	0.13 (0.02,0.23)	0.32 (0.17, 0.47)	0.36 (0.20, 0.52)
Central Latin America	0.69 (0.48,0.92)	18.03 (12.71,24.03)	0.91 (0.63,1.23)	25.35 (17.88,34.21)	0.32 (0.15,0.52)	0.41 (0.22,0.64)	0.97 (0.56, 1.39)	1.14 (0.85, 1.42)
Southern Latin America	1.03 (0.73,1.4)	26.85 (18.86,36.2)	0.86 (0.6,1.14)	21.99 (15.49,28.84)	-0.16 (-0.26,-0.05)	-0.18 (-0.28,-0.06)	-0.55 (-0.91, -0.19)	-0.63 (-1.02, -0.24)
Caribbean	1.07 (0.74,1.43)	30.86 (21.27,40.87)	1.92 (1.34,2.6)	53.69 (37.87,71.73)	0.79 (0.54,1.09)	0.74 (0.48,1.02)	1.96 (1.14, 2.80)	1.88 (1.00, 2.76)
Central Europe	1.48 (1.04,1.96)	38.88 (27.5,51.36)	1.72 (1.23,2.3)	43.7 (31.44,58.21)	0.16 (0.04,0.28)	0.12 (0.01,0.25)	0.42 (0.09, 0.75)	0.34 (-0.02, 0.71)
Eastern Europe	1.64 (1.17,2.17)	48.12 (34.34,63)	2.1 (1.47,2.75)	60.22 (42.49,79.04)	0.28 (0.13,0.45)	0.25 (0.11,0.43)	0.81 (0.35, 1.27)	0.81 (-0.16, 1.78)
Central Asia	1.31 (0.92,1.74)	37.69 (26.66,50)	1.11 (0.77,1.47)	31.2 (21.83,41.64)	-0.16 (-0.25,-0.04)	-0.17 (-0.28,-0.06)	-0.59 (-1.45, 0.28)	-0.67 (-1.52, 0.18)
North Africa and Middle East	0.49 (0.33,0.71)	13.52 (8.99,19)	0.62 (0.42,0.83)	17.04 (11.51,22.61)	0.27 (0.01,0.61)	0.26 (0.01,0.62)	0.76 (0.64, 0.88)	0.71 (0.61, 0.81)
South Asia	0.11 (0.07,0.15)	2.96 (2.02,4.15)	0.23 (0.16,0.35)	6.56 (4.4,9.56)	1.2 (0.71,1.88)	1.22 (0.73,1.91)	2.61 (2.43, 2.80)	2.63 (2.51, 2.75)
Southeast Asia	0.22 (0.14,0.31)	6.84 (4.32,9.75)	0.43 (0.27,0.6)	13.52 (8.19,18.87)	1.01 (0.66,1.43)	0.98 (0.62,1.41)	2.27 (2.16, 2.39)	2.23 (2.10, 2.35)
East Asia	0.27 (0.17,0.4)	8.26 (5.08,12.38)	0.33 (0.2,0.51)	10.23 (6.19,15.84)	0.22 (-0.13,0.73)	0.24 (-0.12,0.76)	0.64 (0.51, 0.78)	0.68 (0.56, 0.80)
Oceania	0.85 (0.5,1.33)	25.26 (14.91,39.56)	1.16 (0.62,1.74)	34.48 (18.34,53.45)	0.38 (-0.01,1)	0.36 (-0.03,1.02)	1.03 (0.94, 1.12)	1.01 (0.86, 1.15)
Western Sub-Saharan Africa	0.3 (0.2,0.45)	8 (5.25,11.51)	0.54 (0.35,0.77)	13.41 (8.62,19.25)	0.77 (0.4,1.29)	0.68 (0.33,1.17)	1.85 (1.77, 1.92)	1.66 (1.57, 1.74)
Eastern Sub-Saharan Africa	0.28 (0.17,0.4)	7.82 (4.62,11.09)	0.45 (0.28,0.68)	12.15 (7.6,18.73)	0.59 (0.2,1.12)	0.55 (0.16,1.13)	1.51 (1.45, 1.56)	1.43 (1.36, 1.51)
Central Sub-Saharan Africa	0.27 (0.17,0.41)	7.4 (4.75,11.16)	0.52 (0.3,0.86)	14.16 (8.23,23.17)	0.94 (0.3,1.79)	0.91 (0.28,1.81)	2.17 (2.13, 2.21)	2.13 (2.08, 2.17)
Southern Sub-Saharan Africa	0.63 (0.42,0.92)	16.45 (11.11,23.76)	1.29 (0.83,1.72)	32.41 (21.25,42.91)	1.04 (0.47,1.55)	0.97 (0.46,1.47)	2.30 (1.83, 2.77)	2.17 (1.84, 2.50)

The trends for uterine and breast cancer were more complex. The burden from uterine cancer, while increasing overall (AAPC for ASDALYR: 0.34%, 95% CI: 0.16–0.52), followed a non-linear path. A brief but significant decline from 2004–2007 (APC: -1.49%) was reversed by a subsequent phase of accelerated growth, particularly from 2014–2018 (APC: 1.53%), suggesting a reversal of earlier gains in prevention or control. Breast cancer presented a different dynamic: while its ASDR remained stable (AAPC not statistically significant), its ASDALYR rose significantly (AAPC: 0.20%, 95% CI: 0.07–0.34) (**Figures 2A, B**). This divergence suggests that while fatal outcomes have been mitigated, the overall health loss has increased, likely reflecting a combination of rising incidence and longer survival times for patients living with the disease.

#### 3.1.2 Overview and temporal trends of disease burden attributed to high BMI in female cancers across different SDI regions

Stratification by SDI revealed profound heterogeneity and a distinct epidemiological transition across regions (**Figure 1**, **Table 1**). In high-SDI regions, a notable divergence emerged. Substantial progress was made in controlling the burden of breast and ovarian cancers, with their respective ASDALYR declining significantly (AAPC: -0.57% and -0.38%). This success, however, was juxtaposed with a persistent and accelerating rise in the burden of uterine cancer. Its fluctuating ASDR trend was characterized by four joinpoints, which marked multiple shifts in trajectory (**Figure 2D**; **Supplementary Table S2**). Its ASDALYR increased from 21.45 to 27.91 per 100,000 between 1990 and 2021 (AAPC: 0.86%) (**Table 1**).

This divergent pattern was also evident in high-middle SDI regions. Here, the burdens of breast and ovarian cancer declined significantly, with their respective ASDRs falling by an average of 0.64% (95% CI: -0.84 to -0.45) and 0.31% (95% CI: -0.36 to -0.25) annually. Conversely, the burden of uterine cancer rose, with its ASDR and ASDALYR increasing by 0.64% and 0.86% per year (AAPC), respectively. This upward trend was also highly volatile. The identification of four distinct joinpoints in its ASDR trend confirmed this instability, which included a sharp but temporary decline from 2004 to 2007 (APC: -4.23%) before reversing into a period of sustained growth (**Figure 2C**, **Supplementary Table S2**).

In sharp contrast to the complex trends in high-income regions, the burden of these three cancers showed a consistent, sustained, and statistically significant upward trend in low- and middle-SDI regions. This pattern highlights a concerning reality: the burden of cancers attributable to high BMI is rapidly shifting toward lower-income countries. This growth was particularly alarming in the lowest-SDI regions. Ovarian cancer saw the most dramatic rise, with its age-standardized death rate increasing at an average annual percentage change (AAPC) of 4.49% (95% CI: 4.31, 4.66), one of the highest growth rates observed in the analysis. Breast cancer (AAPC: 2.96%) and uterine cancer (AAPC: 1.73%) also exhibited extremely rapid increases. Joinpoint analysis confirmed the severity of this situation. In these low-SDI regions, the burden for three cancers increased continuously throughout the entire study period. For instance, the ASDR for breast cancer presented six joinpoints and ovarian cancer presented three; however, every single segment across these points showed a statistically significant increase (**Supplementary Table S2**). Crucially, the analysis found no signs of a slowdown or reversal, indicating that these nations will face substantial and growing pressure on their cancer prevention and control systems.

#### 3.1.3 The burden of female cancers related to high BMI and their temporal trends in 21 large geographical regions

Analysis by GBD region revealed geographic complexities that transcend simple SDI stratification, particularly within high-income areas. The high-income Asia-Pacific region, for instance, emerged as a notable outlier. It defied the general high-SDI trend by exhibiting a sharp increase in its breast cancer burden (ASDR AAPC: 1.82%), a stark contrast to the declines observed in Western Europe (AAPC: -0.72%) and Australasia. Meanwhile, the burden of uterine cancer continued its ascent across nearly all high-income regions, including high-income North America (AAPC: 1.40%) and Australasia (AAPC: 1.11%). This widespread increase reinforces the distinct and persistent challenge posed by uterine cancer, even in the most developed settings.

Conversely, ASDRs for these three cancers surged across most middle- and low-income regions, including South Asia, Southeast Asia, North Africa, the Middle East, and sub-Saharan Africa. This trend was most severe in South Asia, where the ovarian cancer ASDR climbed at a globally unprecedented rate of 5.86% annually (95% CI: 5.75–5.97), accompanied by substantial increases for breast (AAPC: 3.60%) and uterine cancer (AAPC: 2.58%). Joinpoint analysis further highlighted the unrelenting nature of this surge in South Asia, revealing that despite multiple joinpoints in the trend, every distinct period showed statistically significant and rapid growth. Similarly, the breast cancer burden accelerated rapidly in North Africa and the Middle East (AAPC: 3.87%). However, the analysis also uncovered critical exceptions to this pattern. Defying the global trend, uterine cancer ASDR significantly declined in both Southern South America (AAPC: -0.54%) and Central Asia (AAPC: -0.55%). These anomalous declines highlight an urgent need to investigate the unique protective factors or successful regional interventions that may be driving them.

### 3.2 Geographic and regional distribution of cancers in women attributable to high BMI

**Figure 3:**
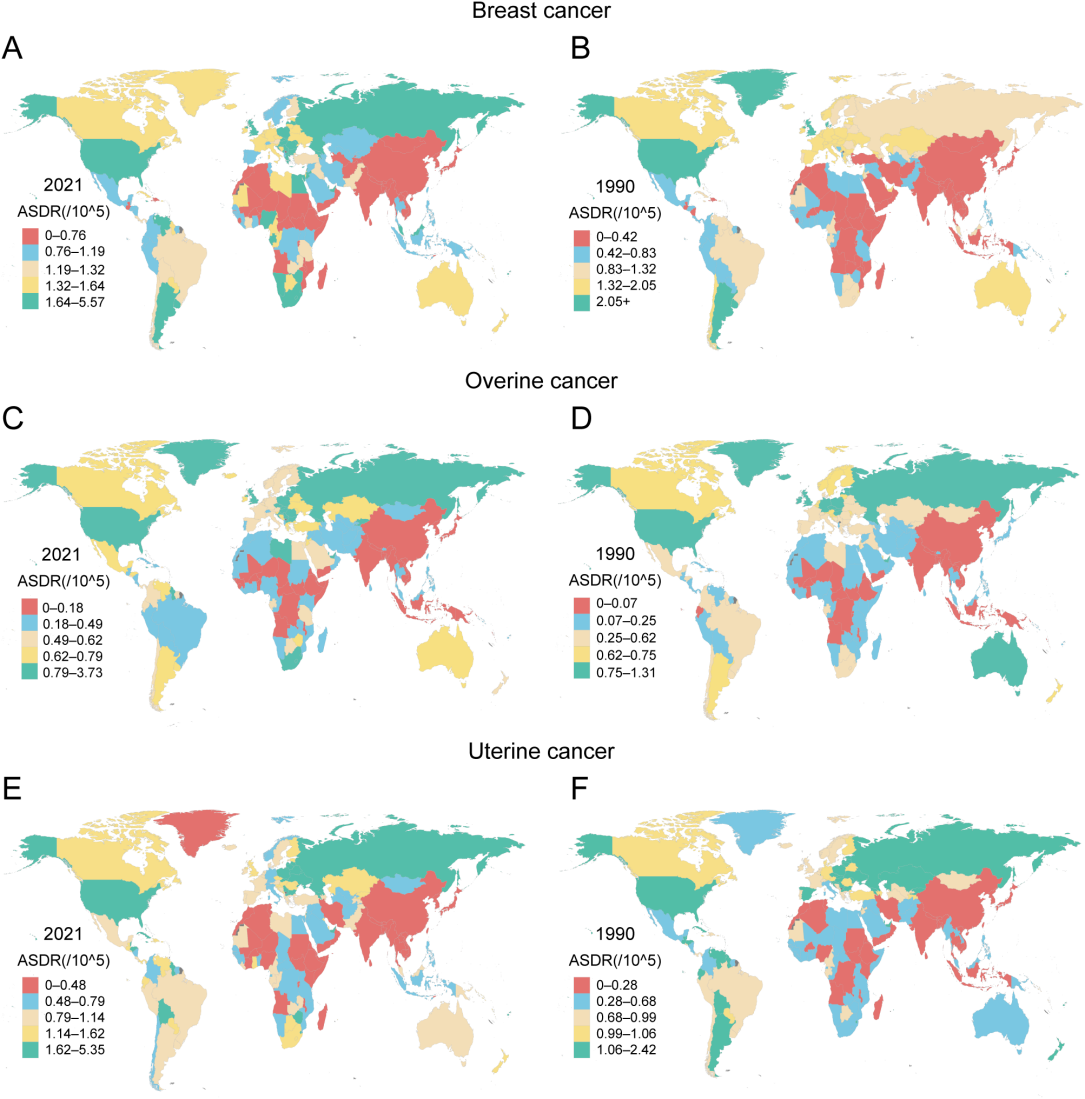
Geographical distribution of ASDR attributable to high BMI for female breast, ovarian, and uterine cancers across 204 countries and territories in 1990 and 2021 **(A)** ASDR of breast cancer attributed to high BMI in 2021. **(B)** ASDR of breast cancer attributed to high BMI in 1990. **(C)** ASDR of ovarian cancer attributed to high BMI in 2021. **(D)** ASDR of ovarian cancer attributed to high BMI in 1990. **(E)** ASDR of uterine cancer attributed to high BMI in 2021. **(F)** ASDR of uterine cancer attributed to high BMI in 1990.

The geographic distribution of the high-BMI-attributable cancer burden shifted dramatically between 1990 and 2021, with distinct patterns for each cancer type (**Figure 3**, **Supplementary Figure S1**, **Supplementary Table S4**). In 1990, the highest burdens of breast and ovarian cancer were concentrated in high-income regions like North America, Western Europe, and Australasia. In contrast, the uterine cancer burden was highest in Eastern Europe, Central Asia, and parts of Latin America (**Supplementary Table S4**). By 2021, this landscape had fundamentally changed, characterized by a transfer of burden from some high-income nations to many low- and middle-income countries. This shift is exemplified by ovarian cancer. While Australia saw its ASDR decrease from 0.87 (95% UI: 0.19–1.63) to 0.63 (95% UI: 0.17–1.13) per 100,000, Angola experienced a more than fivefold increase in its ASDR from 0.03 (95% UI: 0–0.09) to 0.16 (95% UI: 0.02–0.34) per 100,000. A similar divergence occurred in DALYs, with Australia’s rate falling from 24.26 to 16.08 per 100,000 while Angola’s surged nearly fivefold from 1.07 to 5.02 per 100,000 (**Figure 3A**, **Supplementary Table S4**). A comparable trend was evident for breast cancer. In Bangladesh, for instance, the ASDALYR increased a striking ninefold from 0.16 to 1.44 per 100,000, highlighting a rapidly escalating threat even in a region with a historically low absolute burden (**Supplementary Figure S1**, **Supplementary Table S4**).

Uterine cancer displayed a distinct geographic pattern, with its high-BMI-attributable burden increasing across most income levels by 2021. This upward trend was evident even in high-income regions. In Canada, the ASDR rose from 0.99 (95% UI: 0.69–1.36) to 1.14 (95% UI: 0.80–1.53) per 100,000, while American Samoa experienced a more pronounced increase from 1.58 (95% UI: 0.87–2.33) to 2.55 (95% UI: 1.09–4.94) per 100,000. The escalation was even more dramatic in many middle-income regions. Cuba, for example, saw its ASDR nearly double from 1.30 (95% UI: 0.90–1.76) to 2.41 (95% UI: 1.66–3.33) per 100,000, with its corresponding DALY rate surging by 74% from 38.16 to 66.30 per 100,000. However, this trend was not universal. Argentina provided a key counterexample, where the ASDR for uterine cancer significantly declined from 1.12 (95% UI: 0.79–1.54) to 0.93 (95% UI: 0.65–1.24) per 100,000 (**Figure 3E**, **Supplementary Table S4**). This heterogeneity suggests that the factors driving the high-BMI-related uterine cancer burden are complex and operate differently across regions, even those with similar economic profiles.

### 3.3 Relationship between disease burden and SDI

#### 3.3.1 Breast cancer attributable to high BMI: the relationship with SDI and the efficiency frontier

**Figure 4:**
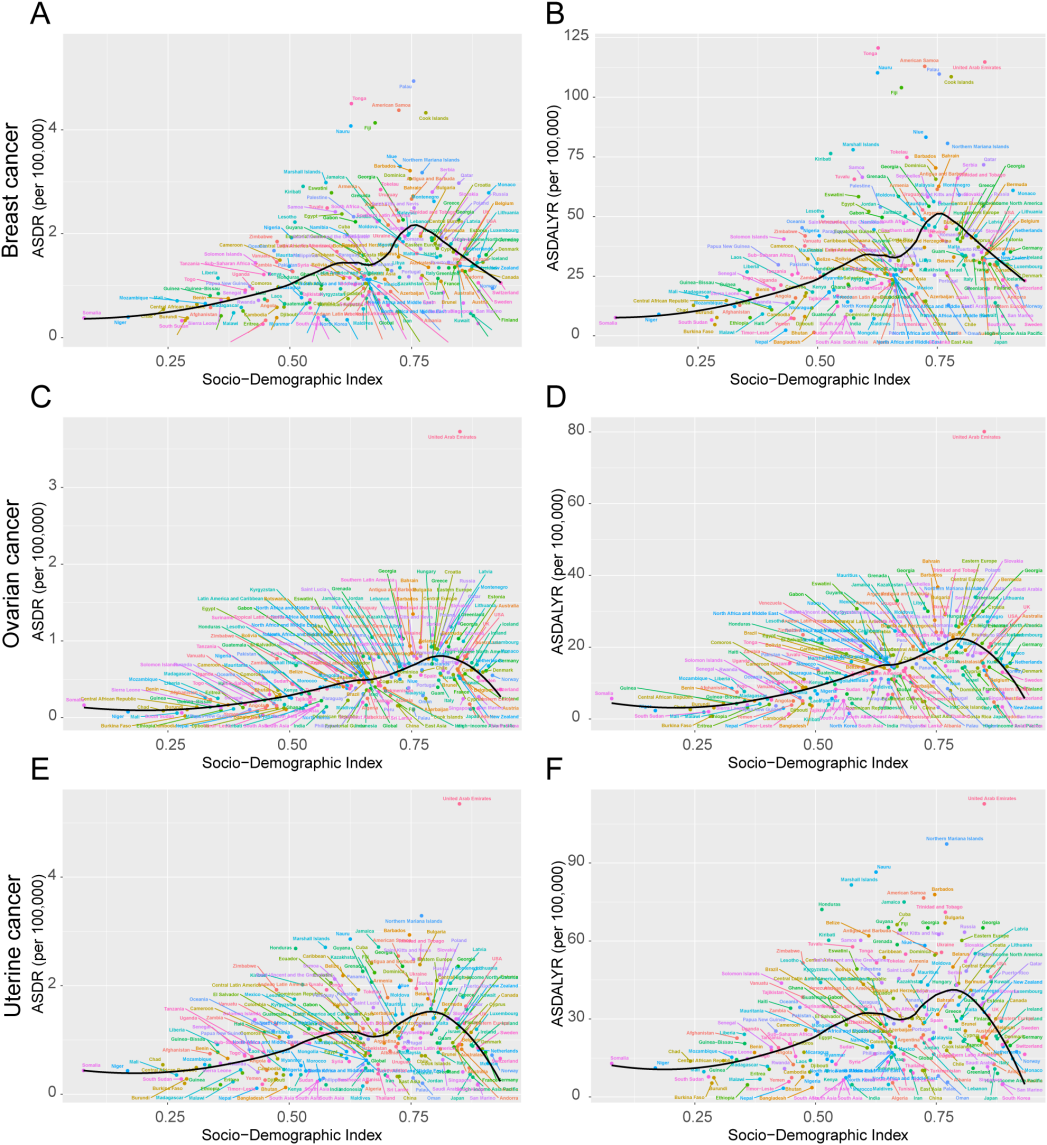
ASDR and ASDALYR attributable to high BMI for breast cancer, ovarian cancer, and uterine cancer across 204 countries and territories by SDI in 2021. **(A)** ASDR for breast cancer. **(B)** ASDALYR for breast cancer. **(C)** ASDR for ovarian cancer. **(D)** ASDALYR for ovarian cancer. **(E)** ASDR for uterine cancer. **(F)** ASDALYR for uterine cancer. Each plot includes dot clusters and curves, indicating trends across the Socio-Demographic Index. Colored dots represent different data points.

**Figure 5:**
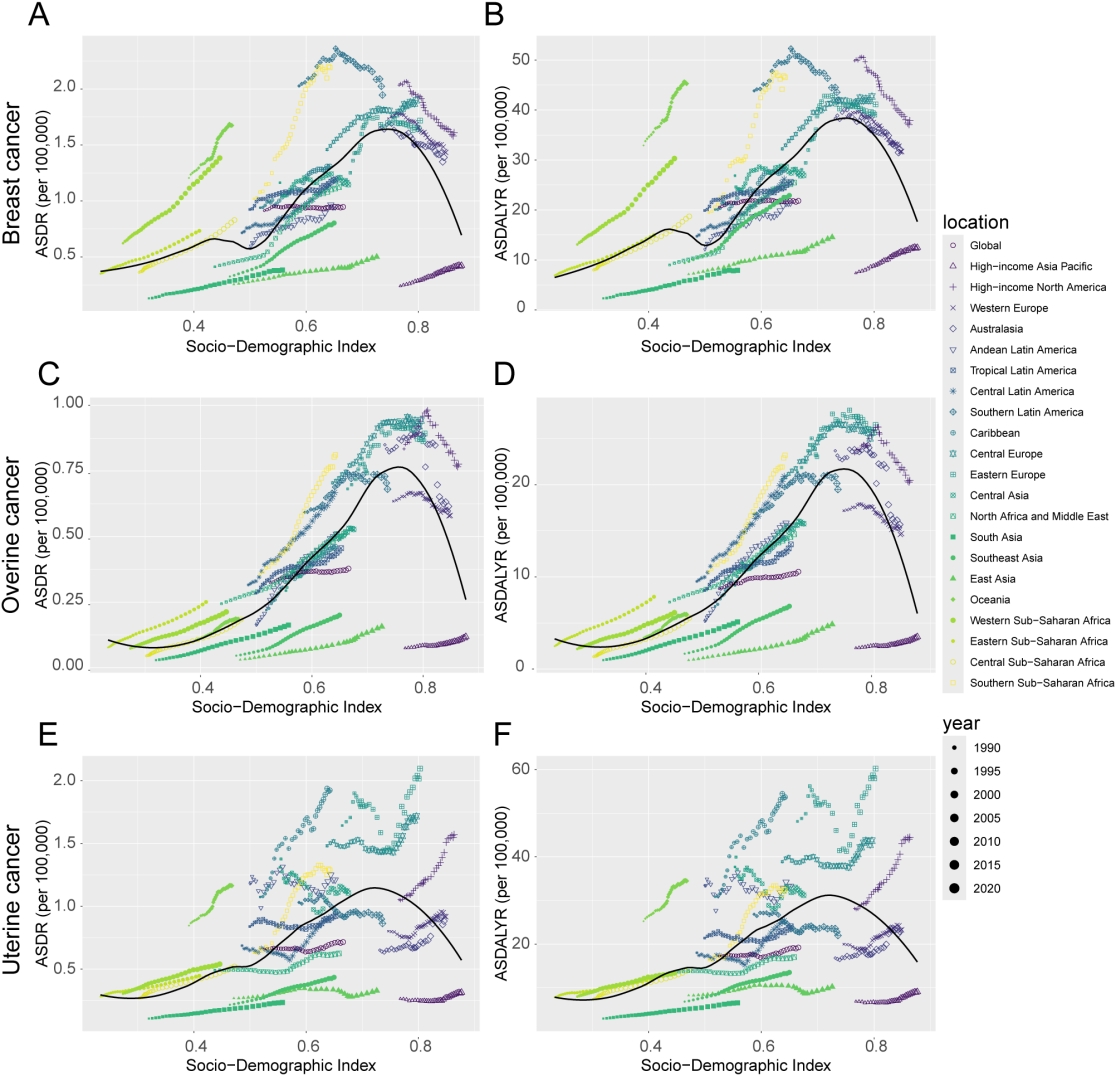
Trends in ASDR and ASDALYR for female breast cancer, ovarian cancer, and uterine cancer attributable to high BMI in 21 regions categorized by SDI from 1990 to 2021. **(A)** ASDR for breast cancer. **(B)** ASDALYR for breast cancer. **(C)** ASDR for ovarian cancer. **(D)** ASDALYR for ovarian cancer. **(E)** ASDR for uterine cancer. **(F)** ASDALYR for uterine cancer.

The high-BMI-attributable breast cancer burden showed a strong positive correlation with the SDI, forming a J-shaped curve where the burden accelerates sharply in regions with an SDI above 0.7 (**Figures 4A, B**). Crucially, the entire curve representing the expected burden shifted upward between 1990 and 2021, indicating a universal increase in risk at all development levels (**Figures 5A, B**). This relationship was evident in 2021, with high-SDI territories like American Samoa (ASDR: 4.38 per 100,000) and Barbados (ASDR: 3.21 per 100,000) exhibiting the highest burdens. Conversely, low-SDI countries such as Somalia and Bangladesh reported the lowest absolute burdens (ASDR: 0.10 per 100,000 for both) (**Supplementary Table S4**).

**Figure 6:**
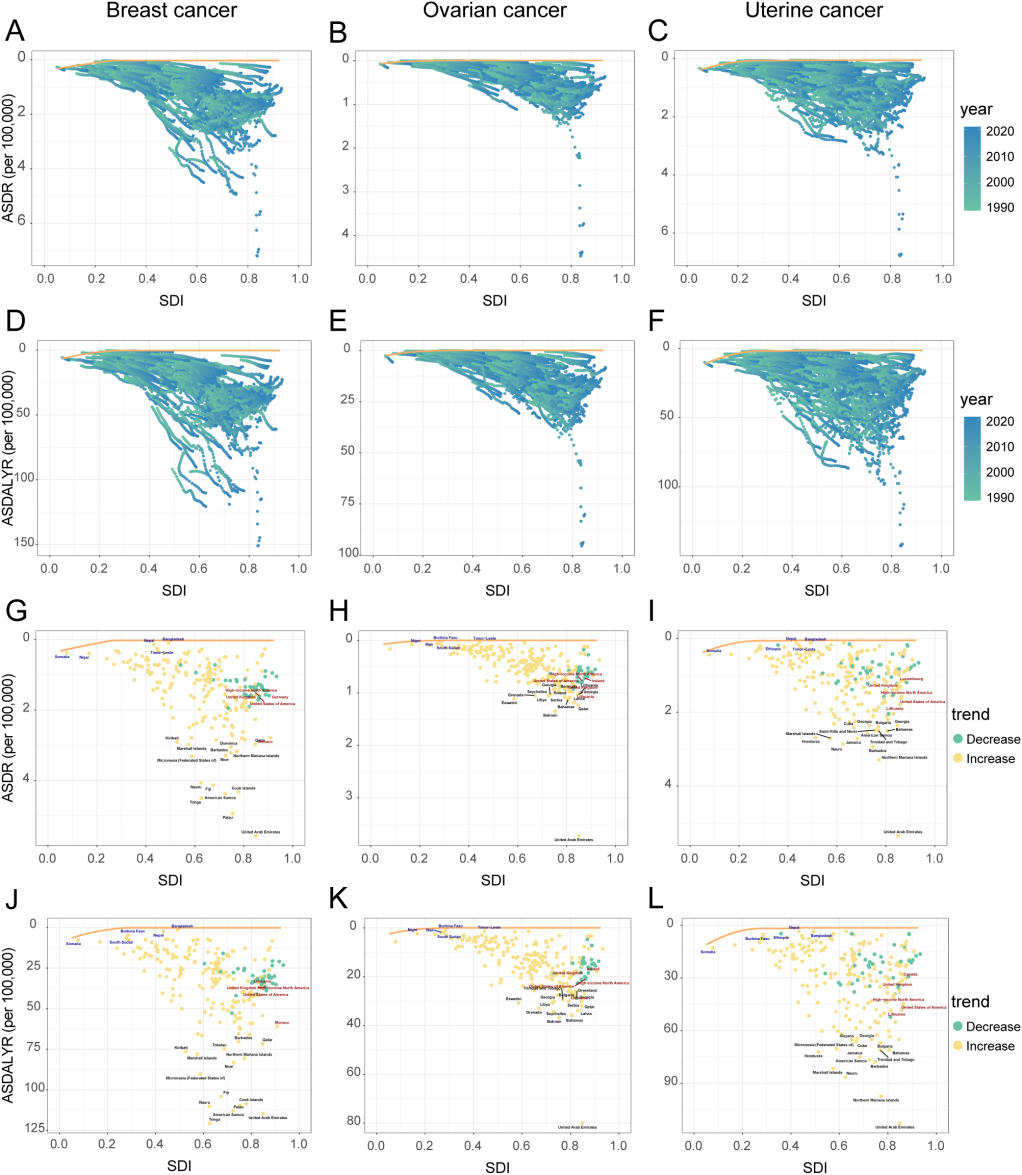
Relationship and temporal trends of age-standardized rates of female breast, ovarian, and uterine cancers attributable to high BMI with SDI in 204 countries and territories, 1990-2021. Panels **(A–C)**: ASDR, and ASDALYR **(D–F)** for breast cancer, ovarian cancer, and uterine cancer from 1990 to 2021, plotted against SDI. Points are colored by year (light to dark for 1990-2021). The yellow line represents the frontier curve. Panels: ASDR **(G–I)**, and ASDALYR **(J–L)** for breast cancer, ovarian cancer, and uterine cancer in 2021, plotted against SDI. Points are colored by trend (green for decrease, orange for increase). Selected countries are labeled.

The country efficiency frontier, which represents the optimal achievable outcome for cancer control, shifted upward between 1990 and 2021 (**Figures 6A, D, G, J**). This indicates an improvement in the global benchmark. However, the performance of individual nations varied widely, creating a widening gap between the highest- and lowest-performing countries. This divergence is most evident at the country level. Several high-SDI countries successfully reduced their burden, moving closer to the ideal frontier. For instance, the United Kingdom’s ASDR fell from 2.16 to 1.56 per 100,000, while the United States saw a similar decline from 2.13 to 1.64 per 100,000 **Figure 6G**. Conversely, many low-SDI countries, including Bangladesh, Somalia, and Nepal, experienced a significant rise in their cancer burden, causing them to fall further behind the benchmark for effective control (**Figure 6D**, **Supplementary Table S6**). This trend was also consistent at the regional level. Western Europe, a predominantly high-SDI region, saw a decrease in its overall burden. In stark contrast, Sub-Saharan Africa experienced a significant increase, further underscoring the growing global disparity in cancer control (**Supplementary Table S5**).

#### 3.3.2 Ovarian cancer attributable to high BMI: the relationship with SDI and the efficiency frontier

The high-BMI-attributable ovarian cancer burden was strongly and positively correlated with the SDI, and the expected burden at every SDI level increased between 1990 and 2021 (**Figures 4C, D**; **Figures 5C, D**). This direct relationship was reflected in 2021 country-level data, where high-SDI nations like Bahrain (ASDR: 1.35 per 100,000) and the Bahamas (ASDR: 1.13 per 100,000) reported some of the highest burdens **Figure 6H**. Conversely, the lowest burdens were found in low-SDI countries such as Burkina Faso (ASDR: 0.02 per 100,000) and Chad (ASDR: 0.09 per 100,000) (**Supplementary Table S4**).

Despite this overall trend, an analysis of the efficiency frontier revealed divergent trajectories in country-level performance (**Figures 6B, E**). Several high-SDI countries successfully reduced their burden, moving closer to the optimal frontier; these included the United Kingdom (ASDR decreased from 0.81 to 0.61 per 100,000) and the United States (ASDR decreased from 0.81 to 0.64 per 100,000). In stark contrast, the burden escalated in many low-SDI nations. This escalation pushed countries like Burkina Faso, Mali, and South Sudan further from the frontier, signifying a growing gap between their actual and potential health outcomes (**Supplementary Table S6**). This divergence was also evident at a regional scale, with the burden decreasing in Australasia while increasing significantly in South Asia (**Supplementary Table S5**).

#### 3.3.3 Uterine cancer attributable to high BMI: the relationship with SDI and the efficiency frontier

The high-BMI-attributable burden of uterine cancer exhibited a unique U-shaped relationship with SDI, with the highest burdens observed at both the lowest and highest ends of the development spectrum and a lower burden in the middle-SDI range (approx. 0.5–0.7) (**Figures 4E, F**). Over time, this curve shifted upward and flattened between 1990 and 2021, indicating a rising burden even in the historically lower-risk middle-SDI regions (**Figures 5E, F**). This U-shaped pattern was evident in 2021, with high burdens seen in high-SDI countries like Barbados (ASDR: 2.94 per 100,000) and Cuba (ASDR: 2.41 per 100,000), as well as in low-SDI countries like Somalia (ASDR: 0.81 per 100,000) (**Supplementary Table S4**).

In a notable departure from the trends for breast and ovarian cancer, the burden of uterine cancer also worsened in many high-SDI nations. This increase occurred even as the efficiency frontier shifted upward, indicating a widespread negative trend (**Figures 6C, F**). For example, despite their high development status, the United States (ASDR rising from 1.11 to 1.41 per 100,000), the United Kingdom (from 0.86 to 1.08 per 100,000), and Canada (from 0.99 to 1.14 per 100,000) all experienced significant increases in their ASDR (**Figures 6I**, **Supplementary Table S6**). This concerning trend was confirmed at a regional level, with the burden rising across High-income North America and Australasia (**Supplementary Table S5**).

### 3.4 Age-specific patterns and decomposition of disease burden changes

#### 3.4.1 Age-specific death and DALY rates

**Figure 7:**
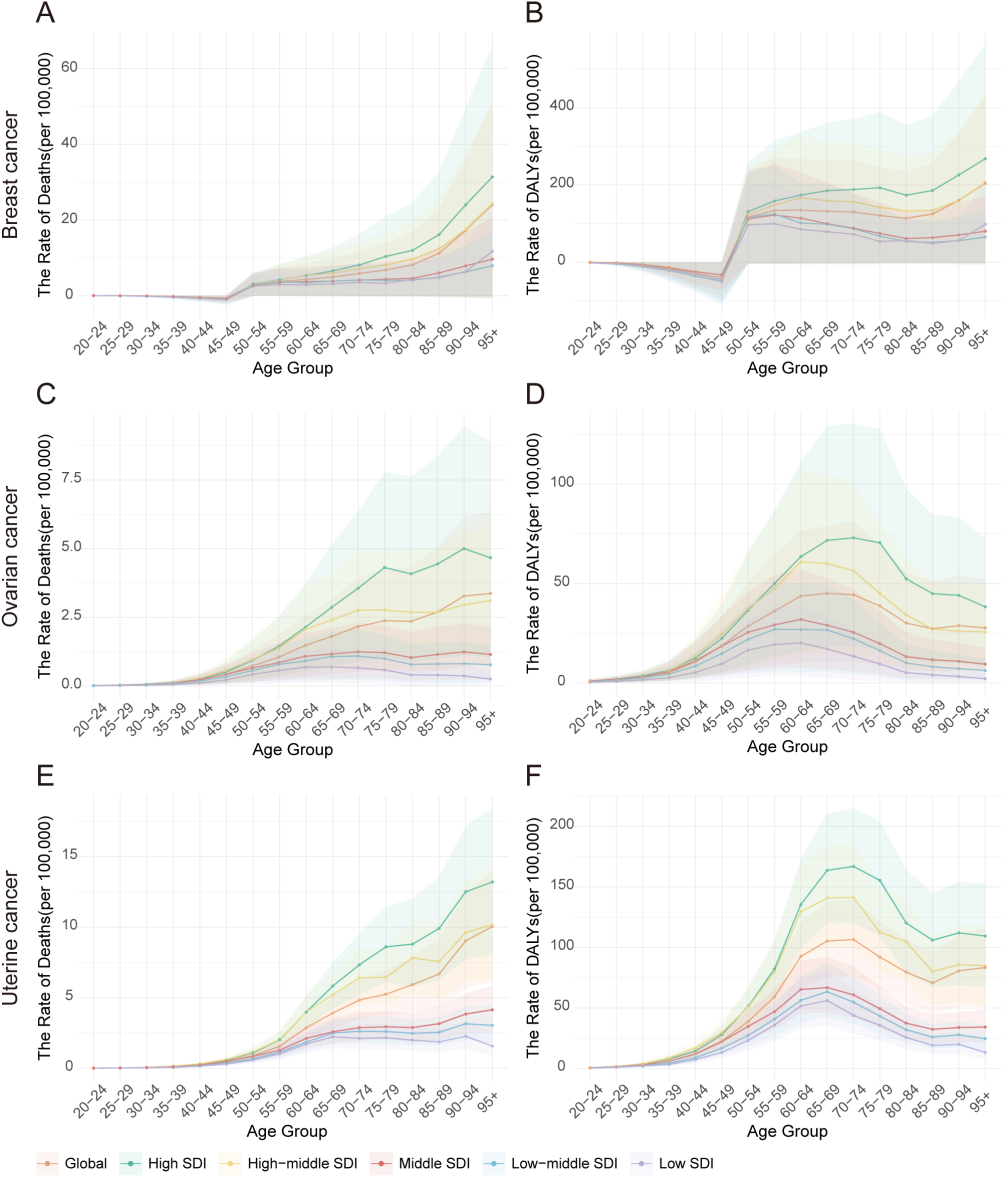
Age-specific death rates and DALY rates of high BMI-attributable female breast, ovarian, and uterine cancers by Socio-Demographic Index (SDI) quintiles, 2021. **(A)** Distribution of breast cancer deaths by age group in 2021. **(B)** Distribution of breast cancer DALYs by age group in 2021. **(C)** Distribution of ovarian cancer deaths by age group in 2021. **(D)** Distribution of ovarian cancer DALYs by age group in 2021. **(E)** Distribution of uterine cancer deaths by age group in 2021. **(F)** Distribution of uterine cancer DALYs by age group in 2021.

Globally, the age-specific burden attributable to high BMI varied significantly by cancer type in 2021 (**Figure 7**, **Supplementary Table S7**). The burden of breast cancer was unique, appearing only after age 50 and then rising sharply to a death peak in the 95+ age group (23.99 per 100,000) (**Figure 7A**). In contrast, the attributable burden for ovarian and uterine cancer was present across all age groups and increased steadily with age (**Figures 7C, E**). However, their peaks for health loss (DALYs) occurred earlier than their death peaks. For ovarian cancer, the DALY rate peaked in the 65–69 age group, whereas for uterine cancer, it peaked in the 70–74 age group, even though death for both cancers was highest in the 95+ age group (**Figures 7D, F**).

Analysis by SDI quintile revealed that this burden was consistently and substantially higher in high-SDI regions across nearly all age groups. For instance, the DALY rate for breast cancer in the ≥95 age group was nearly three times higher in high-SDI regions (267.65 per 100,000) than in low-SDI regions (98.26 per 100,000). Similarly, the peak DALY rate for uterine cancer in the 70–74 age group was almost four times higher in high-SDI regions (167.05 per 100,000) compared to low-SDI regions (43.92 per 100,000). Interestingly, within low-SDI regions, a distinct pattern emerged among the oldest age group (≥95 years), where the death rate from uterine cancer (1.57 per 100,000) was significantly higher than that of ovarian cancer (0.25 per 100,000) but much lower than that of breast cancer (11.70 per 100,000) (**Supplementary Table S7**).

#### 3.4.2 Decomposition of the drivers of changes in disease burden

**Figure 8:**
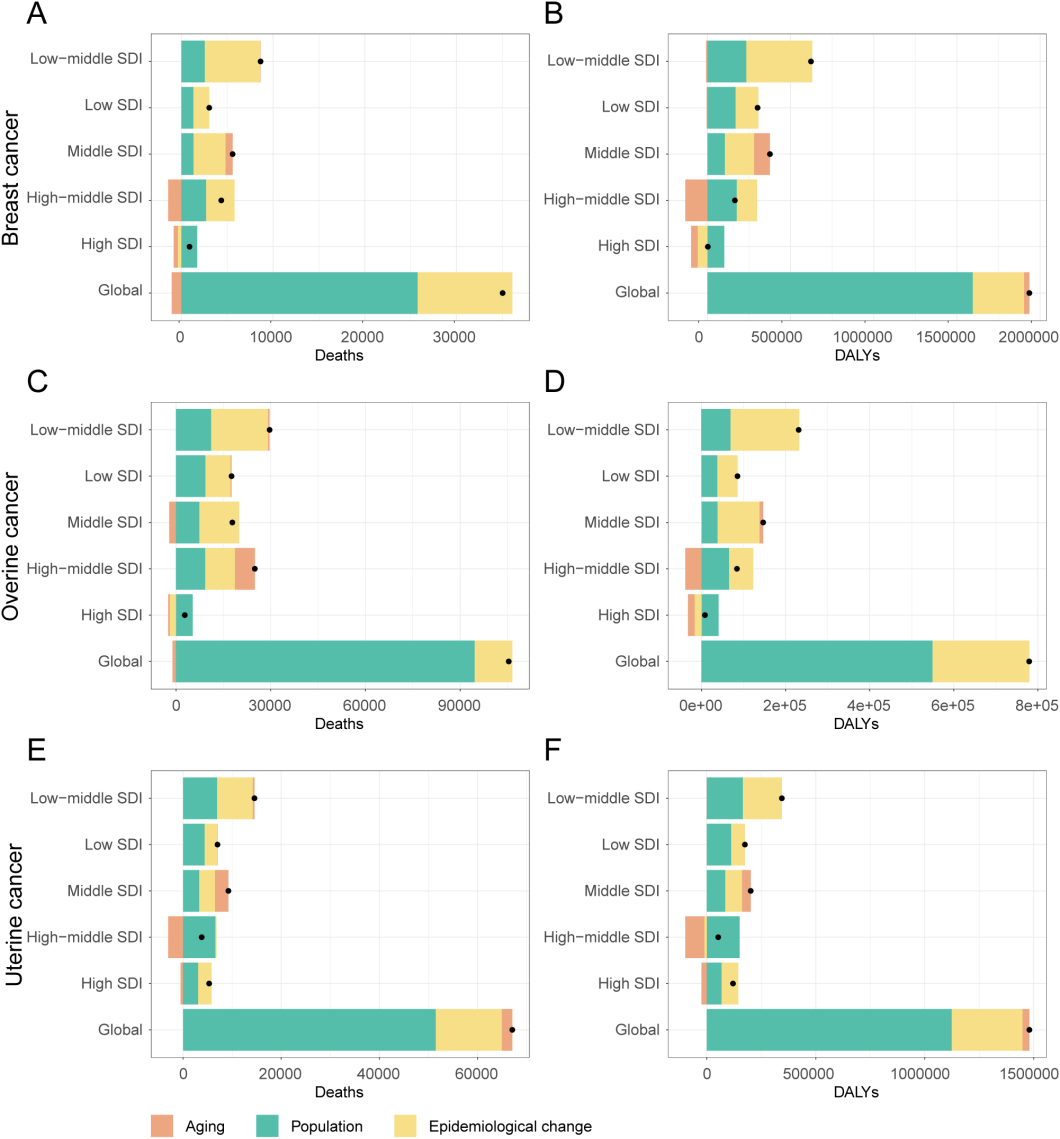
Decomposition of changes in deaths and DALYs attributable to high BMI for female breast, ovarian, and uterine cancers by SDI quintiles, 1990-2021. **(A)** Decomposition analysis of breast cancer death rate, 1990-2021. **(B)** Decomposition analysis of breast cancer dally rate, 1990-2021. **(C)** Decomposition analysis of ovarian cancer death rate, 1990-2021. **(D)** Decomposition analysis of ovarian cancer daly rate, 1990-2021. **(E)** Decomposition analysis of uterine cancer death rate, 1990-2021. **(F)** Decomposition analysis of uterine cancer daly rate, 1990-2021.

Between 1990 and 2021, the absolute burden of breast, ovarian, and uterine cancer attributable to high BMI increased substantially worldwide. A decomposition analysis revealed that this increase was primarily driven by population growth, which accounted for over 70% of the rise in DALYs for each cancer (**Figure 8**, **Supplementary Table S8**). Worsening epidemiological trends (i.e., changes in age-specific rates) were the second-largest contributor to the increased burden for three cancers. The impact of population aging was more complex, helping to decrease breast cancer death while contributing to a rise in uterine cancer death.

This global pattern, however, masked starkly different trends in high-SDI regions, where public health successes were evident for breast and ovarian cancer. For these two cancers, both improvements in age-specific rates (epidemiological changes) and demographic shifts in aging populations successfully drove down the burden, leading to a combined decrease of nearly 100,000 DALYs for breast cancer alone. In stark contrast, these same high-SDI regions failed to control the burden of uterine cancer. Here, worsening epidemiological factors remained a primary driver of the increase in DALYs (+76,540), completely offsetting any potential gains from demographic shifts.

The situation in low-SDI regions was entirely different and more uniformly challenging. Here, the burden of three cancers was propelled upward by the dual forces of rapid population growth and worsening epidemiological trends. Unlike in high-SDI regions, epidemiological changes in low-SDI countries led to a substantial increase in DALYs for breast (+136,906), ovarian (+48,248), and uterine (+62,278) cancer. Furthermore, population aging played a minimal role in these changes. As expected, middle-SDI regions exhibited an intermediate pattern, with both population growth and adverse epidemiological changes contributing to a rising burden across the board.

### 3.5 Health inequalities in female cancers attributable to high BMI

**Figure 9:**
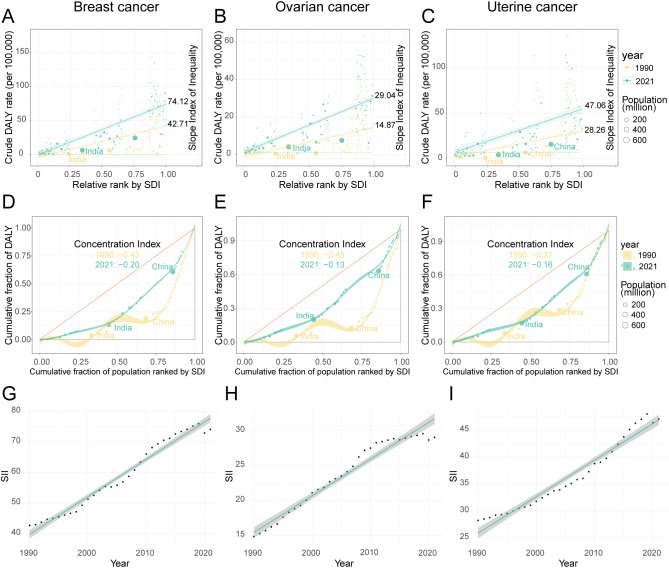
The inequality index of female breast, ovarian, and uterine cancers attributable to high BMI across 204 countries and territories in 1990 and 2021. **(A–C)** show scatter plots of crude DALY rates for breast, ovarian, and uterine cancers against relative rank by SDI, with slope indexes indicating inequality. **(D–F)** depict concentration curves for the cumulative fraction of DALYs by population ranked by SDI, highlighting shifts in concentration indexes from 1990 to 2021 in countries like India and China. Data points are color-coded by year and bubble size represents population. **(G–I)** display line graphs of SII trends over time for the same cancers, illustrating increases between 1990 and 2021.

The analysis revealed a key paradox: while relative inequality in the high-BMI-attributable cancer burden gradually alleviated between 1990 and 2021, absolute inequality persistently worsened. This widening absolute gap was confirmed by the Slope Index of Inequality (SII), which remained positive and increased steadily for three cancers throughout the study period, with only a brief dip in 2020 (**Supplementary Table S9**, **Figures 9G–I**). Visually, this is represented by a markedly steeper positive slope on regression plots comparing DALY rates to SDI levels in 2021 versus 1990 (**Figures 9A–C**). Among the three cancers, breast cancer consistently exhibited the largest absolute disparity. Its SII value grew by 73.5%, rising from 42.71 (95% CI: 33.93–51.48) in 1990 to 74.12 (95% CI: 62.55–85.70) in 2021. Notably, while ovarian cancer had the smallest absolute gap, its disparity grew the fastest. The SII for ovarian cancer surged by 95.3% (from 14.87 to 29.04), indicating that the absolute DALY burden gap between the highest- and lowest-SDI regions expanded more rapidly for this cancer than for the others.

In sharp contrast to the widening absolute gap, indicators of relative inequality pointed toward a converging trend. This was measured by the CI, where a negative value signifies that the disease burden is disproportionately concentrated in high-SDI populations—a “pro-rich” inequality. Over the past three decades, however, this relative inequality has substantially diminished, as the CI for three cancers moved closer to zero. The change was most pronounced for ovarian cancer, with its CI increasing from -0.45 to -0.13, followed by breast cancer (from -0.43 to -0.20) and uterine cancer (from -0.27 to -0.16). This convergence was visually confirmed by Lorenz curves, which moved closer to the line of equality in 2021 compared to 1990 for three cancers (**Figures 9D–F**). The practical implication of this shift is significant. For example, in 1990, the wealthiest 25% of the global population (by SDI) shouldered approximately 60% of the ovarian cancer DALY burden; by 2021, this share had fallen to a more equitable 40%.

### 3.6 Future burden projections of female cancers attributable to high BMI

**Figure 10:**
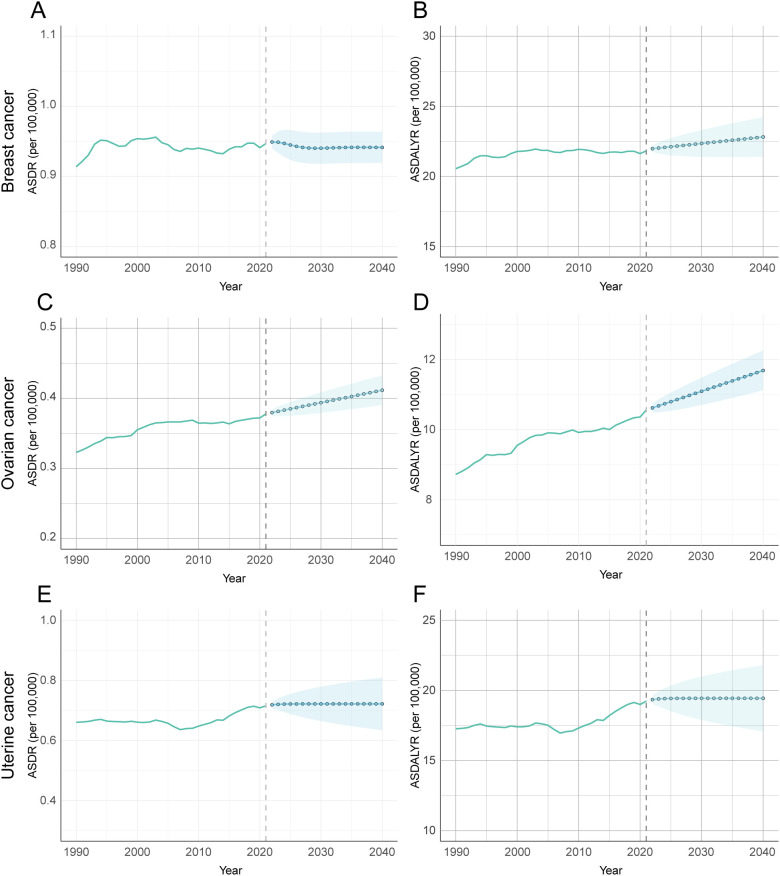
Forecasted trends of age-standardized DALY and death rates attributable to high BMI for female breast, ovarian, and uterine cancers using ARIMA model, 1990-2040. **(A)** Prediction analysis of breast cancer ASDR. **(B)** Prediction analysis of breast cancer ADALYR. **(C)** Prediction analysis of ovarian cancer ASDR. **(D)** Prediction analysis of ovarian cancer ADALYR. **(E)** Prediction analysis of uterine cancer ASDR. **(F)** Prediction analysis of uterine cancer ADALYR.

Projections based on an ARIMA model of historical data (1990–2021) indicate that the global, age-standardized burden of these three cancers attributable to high BMI is not expected to decrease by 2040. Instead, the model forecasts three distinct trajectories: a significant increase for ovarian cancer, a divergent pattern for breast cancer, and a plateau for uterine cancer (**Figure 10**, **Supplementary Table S10**).

The burden of ovarian cancer is projected to see the most pronounced and unambiguous increase. Both its ASDR and ASDALY rate are forecast to rise significantly. The ASDR is expected to climb from 0.378 per 100,000 in 2021 to 0.412 in 2040 (95% CI: 0.390–0.433), while the ASDALY rate will rise from 10.560 to 11.687 per 100,000 (95% CI: 11.121–12.253). This clear upward trajectory suggests that ovarian cancer will pose a growing public health challenge. In contrast, breast cancer is projected to follow a divergent pattern. Its ASDR is expected to remain stable through 2040, with no statistically significant change from its 2021 rate of 0.947 per 100,000 (**Figure 10A**). This stability in death, however, likely masks a rising burden of incidence and disability. This suggests that while death may be controlled, the number of women living with breast cancer attributable to high BMI will continue to grow. Finally, the attributable burden of uterine cancer is forecast to plateau, with no statistically significant change in either its ASDR or ASDALY rate. However, these projections carry a high degree of uncertainty. The prediction intervals for uterine cancer are notably wide (**Figures 10E, F**), reflecting significant variability in the historical data and making its future trend the least certain of the three.

## 4 Discussion

Using GBD 2021 data, this study provides a comprehensive assessment of the global burden of female breast, ovarian, and uterine cancers attributable to high BMI from 1990 to 2021, detailing its dynamic trends, driving factors, and escalating health inequalities. We found that this burden is substantial, growing, and highly unequal, mirroring the unabated rise of the global obesity pandemic (32, 33). Crucially, the burden has not only risen in absolute terms but has also followed divergent trajectories across regions of varying socioeconomic development, exposing profound structural inequities in global health.

A notable divergence in the burden of breast cancer is evident even within high-SDI regions. While the burden in Western Europe and Australasia is stabilizing or declining, the Asia-Pacific region shows a contrasting and robust upward trend. This disparity is likely attributable to a complex interplay between lifestyle factors, such as regional dietary habits, and established racial and ethnic determinants (34). Consistent with this, significant variations in incidence trends across ethnic groups have been documented (35). These variations are hypothesized to stem from fundamental differences in age-related risk factors and carcinogenic pathways among ancestries (36).

Our analysis revealed a second marked divergence in disease burden trajectories within these regions. Specifically, the ASDR for breast and ovarian cancers attributable to high BMI has declined, a success likely attributable to robust health systems featuring organized screening, advanced diagnostics, and standardized treatments (37, 38). However, this reduction in death starkly contrasts with the sustained rise in ASDALYR. This paradox suggests that while treatments are extending survival, they may also prolong the period of morbidity, with patients burdened by both cancer-related sequelae and metabolic comorbidities. Concurrently, the unabated rise in the burden of uterine cancer further challenges the effectiveness of current prevention strategies in mitigating these metabolic risks (39).

In stark contrast, low- and middle-SDI regions faced a widespread and sustained increase in the burden of three cancers. This geographic shift is rooted in a fundamental mismatch: rapid nutritional transitions and urbanization have fueled a surge in obesity, while health systems remain ill-equipped for effective prevention, early diagnosis, and treatment. This growing gap between rising risk exposure and inadequate health system capacity is the primary driver of the worsening cancer burden in these parts of the world.

Our inequality analysis further illuminated this divergence. A consistently rising SII demonstrates a widening absolute gap in DALY rates between the highest- and lowest-SDI regions. This widening gap, alongside a concentration of the burden in lower-SDI regions (as shown by the Concentration Index), is rooted in a profound structural inertia within health systems. Historically, health systems in low- and middle-SDI regions were architected to combat infectious diseases, maternal death, and malnutrition. Their funding, infrastructure, and workforce were geared toward acute interventions. However, globalization has driven rapid nutritional transitions and urbanization, causing obesity-related cancer risks to surge at a pace that has far outstripped the evolution of this legacy infrastructure. This has created a fundamental mismatch: the acute care models effective for infectious diseases are ill-suited for managing chronic NCDs, which demand sustained screening, risk management, and long-term follow-up. This systemic disconnect, compounded by severe shortages of diagnostic technology, specialist physicians, and standardized protocols, directly translates into delayed diagnoses and poorer treatment outcomes, thereby inflating DALYs and exacerbating the disease burden precisely in the regions least equipped to handle it.

The sharp deterioration in ovarian cancer inequality, with the SII increasing by 95.3%, warrants special attention as it exemplifies a cascade of disparities across risk, diagnosis, and treatment. This cascade begins with risk factors, as obesity and metabolic syndrome surge in low- and middle-SDI regions. It is compounded at the diagnostic level, where the insidious early symptoms of ovarian cancer require a level of public awareness, clinical vigilance, and imaging access that is often absent, leading to late-stage diagnoses. This diagnostic dilemma is exacerbated by social determinants like education, as lower educational attainment is linked to barriers in health literacy and navigating care, resulting in more advanced disease at presentation (40–42). Finally, at the treatment level, effective management of ovarian cancer combines complex specialized surgery and platinum-based chemotherapy with expensive targeted agents, such as PARP inhibitors, which have significantly improved patient prognosis (43, 44). In middle- and low-SDI regions, severely limited access to these technical and costly therapies creates substantial survival disparities, which in turn accelerates absolute inequality at an alarming pace. Furthermore, integrating age-specific analyses with biological mechanisms offers key physiological insights into the burden patterns of different cancers. While the attributable burden for three cancers generally rises with age, breast cancer exhibits a distinctive life-course pattern. The burden is negative in the 20–49 age group, potentially reflecting a protective effect of high BMI on certain premenopausal breast cancer subtypes (45). However, this reverses dramatically after menopause, when adipose tissue becomes the primary source of estrogen and age-related metabolic dysfunction elevates risk, causing the burden to spike (46, 47). Furthermore, emerging evidence suggests that poor metabolic health in young adulthood (20–54 years) may be driving an earlier onset of these cancers, shifting the burden toward younger, more socially productive populations (48, 49). Underpinning these trends are the multidimensional biological links between high BMI and female cancers. High BMI is a complex disruptor, promoting carcinogenesis through chronic inflammation, endogenous hormone dysregulation (particularly elevated estrogen), insulin resistance, and abnormal adipokine secretion (50, 51). Crucially, high BMI rarely acts in isolation. It frequently coexists with other metabolic abnormalities as part of a broader metabolic syndrome, creating synergistic and amplifying effects that further exacerbate the overall disease burden (52, 53). Importantly, the causality of these epidemiologically observed associations has been robustly substantiated by Mendelian randomization (MR) studies. Employing genetic variants as instrumental variables, these analyses confirm a causal link between elevated BMI and an increased risk of breast, uterine, and ovarian cancers. This powerful methodology effectively precludes bias from many potential confounders, strengthening the evidence for a direct biological relationship (54).

Our ARIMA model projects a sustained global increase in the death and DALY rates for female cancers attributable to high BMI through 2040. This forecast is rooted in the persistence of three key drivers. First, the unchecked global obesity epidemic ensures a continuously expanding population at risk. Second, the significant lag time between high BMI exposure and cancer onset means the full carcinogenic impact of recent and ongoing obesity trends has yet to materialize. Finally, persistent structural disparities in health system capacity mean that low and middle-SDI regions will remain ill-equipped to manage the rising caseload, further exacerbating death and disability.

Therefore, addressing the disproportionate health and economic burdens borne by women requires a multi-faceted strategy. At the individual level, interventions must promote proactive lifestyle modifications, including plant-based diets and personalized weight management, to mitigate excess adiposity during critical periods like the postpartum phase (55). Concurrently, enhancing adherence to cancer screening among high-risk populations is critical for early detection and treatment (56). However, these individual efforts are insufficient alone and require synergistic support from community and clinical systems. This systemic support includes strengthening health literacy, ensuring equitable access to nutritious food, and applying biomarkers like sex hormone levels to inform more precise, timely interventions. Broader, systemic changes are also essential. Health systems must integrate weight management into routine maternal and child healthcare and existing cancer screening pathways. At the macro-policy level, governments should implement strong fiscal and regulatory measures, such as taxes on sugar-sweetened beverages and mandatory front-of-pack nutrition labeling, to reshape the food environment and curb the obesity epidemic at its source. Ultimately, these structural interventions are fundamental to narrowing the widening gap in health inequalities.

The primary strength of this study lies in its use of the GBD 2021 database. As the most comprehensive global dataset available, it provides standardized data spanning three decades across 204 countries and territories, which ensures the high generalizability and comparability of our findings. A second strength is our application of multiple advanced statistical methods, including Joinpoint regression, frontier efficiency analysis, and ARIMA forecasting. These methods allowed for a multidimensional analysis of the trends, drivers, and inequalities of the female-specific cancer burden, providing robust, forward-looking data to support public health policymakers. It is important to acknowledge several limitations when interpreting the attribution of the female cancer burden to a high BMI in the GBD 2021 estimates. Firstly, as model-based estimates, the GBD data are subject to inherent uncertainty. Secondly, the use of aggregated data for the cancers studied precluded subtype-specific analyses, although these subtypes may exhibit distinct risk factor associations (57, 58). A primary limitation of this study is its reliance on BMI as the sole measure of adiposity. BMI is a crude proxy that does not fully capture the nuanced metabolic effects of other components of metabolic syndrome, such as central obesity, dyslipidemia, hypertension, and insulin resistance. Consequently, this approach may underestimate the collective contribution of these factors to cancer pathogenesis and obscure the precise mechanisms linking adiposity to cancer. Attributing the disease burden solely to BMI may therefore overlook the synergistic effects of these metabolic derangements. Furthermore, this analysis did not incorporate key lifestyle factors, including specific dietary patterns, alcohol consumption, oral contraception, or physical activity levels (59). The exclusion of these variables is a significant concern because they are established determinants of cancer risk, with extensive evidence underscoring the substantial influence of specific dietary patterns (60, 61), alcohol consumption (62), and physical activity (63, 64). Consequently, residual confounding due to these unmeasured lifestyle determinants could bias the estimated burden attributable to high BMI. Finally, the ARIMA model projects future trends based on historical patterns. Therefore, it cannot fully account for the potential impact of major, unforeseen public health interventions or societal shifts.

## 5 Conclusion

This study reveals a rising and increasingly unequal global burden of breast, ovarian, and uterine cancers attributable to high BMI. This finding underscores the urgent need to integrate nutritional strategies, dietary interventions, and lifestyle management into precision-based prevention and control systems. To be effective, such interventions must be differentiated for varying SDI levels, scalable, cost-effective, and implemented in synergy with advances in screening and treatment. The immediate goal is to reduce death and disability rates (ASDR and ASDALY). The ultimate aim, however, is to narrow the absolute inequality gap, thereby alleviating the global burden of these cancers and their profound socioeconomic consequences.

## Data Availability

The datasets presented in this study can be found in online repositories. The names of the repository/repositories and accession number(s) can be found in the article/**Supplementary Material**.
