# Supplementary material for: High BMI-attributable female-specific cancers: a comprehensive analysis of the global disease burden and trends from 1990 to 2021 and projections to 2040
Source: Front Oncol. 2025 Oct 29;15:1704299. doi: 10.3389/fonc.2025.1704299 (PMC12605095; doi:10.3389/fonc.2025.1704299)
Supplement: Supplementary file 1 [file DataSheet1.docx]

**
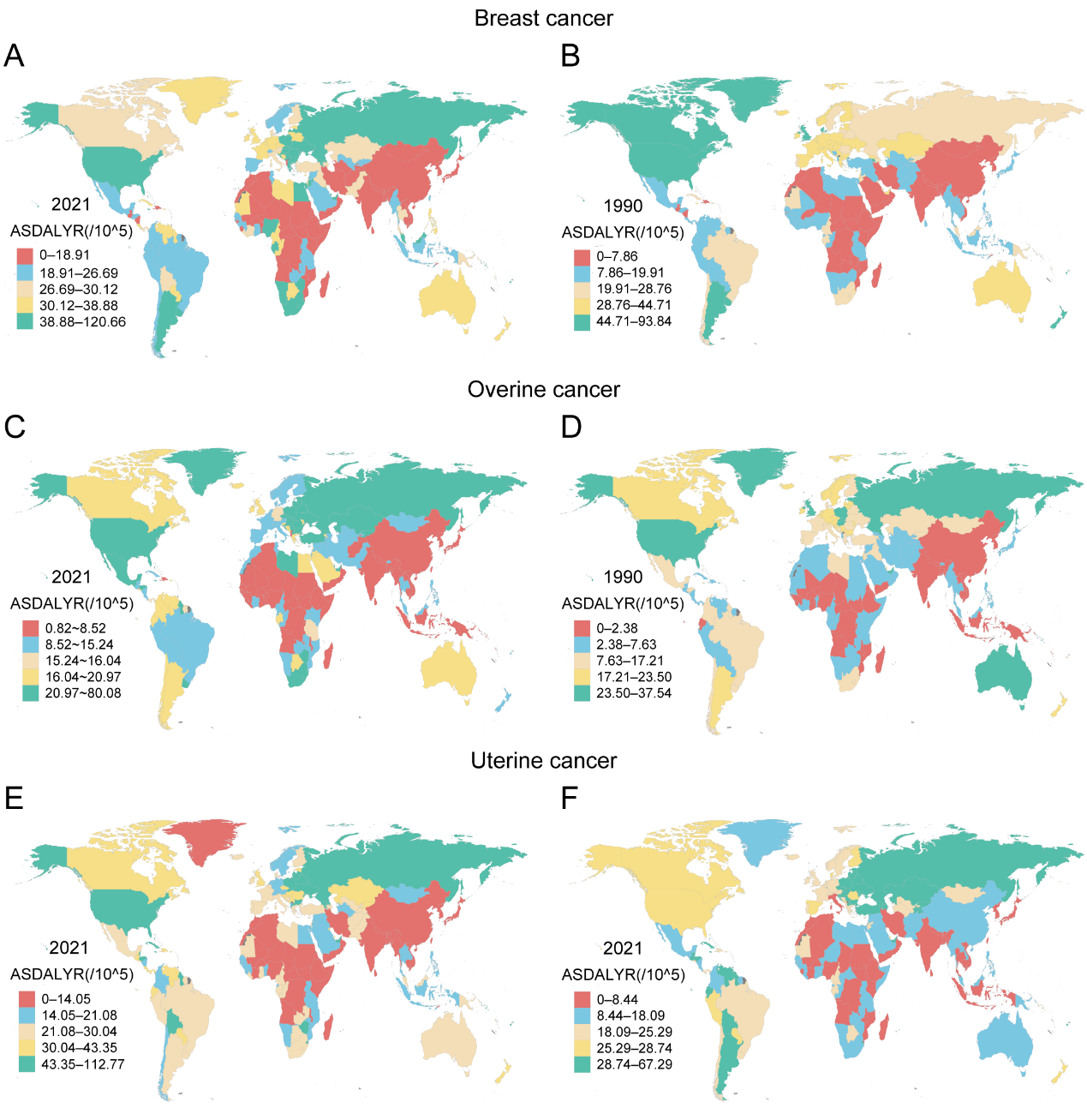
Figure S1.** Geographical distribution of ASDALYR attributable to high BMI for female breast, ovarian, and uterine cancers across 204 countries and territories in 1990 and 2021. (A) ASDALYR of breast cancer attributed to high BMI in 2021. (B) ASDALYR of breast cancer attributed to high BMI in 1990. (C) ASDALYR of ovarian cancer attributed to high BMI in 2021. (D) ASDALYR of ovarian cancer attributed to high BMI in 1990. (E) ASDALYR of uterine cancer attributed to high BMI in 2021. (F) ASDALYR of uterine cancer attributed to high BMI in 1990.
