# Supplementary material for: High BMI-attributable female-specific cancers: a comprehensive analysis of the global disease burden and trends from 1990 to 2021 and projections to 2040
Source: Front Oncol. 2025 Oct 29;15:1704299. doi: 10.3389/fonc.2025.1704299 (PMC12605095; doi:10.3389/fonc.2025.1704299)
Supplement: Supplementary file 2 [file Table1.docx]

| **Table S1**  Temporal trends in the burden of female breast, ovarian, and uterine cancers attributable to high BMI by Socio-demographic Index (SDI) quintiles, 1990-2021. | | | | | |
| --- | --- | --- | --- | --- | --- |
| **Breast cancer** | | | | | |
| **Location** | **year** | **Deaths Number**  **95%UI** | **ASDR**  **95%UI** | **DALYs Number**  **95%UI** | **ASDALYR**  **95%UI** |
| Global | 1990 | 18745.25 (-603.83, 38156.97) | 0.91 (-0.03, 1.87) | 429066.01 (-20023.71, 868900.80) | 20.55 (-0.86, 41.70) |
| Global | 1991 | 19297.17 (-618.16, 39020.81) | 0.92 (-0.03, 1.87) | 440150.38 (-20679.25, 891114.98) | 20.73 (-0.86, 42.06) |
| Global | 1992 | 19855.23 (-635.59, 40399.53) | 0.93 (-0.03, 1.90) | 450663.02 (-21454.53, 906593.87) | 20.92 (-0.86, 42.16) |
| Global | 1993 | 20548.31 (-661.92, 41585.89) | 0.95 (-0.03, 1.92) | 464657.01 (-22449.28, 936228.56) | 21.29 (-0.87, 43.01) |
| Global | 1994 | 21040.56 (-679.48, 42288.96) | 0.95 (-0.03, 1.92) | 474156.27 (-23462.73, 947465.27) | 21.47 (-0.87, 42.97) |
| Global | 1995 | 21375.54 (-688.84, 43124.32) | 0.95 (-0.03, 1.92) | 479415.97 (-24673.86, 959790.08) | 21.48 (-0.88, 43.20) |
| Global | 1996 | 21665.03 (-695.81, 43467.10) | 0.95 (-0.03, 1.91) | 483524.31 (-25117.17, 964139.47) | 21.38 (-0.87, 42.71) |
| Global | 1997 | 22055.05 (-708.58, 44271.85) | 0.94 (-0.03, 1.90) | 492844.72 (-25068.95, 983841.94) | 21.35 (-0.84, 42.72) |
| Global | 1998 | 22558.47 (-721.15, 45116.47) | 0.94 (-0.03, 1.90) | 504377.05 (-25346.98, 999523.49) | 21.40 (-0.83, 42.68) |
| Global | 1999 | 23321.40 (-745.31, 46743.83) | 0.95 (-0.03, 1.91) | 522916.58 (-25650.39, 1049613.69) | 21.63 (-0.82, 43.45) |
| Global | 2000 | 23943.47 (-769.55, 48320.04) | 0.95 (-0.03, 1.92) | 538259.59 (-25955.12, 1075515.92) | 21.78 (-0.81, 43.96) |
| Global | 2001 | 24563.19 (-788.80, 49163.44) | 0.95 (-0.03, 1.92) | 553934.66 (-26240.90, 1103185.56) | 21.80 (-0.81, 43.42) |
| Global | 2002 | 25259.30 (-812.48, 50646.34) | 0.95 (-0.03, 1.91) | 571197.28 (-26784.08, 1141132.53) | 21.85 (-0.81, 43.76) |
| Global | 2003 | 26010.98 (-836.52, 52224.32) | 0.96 (-0.03, 1.93) | 590828.23 (-27470.64, 1164149.69) | 21.95 (-0.82, 43.55) |
| Global | 2004 | 26558.50 (-853.69, 53461.96) | 0.95 (-0.03, 1.91) | 606885.91 (-27227.71, 1204959.55) | 21.86 (-0.80, 43.53) |
| Global | 2005 | 27265.24 (-873.47, 54614.68) | 0.95 (-0.03, 1.90) | 626407.39 (-27355.56, 1233315.93) | 21.86 (-0.79, 43.39) |
| Global | 2006 | 27887.56 (-890.84, 55696.42) | 0.94 (-0.03, 1.88) | 642899.87 (-27254.12, 1269554.54) | 21.73 (-0.78, 42.92) |
| Global | 2007 | 28645.81 (-907.50, 57230.63) | 0.94 (-0.03, 1.87) | 661804.77 (-26914.52, 1312926.72) | 21.70 (-0.77, 43.30) |
| Global | 2008 | 29617.95 (-941.35, 58880.87) | 0.94 (-0.03, 1.87) | 685256.16 (-27439.14, 1363825.05) | 21.83 (-0.78, 43.49) |
| Global | 2009 | 30460.33 (-971.36, 60628.03) | 0.94 (-0.03, 1.87) | 705690.24 (-28637.73, 1385060.29) | 21.85 (-0.78, 43.10) |
| Global | 2010 | 31430.99 (-998.67, 62419.95) | 0.94 (-0.03, 1.87) | 729761.09 (-28650.97, 1430067.97) | 21.93 (-0.78, 43.18) |
| Global | 2011 | 32349.22 (-1022.43, 64125.14) | 0.94 (-0.03, 1.86) | 751696.23 (-28542.40, 1471164.82) | 21.90 (-0.78, 43.03) |
| Global | 2012 | 33318.81 (-1050.34, 66592.29) | 0.94 (-0.03, 1.87) | 773844.10 (-28967.15, 1526185.39) | 21.83 (-0.77, 43.19) |
| Global | 2013 | 34296.63 (-1077.31, 68522.96) | 0.93 (-0.03, 1.86) | 796367.17 (-31093.03, 1565431.50) | 21.70 (-0.80, 42.73) |
| Global | 2014 | 35413.47 (-1131.85, 69942.80) | 0.93 (-0.03, 1.84) | 822689.00 (-31115.63, 1610432.56) | 21.64 (-0.80, 42.40) |
| Global | 2015 | 36837.51 (-1170.43, 73444.40) | 0.94 (-0.03, 1.87) | 855624.91 (-31740.62, 1671210.05) | 21.73 (-0.81, 42.44) |
| Global | 2016 | 38199.54 (-1225.93, 76292.33) | 0.94 (-0.03, 1.88) | 885980.31 (-34164.12, 1735378.75) | 21.74 (-0.87, 42.57) |
| Global | 2017 | 39452.17 (-1263.05, 78883.75) | 0.94 (-0.03, 1.88) | 914896.63 (-33748.94, 1808637.41) | 21.70 (-0.86, 42.82) |
| Global | 2018 | 40964.01 (-1307.12, 82923.77) | 0.95 (-0.03, 1.92) | 949908.34 (-34875.71, 1869364.26) | 21.80 (-0.89, 42.87) |
| Global | 2019 | 42288.10 (-1353.79, 84645.65) | 0.95 (-0.03, 1.90) | 981065.58 (-36372.41, 1922588.03) | 21.79 (-0.92, 42.57) |
| Global | 2020 | 43254.00 (-1419.44, 87341.87) | 0.94 (-0.03, 1.90) | 1003743.51 (-38007.33, 1993377.26) | 21.63 (-0.96, 42.84) |
| Global | 2021 | 44706.88 (-1478.43, 89575.02) | 0.95 (-0.03, 1.89) | 1041308.89 (-40215.85, 2029536.82) | 21.83 (-1.01, 42.51) |
| High SDI | 1990 | 9939.10 (-297.56, 20331.88) | 1.52 (-0.05, 3.11) | 220724.47 (-7455.66, 444093.49) | 35.25 (-1.46, 70.82) |
| High SDI | 1991 | 10155.42 (-303.23, 20706.49) | 1.53 (-0.05, 3.10) | 224370.00 (-7556.56, 448704.87) | 35.39 (-1.47, 70.63) |
| High SDI | 1992 | 10298.20 (-307.16, 20903.31) | 1.53 (-0.05, 3.09) | 226267.62 (-7616.88, 450772.75) | 35.36 (-1.45, 70.23) |
| High SDI | 1993 | 10516.83 (-316.04, 21195.58) | 1.54 (-0.05, 3.10) | 230515.42 (-7815.36, 458619.98) | 35.66 (-1.44, 70.70) |
| High SDI | 1994 | 10649.86 (-320.27, 21292.35) | 1.54 (-0.05, 3.06) | 233055.25 (-7943.17, 462378.21) | 35.67 (-1.44, 70.48) |
| High SDI | 1995 | 10761.50 (-323.51, 21484.41) | 1.53 (-0.05, 3.05) | 235411.85 (-8037.22, 466078.59) | 35.81 (-1.43, 70.58) |
| High SDI | 1996 | 10795.63 (-323.17, 21538.52) | 1.52 (-0.05, 3.01) | 235427.09 (-7999.18, 464035.43) | 35.50 (-1.39, 69.62) |
| High SDI | 1997 | 10848.99 (-324.86, 21663.78) | 1.49 (-0.05, 2.97) | 237599.67 (-8061.70, 468242.43) | 35.07 (-1.34, 68.79) |
| High SDI | 1998 | 10977.36 (-325.96, 21890.81) | 1.48 (-0.05, 2.93) | 240843.87 (-8067.03, 474380.00) | 34.70 (-1.30, 68.10) |
| High SDI | 1999 | 11191.84 (-332.62, 22252.22) | 1.48 (-0.05, 2.92) | 246170.40 (-8231.66, 485109.10) | 34.66 (-1.28, 68.13) |
| High SDI | 2000 | 11344.88 (-338.51, 22584.31) | 1.47 (-0.05, 2.90) | 250303.50 (-8389.97, 494370.93) | 34.54 (-1.28, 68.04) |
| High SDI | 2001 | 11499.74 (-342.77, 22882.49) | 1.46 (-0.04, 2.89) | 254624.17 (-8510.33, 500802.06) | 34.47 (-1.26, 67.66) |
| High SDI | 2002 | 11707.17 (-347.42, 23264.57) | 1.45 (-0.04, 2.88) | 259458.54 (-8607.31, 509760.62) | 34.35 (-1.24, 67.41) |
| High SDI | 2003 | 11850.63 (-352.23, 23600.92) | 1.44 (-0.04, 2.86) | 263776.32 (-8749.09, 517357.10) | 34.19 (-1.22, 66.88) |
| High SDI | 2004 | 11912.27 (-353.89, 23810.97) | 1.42 (-0.04, 2.82) | 266660.95 (-8820.94, 523797.86) | 33.84 (-1.19, 66.17) |
| High SDI | 2005 | 12039.26 (-357.40, 24107.96) | 1.40 (-0.04, 2.79) | 270635.88 (-8922.03, 532747.70) | 33.58 (-1.17, 65.88) |
| High SDI | 2006 | 12189.52 (-360.78, 24383.08) | 1.39 (-0.04, 2.76) | 274918.09 (-9029.34, 539993.65) | 33.37 (-1.17, 65.28) |
| High SDI | 2007 | 12341.60 (-362.43, 24682.15) | 1.38 (-0.04, 2.73) | 278874.49 (-9037.72, 548494.10) | 33.07 (-1.14, 64.83) |
| High SDI | 2008 | 12570.37 (-370.18, 25152.63) | 1.37 (-0.04, 2.72) | 284286.33 (-9248.97, 558629.12) | 32.91 (-1.15, 64.32) |
| High SDI | 2009 | 12736.67 (-375.42, 25447.90) | 1.35 (-0.04, 2.68) | 288485.64 (-9393.77, 563142.60) | 32.61 (-1.14, 63.40) |
| High SDI | 2010 | 12912.06 (-380.41, 25834.96) | 1.34 (-0.04, 2.66) | 292654.87 (-9494.97, 572970.17) | 32.32 (-1.13, 62.97) |
| High SDI | 2011 | 13151.45 (-385.45, 26259.52) | 1.33 (-0.04, 2.64) | 297915.46 (-9584.72, 584579.16) | 32.15 (-1.11, 62.37) |
| High SDI | 2012 | 13348.98 (-389.55, 26626.06) | 1.32 (-0.04, 2.61) | 301288.63 (-9589.52, 591368.65) | 31.73 (-1.09, 61.61) |
| High SDI | 2013 | 13573.77 (-397.42, 27057.40) | 1.31 (-0.04, 2.58) | 305621.68 (-9750.53, 600382.38) | 31.41 (-1.09, 61.03) |
| High SDI | 2014 | 13795.77 (-406.01, 27522.65) | 1.30 (-0.04, 2.56) | 309500.31 (-9942.45, 607855.19) | 31.03 (-1.09, 60.14) |
| High SDI | 2015 | 14151.25 (-416.26, 28287.45) | 1.30 (-0.04, 2.56) | 316045.74 (-10142.70, 620777.36) | 30.95 (-1.09, 59.96) |
| High SDI | 2016 | 14508.30 (-427.43, 29090.18) | 1.30 (-0.04, 2.57) | 322200.62 (-10388.67, 632925.32) | 30.88 (-1.10, 59.63) |
| High SDI | 2017 | 14777.68 (-434.36, 29575.10) | 1.29 (-0.04, 2.56) | 326146.05 (-10487.27, 642071.31) | 30.62 (-1.09, 59.70) |
| High SDI | 2018 | 15094.02 (-444.29, 30196.48) | 1.29 (-0.04, 2.55) | 331024.09 (-10703.96, 647923.74) | 30.44 (-1.09, 59.55) |
| High SDI | 2019 | 15250.27 (-452.97, 30312.68) | 1.28 (-0.04, 2.52) | 333372.20 (-10949.16, 655040.93) | 30.06 (-1.10, 58.80) |
| High SDI | 2020 | 15272.89 (-457.45, 30397.02) | 1.25 (-0.04, 2.48) | 332557.26 (-11035.09, 655485.91) | 29.42 (-1.09, 58.05) |
| High SDI | 2021 | 15594.60 (-469.52, 30954.65) | 1.26 (-0.04, 2.48) | 340511.24 (-11349.80, 665329.39) | 29.71 (-1.11, 58.00) |
| High-middle SDI | 1990 | 5868.84 (-196.11, 11946.59) | 1.03 (-0.03, 2.08) | 142870.11 (-5364.65, 285586.08) | 24.17 (-1.07, 48.35) |
| High-middle SDI | 1991 | 6070.39 (-200.51, 12308.19) | 1.04 (-0.03, 2.11) | 147460.85 (-5468.82, 295995.58) | 24.68 (-1.05, 49.59) |
| High-middle SDI | 1992 | 6311.24 (-208.17, 12820.82) | 1.07 (-0.04, 2.18) | 152451.70 (-5649.23, 308457.38) | 25.35 (-1.02, 51.36) |
| High-middle SDI | 1993 | 6624.25 (-219.08, 13372.90) | 1.11 (-0.04, 2.25) | 158816.20 (-5916.36, 318771.76) | 26.38 (-1.05, 52.84) |
| High-middle SDI | 1994 | 6786.83 (-225.14, 13631.26) | 1.13 (-0.04, 2.27) | 161509.49 (-6058.80, 320257.65) | 26.86 (-1.06, 53.27) |
| High-middle SDI | 1995 | 6854.45 (-225.72, 13779.33) | 1.13 (-0.04, 2.28) | 161313.65 (-6364.36, 321060.25) | 26.82 (-1.07, 53.41) |
| High-middle SDI | 1996 | 6945.57 (-227.67, 13935.64) | 1.13 (-0.04, 2.28) | 162092.27 (-6458.16, 321513.46) | 26.77 (-1.04, 53.10) |
| High-middle SDI | 1997 | 7107.27 (-231.28, 14201.75) | 1.14 (-0.04, 2.28) | 165508.49 (-6370.14, 327631.91) | 26.96 (-0.99, 53.36) |
| High-middle SDI | 1998 | 7270.19 (-234.14, 14568.76) | 1.15 (-0.04, 2.31) | 168988.87 (-6305.51, 334091.58) | 27.26 (-0.98, 53.93) |
| High-middle SDI | 1999 | 7603.00 (-244.89, 15313.91) | 1.18 (-0.04, 2.38) | 177144.84 (-6415.80, 351773.70) | 28.02 (-1.00, 55.69) |
| High-middle SDI | 2000 | 7858.61 (-253.90, 15832.21) | 1.20 (-0.04, 2.41) | 183563.71 (-6649.13, 366067.48) | 28.45 (-1.02, 56.73) |
| High-middle SDI | 2001 | 8066.07 (-258.84, 16227.01) | 1.20 (-0.04, 2.41) | 188772.61 (-6747.01, 372516.61) | 28.52 (-1.01, 56.26) |
| High-middle SDI | 2002 | 8262.87 (-265.81, 16602.40) | 1.20 (-0.04, 2.41) | 194328.53 (-6942.94, 384280.50) | 28.65 (-1.01, 56.71) |
| High-middle SDI | 2003 | 8550.69 (-277.38, 17130.21) | 1.22 (-0.04, 2.44) | 202046.13 (-7263.59, 396886.18) | 29.08 (-1.04, 57.10) |
| High-middle SDI | 2004 | 8704.66 (-281.34, 17599.82) | 1.21 (-0.04, 2.45) | 206693.23 (-7373.24, 409344.71) | 28.95 (-1.03, 57.38) |
| High-middle SDI | 2005 | 8928.19 (-288.12, 17935.03) | 1.21 (-0.04, 2.43) | 212834.11 (-7578.76, 421221.67) | 28.96 (-1.03, 57.30) |
| High-middle SDI | 2006 | 9015.22 (-291.09, 17955.92) | 1.19 (-0.04, 2.37) | 215180.65 (-7643.50, 421939.16) | 28.45 (-1.01, 55.74) |
| High-middle SDI | 2007 | 9219.42 (-293.46, 18300.21) | 1.18 (-0.04, 2.35) | 219830.73 (-7659.47, 437156.52) | 28.30 (-0.99, 56.22) |
| High-middle SDI | 2008 | 9518.54 (-304.07, 18762.99) | 1.19 (-0.04, 2.35) | 226875.89 (-7947.69, 447064.25) | 28.53 (-1.01, 56.28) |
| High-middle SDI | 2009 | 9736.01 (-309.00, 19213.98) | 1.19 (-0.04, 2.35) | 231980.04 (-8055.52, 455364.01) | 28.51 (-1.00, 56.02) |
| High-middle SDI | 2010 | 10021.37 (-316.41, 19694.16) | 1.20 (-0.04, 2.35) | 238932.68 (-8236.98, 465632.55) | 28.65 (-1.00, 55.90) |
| High-middle SDI | 2011 | 10184.98 (-316.14, 20006.54) | 1.18 (-0.04, 2.32) | 242582.81 (-8191.61, 473146.42) | 28.32 (-0.97, 55.32) |
| High-middle SDI | 2012 | 10424.85 (-322.43, 20595.91) | 1.18 (-0.04, 2.33) | 248263.16 (-8340.16, 490837.44) | 28.18 (-0.96, 55.69) |
| High-middle SDI | 2013 | 10640.37 (-325.65, 21319.51) | 1.17 (-0.04, 2.34) | 253370.49 (-8401.99, 503837.20) | 27.91 (-0.94, 55.50) |
| High-middle SDI | 2014 | 10891.16 (-338.39, 21392.28) | 1.16 (-0.04, 2.28) | 259185.52 (-8744.60, 508568.00) | 27.68 (-0.96, 54.53) |
| High-middle SDI | 2015 | 11198.71 (-342.62, 22269.55) | 1.16 (-0.04, 2.30) | 265823.61 (-8763.79, 525056.30) | 27.53 (-0.93, 54.18) |
| High-middle SDI | 2016 | 11460.48 (-357.26, 22638.89) | 1.15 (-0.04, 2.27) | 271880.68 (-9195.67, 526853.51) | 27.31 (-0.95, 53.14) |
| High-middle SDI | 2017 | 11695.50 (-361.59, 23341.13) | 1.14 (-0.04, 2.27) | 278414.00 (-9301.27, 549387.70) | 27.15 (-0.94, 53.36) |
| High-middle SDI | 2018 | 12032.20 (-366.58, 24092.38) | 1.14 (-0.04, 2.29) | 286926.23 (-9380.79, 563748.91) | 27.18 (-0.93, 53.47) |
| High-middle SDI | 2019 | 12331.33 (-379.31, 24636.98) | 1.14 (-0.04, 2.26) | 294196.81 (-9751.48, 579746.50) | 27.10 (-0.94, 53.44) |
| High-middle SDI | 2020 | 12549.38 (-386.52, 25089.92) | 1.13 (-0.04, 2.25) | 299464.15 (-9930.52, 588977.58) | 26.91 (-0.94, 52.46) |
| High-middle SDI | 2021 | 12962.00 (-403.89, 26097.05) | 1.14 (-0.04, 2.28) | 311069.79 (-10471.29, 606748.74) | 27.31 (-0.97, 52.79) |
| Low SDI | 1990 | 268.50 (-25.90, 548.10) | 0.29 (-0.01, 0.59) | 5066.18 (-1324.41, 11547.25) | 5.96 (-0.76, 12.51) |
| Low SDI | 1991 | 277.04 (-26.93, 561.83) | 0.29 (-0.01, 0.60) | 5184.67 (-1398.92, 11763.28) | 6.04 (-0.77, 12.17) |
| Low SDI | 1992 | 287.15 (-27.83, 587.39) | 0.29 (-0.01, 0.61) | 5359.77 (-1541.21, 12408.34) | 6.14 (-0.78, 12.78) |
| Low SDI | 1993 | 298.35 (-27.61, 618.16) | 0.30 (-0.01, 0.62) | 5539.07 (-1536.03, 13008.26) | 6.27 (-0.76, 12.88) |
| Low SDI | 1994 | 309.62 (-29.61, 631.87) | 0.31 (-0.01, 0.63) | 5730.08 (-1471.04, 13505.63) | 6.39 (-0.83, 13.33) |
| Low SDI | 1995 | 318.69 (-29.91, 667.11) | 0.31 (-0.01, 0.65) | 5835.51 (-1655.66, 13687.95) | 6.49 (-0.78, 13.63) |
| Low SDI | 1996 | 327.64 (-31.15, 677.59) | 0.32 (-0.01, 0.66) | 5933.99 (-1770.15, 13784.38) | 6.58 (-0.79, 13.75) |
| Low SDI | 1997 | 335.82 (-32.42, 691.64) | 0.32 (-0.01, 0.66) | 6015.31 (-1886.85, 13644.93) | 6.64 (-0.81, 13.75) |
| Low SDI | 1998 | 346.54 (-34.96, 721.27) | 0.32 (-0.01, 0.66) | 6143.20 (-1970.11, 14261.15) | 6.74 (-0.87, 14.23) |
| Low SDI | 1999 | 355.16 (-35.59, 736.64) | 0.33 (-0.01, 0.68) | 6181.99 (-2144.99, 14129.69) | 6.80 (-0.82, 14.12) |
| Low SDI | 2000 | 364.48 (-35.88, 760.56) | 0.33 (-0.01, 0.68) | 6213.81 (-2213.80, 14441.15) | 6.87 (-0.81, 14.57) |
| Low SDI | 2001 | 380.22 (-37.13, 785.32) | 0.33 (-0.02, 0.70) | 6496.24 (-2245.58, 14800.95) | 6.95 (-0.81, 14.55) |
| Low SDI | 2002 | 397.86 (-37.11, 819.11) | 0.34 (-0.02, 0.69) | 6846.00 (-2291.62, 15641.78) | 7.06 (-0.79, 14.79) |
| Low SDI | 2003 | 416.29 (-37.55, 853.31) | 0.35 (-0.02, 0.70) | 7182.44 (-2415.01, 16612.78) | 7.20 (-0.78, 14.96) |
| Low SDI | 2004 | 434.36 (-38.18, 881.99) | 0.35 (-0.02, 0.71) | 7470.03 (-2548.17, 17341.94) | 7.32 (-0.77, 15.00) |
| Low SDI | 2005 | 452.68 (-39.93, 916.34) | 0.36 (-0.02, 0.72) | 7790.05 (-2612.23, 17716.76) | 7.43 (-0.78, 15.24) |
| Low SDI | 2006 | 473.02 (-41.87, 966.13) | 0.36 (-0.02, 0.73) | 8112.14 (-2893.84, 18808.58) | 7.53 (-0.79, 15.47) |
| Low SDI | 2007 | 494.17 (-44.42, 980.04) | 0.37 (-0.02, 0.71) | 8473.52 (-2931.26, 19270.08) | 7.64 (-0.81, 15.22) |
| Low SDI | 2008 | 518.72 (-45.95, 1040.37) | 0.37 (-0.02, 0.73) | 8893.48 (-3077.41, 20567.60) | 7.78 (-0.82, 15.78) |
| Low SDI | 2009 | 549.25 (-46.95, 1091.89) | 0.38 (-0.02, 0.77) | 9457.16 (-3212.43, 21688.83) | 8.00 (-0.81, 16.05) |
| Low SDI | 2010 | 582.30 (-48.23, 1164.69) | 0.39 (-0.02, 0.77) | 10113.01 (-3272.98, 22965.34) | 8.22 (-0.80, 16.62) |
| Low SDI | 2011 | 616.93 (-51.94, 1237.22) | 0.41 (-0.02, 0.80) | 10721.90 (-3509.89, 24603.01) | 8.45 (-0.84, 17.02) |
| Low SDI | 2012 | 655.55 (-53.61, 1300.89) | 0.42 (-0.02, 0.81) | 11376.69 (-3676.22, 25947.77) | 8.71 (-0.84, 17.52) |
| Low SDI | 2013 | 701.03 (-60.42, 1396.15) | 0.43 (-0.02, 0.86) | 12157.59 (-3792.70, 27807.05) | 9.04 (-0.92, 18.31) |
| Low SDI | 2014 | 750.13 (-61.51, 1506.94) | 0.45 (-0.02, 0.90) | 13122.53 (-3877.53, 29931.07) | 9.40 (-0.90, 19.07) |
| Low SDI | 2015 | 795.64 (-65.76, 1590.07) | 0.46 (-0.02, 0.94) | 14000.60 (-4463.91, 31785.19) | 9.67 (-0.93, 19.40) |
| Low SDI | 2016 | 844.91 (-70.17, 1717.62) | 0.48 (-0.02, 0.97) | 14862.96 (-4771.42, 33529.30) | 9.95 (-0.96, 20.35) |
| Low SDI | 2017 | 899.56 (-72.56, 1833.29) | 0.49 (-0.02, 1.00) | 15848.19 (-4523.72, 36000.33) | 10.26 (-0.96, 21.10) |
| Low SDI | 2018 | 959.36 (-81.07, 1932.85) | 0.50 (-0.02, 1.03) | 17108.38 (-4700.39, 39738.00) | 10.58 (-1.04, 21.50) |
| Low SDI | 2019 | 1020.68 (-85.08, 2056.91) | 0.52 (-0.02, 1.06) | 18262.55 (-5201.50, 41535.29) | 10.87 (-1.05, 22.19) |
| Low SDI | 2020 | 1073.58 (-87.51, 2166.58) | 0.53 (-0.02, 1.07) | 19185.68 (-5819.27, 44100.93) | 11.07 (-1.05, 22.40) |
| Low SDI | 2021 | 1124.96 (-99.96, 2287.25) | 0.54 (-0.02, 1.11) | 20251.41 (-5950.37, 46456.23) | 11.29 (-1.16, 22.86) |
| Low-middle SDI | 1990 | 670.07 (-54.55, 1377.92) | 0.26 (-0.01, 0.54) | 12838.00 (-2886.59, 28840.70) | 5.34 (-0.63, 11.20) |
| Low-middle SDI | 1991 | 703.19 (-57.27, 1464.04) | 0.26 (-0.01, 0.56) | 13427.16 (-2998.21, 29314.71) | 5.46 (-0.64, 11.47) |
| Low-middle SDI | 1992 | 743.32 (-59.65, 1541.54) | 0.27 (-0.01, 0.57) | 14119.61 (-3336.68, 30830.49) | 5.62 (-0.65, 11.58) |
| Low-middle SDI | 1993 | 781.29 (-62.27, 1633.04) | 0.28 (-0.01, 0.59) | 14787.10 (-3478.60, 31692.17) | 5.77 (-0.66, 12.04) |
| Low-middle SDI | 1994 | 836.35 (-66.03, 1716.20) | 0.29 (-0.01, 0.60) | 15876.26 (-3647.50, 33957.10) | 6.03 (-0.69, 12.37) |
| Low-middle SDI | 1995 | 876.78 (-67.96, 1809.76) | 0.30 (-0.01, 0.62) | 16570.18 (-3898.60, 35650.55) | 6.19 (-0.69, 12.73) |
| Low-middle SDI | 1996 | 916.32 (-71.53, 1897.80) | 0.30 (-0.01, 0.63) | 17242.65 (-4215.90, 37402.68) | 6.34 (-0.70, 13.02) |
| Low-middle SDI | 1997 | 961.31 (-74.72, 1979.66) | 0.31 (-0.01, 0.65) | 18022.62 (-4635.58, 38493.73) | 6.51 (-0.72, 13.21) |
| Low-middle SDI | 1998 | 1019.29 (-79.23, 2063.92) | 0.32 (-0.01, 0.66) | 19124.58 (-4824.26, 41340.67) | 6.77 (-0.73, 13.62) |
| Low-middle SDI | 1999 | 1068.91 (-82.21, 2222.19) | 0.33 (-0.02, 0.69) | 19967.35 (-5161.07, 43097.91) | 6.94 (-0.73, 14.15) |
| Low-middle SDI | 2000 | 1131.38 (-86.18, 2270.17) | 0.34 (-0.02, 0.70) | 21209.53 (-5266.65, 46001.79) | 7.21 (-0.73, 14.37) |
| Low-middle SDI | 2001 | 1210.60 (-92.28, 2420.03) | 0.35 (-0.02, 0.72) | 22995.24 (-5448.84, 49093.74) | 7.49 (-0.73, 14.73) |
| Low-middle SDI | 2002 | 1316.78 (-98.99, 2642.51) | 0.37 (-0.02, 0.75) | 25296.18 (-5914.21, 53139.54) | 7.90 (-0.77, 15.45) |
| Low-middle SDI | 2003 | 1431.98 (-106.33, 2826.87) | 0.39 (-0.02, 0.79) | 27949.34 (-5906.46, 58568.35) | 8.34 (-0.77, 16.44) |
| Low-middle SDI | 2004 | 1538.66 (-111.44, 3071.36) | 0.40 (-0.02, 0.80) | 30435.81 (-5915.46, 62516.08) | 8.71 (-0.76, 17.09) |
| Low-middle SDI | 2005 | 1652.90 (-118.76, 3240.80) | 0.42 (-0.02, 0.84) | 33043.76 (-5657.52, 65988.23) | 9.07 (-0.74, 17.44) |
| Low-middle SDI | 2006 | 1772.84 (-126.30, 3434.63) | 0.43 (-0.02, 0.85) | 35860.39 (-6080.22, 72949.78) | 9.43 (-0.76, 18.34) |
| Low-middle SDI | 2007 | 1897.74 (-124.97, 3684.25) | 0.44 (-0.02, 0.89) | 38846.23 (-6147.26, 77979.88) | 9.78 (-0.77, 18.74) |
| Low-middle SDI | 2008 | 2030.04 (-127.70, 3960.52) | 0.46 (-0.02, 0.90) | 41809.77 (-6623.03, 85075.29) | 10.10 (-0.79, 19.59) |
| Low-middle SDI | 2009 | 2171.77 (-136.96, 4274.32) | 0.47 (-0.02, 0.92) | 45151.95 (-6631.91, 89919.53) | 10.43 (-0.83, 20.36) |
| Low-middle SDI | 2010 | 2317.21 (-138.17, 4540.90) | 0.49 (-0.02, 0.96) | 48693.07 (-7083.94, 96145.06) | 10.74 (-0.85, 20.82) |
| Low-middle SDI | 2011 | 2477.92 (-140.91, 4936.97) | 0.50 (-0.02, 0.98) | 52678.93 (-6837.70, 105104.69) | 11.09 (-0.89, 21.66) |
| Low-middle SDI | 2012 | 2636.45 (-153.33, 5217.64) | 0.51 (-0.02, 1.02) | 56237.42 (-6955.24, 110349.53) | 11.37 (-0.91, 22.03) |
| Low-middle SDI | 2013 | 2804.71 (-177.11, 5511.22) | 0.52 (-0.02, 1.02) | 59870.84 (-7814.92, 120903.48) | 11.64 (-0.95, 23.41) |
| Low-middle SDI | 2014 | 3012.50 (-171.72, 5796.96) | 0.54 (-0.02, 1.06) | 64921.19 (-7927.82, 129442.58) | 12.07 (-0.98, 23.53) |
| Low-middle SDI | 2015 | 3258.88 (-177.25, 6413.71) | 0.56 (-0.02, 1.13) | 70881.05 (-8434.70, 143533.17) | 12.63 (-1.03, 24.60) |
| Low-middle SDI | 2016 | 3463.62 (-191.62, 6857.97) | 0.58 (-0.02, 1.16) | 75324.88 (-9184.64, 151619.15) | 12.92 (-1.07, 25.42) |
| Low-middle SDI | 2017 | 3670.66 (-196.42, 7223.11) | 0.59 (-0.02, 1.18) | 79710.19 (-9519.67, 157066.77) | 13.17 (-1.08, 25.63) |
| Low-middle SDI | 2018 | 3916.97 (-203.04, 7690.38) | 0.61 (-0.02, 1.21) | 85462.24 (-10123.38, 171202.72) | 13.56 (-1.16, 26.61) |
| Low-middle SDI | 2019 | 4152.42 (-204.57, 8481.26) | 0.62 (-0.02, 1.27) | 90989.87 (-10759.55, 184517.73) | 13.87 (-1.17, 27.72) |
| Low-middle SDI | 2020 | 4336.08 (-230.53, 8660.86) | 0.63 (-0.02, 1.27) | 94998.73 (-10954.84, 193845.05) | 14.00 (-1.20, 27.74) |
| Low-middle SDI | 2021 | 4510.91 (-247.60, 9086.21) | 0.64 (-0.03, 1.30) | 99213.00 (-11906.34, 200580.92) | 14.15 (-1.24, 28.39) |
| Middle SDI | 1990 | 1966.77 (-68.98, 3902.13) | 0.40 (-0.01, 0.79) | 46837.32 (-3752.37, 92167.77) | 9.30 (-0.49, 18.44) |
| Middle SDI | 1991 | 2057.80 (-70.57, 4063.97) | 0.41 (-0.01, 0.81) | 48949.97 (-3839.22, 97270.59) | 9.50 (-0.50, 18.91) |
| Middle SDI | 1992 | 2180.52 (-75.22, 4428.04) | 0.42 (-0.01, 0.86) | 51677.57 (-4120.07, 101954.06) | 9.84 (-0.54, 19.66) |
| Middle SDI | 1993 | 2291.36 (-79.77, 4640.28) | 0.43 (-0.01, 0.88) | 54182.08 (-4333.03, 107847.31) | 10.14 (-0.54, 20.41) |
| Middle SDI | 1994 | 2420.24 (-84.10, 4812.40) | 0.45 (-0.01, 0.89) | 57138.21 (-4532.14, 113692.88) | 10.51 (-0.52, 20.97) |
| Middle SDI | 1995 | 2524.93 (-87.72, 5164.11) | 0.46 (-0.01, 0.93) | 59408.65 (-4671.14, 119273.57) | 10.74 (-0.54, 21.85) |
| Middle SDI | 1996 | 2639.34 (-91.71, 5250.71) | 0.47 (-0.02, 0.93) | 61927.11 (-4874.34, 123446.05) | 10.96 (-0.54, 21.89) |
| Middle SDI | 1997 | 2759.73 (-96.11, 5601.48) | 0.48 (-0.02, 0.97) | 64764.30 (-5092.46, 129583.68) | 11.18 (-0.54, 22.66) |
| Middle SDI | 1998 | 2903.46 (-101.57, 5868.72) | 0.49 (-0.02, 0.98) | 68346.07 (-5450.50, 136539.45) | 11.50 (-0.54, 23.16) |
| Middle SDI | 1999 | 3061.91 (-105.30, 6269.74) | 0.49 (-0.02, 1.01) | 72541.71 (-5575.24, 149337.75) | 11.76 (-0.53, 24.22) |
| Middle SDI | 2000 | 3203.50 (-112.04, 6529.87) | 0.50 (-0.02, 1.03) | 76055.88 (-5830.00, 153134.77) | 12.01 (-0.54, 24.42) |
| Middle SDI | 2001 | 3364.44 (-116.19, 6913.20) | 0.51 (-0.02, 1.04) | 80096.46 (-6125.38, 163124.20) | 12.19 (-0.53, 25.15) |
| Middle SDI | 2002 | 3531.28 (-123.35, 7178.12) | 0.52 (-0.02, 1.05) | 84287.00 (-6535.21, 170036.14) | 12.35 (-0.56, 25.22) |
| Middle SDI | 2003 | 3716.55 (-128.43, 7481.61) | 0.52 (-0.02, 1.07) | 88856.90 (-6869.92, 174060.87) | 12.50 (-0.58, 24.75) |
| Middle SDI | 2004 | 3922.66 (-135.34, 7925.78) | 0.53 (-0.02, 1.07) | 94583.46 (-6914.55, 193542.00) | 12.69 (-0.57, 25.74) |
| Middle SDI | 2005 | 4145.10 (-138.73, 8398.77) | 0.54 (-0.02, 1.09) | 101030.41 (-6761.71, 202655.50) | 12.92 (-0.55, 26.08) |
| Middle SDI | 2006 | 4389.06 (-147.51, 8807.44) | 0.55 (-0.02, 1.10) | 107733.58 (-6830.79, 217668.39) | 13.17 (-0.55, 26.58) |
| Middle SDI | 2007 | 4643.43 (-152.43, 9402.07) | 0.56 (-0.02, 1.13) | 114646.65 (-6868.82, 228355.14) | 13.45 (-0.54, 26.96) |
| Middle SDI | 2008 | 4929.53 (-164.16, 10027.77) | 0.57 (-0.02, 1.15) | 122229.20 (-7016.37, 245157.17) | 13.82 (-0.54, 28.04) |
| Middle SDI | 2009 | 5214.71 (-174.55, 10530.46) | 0.58 (-0.02, 1.18) | 129427.13 (-7596.94, 255996.38) | 14.15 (-0.57, 28.38) |
| Middle SDI | 2010 | 5545.51 (-182.92, 11150.68) | 0.60 (-0.02, 1.20) | 138168.33 (-7742.88, 272981.11) | 14.56 (-0.57, 29.07) |
| Middle SDI | 2011 | 5863.87 (-195.63, 11757.46) | 0.61 (-0.02, 1.23) | 146564.64 (-7645.80, 288584.59) | 14.86 (-0.55, 29.54) |
| Middle SDI | 2012 | 6196.98 (-204.38, 12614.08) | 0.62 (-0.02, 1.26) | 155404.75 (-7523.21, 307820.82) | 15.08 (-0.54, 30.28) |
| Middle SDI | 2013 | 6519.47 (-212.17, 13190.01) | 0.62 (-0.02, 1.26) | 164050.45 (-8261.29, 322424.60) | 15.18 (-0.59, 30.12) |
| Middle SDI | 2014 | 6905.18 (-232.89, 13809.85) | 0.63 (-0.02, 1.26) | 174637.87 (-8415.09, 340258.56) | 15.37 (-0.60, 30.08) |
| Middle SDI | 2015 | 7372.34 (-243.70, 14800.33) | 0.64 (-0.02, 1.29) | 187514.98 (-8595.88, 368215.76) | 15.70 (-0.62, 31.01) |
| Middle SDI | 2016 | 7860.53 (-265.00, 15744.65) | 0.65 (-0.02, 1.31) | 200336.35 (-9386.86, 392227.05) | 15.99 (-0.68, 31.40) |
| Middle SDI | 2017 | 8345.89 (-277.25, 16630.58) | 0.66 (-0.02, 1.33) | 213384.97 (-8959.91, 418795.87) | 16.23 (-0.66, 31.87) |
| Middle SDI | 2018 | 8896.95 (-294.14, 18325.40) | 0.68 (-0.02, 1.40) | 227961.46 (-9705.83, 456571.72) | 16.57 (-0.72, 33.24) |
| Middle SDI | 2019 | 9467.81 (-315.11, 19105.62) | 0.69 (-0.02, 1.40) | 242797.10 (-9813.08, 489932.71) | 16.91 (-0.73, 34.13) |
| Middle SDI | 2020 | 9955.91 (-342.20, 20074.26) | 0.70 (-0.02, 1.42) | 256081.45 (-10515.78, 514516.41) | 17.14 (-0.78, 34.47) |
| Middle SDI | 2021 | 10446.76 (-360.62, 21352.40) | 0.71 (-0.02, 1.45) | 268770.10 (-11318.84, 534437.65) | 17.35 (-0.84, 34.36) |
| **Ovarian cancer** | | | | | |
| **Location** | **year** | **Deaths Number**  **95%UI** | **ASDR**  **95%UI** | **DALYs Number**  **95%UI** | **ASDALYR**  **95%UI** |
| Global | 1990 | 6850.05 (1422.80, 12864.70) | 0.32 (0.07, 0.61) | 188874.09 (38400.57, 355691.42) | 8.72 (1.78, 16.41) |
| Global | 1991 | 7075.47 (1475.20, 13235.78) | 0.33 (0.07, 0.61) | 194708.56 (39804.31, 364556.17) | 8.81 (1.81, 16.47) |
| Global | 1992 | 7317.05 (1521.52, 13617.26) | 0.33 (0.07, 0.62) | 201053.65 (40908.61, 374019.84) | 8.91 (1.82, 16.56) |
| Global | 1993 | 7583.17 (1582.11, 14163.99) | 0.34 (0.07, 0.63) | 208284.84 (42755.31, 389221.07) | 9.05 (1.86, 16.89) |
| Global | 1994 | 7825.31 (1633.17, 14560.50) | 0.34 (0.07, 0.63) | 214873.79 (44225.66, 400213.33) | 9.14 (1.89, 17.01) |
| Global | 1995 | 8111.01 (1700.69, 15054.79) | 0.34 (0.07, 0.64) | 222715.41 (46222.38, 414794.16) | 9.29 (1.93, 17.28) |
| Global | 1996 | 8283.23 (1738.12, 15383.88) | 0.34 (0.07, 0.64) | 226978.70 (47291.05, 422074.70) | 9.27 (1.93, 17.22) |
| Global | 1997 | 8503.80 (1786.29, 15710.84) | 0.35 (0.07, 0.64) | 232677.39 (48316.91, 431617.27) | 9.29 (1.93, 17.22) |
| Global | 1998 | 8702.77 (1831.97, 16067.02) | 0.35 (0.07, 0.64) | 237650.05 (49573.74, 438466.25) | 9.29 (1.94, 17.11) |
| Global | 1999 | 8945.56 (1882.53, 16509.98) | 0.35 (0.07, 0.64) | 244186.23 (50818.53, 451005.31) | 9.33 (1.95, 17.20) |
| Global | 2000 | 9368.57 (1995.71, 17283.39) | 0.36 (0.08, 0.66) | 255687.15 (53697.42, 470164.66) | 9.55 (2.01, 17.55) |
| Global | 2001 | 9693.76 (2080.87, 17815.02) | 0.36 (0.08, 0.66) | 264687.74 (55974.54, 485779.65) | 9.65 (2.05, 17.69) |
| Global | 2002 | 10031.77 (2175.35, 18352.26) | 0.36 (0.08, 0.66) | 274270.85 (58782.29, 501431.20) | 9.77 (2.10, 17.84) |
| Global | 2003 | 10347.21 (2271.65, 18949.06) | 0.36 (0.08, 0.67) | 282925.77 (61248.71, 518582.48) | 9.84 (2.14, 17.99) |
| Global | 2004 | 10623.20 (2337.56, 19433.08) | 0.37 (0.08, 0.67) | 290265.20 (63273.67, 531694.78) | 9.85 (2.15, 18.00) |
| Global | 2005 | 10924.25 (2408.18, 20031.16) | 0.37 (0.08, 0.67) | 299471.92 (65700.24, 552640.02) | 9.91 (2.18, 18.25) |
| Global | 2006 | 11216.18 (2471.69, 20513.91) | 0.37 (0.08, 0.67) | 306967.37 (67741.81, 563394.82) | 9.90 (2.20, 18.14) |
| Global | 2007 | 11518.97 (2560.07, 21023.24) | 0.37 (0.08, 0.67) | 314468.41 (69929.22, 573924.38) | 9.88 (2.21, 18.02) |
| Global | 2008 | 11880.45 (2662.09, 21663.97) | 0.37 (0.08, 0.67) | 324693.29 (72899.76, 593789.90) | 9.94 (2.24, 18.16) |
| Global | 2009 | 12252.21 (2752.73, 22416.75) | 0.37 (0.08, 0.67) | 335249.01 (75707.09, 611801.79) | 9.99 (2.26, 18.22) |
| Global | 2010 | 12447.58 (2803.50, 22628.03) | 0.36 (0.08, 0.66) | 341812.48 (77583.20, 618552.37) | 9.92 (2.25, 17.94) |
| Global | 2011 | 12812.38 (2912.09, 23088.92) | 0.36 (0.08, 0.66) | 352038.39 (80164.91, 637996.94) | 9.95 (2.27, 18.02) |
| Global | 2012 | 13152.41 (3004.27, 23777.96) | 0.36 (0.08, 0.66) | 361429.59 (82449.62, 651843.74) | 9.95 (2.27, 17.93) |
| Global | 2013 | 13551.25 (3121.35, 24243.11) | 0.36 (0.08, 0.65) | 372430.15 (86017.52, 664515.52) | 9.98 (2.31, 17.81) |
| Global | 2014 | 13983.99 (3243.75, 25180.54) | 0.37 (0.08, 0.66) | 384454.23 (89344.28, 689097.85) | 10.04 (2.33, 17.99) |
| Global | 2015 | 14272.94 (3323.88, 25556.87) | 0.36 (0.08, 0.65) | 392898.01 (90769.24, 699725.54) | 10.00 (2.31, 17.81) |
| Global | 2016 | 14809.36 (3479.24, 26466.71) | 0.37 (0.09, 0.66) | 407693.70 (95441.66, 721706.27) | 10.12 (2.37, 17.92) |
| Global | 2017 | 15285.77 (3601.02, 27410.86) | 0.37 (0.09, 0.66) | 420984.47 (98832.41, 752622.05) | 10.20 (2.39, 18.24) |
| Global | 2018 | 15769.79 (3740.63, 28235.45) | 0.37 (0.09, 0.66) | 434455.39 (102526.37, 778561.60) | 10.27 (2.42, 18.41) |
| Global | 2019 | 16258.34 (3847.73, 29126.89) | 0.37 (0.09, 0.67) | 447564.20 (105852.98, 799132.52) | 10.34 (2.44, 18.47) |
| Global | 2020 | 16682.41 (3979.64, 29978.17) | 0.37 (0.09, 0.67) | 458690.61 (109300.20, 816259.62) | 10.36 (2.46, 18.44) |
| Global | 2021 | 17344.47 (4141.33, 30810.14) | 0.38 (0.09, 0.67) | 477248.38 (113449.26, 840002.14) | 10.56 (2.50, 18.57) |
| High SDI | 1990 | 3801.70 (811.75, 7120.06) | 0.61 (0.13, 1.15) | 96741.78 (20587.01, 180827.63) | 16.78 (3.57, 31.36) |
| High SDI | 1991 | 3895.94 (833.78, 7289.72) | 0.62 (0.13, 1.16) | 98763.14 (21019.40, 183554.86) | 16.90 (3.59, 31.41) |
| High SDI | 1992 | 3974.90 (853.88, 7424.62) | 0.62 (0.13, 1.16) | 100356.78 (21381.82, 185992.06) | 16.92 (3.60, 31.34) |
| High SDI | 1993 | 4063.43 (875.67, 7597.90) | 0.63 (0.13, 1.17) | 102277.54 (21844.38, 189715.75) | 16.98 (3.62, 31.47) |
| High SDI | 1994 | 4163.14 (898.98, 7776.95) | 0.63 (0.14, 1.18) | 104525.05 (22472.37, 193350.61) | 17.10 (3.69, 31.57) |
| High SDI | 1995 | 4271.33 (924.19, 7983.02) | 0.64 (0.14, 1.19) | 107211.94 (23253.36, 198212.39) | 17.30 (3.77, 31.95) |
| High SDI | 1996 | 4341.83 (940.44, 8119.99) | 0.64 (0.14, 1.19) | 108510.82 (23796.62, 200180.06) | 17.25 (3.80, 31.77) |
| High SDI | 1997 | 4439.33 (971.43, 8273.49) | 0.64 (0.14, 1.19) | 110607.51 (24585.00, 203189.76) | 17.29 (3.84, 31.71) |
| High SDI | 1998 | 4516.76 (998.83, 8415.58) | 0.64 (0.14, 1.19) | 112278.09 (25228.27, 206281.74) | 17.26 (3.88, 31.60) |
| High SDI | 1999 | 4625.69 (1039.95, 8577.81) | 0.64 (0.15, 1.19) | 114851.28 (26191.30, 210278.47) | 17.37 (3.96, 31.69) |
| High SDI | 2000 | 4768.87 (1080.08, 8860.92) | 0.65 (0.15, 1.21) | 118034.70 (27192.28, 216429.50) | 17.56 (4.05, 32.08) |
| High SDI | 2001 | 4897.77 (1116.63, 9061.72) | 0.66 (0.15, 1.22) | 121185.61 (28158.64, 221260.00) | 17.76 (4.13, 32.30) |
| High SDI | 2002 | 5004.33 (1148.45, 9246.92) | 0.66 (0.15, 1.22) | 123815.69 (28903.49, 225707.08) | 17.84 (4.17, 32.40) |
| High SDI | 2003 | 5100.02 (1184.66, 9370.92) | 0.66 (0.16, 1.21) | 125988.10 (29726.99, 228309.46) | 17.83 (4.21, 32.19) |
| High SDI | 2004 | 5183.16 (1213.62, 9486.04) | 0.66 (0.16, 1.21) | 127695.45 (30344.89, 231005.90) | 17.74 (4.23, 31.99) |
| High SDI | 2005 | 5191.53 (1221.70, 9505.19) | 0.65 (0.15, 1.18) | 127800.48 (30405.54, 230366.06) | 17.45 (4.16, 31.31) |
| High SDI | 2006 | 5284.05 (1245.53, 9661.87) | 0.65 (0.15, 1.18) | 129654.02 (30982.02, 232994.11) | 17.40 (4.17, 31.16) |
| High SDI | 2007 | 5328.93 (1263.39, 9745.56) | 0.64 (0.15, 1.16) | 130162.90 (31234.26, 233327.09) | 17.13 (4.12, 30.67) |
| High SDI | 2008 | 5396.71 (1286.44, 9865.75) | 0.63 (0.15, 1.16) | 131531.21 (31814.14, 236756.49) | 17.00 (4.12, 30.57) |
| High SDI | 2009 | 5503.51 (1318.67, 10019.38) | 0.63 (0.15, 1.15) | 134045.95 (32579.77, 241250.99) | 17.02 (4.15, 30.62) |
| High SDI | 2010 | 5405.28 (1293.10, 9875.94) | 0.61 (0.15, 1.11) | 131399.99 (31892.23, 235446.83) | 16.39 (3.99, 29.33) |
| High SDI | 2011 | 5529.41 (1330.42, 10022.84) | 0.61 (0.15, 1.11) | 133997.96 (32772.90, 239363.76) | 16.43 (4.03, 29.36) |
| High SDI | 2012 | 5583.52 (1347.87, 10071.87) | 0.61 (0.15, 1.09) | 134732.78 (32999.81, 241154.73) | 16.22 (3.98, 29.04) |
| High SDI | 2013 | 5655.24 (1371.51, 10160.03) | 0.60 (0.15, 1.08) | 135863.51 (33430.23, 243641.11) | 16.07 (3.96, 28.81) |
| High SDI | 2014 | 5744.18 (1402.36, 10300.27) | 0.60 (0.15, 1.07) | 137512.32 (34069.43, 245200.45) | 15.99 (3.97, 28.59) |
| High SDI | 2015 | 5687.17 (1388.54, 10157.53) | 0.58 (0.14, 1.04) | 135832.02 (33552.43, 242121.62) | 15.53 (3.84, 27.71) |
| High SDI | 2016 | 5825.29 (1429.17, 10409.59) | 0.59 (0.14, 1.05) | 138878.53 (34464.27, 246796.40) | 15.65 (3.89, 27.81) |
| High SDI | 2017 | 5918.67 (1457.67, 10511.95) | 0.58 (0.14, 1.03) | 140491.75 (35001.15, 249147.73) | 15.58 (3.89, 27.67) |
| High SDI | 2018 | 5983.24 (1476.61, 10692.74) | 0.58 (0.14, 1.03) | 141672.99 (35311.53, 251671.60) | 15.49 (3.87, 27.43) |
| High SDI | 2019 | 6016.52 (1490.53, 10737.45) | 0.57 (0.14, 1.02) | 141614.51 (35397.79, 250525.55) | 15.26 (3.82, 26.87) |
| High SDI | 2020 | 6036.87 (1500.10, 10693.72) | 0.56 (0.14, 1.00) | 141240.27 (35443.58, 249210.98) | 14.99 (3.77, 26.49) |
| High SDI | 2021 | 6186.80 (1531.13, 10979.34) | 0.57 (0.14, 1.01) | 144449.46 (36080.39, 255582.85) | 15.13 (3.79, 26.82) |
| High-middle SDI | 1990 | 2243.75 (483.65, 4206.94) | 0.40 (0.09, 0.75) | 65388.64 (13955.94, 121986.45) | 11.71 (2.49, 21.84) |
| High-middle SDI | 1991 | 2321.83 (497.99, 4307.60) | 0.40 (0.09, 0.75) | 67438.24 (14436.73, 124568.29) | 11.86 (2.53, 21.94) |
| High-middle SDI | 1992 | 2418.38 (525.75, 4436.13) | 0.41 (0.09, 0.76) | 69983.20 (15069.82, 127797.22) | 12.10 (2.60, 22.12) |
| High-middle SDI | 1993 | 2534.68 (547.93, 4682.84) | 0.43 (0.09, 0.79) | 73236.04 (15787.16, 135252.06) | 12.47 (2.68, 23.07) |
| High-middle SDI | 1994 | 2608.15 (569.70, 4817.44) | 0.43 (0.09, 0.80) | 75345.51 (16292.53, 138787.87) | 12.63 (2.73, 23.29) |
| High-middle SDI | 1995 | 2707.82 (591.00, 4987.31) | 0.44 (0.10, 0.81) | 78034.77 (16922.44, 143933.31) | 12.87 (2.79, 23.78) |
| High-middle SDI | 1996 | 2727.52 (588.54, 5013.47) | 0.44 (0.09, 0.80) | 78302.14 (16816.89, 143240.28) | 12.67 (2.72, 23.20) |
| High-middle SDI | 1997 | 2764.66 (596.67, 5074.15) | 0.43 (0.09, 0.80) | 79053.48 (16952.18, 144784.25) | 12.56 (2.69, 23.00) |
| High-middle SDI | 1998 | 2799.15 (603.81, 5083.55) | 0.43 (0.09, 0.78) | 79472.02 (17020.47, 143670.26) | 12.40 (2.65, 22.44) |
| High-middle SDI | 1999 | 2844.07 (621.11, 5208.62) | 0.43 (0.09, 0.79) | 80522.08 (17457.52, 146880.53) | 12.34 (2.67, 22.49) |
| High-middle SDI | 2000 | 3023.41 (670.82, 5533.41) | 0.45 (0.10, 0.82) | 85531.43 (18893.63, 156124.16) | 12.86 (2.83, 23.48) |
| High-middle SDI | 2001 | 3116.36 (695.06, 5686.37) | 0.45 (0.10, 0.82) | 87890.01 (19607.28, 159724.97) | 12.94 (2.88, 23.50) |
| High-middle SDI | 2002 | 3223.78 (724.74, 5921.92) | 0.46 (0.10, 0.84) | 90825.80 (20304.06, 166266.70) | 13.11 (2.93, 23.99) |
| High-middle SDI | 2003 | 3314.71 (746.95, 6038.99) | 0.46 (0.10, 0.84) | 93141.13 (20979.50, 169201.30) | 13.20 (2.97, 23.97) |
| High-middle SDI | 2004 | 3374.82 (765.50, 6150.17) | 0.46 (0.10, 0.84) | 94518.72 (21314.19, 171494.74) | 13.14 (2.96, 23.85) |
| High-middle SDI | 2005 | 3519.49 (804.33, 6409.46) | 0.47 (0.11, 0.86) | 98819.65 (22454.94, 180873.88) | 13.46 (3.06, 24.63) |
| High-middle SDI | 2006 | 3568.33 (814.74, 6476.89) | 0.47 (0.11, 0.85) | 99613.09 (22595.00, 181207.14) | 13.28 (3.01, 24.14) |
| High-middle SDI | 2007 | 3672.07 (848.29, 6610.55) | 0.47 (0.11, 0.85) | 101700.69 (23345.28, 182679.63) | 13.27 (3.04, 23.83) |
| High-middle SDI | 2008 | 3784.06 (882.49, 6797.85) | 0.48 (0.11, 0.85) | 104640.28 (24280.27, 187812.76) | 13.37 (3.10, 23.99) |
| High-middle SDI | 2009 | 3866.00 (905.17, 6976.04) | 0.48 (0.11, 0.86) | 106697.40 (24813.62, 191559.62) | 13.34 (3.10, 23.94) |
| High-middle SDI | 2010 | 3981.05 (939.59, 7134.49) | 0.48 (0.11, 0.86) | 110059.26 (25797.87, 197065.63) | 13.46 (3.15, 24.11) |
| High-middle SDI | 2011 | 4023.55 (957.64, 7166.53) | 0.47 (0.11, 0.84) | 111333.45 (26274.99, 197710.32) | 13.32 (3.14, 23.65) |
| High-middle SDI | 2012 | 4110.06 (978.48, 7355.13) | 0.47 (0.11, 0.84) | 113582.96 (26801.13, 203454.96) | 13.29 (3.13, 23.83) |
| High-middle SDI | 2013 | 4210.22 (1011.24, 7469.63) | 0.47 (0.11, 0.84) | 116152.12 (27699.02, 207092.47) | 13.30 (3.16, 23.76) |
| High-middle SDI | 2014 | 4307.65 (1039.96, 7644.27) | 0.47 (0.11, 0.84) | 118572.71 (28418.04, 209778.81) | 13.29 (3.18, 23.54) |
| High-middle SDI | 2015 | 4386.63 (1062.24, 7774.24) | 0.47 (0.11, 0.83) | 120257.08 (28900.07, 213760.70) | 13.19 (3.16, 23.53) |
| High-middle SDI | 2016 | 4488.09 (1083.36, 8001.77) | 0.47 (0.11, 0.84) | 122689.70 (29380.12, 217904.98) | 13.19 (3.15, 23.42) |
| High-middle SDI | 2017 | 4584.32 (1114.16, 8188.01) | 0.47 (0.11, 0.84) | 125459.85 (30335.68, 226472.59) | 13.22 (3.19, 23.92) |
| High-middle SDI | 2018 | 4698.37 (1160.96, 8372.05) | 0.47 (0.12, 0.84) | 128338.77 (31608.44, 231385.81) | 13.26 (3.26, 23.98) |
| High-middle SDI | 2019 | 4822.49 (1179.47, 8614.99) | 0.47 (0.11, 0.84) | 131410.20 (31876.01, 237273.52) | 13.33 (3.22, 24.18) |
| High-middle SDI | 2020 | 4911.44 (1209.53, 8733.55) | 0.47 (0.12, 0.84) | 133135.99 (32718.29, 238975.08) | 13.26 (3.24, 23.92) |
| High-middle SDI | 2021 | 5094.95 (1240.98, 9059.80) | 0.48 (0.12, 0.85) | 138126.25 (33461.41, 244870.67) | 13.54 (3.26, 24.13) |
| Low SDI | 1990 | 62.43 (0.00, 138.85) | 0.05 (-0.00, 0.11) | 2127.12 (24.10, 4780.70) | 1.60 (0.01, 3.56) |
| Low SDI | 1991 | 65.52 (0.23, 142.02) | 0.05 (-0.00, 0.11) | 2233.26 (43.02, 4908.75) | 1.64 (0.03, 3.57) |
| Low SDI | 1992 | 68.69 (0.35, 148.16) | 0.05 (-0.00, 0.11) | 2342.10 (41.03, 5102.62) | 1.69 (0.02, 3.65) |
| Low SDI | 1993 | 72.39 (1.13, 157.55) | 0.05 (-0.00, 0.12) | 2469.16 (56.42, 5512.03) | 1.74 (0.04, 3.83) |
| Low SDI | 1994 | 75.93 (1.66, 165.41) | 0.06 (0.00, 0.12) | 2589.87 (77.31, 5809.71) | 1.79 (0.05, 3.95) |
| Low SDI | 1995 | 79.31 (2.01, 174.65) | 0.06 (0.00, 0.13) | 2705.45 (99.21, 6005.31) | 1.84 (0.06, 4.04) |
| Low SDI | 1996 | 83.28 (2.54, 177.22) | 0.06 (0.00, 0.13) | 2841.15 (131.18, 6185.58) | 1.89 (0.08, 4.06) |
| Low SDI | 1997 | 87.46 (3.09, 192.35) | 0.06 (0.00, 0.13) | 2985.20 (151.46, 6562.64) | 1.94 (0.09, 4.24) |
| Low SDI | 1998 | 92.25 (3.90, 202.66) | 0.06 (0.00, 0.14) | 3148.80 (174.60, 6931.15) | 2.00 (0.10, 4.40) |
| Low SDI | 1999 | 97.31 (4.64, 210.40) | 0.06 (0.00, 0.14) | 3322.10 (209.70, 7195.49) | 2.06 (0.13, 4.44) |
| Low SDI | 2000 | 102.81 (5.55, 220.26) | 0.07 (0.00, 0.14) | 3508.81 (240.51, 7534.75) | 2.13 (0.15, 4.54) |
| Low SDI | 2001 | 108.84 (6.33, 233.44) | 0.07 (0.00, 0.15) | 3716.70 (286.26, 7926.15) | 2.19 (0.16, 4.68) |
| Low SDI | 2002 | 115.18 (8.32, 242.77) | 0.07 (0.00, 0.15) | 3928.33 (321.85, 8278.77) | 2.26 (0.18, 4.76) |
| Low SDI | 2003 | 122.99 (9.70, 256.29) | 0.07 (0.00, 0.16) | 4195.88 (366.77, 8744.85) | 2.35 (0.20, 4.89) |
| Low SDI | 2004 | 131.45 (11.22, 268.23) | 0.08 (0.01, 0.16) | 4487.53 (385.46, 9157.10) | 2.44 (0.21, 4.97) |
| Low SDI | 2005 | 139.66 (12.03, 287.12) | 0.08 (0.01, 0.16) | 4767.16 (436.87, 9817.79) | 2.52 (0.23, 5.18) |
| Low SDI | 2006 | 148.57 (13.13, 303.27) | 0.08 (0.01, 0.17) | 5069.72 (477.10, 10408.46) | 2.60 (0.25, 5.32) |
| Low SDI | 2007 | 158.26 (15.03, 320.44) | 0.09 (0.01, 0.17) | 5402.54 (559.19, 10971.99) | 2.69 (0.28, 5.43) |
| Low SDI | 2008 | 168.89 (18.09, 340.91) | 0.09 (0.01, 0.18) | 5765.84 (671.22, 11582.93) | 2.79 (0.32, 5.58) |
| Low SDI | 2009 | 180.90 (21.55, 360.44) | 0.09 (0.01, 0.18) | 6179.19 (786.47, 12294.58) | 2.89 (0.37, 5.74) |
| Low SDI | 2010 | 192.97 (24.85, 387.57) | 0.09 (0.01, 0.19) | 6589.03 (908.73, 13090.94) | 2.99 (0.41, 5.97) |
| Low SDI | 2011 | 206.72 (27.34, 412.97) | 0.10 (0.01, 0.20) | 7063.94 (989.85, 14215.50) | 3.10 (0.43, 6.20) |
| Low SDI | 2012 | 222.26 (29.91, 443.31) | 0.10 (0.01, 0.20) | 7601.83 (1053.20, 15087.69) | 3.23 (0.45, 6.43) |
| Low SDI | 2013 | 239.97 (33.59, 463.05) | 0.11 (0.01, 0.21) | 8222.06 (1183.17, 15723.30) | 3.38 (0.49, 6.48) |
| Low SDI | 2014 | 259.42 (36.57, 506.02) | 0.11 (0.02, 0.22) | 8900.59 (1290.41, 17433.02) | 3.53 (0.51, 6.88) |
| Low SDI | 2015 | 278.91 (39.65, 538.96) | 0.12 (0.02, 0.23) | 9574.68 (1368.71, 18446.24) | 3.68 (0.53, 7.09) |
| Low SDI | 2016 | 300.72 (44.15, 582.30) | 0.12 (0.02, 0.23) | 10335.39 (1522.30, 19877.65) | 3.84 (0.57, 7.37) |
| Low SDI | 2017 | 324.36 (47.31, 631.14) | 0.13 (0.02, 0.25) | 11153.62 (1628.30, 21683.66) | 4.00 (0.59, 7.77) |
| Low SDI | 2018 | 348.57 (51.89, 679.96) | 0.13 (0.02, 0.26) | 11990.14 (1790.53, 23377.19) | 4.16 (0.62, 8.09) |
| Low SDI | 2019 | 375.67 (55.54, 728.55) | 0.14 (0.02, 0.27) | 12931.47 (1914.86, 24963.95) | 4.33 (0.64, 8.37) |
| Low SDI | 2020 | 405.04 (61.70, 787.14) | 0.14 (0.02, 0.28) | 13968.35 (2135.79, 27012.47) | 4.52 (0.69, 8.76) |
| Low SDI | 2021 | 432.55 (67.53, 839.64) | 0.15 (0.02, 0.29) | 14942.61 (2348.90, 28748.14) | 4.68 (0.74, 9.04) |
| Low-middle SDI | 1990 | 187.03 (13.39, 391.74) | 0.06 (0.00, 0.12) | 6283.22 (459.39, 13116.94) | 1.78 (0.13, 3.74) |
| Low-middle SDI | 1991 | 199.70 (14.87, 418.18) | 0.06 (0.00, 0.13) | 6719.21 (510.89, 14051.68) | 1.86 (0.14, 3.87) |
| Low-middle SDI | 1992 | 214.39 (16.83, 443.41) | 0.06 (0.00, 0.13) | 7215.83 (571.11, 14949.09) | 1.94 (0.16, 4.02) |
| Low-middle SDI | 1993 | 227.79 (19.11, 469.00) | 0.06 (0.01, 0.13) | 7684.07 (647.40, 16020.28) | 2.02 (0.17, 4.16) |
| Low-middle SDI | 1994 | 243.90 (21.81, 495.00) | 0.07 (0.01, 0.14) | 8213.25 (742.82, 16984.38) | 2.11 (0.19, 4.31) |
| Low-middle SDI | 1995 | 266.97 (25.30, 541.57) | 0.07 (0.01, 0.15) | 8953.74 (898.11, 18244.02) | 2.25 (0.22, 4.56) |
| Low-middle SDI | 1996 | 294.22 (31.73, 593.61) | 0.08 (0.01, 0.16) | 9877.66 (1115.48, 19910.60) | 2.42 (0.27, 4.85) |
| Low-middle SDI | 1997 | 319.79 (37.17, 637.97) | 0.08 (0.01, 0.16) | 10760.80 (1300.14, 21444.75) | 2.56 (0.31, 5.11) |
| Low-middle SDI | 1998 | 343.93 (42.03, 689.46) | 0.09 (0.01, 0.17) | 11568.33 (1472.86, 23070.69) | 2.69 (0.34, 5.38) |
| Low-middle SDI | 1999 | 370.45 (47.69, 746.04) | 0.09 (0.01, 0.18) | 12445.72 (1657.52, 24895.07) | 2.82 (0.38, 5.65) |
| Low-middle SDI | 2000 | 402.34 (54.92, 786.18) | 0.10 (0.01, 0.19) | 13520.06 (1901.72, 26394.76) | 2.98 (0.42, 5.82) |
| Low-middle SDI | 2001 | 437.62 (61.28, 862.13) | 0.10 (0.01, 0.20) | 14700.01 (2126.50, 28924.21) | 3.16 (0.46, 6.21) |
| Low-middle SDI | 2002 | 483.39 (74.21, 936.83) | 0.11 (0.02, 0.21) | 16188.59 (2558.95, 31439.99) | 3.38 (0.54, 6.58) |
| Low-middle SDI | 2003 | 527.54 (85.76, 1021.43) | 0.11 (0.02, 0.22) | 17649.36 (2958.98, 34295.47) | 3.59 (0.60, 6.94) |
| Low-middle SDI | 2004 | 574.48 (95.24, 1096.50) | 0.12 (0.02, 0.23) | 19184.81 (3249.37, 36643.03) | 3.79 (0.64, 7.23) |
| Low-middle SDI | 2005 | 624.40 (103.76, 1187.07) | 0.13 (0.02, 0.24) | 20810.74 (3451.78, 39556.52) | 3.99 (0.66, 7.58) |
| Low-middle SDI | 2006 | 673.18 (113.63, 1259.83) | 0.13 (0.02, 0.25) | 22408.08 (3766.74, 41923.48) | 4.17 (0.71, 7.80) |
| Low-middle SDI | 2007 | 723.85 (122.69, 1348.86) | 0.14 (0.02, 0.26) | 24044.20 (4101.33, 44884.97) | 4.35 (0.74, 8.13) |
| Low-middle SDI | 2008 | 782.34 (136.52, 1474.02) | 0.15 (0.03, 0.28) | 25978.23 (4618.08, 48884.71) | 4.56 (0.81, 8.57) |
| Low-middle SDI | 2009 | 836.50 (150.03, 1573.58) | 0.15 (0.03, 0.29) | 27759.04 (5127.96, 51753.11) | 4.73 (0.87, 8.83) |
| Low-middle SDI | 2010 | 890.92 (164.12, 1675.02) | 0.16 (0.03, 0.29) | 29550.71 (5534.46, 55367.53) | 4.88 (0.91, 9.14) |
| Low-middle SDI | 2011 | 952.29 (176.34, 1790.41) | 0.16 (0.03, 0.31) | 31573.25 (5888.24, 59237.89) | 5.06 (0.94, 9.51) |
| Low-middle SDI | 2012 | 1017.78 (194.94, 1898.83) | 0.17 (0.03, 0.31) | 33706.03 (6510.13, 63131.07) | 5.24 (1.01, 9.82) |
| Low-middle SDI | 2013 | 1084.76 (210.62, 1993.63) | 0.17 (0.03, 0.32) | 35921.64 (7022.15, 66028.13) | 5.41 (1.06, 9.93) |
| Low-middle SDI | 2014 | 1158.32 (227.32, 2132.25) | 0.18 (0.04, 0.33) | 38321.21 (7563.30, 70307.90) | 5.60 (1.11, 10.29) |
| Low-middle SDI | 2015 | 1242.38 (242.40, 2319.21) | 0.19 (0.04, 0.35) | 41054.14 (8109.07, 76310.27) | 5.83 (1.15, 10.82) |
| Low-middle SDI | 2016 | 1331.34 (270.48, 2460.89) | 0.19 (0.04, 0.36) | 43876.25 (8993.39, 81368.79) | 6.05 (1.24, 11.21) |
| Low-middle SDI | 2017 | 1417.24 (283.73, 2607.60) | 0.20 (0.04, 0.37) | 46597.75 (9385.00, 85585.43) | 6.24 (1.26, 11.48) |
| Low-middle SDI | 2018 | 1508.65 (294.67, 2784.88) | 0.21 (0.04, 0.38) | 49472.66 (9697.53, 91205.22) | 6.44 (1.26, 11.87) |
| Low-middle SDI | 2019 | 1593.91 (314.04, 2959.85) | 0.21 (0.04, 0.40) | 52114.25 (10302.42, 95725.50) | 6.60 (1.31, 12.16) |
| Low-middle SDI | 2020 | 1684.18 (333.39, 3131.98) | 0.22 (0.04, 0.41) | 55060.82 (11166.48, 101226.68) | 6.79 (1.37, 12.52) |
| Low-middle SDI | 2021 | 1771.25 (357.16, 3219.98) | 0.22 (0.04, 0.41) | 57905.91 (11972.87, 104670.77) | 6.96 (1.43, 12.58) |
| Middle SDI | 1990 | 542.54 (65.34, 1064.41) | 0.10 (0.01, 0.19) | 17976.12 (2083.04, 35888.37) | 2.99 (0.36, 5.90) |
| Middle SDI | 1991 | 579.37 (75.90, 1121.11) | 0.10 (0.01, 0.19) | 19183.64 (2442.11, 37558.73) | 3.10 (0.41, 6.02) |
| Middle SDI | 1992 | 626.96 (84.97, 1234.62) | 0.11 (0.01, 0.21) | 20766.56 (2743.68, 41039.94) | 3.26 (0.44, 6.43) |
| Middle SDI | 1993 | 670.46 (96.87, 1327.30) | 0.11 (0.02, 0.22) | 22210.23 (3153.30, 44333.07) | 3.40 (0.49, 6.75) |
| Middle SDI | 1994 | 719.18 (104.60, 1404.04) | 0.11 (0.02, 0.22) | 23776.98 (3400.38, 46613.56) | 3.54 (0.52, 6.92) |
| Middle SDI | 1995 | 769.67 (117.30, 1513.11) | 0.12 (0.02, 0.24) | 25360.69 (3816.57, 50158.66) | 3.69 (0.56, 7.28) |
| Middle SDI | 1996 | 819.84 (130.72, 1590.14) | 0.12 (0.02, 0.24) | 26982.68 (4283.88, 52579.46) | 3.82 (0.61, 7.42) |
| Middle SDI | 1997 | 875.30 (140.35, 1688.56) | 0.13 (0.02, 0.25) | 28786.12 (4596.80, 55866.53) | 3.97 (0.64, 7.68) |
| Middle SDI | 1998 | 933.46 (153.22, 1801.23) | 0.13 (0.02, 0.26) | 30700.04 (5029.93, 59200.36) | 4.11 (0.68, 7.92) |
| Middle SDI | 1999 | 991.33 (164.99, 1909.03) | 0.14 (0.02, 0.27) | 32577.58 (5454.61, 62834.76) | 4.24 (0.71, 8.17) |
| Middle SDI | 2000 | 1054.05 (176.28, 2020.24) | 0.14 (0.02, 0.27) | 34616.82 (5827.67, 66423.16) | 4.38 (0.74, 8.41) |
| Middle SDI | 2001 | 1115.35 (188.69, 2135.06) | 0.15 (0.02, 0.28) | 36700.84 (6257.67, 70173.58) | 4.50 (0.77, 8.63) |
| Middle SDI | 2002 | 1186.82 (209.82, 2265.21) | 0.15 (0.03, 0.29) | 39007.34 (6961.85, 74133.43) | 4.64 (0.83, 8.82) |
| Middle SDI | 2003 | 1262.90 (230.24, 2415.54) | 0.16 (0.03, 0.30) | 41427.10 (7563.13, 79444.47) | 4.78 (0.88, 9.14) |
| Middle SDI | 2004 | 1339.77 (247.53, 2537.87) | 0.16 (0.03, 0.30) | 43844.10 (8149.54, 83211.53) | 4.91 (0.91, 9.29) |
| Middle SDI | 2005 | 1429.19 (268.27, 2705.87) | 0.17 (0.03, 0.31) | 46727.40 (8800.51, 88339.69) | 5.07 (0.96, 9.56) |
| Middle SDI | 2006 | 1521.69 (295.01, 2843.46) | 0.17 (0.03, 0.32) | 49666.56 (9677.21, 93274.97) | 5.22 (1.02, 9.77) |
| Middle SDI | 2007 | 1615.05 (316.34, 3016.28) | 0.17 (0.03, 0.33) | 52590.70 (10252.32, 98033.93) | 5.36 (1.05, 9.98) |
| Middle SDI | 2008 | 1727.25 (344.41, 3221.99) | 0.18 (0.04, 0.34) | 56202.36 (11147.43, 104650.48) | 5.54 (1.10, 10.31) |
| Middle SDI | 2009 | 1843.70 (365.34, 3386.23) | 0.19 (0.04, 0.34) | 59981.62 (11825.51, 110524.36) | 5.72 (1.13, 10.52) |
| Middle SDI | 2010 | 1955.61 (391.41, 3597.13) | 0.19 (0.04, 0.35) | 63624.48 (12701.56, 117091.71) | 5.88 (1.17, 10.81) |
| Middle SDI | 2011 | 2077.90 (426.23, 3850.69) | 0.20 (0.04, 0.36) | 67462.95 (13897.50, 124912.68) | 6.04 (1.24, 11.20) |
| Middle SDI | 2012 | 2195.90 (455.60, 4052.81) | 0.20 (0.04, 0.37) | 71191.00 (14797.34, 131230.15) | 6.18 (1.28, 11.40) |
| Middle SDI | 2013 | 2337.51 (497.35, 4256.43) | 0.21 (0.04, 0.38) | 75638.81 (16123.62, 137161.99) | 6.37 (1.36, 11.56) |
| Middle SDI | 2014 | 2490.37 (543.96, 4500.34) | 0.21 (0.05, 0.38) | 80504.49 (17619.10, 145226.09) | 6.58 (1.44, 11.87) |
| Middle SDI | 2015 | 2653.55 (578.07, 4735.86) | 0.22 (0.05, 0.39) | 85532.18 (18626.04, 152533.18) | 6.79 (1.48, 12.13) |
| Middle SDI | 2016 | 2839.48 (634.06, 5057.28) | 0.23 (0.05, 0.41) | 91265.54 (20399.01, 162453.50) | 7.05 (1.57, 12.56) |
| Middle SDI | 2017 | 3016.30 (688.18, 5364.89) | 0.23 (0.05, 0.42) | 96624.38 (22051.64, 171170.97) | 7.26 (1.65, 12.88) |
| Middle SDI | 2018 | 3205.55 (724.65, 5787.77) | 0.24 (0.05, 0.43) | 102312.86 (23411.77, 185350.92) | 7.49 (1.71, 13.58) |
| Middle SDI | 2019 | 3424.17 (772.08, 6096.63) | 0.25 (0.06, 0.44) | 108822.46 (24855.10, 193384.09) | 7.76 (1.77, 13.79) |
| Middle SDI | 2020 | 3619.27 (842.82, 6533.81) | 0.26 (0.06, 0.46) | 114618.78 (26840.46, 204931.89) | 7.98 (1.86, 14.26) |
| Middle SDI | 2021 | 3832.58 (903.66, 6971.62) | 0.26 (0.06, 0.48) | 121138.56 (28726.80, 221614.16) | 8.25 (1.95, 15.09) |
| **Uterine cancer** | | | | | |
| **Location** | **year** | **Deaths Number**  **95%UI** | **ASDR**  **95%UI** | **DALYs Number**  **95%UI** | **ASDALYR**  **95%UI** |
| Global | 1990 | 13893.30 (9874.05, 18652.61) | 0.66 (0.47, 0.89) | 372641.14 (264224.34, 500196.91) | 17.26 (12.25, 23.16) |
| Global | 1991 | 14228.89 (10074.32, 18972.89) | 0.66 (0.47, 0.88) | 381286.39 (269058.51, 508249.99) | 17.29 (12.22, 23.06) |
| Global | 1992 | 14586.11 (10323.11, 19416.19) | 0.66 (0.47, 0.88) | 390545.33 (273830.50, 521042.05) | 17.35 (12.18, 23.15) |
| Global | 1993 | 15001.58 (10634.78, 19992.58) | 0.67 (0.47, 0.89) | 402381.00 (284862.22, 536539.52) | 17.51 (12.40, 23.33) |
| Global | 1994 | 15386.92 (10882.80, 20491.19) | 0.67 (0.47, 0.89) | 413045.64 (290768.85, 550178.96) | 17.61 (12.41, 23.44) |
| Global | 1995 | 15574.84 (11076.18, 20578.03) | 0.66 (0.47, 0.88) | 417554.71 (295405.52, 553161.08) | 17.46 (12.35, 23.11) |
| Global | 1996 | 15886.45 (11307.13, 21070.61) | 0.66 (0.47, 0.88) | 425466.46 (301654.48, 563084.97) | 17.42 (12.35, 23.04) |
| Global | 1997 | 16233.96 (11541.78, 21455.30) | 0.66 (0.47, 0.88) | 433955.72 (307386.66, 572247.60) | 17.38 (12.32, 22.92) |
| Global | 1998 | 16578.13 (11819.44, 21836.66) | 0.66 (0.47, 0.87) | 442614.70 (312962.54, 585913.40) | 17.35 (12.27, 22.95) |
| Global | 1999 | 17030.67 (12112.24, 22562.88) | 0.66 (0.47, 0.88) | 455449.17 (321637.59, 601198.68) | 17.46 (12.33, 23.05) |
| Global | 2000 | 17349.63 (12265.80, 23018.18) | 0.66 (0.47, 0.88) | 463924.97 (325475.48, 613001.51) | 17.40 (12.23, 23.00) |
| Global | 2001 | 17742.08 (12650.06, 23390.15) | 0.66 (0.47, 0.87) | 474911.25 (336452.22, 625348.76) | 17.41 (12.33, 22.91) |
| Global | 2002 | 18220.38 (13004.94, 24159.66) | 0.66 (0.47, 0.88) | 487900.00 (345211.40, 642476.82) | 17.47 (12.37, 22.99) |
| Global | 2003 | 18837.98 (13534.29, 24713.96) | 0.67 (0.48, 0.88) | 505230.01 (361319.66, 661276.06) | 17.67 (12.64, 23.13) |
| Global | 2004 | 19179.05 (13695.98, 25245.94) | 0.66 (0.47, 0.87) | 515931.90 (364234.44, 675042.82) | 17.62 (12.46, 23.09) |
| Global | 2005 | 19475.44 (13846.45, 25579.07) | 0.66 (0.47, 0.86) | 525519.18 (372204.69, 688482.23) | 17.52 (12.42, 22.96) |
| Global | 2006 | 19636.32 (13955.56, 25900.17) | 0.65 (0.46, 0.85) | 529665.60 (374142.43, 694197.90) | 17.21 (12.17, 22.59) |
| Global | 2007 | 19880.18 (14168.89, 26006.60) | 0.64 (0.45, 0.83) | 535654.32 (382111.60, 702057.37) | 16.96 (12.10, 22.24) |
| Global | 2008 | 20535.19 (14546.94, 27018.26) | 0.64 (0.45, 0.84) | 553172.50 (391620.81, 728428.02) | 17.06 (12.09, 22.47) |
| Global | 2009 | 21151.29 (14895.78, 27709.72) | 0.64 (0.45, 0.84) | 570044.45 (399917.40, 748424.17) | 17.11 (12.01, 22.47) |
| Global | 2010 | 21984.67 (15539.01, 28839.57) | 0.65 (0.46, 0.85) | 592347.20 (416206.14, 777200.58) | 17.32 (12.17, 22.73) |
| Global | 2011 | 22831.20 (16194.33, 29908.24) | 0.65 (0.46, 0.86) | 615277.18 (434742.43, 806729.56) | 17.50 (12.37, 22.95) |
| Global | 2012 | 23696.73 (16857.51, 31269.39) | 0.66 (0.47, 0.87) | 637843.15 (452558.06, 837989.23) | 17.65 (12.52, 23.18) |
| Global | 2013 | 24736.34 (17670.67, 32364.50) | 0.67 (0.48, 0.87) | 665209.62 (474269.79, 872481.80) | 17.91 (12.77, 23.49) |
| Global | 2014 | 25417.75 (18103.47, 33119.32) | 0.67 (0.48, 0.87) | 681819.17 (483523.84, 885899.24) | 17.86 (12.67, 23.20) |
| Global | 2015 | 26718.65 (19080.58, 34935.02) | 0.68 (0.49, 0.89) | 714387.68 (507516.27, 932584.78) | 18.21 (12.94, 23.77) |
| Global | 2016 | 27954.60 (20105.28, 36552.46) | 0.69 (0.50, 0.91) | 746152.55 (534670.37, 973042.21) | 18.51 (13.26, 24.14) |
| Global | 2017 | 29151.93 (20851.48, 38413.22) | 0.70 (0.50, 0.92) | 777107.97 (554407.09, 1020338.04) | 18.77 (13.39, 24.64) |
| Global | 2018 | 30356.50 (21838.77, 39877.45) | 0.71 (0.51, 0.93) | 807797.98 (580346.84, 1063308.09) | 19.00 (13.65, 25.02) |
| Global | 2019 | 31410.24 (22570.30, 41177.61) | 0.71 (0.51, 0.94) | 834976.01 (599286.23, 1099380.98) | 19.14 (13.74, 25.20) |
| Global | 2020 | 32018.93 (22757.27, 42335.25) | 0.71 (0.50, 0.94) | 849603.80 (603600.20, 1116225.52) | 19.00 (13.50, 24.97) |
| Global | 2021 | 33134.48 (23878.10, 43299.38) | 0.72 (0.52, 0.94) | 880146.92 (631164.68, 1160930.41) | 19.23 (13.80, 25.38) |
| High SDI | 1990 | 5501.35 (3911.77, 7459.67) | 0.84 (0.60, 1.14) | 131275.84 (94742.22, 176287.47) | 21.45 (15.47, 28.69) |
| High SDI | 1991 | 5550.42 (3944.45, 7524.08) | 0.83 (0.59, 1.13) | 132222.42 (95190.98, 176801.29) | 21.31 (15.36, 28.40) |
| High SDI | 1992 | 5616.06 (3997.13, 7609.70) | 0.83 (0.59, 1.12) | 133238.65 (95649.47, 178405.39) | 21.15 (15.19, 28.22) |
| High SDI | 1993 | 5646.72 (4011.81, 7636.51) | 0.82 (0.58, 1.11) | 134022.90 (96259.23, 178816.54) | 20.99 (15.09, 27.90) |
| High SDI | 1994 | 5711.22 (4049.19, 7699.97) | 0.82 (0.58, 1.10) | 135545.80 (96952.32, 180574.02) | 20.95 (14.99, 27.79) |
| High SDI | 1995 | 5783.85 (4098.58, 7803.37) | 0.81 (0.58, 1.09) | 137326.27 (97925.45, 182625.19) | 20.94 (14.94, 27.79) |
| High SDI | 1996 | 5871.96 (4158.00, 7903.62) | 0.81 (0.58, 1.09) | 139448.48 (99636.30, 185013.44) | 20.98 (15.00, 27.75) |
| High SDI | 1997 | 5941.67 (4205.05, 7986.54) | 0.81 (0.57, 1.08) | 141214.22 (100677.59, 186867.45) | 20.93 (14.96, 27.63) |
| High SDI | 1998 | 6062.75 (4292.42, 8110.67) | 0.81 (0.57, 1.08) | 144066.95 (102755.99, 190752.55) | 21.02 (15.03, 27.78) |
| High SDI | 1999 | 6182.36 (4369.92, 8266.21) | 0.81 (0.58, 1.08) | 147425.76 (105048.15, 194665.85) | 21.22 (15.17, 27.99) |
| High SDI | 2000 | 6322.51 (4475.23, 8442.81) | 0.82 (0.58, 1.09) | 151405.77 (108101.23, 200041.51) | 21.50 (15.39, 28.33) |
| High SDI | 2001 | 6495.09 (4602.43, 8658.84) | 0.83 (0.59, 1.10) | 155813.36 (111403.46, 205441.44) | 21.81 (15.63, 28.71) |
| High SDI | 2002 | 6631.88 (4708.81, 8829.04) | 0.83 (0.59, 1.10) | 159119.18 (114056.41, 208899.69) | 21.90 (15.72, 28.66) |
| High SDI | 2003 | 6736.95 (4791.48, 8945.51) | 0.83 (0.59, 1.10) | 162145.02 (116476.69, 212194.05) | 21.95 (15.80, 28.64) |
| High SDI | 2004 | 6837.68 (4874.37, 9058.50) | 0.83 (0.59, 1.09) | 164937.21 (118508.64, 215735.08) | 21.95 (15.79, 28.64) |
| High SDI | 2005 | 6963.81 (4964.68, 9202.81) | 0.83 (0.59, 1.09) | 169237.41 (121676.85, 221041.78) | 22.20 (15.98, 28.92) |
| High SDI | 2006 | 7120.28 (5096.99, 9408.68) | 0.83 (0.60, 1.10) | 173515.24 (125099.87, 226831.76) | 22.40 (16.13, 29.21) |
| High SDI | 2007 | 7289.15 (5222.98, 9628.24) | 0.84 (0.60, 1.10) | 177995.52 (128780.78, 232665.18) | 22.55 (16.30, 29.32) |
| High SDI | 2008 | 7508.94 (5368.34, 9925.50) | 0.84 (0.61, 1.11) | 183650.20 (133170.47, 240114.42) | 22.82 (16.49, 29.68) |
| High SDI | 2009 | 7721.49 (5511.10, 10178.02) | 0.85 (0.61, 1.12) | 189367.54 (136690.73, 247070.83) | 23.09 (16.61, 29.95) |
| High SDI | 2010 | 8071.67 (5758.67, 10647.53) | 0.87 (0.63, 1.15) | 198039.89 (143154.91, 258030.31) | 23.69 (17.07, 30.75) |
| High SDI | 2011 | 8361.90 (5963.45, 11036.98) | 0.89 (0.64, 1.16) | 205460.59 (148312.11, 268434.18) | 24.15 (17.37, 31.44) |
| High SDI | 2012 | 8664.24 (6160.39, 11421.83) | 0.90 (0.65, 1.18) | 212384.94 (153694.70, 276822.03) | 24.47 (17.66, 31.77) |
| High SDI | 2013 | 8991.65 (6410.55, 11831.22) | 0.91 (0.66, 1.20) | 220417.51 (158999.06, 288207.26) | 24.93 (18.02, 32.48) |
| High SDI | 2014 | 9364.58 (6678.34, 12319.47) | 0.93 (0.67, 1.22) | 229174.39 (165590.72, 298429.05) | 25.41 (18.41, 32.96) |
| High SDI | 2015 | 9866.61 (7018.55, 12985.38) | 0.96 (0.69, 1.26) | 240673.94 (174033.85, 313704.34) | 26.16 (18.97, 33.98) |
| High SDI | 2016 | 10382.69 (7402.55, 13644.22) | 0.99 (0.71, 1.29) | 253389.38 (183181.84, 330718.79) | 27.11 (19.76, 35.29) |
| High SDI | 2017 | 10833.27 (7728.74, 14250.20) | 1.01 (0.73, 1.32) | 263645.03 (190958.37, 343197.14) | 27.71 (20.28, 35.97) |
| High SDI | 2018 | 11210.98 (8030.52, 14786.28) | 1.02 (0.74, 1.34) | 271395.42 (196816.64, 354363.68) | 28.01 (20.51, 36.37) |
| High SDI | 2019 | 11504.62 (8225.87, 15152.26) | 1.03 (0.74, 1.35) | 277694.18 (201805.46, 360833.45) | 28.18 (20.71, 36.48) |
| High SDI | 2020 | 11544.53 (8176.26, 15214.14) | 1.01 (0.73, 1.32) | 277372.49 (200342.13, 360437.80) | 27.66 (20.21, 35.72) |
| High SDI | 2021 | 11838.05 (8412.47, 15582.73) | 1.02 (0.74, 1.34) | 284155.61 (206126.84, 368034.30) | 27.91 (20.50, 36.15) |
| High-middle SDI | 1990 | 5428.22 (3843.73, 7215.31) | 0.96 (0.68, 1.28) | 151011.50 (106435.22, 201012.90) | 26.67 (18.82, 35.51) |
| High-middle SDI | 1991 | 5596.13 (3953.87, 7413.21) | 0.97 (0.68, 1.28) | 155166.86 (109623.58, 204993.14) | 26.88 (18.96, 35.49) |
| High-middle SDI | 1992 | 5754.19 (4033.32, 7611.33) | 0.98 (0.68, 1.29) | 159474.61 (111482.19, 210190.40) | 27.12 (18.94, 35.77) |
| High-middle SDI | 1993 | 6040.22 (4266.30, 7996.04) | 1.01 (0.71, 1.33) | 167695.97 (118534.00, 222505.41) | 28.06 (19.83, 37.25) |
| High-middle SDI | 1994 | 6209.53 (4404.71, 8216.21) | 1.02 (0.72, 1.34) | 172414.42 (122686.83, 227289.08) | 28.40 (20.23, 37.46) |
| High-middle SDI | 1995 | 6224.65 (4392.71, 8212.26) | 1.00 (0.71, 1.32) | 172197.91 (121344.73, 226464.32) | 27.91 (19.64, 36.70) |
| High-middle SDI | 1996 | 6293.79 (4450.13, 8328.15) | 0.99 (0.70, 1.31) | 173345.48 (122678.68, 228280.55) | 27.58 (19.52, 36.34) |
| High-middle SDI | 1997 | 6369.62 (4519.11, 8374.45) | 0.99 (0.70, 1.30) | 174216.88 (123971.37, 229143.68) | 27.20 (19.33, 35.84) |
| High-middle SDI | 1998 | 6428.15 (4531.32, 8478.29) | 0.98 (0.69, 1.29) | 175059.82 (123118.36, 230828.64) | 26.88 (18.90, 35.44) |
| High-middle SDI | 1999 | 6597.18 (4651.90, 8670.34) | 0.98 (0.69, 1.30) | 179681.89 (126713.17, 236101.53) | 27.12 (19.13, 35.60) |
| High-middle SDI | 2000 | 6609.41 (4686.38, 8700.30) | 0.97 (0.69, 1.27) | 179291.15 (127435.98, 235711.89) | 26.57 (18.87, 34.95) |
| High-middle SDI | 2001 | 6645.42 (4765.79, 8713.45) | 0.95 (0.68, 1.25) | 180415.26 (129487.03, 236908.38) | 26.23 (18.81, 34.43) |
| High-middle SDI | 2002 | 6789.65 (4827.63, 8892.33) | 0.96 (0.68, 1.25) | 184135.90 (130573.30, 241646.81) | 26.27 (18.62, 34.48) |
| High-middle SDI | 2003 | 7055.02 (5053.71, 9157.29) | 0.98 (0.70, 1.27) | 191103.91 (136450.32, 247858.47) | 26.84 (19.18, 34.83) |
| High-middle SDI | 2004 | 7089.48 (5041.45, 9283.99) | 0.97 (0.69, 1.27) | 192767.08 (136657.06, 251376.89) | 26.61 (18.83, 34.69) |
| High-middle SDI | 2005 | 7062.57 (5037.07, 9213.38) | 0.94 (0.67, 1.23) | 192198.03 (137767.40, 250967.45) | 26.02 (18.64, 33.98) |
| High-middle SDI | 2006 | 6889.89 (4886.79, 9048.14) | 0.90 (0.64, 1.18) | 186828.58 (132768.69, 244147.68) | 24.77 (17.60, 32.39) |
| High-middle SDI | 2007 | 6744.51 (4831.48, 8836.01) | 0.86 (0.62, 1.13) | 181821.08 (130959.13, 237903.18) | 23.59 (17.01, 30.88) |
| High-middle SDI | 2008 | 6888.25 (4915.10, 9004.02) | 0.86 (0.61, 1.13) | 185142.87 (133044.74, 240999.91) | 23.54 (16.94, 30.66) |
| High-middle SDI | 2009 | 6997.03 (4998.73, 9173.81) | 0.86 (0.61, 1.12) | 187573.72 (133785.72, 246582.77) | 23.33 (16.63, 30.68) |
| High-middle SDI | 2010 | 7223.16 (5149.47, 9512.10) | 0.86 (0.61, 1.14) | 193762.33 (137606.68, 254231.68) | 23.57 (16.72, 30.94) |
| High-middle SDI | 2011 | 7519.81 (5404.11, 9884.91) | 0.88 (0.63, 1.15) | 201713.97 (143697.56, 262654.90) | 23.96 (17.06, 31.21) |
| High-middle SDI | 2012 | 7778.69 (5547.24, 10290.62) | 0.88 (0.63, 1.17) | 208589.70 (149354.74, 276194.58) | 24.21 (17.32, 32.11) |
| High-middle SDI | 2013 | 8184.90 (5905.68, 10723.19) | 0.91 (0.66, 1.19) | 219035.42 (156769.86, 288259.45) | 24.84 (17.80, 32.70) |
| High-middle SDI | 2014 | 8174.46 (5841.62, 10635.56) | 0.88 (0.63, 1.15) | 217809.40 (157643.61, 283238.36) | 24.13 (17.46, 31.38) |
| High-middle SDI | 2015 | 8589.69 (6115.12, 11294.47) | 0.91 (0.65, 1.19) | 228057.58 (163678.16, 298994.13) | 24.66 (17.74, 32.34) |
| High-middle SDI | 2016 | 8860.25 (6348.16, 11702.45) | 0.91 (0.65, 1.20) | 234425.10 (168750.05, 307180.44) | 24.74 (17.85, 32.39) |
| High-middle SDI | 2017 | 9136.29 (6511.74, 12062.71) | 0.91 (0.65, 1.21) | 241976.62 (174418.18, 319987.45) | 24.96 (18.01, 33.00) |
| High-middle SDI | 2018 | 9499.50 (6853.84, 12573.84) | 0.93 (0.67, 1.23) | 251684.95 (183047.75, 331029.41) | 25.38 (18.42, 33.40) |
| High-middle SDI | 2019 | 9730.19 (6923.20, 12759.74) | 0.93 (0.66, 1.21) | 257727.36 (184253.91, 338062.07) | 25.43 (18.18, 33.39) |
| High-middle SDI | 2020 | 9848.80 (6943.55, 13019.84) | 0.92 (0.65, 1.21) | 259739.28 (184993.96, 345830.84) | 25.08 (17.83, 33.45) |
| High-middle SDI | 2021 | 10214.55 (7254.84, 13456.15) | 0.93 (0.66, 1.22) | 269987.58 (191569.32, 355601.52) | 25.57 (18.19, 33.72) |
| Low SDI | 1990 | 264.49 (173.72, 373.08) | 0.23 (0.15, 0.33) | 8060.58 (5296.12, 11369.53) | 6.49 (4.28, 9.15) |
| Low SDI | 1991 | 272.53 (178.19, 385.11) | 0.23 (0.15, 0.33) | 8303.04 (5446.51, 11741.58) | 6.55 (4.29, 9.25) |
| Low SDI | 1992 | 281.39 (186.40, 395.17) | 0.24 (0.16, 0.34) | 8569.76 (5685.03, 11984.46) | 6.62 (4.38, 9.27) |
| Low SDI | 1993 | 291.47 (190.21, 410.50) | 0.24 (0.16, 0.34) | 8876.26 (5843.00, 12447.90) | 6.71 (4.40, 9.44) |
| Low SDI | 1994 | 302.15 (198.24, 427.64) | 0.25 (0.16, 0.35) | 9191.28 (6027.41, 12965.95) | 6.82 (4.49, 9.63) |
| Low SDI | 1995 | 311.55 (203.34, 441.53) | 0.25 (0.16, 0.35) | 9470.42 (6250.63, 13451.85) | 6.89 (4.53, 9.78) |
| Low SDI | 1996 | 321.54 (206.96, 457.47) | 0.25 (0.16, 0.36) | 9775.85 (6308.15, 13894.37) | 6.96 (4.49, 9.87) |
| Low SDI | 1997 | 331.17 (213.15, 471.51) | 0.25 (0.16, 0.36) | 10070.43 (6538.17, 14199.06) | 7.00 (4.53, 9.92) |
| Low SDI | 1998 | 342.74 (221.01, 486.82) | 0.25 (0.16, 0.36) | 10417.33 (6783.26, 14785.10) | 7.08 (4.59, 9.99) |
| Low SDI | 1999 | 353.97 (228.61, 499.53) | 0.26 (0.17, 0.36) | 10757.85 (6973.72, 15097.55) | 7.14 (4.63, 10.12) |
| Low SDI | 2000 | 366.42 (237.02, 518.57) | 0.26 (0.17, 0.37) | 11121.88 (7221.20, 15648.85) | 7.21 (4.68, 10.23) |
| Low SDI | 2001 | 379.48 (245.51, 532.88) | 0.26 (0.17, 0.37) | 11513.09 (7497.55, 16270.25) | 7.28 (4.72, 10.29) |
| Low SDI | 2002 | 393.45 (255.37, 551.13) | 0.27 (0.17, 0.37) | 11906.38 (7771.80, 16675.47) | 7.35 (4.79, 10.28) |
| Low SDI | 2003 | 409.75 (262.22, 571.48) | 0.27 (0.17, 0.38) | 12404.92 (8005.48, 17327.98) | 7.47 (4.78, 10.44) |
| Low SDI | 2004 | 426.20 (277.18, 593.15) | 0.27 (0.18, 0.38) | 12913.23 (8451.04, 18070.11) | 7.57 (4.91, 10.61) |
| Low SDI | 2005 | 441.66 (284.84, 615.33) | 0.28 (0.18, 0.39) | 13377.44 (8681.82, 18536.94) | 7.63 (4.93, 10.61) |
| Low SDI | 2006 | 459.67 (296.71, 650.76) | 0.28 (0.18, 0.39) | 13905.09 (8868.44, 19625.89) | 7.70 (4.94, 10.89) |
| Low SDI | 2007 | 477.61 (313.62, 676.16) | 0.28 (0.18, 0.40) | 14448.12 (9512.49, 20483.17) | 7.77 (5.12, 11.03) |
| Low SDI | 2008 | 498.35 (320.95, 707.00) | 0.28 (0.18, 0.41) | 15063.98 (9836.33, 21283.66) | 7.87 (5.09, 11.14) |
| Low SDI | 2009 | 520.98 (340.03, 750.50) | 0.29 (0.19, 0.42) | 15747.31 (10179.22, 22386.42) | 7.98 (5.17, 11.41) |
| Low SDI | 2010 | 543.87 (350.52, 770.22) | 0.29 (0.19, 0.42) | 16437.41 (10622.08, 23591.14) | 8.08 (5.19, 11.52) |
| Low SDI | 2011 | 570.71 (365.66, 812.57) | 0.30 (0.19, 0.43) | 17251.81 (11147.70, 24552.55) | 8.21 (5.30, 11.68) |
| Low SDI | 2012 | 601.73 (385.34, 848.63) | 0.30 (0.19, 0.43) | 18197.96 (11644.78, 25985.25) | 8.39 (5.37, 11.92) |
| Low SDI | 2013 | 634.25 (407.79, 912.94) | 0.31 (0.20, 0.44) | 19200.26 (12400.48, 27951.39) | 8.57 (5.53, 12.50) |
| Low SDI | 2014 | 669.77 (423.82, 956.62) | 0.32 (0.20, 0.45) | 20313.97 (12777.52, 29039.30) | 8.77 (5.55, 12.52) |
| Low SDI | 2015 | 703.35 (453.35, 1006.63) | 0.32 (0.21, 0.46) | 21355.51 (13702.62, 31044.85) | 8.93 (5.76, 12.89) |
| Low SDI | 2016 | 740.50 (472.16, 1079.44) | 0.33 (0.21, 0.48) | 22511.23 (14443.32, 32775.21) | 9.10 (5.83, 13.25) |
| Low SDI | 2017 | 780.13 (510.41, 1135.25) | 0.34 (0.22, 0.49) | 23726.50 (15546.62, 34369.21) | 9.28 (6.09, 13.45) |
| Low SDI | 2018 | 819.73 (536.92, 1181.30) | 0.34 (0.22, 0.49) | 24940.79 (16419.48, 36052.75) | 9.43 (6.21, 13.57) |
| Low SDI | 2019 | 863.76 (564.53, 1236.47) | 0.35 (0.23, 0.50) | 26307.57 (17249.81, 37703.05) | 9.62 (6.30, 13.74) |
| Low SDI | 2020 | 907.17 (593.58, 1291.61) | 0.35 (0.23, 0.50) | 27700.52 (18187.38, 39974.84) | 9.79 (6.42, 14.11) |
| Low SDI | 2021 | 947.04 (621.26, 1364.98) | 0.36 (0.23, 0.51) | 29006.95 (19141.18, 42163.99) | 9.94 (6.52, 14.40) |
| Low-middle SDI | 1990 | 739.99 (511.80, 1004.63) | 0.25 (0.17, 0.34) | 22214.71 (15281.96, 30099.69) | 6.72 (4.63, 9.14) |
| Low-middle SDI | 1991 | 773.73 (536.25, 1050.07) | 0.25 (0.17, 0.34) | 23245.48 (16029.83, 31699.89) | 6.85 (4.73, 9.32) |
| Low-middle SDI | 1992 | 807.76 (559.41, 1104.55) | 0.26 (0.18, 0.35) | 24249.46 (16716.36, 33183.52) | 6.97 (4.81, 9.58) |
| Low-middle SDI | 1993 | 839.45 (581.38, 1146.39) | 0.26 (0.18, 0.35) | 25161.72 (17366.50, 34210.56) | 7.05 (4.87, 9.63) |
| Low-middle SDI | 1994 | 883.71 (609.90, 1206.78) | 0.27 (0.18, 0.36) | 26432.37 (18277.13, 35936.79) | 7.23 (5.00, 9.84) |
| Low-middle SDI | 1995 | 915.83 (633.16, 1248.47) | 0.27 (0.19, 0.36) | 27355.12 (18938.38, 37379.96) | 7.30 (5.05, 9.98) |
| Low-middle SDI | 1996 | 956.87 (667.86, 1307.86) | 0.27 (0.19, 0.37) | 28580.15 (19822.12, 38862.24) | 7.43 (5.16, 10.12) |
| Low-middle SDI | 1997 | 1005.12 (703.75, 1364.45) | 0.28 (0.19, 0.38) | 30024.98 (20981.09, 40804.91) | 7.60 (5.32, 10.34) |
| Low-middle SDI | 1998 | 1047.36 (734.65, 1416.73) | 0.28 (0.20, 0.38) | 31278.22 (21894.42, 42329.68) | 7.71 (5.39, 10.43) |
| Low-middle SDI | 1999 | 1091.55 (753.21, 1495.95) | 0.29 (0.20, 0.39) | 32580.43 (22374.59, 44365.63) | 7.82 (5.38, 10.69) |
| Low-middle SDI | 2000 | 1147.50 (787.83, 1560.00) | 0.29 (0.20, 0.40) | 34210.65 (23335.59, 46273.06) | 8.00 (5.48, 10.84) |
| Low-middle SDI | 2001 | 1200.97 (827.27, 1622.30) | 0.30 (0.20, 0.40) | 35858.52 (24655.08, 48819.34) | 8.14 (5.59, 11.06) |
| Low-middle SDI | 2002 | 1270.45 (865.38, 1725.53) | 0.30 (0.21, 0.41) | 37883.85 (25736.29, 51066.29) | 8.36 (5.68, 11.31) |
| Low-middle SDI | 2003 | 1337.16 (917.07, 1806.31) | 0.31 (0.22, 0.42) | 39784.12 (27228.78, 53754.50) | 8.54 (5.85, 11.55) |
| Low-middle SDI | 2004 | 1389.70 (964.06, 1866.40) | 0.31 (0.22, 0.42) | 41332.16 (28470.86, 55181.08) | 8.62 (5.93, 11.50) |
| Low-middle SDI | 2005 | 1452.99 (1016.73, 1955.61) | 0.32 (0.22, 0.43) | 43176.66 (29701.95, 58224.20) | 8.74 (6.07, 11.76) |
| Low-middle SDI | 2006 | 1520.35 (1036.03, 2042.46) | 0.32 (0.22, 0.43) | 45052.95 (30668.96, 60460.00) | 8.86 (6.03, 11.90) |
| Low-middle SDI | 2007 | 1592.57 (1108.47, 2154.19) | 0.33 (0.23, 0.44) | 47131.93 (32563.10, 63177.15) | 9.00 (6.21, 12.08) |
| Low-middle SDI | 2008 | 1679.94 (1150.06, 2263.37) | 0.34 (0.23, 0.45) | 49706.71 (33997.30, 66693.57) | 9.21 (6.30, 12.36) |
| Low-middle SDI | 2009 | 1772.54 (1209.96, 2402.72) | 0.34 (0.23, 0.47) | 52435.36 (35684.97, 70320.34) | 9.43 (6.43, 12.68) |
| Low-middle SDI | 2010 | 1861.34 (1274.32, 2540.99) | 0.35 (0.24, 0.48) | 55036.81 (37128.13, 74383.68) | 9.60 (6.48, 13.01) |
| Low-middle SDI | 2011 | 1956.09 (1340.35, 2637.01) | 0.35 (0.24, 0.48) | 57862.97 (39637.97, 77559.94) | 9.77 (6.69, 13.11) |
| Low-middle SDI | 2012 | 2060.39 (1409.56, 2784.66) | 0.36 (0.25, 0.49) | 60907.30 (41630.00, 81625.91) | 9.97 (6.81, 13.38) |
| Low-middle SDI | 2013 | 2162.18 (1486.18, 2927.99) | 0.37 (0.25, 0.50) | 63915.55 (43547.79, 86503.49) | 10.13 (6.94, 13.74) |
| Low-middle SDI | 2014 | 2281.67 (1595.50, 3094.20) | 0.37 (0.26, 0.51) | 67445.60 (46831.20, 90751.00) | 10.37 (7.19, 13.96) |
| Low-middle SDI | 2015 | 2419.97 (1651.22, 3296.72) | 0.39 (0.26, 0.53) | 71510.14 (48789.89, 96813.58) | 10.66 (7.28, 14.44) |
| Low-middle SDI | 2016 | 2559.30 (1791.72, 3444.66) | 0.39 (0.28, 0.53) | 75599.87 (52364.52, 100202.92) | 10.93 (7.58, 14.51) |
| Low-middle SDI | 2017 | 2691.50 (1877.79, 3644.85) | 0.40 (0.28, 0.55) | 79237.05 (55036.88, 107348.19) | 11.11 (7.74, 15.08) |
| Low-middle SDI | 2018 | 2816.57 (1937.41, 3833.10) | 0.41 (0.28, 0.55) | 82732.90 (56669.03, 111674.89) | 11.26 (7.72, 15.22) |
| Low-middle SDI | 2019 | 2951.38 (2039.85, 3961.09) | 0.41 (0.29, 0.56) | 86487.82 (59761.32, 115965.44) | 11.43 (7.90, 15.32) |
| Low-middle SDI | 2020 | 3070.85 (2082.88, 4183.83) | 0.42 (0.28, 0.57) | 90050.10 (61224.11, 122569.46) | 11.57 (7.86, 15.71) |
| Low-middle SDI | 2021 | 3174.24 (2190.38, 4304.13) | 0.42 (0.29, 0.57) | 93283.28 (63671.85, 124563.54) | 11.67 (7.97, 15.63) |
| Middle SDI | 1990 | 1929.85 (1295.68, 2641.48) | 0.36 (0.24, 0.49) | 59297.69 (39260.70, 81784.94) | 10.18 (6.80, 14.03) |
| Middle SDI | 1991 | 2005.50 (1332.99, 2752.10) | 0.36 (0.24, 0.50) | 61534.80 (41216.18, 84354.53) | 10.28 (6.86, 14.10) |
| Middle SDI | 1992 | 2094.88 (1392.31, 2854.08) | 0.37 (0.25, 0.50) | 64167.38 (42648.24, 87725.19) | 10.44 (6.93, 14.25) |
| Middle SDI | 1993 | 2151.24 (1452.50, 2940.31) | 0.37 (0.25, 0.50) | 65760.93 (43754.41, 90553.40) | 10.42 (6.96, 14.32) |
| Middle SDI | 1994 | 2246.89 (1483.76, 3038.31) | 0.37 (0.25, 0.51) | 68575.74 (44511.90, 92729.27) | 10.59 (6.90, 14.26) |
| Middle SDI | 1995 | 2304.29 (1545.85, 3085.19) | 0.37 (0.25, 0.50) | 70284.70 (47019.89, 94632.94) | 10.58 (7.06, 14.20) |
| Middle SDI | 1996 | 2406.09 (1588.13, 3218.01) | 0.38 (0.25, 0.51) | 73355.74 (47631.36, 98514.74) | 10.75 (7.03, 14.42) |
| Middle SDI | 1997 | 2548.17 (1735.39, 3427.05) | 0.39 (0.27, 0.53) | 77413.10 (52631.01, 104023.28) | 11.05 (7.52, 14.88) |
| Middle SDI | 1998 | 2659.02 (1790.57, 3587.54) | 0.40 (0.27, 0.53) | 80779.43 (54490.06, 109245.68) | 11.22 (7.54, 15.17) |
| Middle SDI | 1999 | 2768.85 (1843.14, 3704.48) | 0.40 (0.27, 0.54) | 84024.50 (55866.32, 112824.64) | 11.35 (7.58, 15.23) |
| Middle SDI | 2000 | 2867.31 (1923.44, 3919.03) | 0.40 (0.27, 0.55) | 86928.16 (57793.48, 119736.17) | 11.42 (7.59, 15.66) |
| Middle SDI | 2001 | 2984.22 (2029.89, 4013.37) | 0.41 (0.28, 0.55) | 90330.91 (61405.75, 121901.71) | 11.52 (7.83, 15.49) |
| Middle SDI | 2002 | 3098.02 (2064.70, 4168.37) | 0.41 (0.28, 0.56) | 93877.92 (62903.58, 125915.88) | 11.61 (7.78, 15.55) |
| Middle SDI | 2003 | 3260.74 (2216.56, 4407.20) | 0.42 (0.29, 0.57) | 98780.02 (66640.26, 132910.28) | 11.85 (8.00, 15.92) |
| Middle SDI | 2004 | 3397.02 (2282.47, 4514.42) | 0.42 (0.28, 0.56) | 102957.50 (69663.40, 137183.15) | 11.97 (8.09, 15.93) |
| Middle SDI | 2005 | 3514.64 (2326.72, 4683.19) | 0.42 (0.28, 0.57) | 106480.29 (71382.31, 142106.13) | 12.00 (8.03, 15.95) |
| Middle SDI | 2006 | 3605.80 (2401.25, 4816.35) | 0.42 (0.28, 0.56) | 109301.41 (72482.52, 146585.95) | 11.92 (7.91, 15.97) |
| Middle SDI | 2007 | 3734.79 (2482.34, 4963.31) | 0.42 (0.28, 0.56) | 113163.54 (74696.33, 150513.27) | 11.94 (7.90, 15.90) |
| Middle SDI | 2008 | 3916.95 (2585.04, 5234.74) | 0.43 (0.28, 0.57) | 118485.87 (78084.88, 157831.05) | 12.10 (7.99, 16.08) |
| Middle SDI | 2009 | 4095.46 (2718.45, 5456.83) | 0.43 (0.29, 0.57) | 123775.00 (80825.67, 165036.91) | 12.22 (8.00, 16.28) |
| Middle SDI | 2010 | 4240.39 (2820.02, 5605.99) | 0.43 (0.29, 0.57) | 127916.65 (84544.29, 168904.45) | 12.23 (8.09, 16.19) |
| Middle SDI | 2011 | 4377.21 (2921.08, 5880.72) | 0.43 (0.29, 0.58) | 131803.66 (87724.77, 175775.52) | 12.19 (8.10, 16.29) |
| Middle SDI | 2012 | 4544.35 (3049.28, 6037.47) | 0.43 (0.29, 0.57) | 136537.18 (91169.66, 179271.52) | 12.22 (8.18, 16.04) |
| Middle SDI | 2013 | 4713.67 (3184.26, 6280.07) | 0.43 (0.29, 0.57) | 141358.48 (95493.78, 187395.42) | 12.24 (8.28, 16.23) |
| Middle SDI | 2014 | 4875.89 (3331.92, 6504.12) | 0.43 (0.29, 0.57) | 145756.21 (98843.02, 194511.04) | 12.22 (8.29, 16.31) |
| Middle SDI | 2015 | 5085.15 (3497.57, 6781.31) | 0.43 (0.30, 0.58) | 151417.14 (103535.70, 202632.22) | 12.31 (8.43, 16.47) |
| Middle SDI | 2016 | 5356.62 (3669.49, 7108.52) | 0.44 (0.30, 0.58) | 158825.45 (109433.75, 210144.68) | 12.52 (8.63, 16.55) |
| Middle SDI | 2017 | 5653.61 (3891.03, 7739.27) | 0.45 (0.31, 0.62) | 167078.45 (114636.08, 227265.30) | 12.77 (8.77, 17.37) |
| Middle SDI | 2018 | 5950.76 (4111.99, 7940.04) | 0.46 (0.32, 0.61) | 175558.76 (122419.52, 234561.45) | 13.01 (9.08, 17.37) |
| Middle SDI | 2019 | 6299.99 (4381.71, 8524.52) | 0.47 (0.32, 0.63) | 185243.62 (127249.66, 251168.94) | 13.33 (9.19, 18.07) |
| Middle SDI | 2020 | 6586.98 (4516.95, 8835.03) | 0.47 (0.32, 0.63) | 193230.18 (133665.36, 259809.07) | 13.52 (9.35, 18.18) |
| Middle SDI | 2021 | 6898.58 (4838.74, 9442.07) | 0.48 (0.34, 0.66) | 202165.57 (141665.94, 276176.84) | 13.78 (9.65, 18.81) |
