# Supplementary material for: High BMI-attributable female-specific cancers: a comprehensive analysis of the global disease burden and trends from 1990 to 2021 and projections to 2040
Source: Front Oncol. 2025 Oct 29;15:1704299. doi: 10.3389/fonc.2025.1704299 (PMC12605095; doi:10.3389/fonc.2025.1704299)
Supplement: Supplementary file 3 [file Table2.docx]

**Table S2**

Joinpoint analysis of ASDR in breast, ovarian, and uterine cancers, 1990–2021.

| **Breast cancer** | | | | | |
| --- | --- | --- | --- | --- | --- |
| location_name | Segment Start | Segment End | ASDR APC 95%CI | Test Statistic | P-Value |
| Global | 1990 | 1994 | 1.1 (0.77, 1.43) | 8.1347 | 0.000185 |
| Global | 1994 | 1997 | -0.38 (-1.41, 0.65) | -0.9136 | 0.396154 |
| Global | 1997 | 2000 | 0.38 (-0.65, 1.41) | 0.8976 | 0.403976 |
| Global | 2000 | 2003 | 0.07 (-0.95, 1.1) | 0.167 | 0.872884 |
| Global | 2003 | 2006 | -0.62 (-1.64, 0.41) | -1.475 | 0.190663 |
| Global | 2006 | 2010 | 0.06 (-0.45, 0.57) | 0.2983 | 0.775548 |
| Global | 2010 | 2014 | -0.18 (-0.68, 0.33) | -0.8424 | 0.431867 |
| Global | 2014 | 2018 | 0.37 (-0.14, 0.88) | 1.7552 | 0.129749 |
| Global | 2018 | 2021 | -0.1 (-0.61, 0.42) | -0.4589 | 0.662484 |
| High SDI | 1990 | 1995 | 0.13 (-0.15, 0.4) | 0.9907 | 0.337538 |
| High SDI | 1995 | 1998 | -1.32 (-2.49, -0.13) | -2.3615 | 0.032152 |
| High SDI | 1998 | 2001 | -0.37 (-1.55, 0.83) | -0.6614 | 0.518381 |
| High SDI | 2001 | 2014 | -0.93 (-1, -0.86) | -28.2279 | 0 |
| High SDI | 2014 | 2017 | 0.07 (-1.11, 1.27) | 0.1278 | 0.899998 |
| High SDI | 2017 | 2021 | -0.9 (-1.27, -0.52) | -5.0955 | 0.000132 |
| High-middle SDI | 1990 | 1994 | 2.63 (2.1, 3.16) | 11.417 | 0.000001 |
| High-middle SDI | 1994 | 1997 | 0.11 (-1.49, 1.74) | 0.1567 | 0.878924 |
| High-middle SDI | 1997 | 2000 | 1.63 (0, 3.28) | 2.2643 | 0.049829 |
| High-middle SDI | 2000 | 2003 | 0.56 (-1.05, 2.2) | 0.7879 | 0.451011 |
| High-middle SDI | 2003 | 2007 | -0.58 (-1.38, 0.23) | -1.6274 | 0.138099 |
| High-middle SDI | 2007 | 2010 | 0.17 (-1.41, 1.77) | 0.2405 | 0.815352 |
| High-middle SDI | 2010 | 2017 | -0.62 (-0.88, -0.35) | -5.2328 | 0.00054 |
| High-middle SDI | 2017 | 2021 | -0.17 (-0.67, 0.34) | -0.7433 | 0.476274 |
| Low SDI | 1990 | 1995 | 1.83 (1.71, 1.95) | 33.5357 | 0 |
| Low SDI | 1995 | 2001 | 1.2 (1.08, 1.32) | 21.9082 | 0 |
| Low SDI | 2001 | 2008 | 1.61 (1.52, 1.7) | 40.3446 | 0 |
| Low SDI | 2008 | 2011 | 2.57 (2.06, 3.09) | 10.9495 | 0 |
| Low SDI | 2011 | 2014 | 3.67 (3.15, 4.19) | 15.6354 | 0 |
| Low SDI | 2014 | 2019 | 2.89 (2.72, 3.06) | 37.8066 | 0 |
| Low SDI | 2019 | 2021 | 1.71 (1.18, 2.24) | 7.0945 | 0.000013 |
| Low-middle SDI | 1990 | 2001 | 2.88 (2.79, 2.96) | 72.042 | 0 |
| Low-middle SDI | 2001 | 2004 | 4.74 (3.45, 6.05) | 8.1617 | 0.000003 |
| Low-middle SDI | 2004 | 2009 | 3.22 (2.83, 3.62) | 18.1411 | 0 |
| Low-middle SDI | 2009 | 2013 | 2.56 (1.95, 3.18) | 9.1333 | 0.000001 |
| Low-middle SDI | 2013 | 2016 | 3.49 (2.26, 4.75) | 6.2183 | 0.000045 |
| Low-middle SDI | 2016 | 2019 | 2.38 (1.15, 3.62) | 4.2433 | 0.001141 |
| Low-middle SDI | 2019 | 2021 | 1.02 (-0.22, 2.28) | 1.794 | 0.098031 |
| Middle SDI | 1990 | 1995 | 2.82 (2.65, 2.99) | 36.4648 | 0 |
| Middle SDI | 1995 | 2000 | 1.92 (1.68, 2.16) | 17.4267 | 0 |
| Middle SDI | 2000 | 2006 | 1.35 (1.18, 1.53) | 17.3191 | 0 |
| Middle SDI | 2006 | 2011 | 2.17 (1.93, 2.41) | 19.6576 | 0 |
| Middle SDI | 2011 | 2014 | 1.08 (0.31, 1.85) | 3.0757 | 0.009615 |
| Middle SDI | 2014 | 2019 | 1.97 (1.72, 2.21) | 17.72 | 0 |
| Middle SDI | 2019 | 2021 | 1.25 (0.48, 2.02) | 3.5575 | 0.003942 |
| **Ovarian cancer** | | | | | |
| location_name | Segment Start | Segment End | ASDR APC 95%CI | Test Statistic | P-Value |
| Global | 1990 | 1995 | 1.28 (1.02, 1.55) | 10.3598 | 0 |
| Global | 1995 | 1998 | 0.14 (-1, 1.29) | 0.2545 | 0.802013 |
| Global | 1998 | 2003 | 1.21 (0.85, 1.58) | 7.0782 | 0.000001 |
| Global | 2003 | 2015 | -0.04 (-0.12, 0.03) | -1.148 | 0.265982 |
| Global | 2015 | 2021 | 0.5 (0.32, 0.68) | 5.7327 | 0.00002 |
| High-middle SDI | 1990 | 1995 | 2.08 (1.5, 2.66) | 7.56 | 0 |
| High-middle SDI | 1995 | 1998 | -0.78 (-3.23, 1.72) | -0.6563 | 0.518777 |
| High-middle SDI | 1998 | 2005 | 1.38 (0.96, 1.8) | 6.8349 | 0.000001 |
| High-middle SDI | 2005 | 2021 | -0.05 (-0.14, 0.04) | -1.1515 | 0.262469 |
| High SDI | 1990 | 2003 | 0.57 (0.46, 0.69) | 10.5936 | 0 |
| High SDI | 2003 | 2021 | -0.94 (-1, -0.87) | -29.8815 | 0 |
| Low-middle SDI | 1990 | 1994 | 4.21 (3.84, 4.59) | 25.2005 | 0 |
| Low-middle SDI | 1994 | 1997 | 6.76 (5.61, 7.93) | 13.1178 | 0 |
| Low-middle SDI | 1997 | 2000 | 4.97 (3.88, 6.08) | 10.0856 | 0 |
| Low-middle SDI | 2000 | 2004 | 6.42 (5.88, 6.96) | 26.708 | 0 |
| Low-middle SDI | 2004 | 2008 | 4.63 (4.12, 5.13) | 20.4143 | 0 |
| Low-middle SDI | 2008 | 2018 | 3.53 (3.44, 3.62) | 87.7144 | 0 |
| Low-middle SDI | 2018 | 2021 | 2.58 (2.11, 3.06) | 11.9897 | 0 |
| Low SDI | 1990 | 1999 | 2.87 (2.81, 2.94) | 90.592 | 0 |
| Low SDI | 1999 | 2011 | 3.58 (3.53, 3.62) | 166.461 | 0 |
| Low SDI | 2011 | 2017 | 4.38 (4.24, 4.52) | 66.9148 | 0 |
| Low SDI | 2017 | 2021 | 3.96 (3.77, 4.15) | 43.4815 | 0 |
| Middle SDI | 1990 | 1995 | 4.44 (4.26, 4.62) | 53.9253 | 0 |
| Middle SDI | 1995 | 1998 | 3.77 (3, 4.53) | 10.5313 | 0 |
| Middle SDI | 1998 | 2009 | 3.04 (2.98, 3.1) | 113.481 | 0 |
| Middle SDI | 2009 | 2013 | 2.59 (2.24, 2.94) | 15.7137 | 0 |
| Middle SDI | 2013 | 2021 | 3.16 (3.08, 3.23) | 91.0792 | 0 |
| **Uterine cancer** | | | | | |
| location_name | Segment Start | Segment End | ASDR APC 95%CI | Test Statistic | P-Value |
| Global | 1990 | 2004 | 0 (-0.06, 0.07) | 0.1086 | 0.914751 |
| Global | 2004 | 2007 | -1.49 (-2.8, -0.16) | -2.3592 | 0.029817 |
| Global | 2007 | 2014 | 0.78 (0.55, 1) | 7.2419 | 0.000001 |
| Global | 2014 | 2018 | 1.53 (0.86, 2.2) | 4.8182 | 0.000138 |
| Global | 2018 | 2021 | 0.07 (-0.58, 0.72) | 0.2255 | 0.824155 |
| High-middle SDI | 1990 | 1994 | 1.49 (0.58, 2.42) | 3.4287 | 0.002995 |
| High-middle SDI | 1994 | 2001 | -0.82 (-1.29, -0.35) | -3.6281 | 0.001923 |
| High-middle SDI | 2001 | 2004 | 0.66 (-2.06, 3.46) | 0.5064 | 0.618704 |
| High-middle SDI | 2004 | 2007 | -4.23 (-6.85, -1.54) | -3.2742 | 0.004214 |
| High-middle SDI | 2007 | 2021 | 0.62 (0.49, 0.75) | 9.8739 | 0 |
| High SDI | 1990 | 1996 | -0.61 (-0.85, -0.37) | -5.2665 | 0.000052 |
| High SDI | 1996 | 2008 | 0.33 (0.23, 0.42) | 7.3124 | 0.000001 |
| High SDI | 2008 | 2014 | 1.69 (1.39, 2) | 11.7841 | 0 |
| High SDI | 2014 | 2017 | 3.13 (1.76, 4.51) | 4.8512 | 0.000128 |
| High SDI | 2017 | 2021 | 0.07 (-0.35, 0.49) | 0.3417 | 0.736497 |
| Low-middle SDI | 1990 | 2000 | 1.68 (1.61, 1.75) | 56.4142 | 0 |
| Low-middle SDI | 2000 | 2003 | 2.05 (1.17, 2.94) | 5.2973 | 0.000496 |
| Low-middle SDI | 2003 | 2006 | 1.23 (0.39, 2.08) | 3.3221 | 0.00891 |
| Low-middle SDI | 2006 | 2009 | 2.15 (1.28, 3.02) | 5.6272 | 0.000323 |
| Low-middle SDI | 2009 | 2013 | 1.69 (1.25, 2.13) | 8.6983 | 0.000011 |
| Low-middle SDI | 2013 | 2016 | 2.51 (1.63, 3.4) | 6.5009 | 0.000111 |
| Low-middle SDI | 2016 | 2019 | 1.62 (0.75, 2.5) | 4.2396 | 0.002175 |
| Low-middle SDI | 2019 | 2021 | 0.74 (-0.14, 1.63) | 1.9008 | 0.089773 |
| Low SDI | 1990 | 1992 | 1.03 (0.65, 1.4) | 8.8013 | 0.00309 |
| Low SDI | 1992 | 1995 | 1.5 (1.13, 1.88) | 12.9401 | 0.000996 |
| Low SDI | 1995 | 1998 | 0.82 (0.44, 1.2) | 6.8441 | 0.006384 |
| Low SDI | 1998 | 2001 | 1 (0.62, 1.37) | 8.4728 | 0.003452 |
| Low SDI | 2001 | 2004 | 1.35 (0.98, 1.73) | 11.542 | 0.001396 |
| Low SDI | 2004 | 2007 | 0.98 (0.61, 1.35) | 8.4302 | 0.003503 |
| Low SDI | 2007 | 2011 | 1.4 (1.21, 1.59) | 23.4327 | 0.00017 |
| Low SDI | 2011 | 2014 | 2.32 (1.93, 2.7) | 19.2795 | 0.000305 |
| Low SDI | 2014 | 2019 | 1.82 (1.69, 1.94) | 47.8116 | 0.00002 |
| Low SDI | 2019 | 2021 | 1.48 (1.1, 1.86) | 12.4693 | 0.001112 |
| Middle SDI | 1990 | 1995 | 0.85 (0.51, 1.19) | 5.2473 | 0.000055 |
| Middle SDI | 1995 | 1998 | 1.95 (0.46, 3.47) | 2.7526 | 0.0131 |
| Middle SDI | 1998 | 2004 | 0.99 (0.65, 1.32) | 6.2245 | 0.000007 |
| Middle SDI | 2004 | 2015 | 0.25 (0.14, 0.36) | 4.624 | 0.000211 |
| Middle SDI | 2015 | 2021 | 1.79 (1.54, 2.04) | 15.3057 | 0 |
