# Supplementary material for: High BMI-attributable female-specific cancers: a comprehensive analysis of the global disease burden and trends from 1990 to 2021 and projections to 2040
Source: Front Oncol. 2025 Oct 29;15:1704299. doi: 10.3389/fonc.2025.1704299 (PMC12605095; doi:10.3389/fonc.2025.1704299)
Supplement: Supplementary file 4 [file Table3.docx]

**Table S3**

Joinpoint analysis of ASDALYR in breast, ovarian, and uterine cancers, 1990–2021.

| **Breast cancer** | | | | | |
| --- | --- | --- | --- | --- | --- |
| location_name | Segment Start | Segment End | ASDALYR  APC (95%CI) | Test Statistic | P-Value |
| Global | 1990 | 1994 | 1.17 (0.87, 1.47) | 8.5096 | 0.000002 |
| Global | 1994 | 1997 | -0.21 (-1.14, 0.72) | -0.5002 | 0.62599 |
| Global | 1997 | 2002 | 0.55 (0.26, 0.84) | 4.0871 | 0.001507 |
| Global | 2002 | 2007 | -0.2 (-0.49, 0.09) | -1.4936 | 0.161099 |
| Global | 2007 | 2010 | 0.33 (-0.58, 1.26) | 0.7887 | 0.445576 |
| Global | 2010 | 2014 | -0.3 (-0.76, 0.15) | -1.4458 | 0.173839 |
| Global | 2014 | 2021 | 0.07 (-0.06, 0.19) | 1.1803 | 0.260763 |
| High SDI | 1990 | 1994 | 2.77 (2.24, 3.3) | 11.9681 | 0.000001 |
| High SDI | 1994 | 1997 | -0.04 (-1.64, 1.59) | -0.0493 | 0.961775 |
| High SDI | 1997 | 2000 | 1.93 (0.3, 3.58) | 2.6827 | 0.025094 |
| High SDI | 2000 | 2004 | 0.55 (-0.25, 1.36) | 1.5534 | 0.154756 |
| High SDI | 2004 | 2007 | -0.86 (-2.43, 0.74) | -1.2158 | 0.254982 |
| High SDI | 2007 | 2010 | 0.32 (-1.26, 1.94) | 0.4595 | 0.656748 |
| High SDI | 2010 | 2017 | -0.77 (-1.04, -0.5) | -6.5296 | 0.000108 |
| High SDI | 2017 | 2021 | 0.05 (-0.45, 0.55) | 0.215 | 0.834567 |
| High-middle SDI | 1990 | 1994 | 0.35 (-0.1, 0.8) | 1.6198 | 0.118347 |
| High-middle SDI | 1994 | 2004 | -0.52 (-0.65, -0.4) | -8.5459 | 0 |
| High-middle SDI | 2004 | 2021 | -0.81 (-0.85, -0.76) | -34.3866 | 0 |
| Low SDI | 1990 | 2000 | 3.08 (2.94, 3.21) | 47.3827 | 0 |
| Low SDI | 2000 | 2006 | 4.8 (4.42, 5.17) | 27.2264 | 0 |
| Low SDI | 2006 | 2018 | 3.05 (2.94, 3.16) | 58.3668 | 0 |
| Low SDI | 2018 | 2021 | 1.27 (0.44, 2.1) | 3.196 | 0.004344 |
| Low-middle SDI | 1990 | 1995 | 1.79 (1.65, 1.94) | 27.5254 | 0 |
| Low-middle SDI | 1995 | 2001 | 1.16 (1.02, 1.3) | 17.744 | 0 |
| Low-middle SDI | 2001 | 2008 | 1.61 (1.51, 1.72) | 33.5504 | 0 |
| Low-middle SDI | 2008 | 2011 | 2.71 (2.09, 3.33) | 9.6752 | 0.000001 |
| Low-middle SDI | 2011 | 2014 | 3.62 (2.99, 4.24) | 12.825 | 0 |
| Low-middle SDI | 2014 | 2019 | 3 (2.8, 3.19) | 33.6914 | 0 |
| Low-middle SDI | 2019 | 2021 | 1.86 (1.25, 2.48) | 6.6356 | 0.000024 |
| Middle SDI | 1990 | 1994 | 3.14 (2.88, 3.4) | 25.704 | 0 |
| Middle SDI | 1994 | 2000 | 2.31 (2.12, 2.5) | 26.4039 | 0 |
| Middle SDI | 2000 | 2005 | 1.37 (1.11, 1.64) | 11.1452 | 0 |
| Middle SDI | 2005 | 2011 | 2.44 (2.26, 2.63) | 28.4239 | 0 |
| Middle SDI | 2011 | 2014 | 1.19 (0.38, 2.01) | 3.1451 | 0.006673 |
| Middle SDI | 2014 | 2021 | 1.79 (1.68, 1.9) | 35.3252 | 0 |
| **Ovarian cancer** | | | | | |
| location_name | Segment Start | Segment End | ASDALYR  APC (95%CI) | Test Statistic | P-Value |
| Global | 1990 | 1995 | 1.24 (1.02, 1.45) | 12.0982 | 0 |
| Global | 1995 | 1999 | 0.2 (-0.27, 0.67) | 0.8875 | 0.386519 |
| Global | 1999 | 2002 | 1.71 (0.77, 2.66) | 3.831 | 0.001224 |
| Global | 2002 | 2015 | 0.16 (0.1, 0.21) | 6.2007 | 0.000007 |
| Global | 2015 | 2021 | 0.79 (0.64, 0.94) | 11.1194 | 0 |
| High SDI | 1990 | 1995 | 1.98 (1.42, 2.55) | 7.3804 | 0 |
| High SDI | 1995 | 1998 | -1.39 (-3.77, 1.04) | -1.1957 | 0.245157 |
| High SDI | 1998 | 2003 | 1.47 (0.7, 2.25) | 3.9652 | 0.000706 |
| High SDI | 2003 | 2021 | 0.01 (-0.06, 0.09) | 0.3279 | 0.746237 |
| High-middle SDI | 1990 | 2003 | 0.47 (0.35, 0.58) | 8.1831 | 0 |
| High-middle SDI | 2003 | 2021 | -0.98 (-1.05, -0.91) | -29.7658 | 0 |
| Low SDI | 1990 | 1994 | 4.31 (4.02, 4.6) | 33.0249 | 0 |
| Low SDI | 1994 | 1997 | 6.74 (5.84, 7.64) | 16.8941 | 0 |
| Low SDI | 1997 | 2000 | 5.09 (4.23, 5.96) | 13.1482 | 0 |
| Low SDI | 2000 | 2004 | 6.36 (5.94, 6.78) | 34.0361 | 0 |
| Low SDI | 2004 | 2008 | 4.6 (4.2, 4.99) | 25.9679 | 0 |
| Low SDI | 2008 | 2018 | 3.54 (3.47, 3.61) | 113.5395 | 0 |
| Low SDI | 2018 | 2021 | 2.51 (2.14, 2.87) | 15.1121 | 0 |
| Low-middle SDI | 1990 | 2000 | 2.87 (2.81, 2.93) | 101.8859 | 0 |
| Low-middle SDI | 2000 | 2011 | 3.52 (3.47, 3.57) | 137.7934 | 0 |
| Low-middle SDI | 2011 | 2016 | 4.4 (4.19, 4.61) | 45.3226 | 0 |
| Low-middle SDI | 2016 | 2021 | 4.08 (3.94, 4.22) | 60.4998 | 0 |
| Middle SDI | 1990 | 1994 | 4.44 (4.18, 4.7) | 36.1659 | 0 |
| Middle SDI | 1994 | 1998 | 3.8 (3.4, 4.21) | 20.1755 | 0 |
| Middle SDI | 1998 | 2010 | 3.01 (2.96, 3.06) | 125.4735 | 0 |
| Middle SDI | 2010 | 2013 | 2.73 (2.01, 3.46) | 7.9818 | 0 |
| Middle SDI | 2013 | 2021 | 3.3 (3.22, 3.38) | 91.7353 | 0 |
| **Uterine cancer** | | | | | |
| location_name | Segment Start | Segment End | ASDALYR  APC (95%CI) | Test Statistic | P-Value |
| Global | 1990 | 1994 | 0.47 (0.06, 0.89) | 2.4712 | 0.029427 |
| Global | 1994 | 1998 | -0.34 (-0.98, 0.3) | -1.167 | 0.265873 |
| Global | 1998 | 2004 | 0.33 (0.05, 0.61) | 2.5328 | 0.026283 |
| Global | 2004 | 2007 | -1.4 (-2.63, -0.15) | -2.4466 | 0.030786 |
| Global | 2007 | 2014 | 0.83 (0.61, 1.04) | 8.4517 | 0.000002 |
| Global | 2014 | 2018 | 1.49 (0.86, 2.12) | 5.1904 | 0.000225 |
| Global | 2018 | 2021 | 0.24 (-0.39, 0.87) | 0.8324 | 0.42142 |
| High SDI | 1990 | 1994 | 1.58 (0.61, 2.55) | 3.4471 | 0.002875 |
| High SDI | 1994 | 2001 | -1.06 (-1.55, -0.56) | -4.4899 | 0.000283 |
| High SDI | 2001 | 2004 | 0.9 (-1.95, 3.85) | 0.658 | 0.518877 |
| High SDI | 2004 | 2007 | -4.57 (-7.27, -1.79) | -3.4199 | 0.003054 |
| High SDI | 2007 | 2021 | 0.63 (0.49, 0.77) | 9.7144 | 0 |
| High-middle SDI | 1990 | 1996 | -0.43 (-0.69, -0.16) | -3.4174 | 0.003071 |
| High-middle SDI | 1996 | 2008 | 0.74 (0.64, 0.84) | 15.2588 | 0 |
| High-middle SDI | 2008 | 2014 | 1.86 (1.53, 2.2) | 11.7936 | 0 |
| High-middle SDI | 2014 | 2017 | 3.15 (1.66, 4.67) | 4.4814 | 0.000289 |
| High-middle SDI | 2017 | 2021 | 0 (-0.45, 0.46) | 0.0103 | 0.991877 |
| Low SDI | 1990 | 1999 | 1.7 (1.62, 1.77) | 48.6984 | 0 |
| Low SDI | 1999 | 2003 | 2.15 (1.73, 2.56) | 11.0896 | 0 |
| Low SDI | 2003 | 2006 | 1.28 (0.49, 2.08) | 3.4498 | 0.003573 |
| Low SDI | 2006 | 2013 | 1.98 (1.84, 2.11) | 30.9582 | 0 |
| Low SDI | 2013 | 2017 | 2.35 (1.95, 2.75) | 12.6852 | 0 |
| Low SDI | 2017 | 2021 | 1.19 (0.93, 1.45) | 9.9204 | 0 |
| Low-middle SDI | 1990 | 1992 | 1.06 (0.68, 1.43) | 6.3681 | 0.00013 |
| Low-middle SDI | 1992 | 1995 | 1.36 (0.99, 1.74) | 8.2264 | 0.000018 |
| Low-middle SDI | 1995 | 2001 | 0.89 (0.8, 0.97) | 23.4565 | 0 |
| Low-middle SDI | 2001 | 2004 | 1.32 (0.94, 1.7) | 7.9838 | 0.000022 |
| Low-middle SDI | 2004 | 2007 | 0.89 (0.51, 1.27) | 5.3417 | 0.000467 |
| Low-middle SDI | 2007 | 2011 | 1.36 (1.17, 1.56) | 15.9413 | 0 |
| Low-middle SDI | 2011 | 2014 | 2.29 (1.89, 2.69) | 12.979 | 0 |
| Low-middle SDI | 2014 | 2021 | 1.83 (1.77, 1.88) | 77.8703 | 0 |
| Middle SDI | 1990 | 1995 | 0.76 (0.5, 1.02) | 6.4691 | 0.000031 |
| Middle SDI | 1995 | 1998 | 1.84 (0.73, 2.96) | 3.6237 | 0.003491 |
| Middle SDI | 1998 | 2004 | 1.06 (0.82, 1.31) | 9.4399 | 0.000001 |
| Middle SDI | 2004 | 2007 | 0.1 (-0.98, 1.19) | 0.2063 | 0.840039 |
| Middle SDI | 2007 | 2010 | 0.69 (-0.4, 1.8) | 1.3819 | 0.192194 |
| Middle SDI | 2010 | 2015 | 0.07 (-0.26, 0.41) | 0.4776 | 0.641524 |
| Middle SDI | 2015 | 2021 | 1.99 (1.81, 2.17) | 24.0136 | 0 |
