# Supplementary material for: High BMI-attributable female-specific cancers: a comprehensive analysis of the global disease burden and trends from 1990 to 2021 and projections to 2040
Source: Front Oncol. 2025 Oct 29;15:1704299. doi: 10.3389/fonc.2025.1704299 (PMC12605095; doi:10.3389/fonc.2025.1704299)
Supplement: Supplementary file 5 [file Table4.docx]

| **Table S4**  The high BMI-attributable breast, ovarian, and uterine cancer ASDR and ASDALYR for all age groups in 204 GBD countries in 1990 and 2021. | | | | | | | | | | | | |
| --- | --- | --- | --- | --- | --- | --- | --- | --- | --- | --- | --- | --- |
|  | Breast cancer | | | | Ovarian cancer | | | | Uterine cancer | | | |
|  | ASDR | | ASRDALY | | ASDR | | ASRDALY | | ASDR | | ASRDALY | |
| location | 1990 | 2021 | 1990 | 2021 | 1990 | 2021 | 1990 | 2021 | 1990 | 2021 | 1990 | 2021 |
| Afghanistan | 0.37 (-0.02,0.9) | 0.58 (-0.04,1.37) | 8.38 (-1.11,21.06) | 12.84 (-1.85,33.84) | 0.13 (0.02,0.41) | 0.23 (0.04,0.61) | 4.28 (0.55,13.79) | 7.49 (1.45,19.39) | 0.53 (0.22,1.13) | 0.75 (0.35,1.4) | 15.89 (6.36,35.05) | 22.65 (10.15,42.9) |
| Albania | 0.59 (-0.02,1.27) | 0.89 (-0.02,1.92) | 11.5 (-0.93,24.58) | 17.91 (-0.96,38.71) | 0.19 (0.04,0.37) | 0.29 (0.07,0.57) | 5.17 (1.07,10.4) | 7.78 (1.85,15.22) | 0.81 (0.48,1.25) | 0.8 (0.44,1.23) | 20.06 (12.17,30.66) | 19.6 (11.13,30) |
| Algeria | 0.39 (-0.01,0.81) | 0.67 (-0.03,1.41) | 7.14 (-0.6,15.02) | 13.09 (-1.09,27.98) | 0.12 (0.02,0.24) | 0.25 (0.07,0.47) | 3.35 (0.68,6.7) | 6.86 (1.81,12.54) | 0.15 (0.09,0.24) | 0.22 (0.13,0.33) | 3.82 (2.31,5.92) | 5.47 (3.34,8.41) |
| American Samoa | 2.41 (-0.07,4.79) | 4.38 (-0.15,8.81) | 62.72 (-1.88,121.67) | 112.96 (-4.19,222.5) | 0.26 (0.07,0.49) | 0.56 (0.16,1.04) | 8.73 (2.24,16.36) | 18.19 (5.36,33.95) | 1.58 (0.87,2.33) | 2.55 (1.09,4.94) | 46.92 (25.34,68.99) | 76.56 (33.01,144.79) |
| Andorra | 1.38 (-0.05,3) | 1.29 (-0.04,2.87) | 30.66 (-1.91,66.92) | 29.82 (-1.5,66.09) | 0.19 (0.03,0.41) | 0.18 (0.04,0.36) | 5 (0.82,11.07) | 4.71 (1.02,9.76) | 0.55 (0.29,0.93) | 0.48 (0.28,0.79) | 13.97 (7.59,24.06) | 12.59 (6.94,20.85) |
| Angola | 0.29 (-0.01,0.65) | 0.71 (-0.03,1.68) | 6.23 (-0.94,16.01) | 15.9 (-1.47,37.3) | 0.03 (0,0.09) | 0.16 (0.02,0.34) | 1.07 (-0.02,2.78) | 5.02 (0.75,10.83) | 0.24 (0.14,0.37) | 0.44 (0.24,0.78) | 6.86 (4.09,10.94) | 12.47 (6.55,21.74) |
| Argentina | 2.18 (-0.07,4.4) | 2.25 (-0.07,4.36) | 46.85 (-2.82,92.66) | 49.08 (-2.02,93.54) | 0.64 (0.15,1.21) | 0.71 (0.19,1.27) | 18.2 (4.13,34.54) | 20.15 (5.44,35.71) | 1.12 (0.79,1.54) | 0.93 (0.65,1.24) | 29.59 (20.74,39.91) | 23.85 (16.78,31.27) |
| Armenia | 2.02 (-0.07,3.99) | 2.17 (-0.07,4.37) | 47.28 (-3.33,89.41) | 51.27 (-1.82,102.35) | 0.47 (0.11,0.87) | 0.73 (0.2,1.31) | 13.69 (3.21,26.03) | 20.19 (5.51,36.53) | 1.56 (1.09,2.04) | 1.78 (1.2,2.43) | 45.03 (31.55,59) | 47.1 (32.19,63.98) |
| Australia | 1.58 (-0.05,3.2) | 1.39 (-0.04,2.76) | 36.47 (-1.86,72.45) | 32.08 (-1.22,62.83) | 0.87 (0.19,1.63) | 0.63 (0.17,1.13) | 24.26 (5.12,45.55) | 16.08 (4.28,28.7) | 0.57 (0.4,0.79) | 0.81 (0.57,1.08) | 14.64 (10.19,20.23) | 21.08 (14.97,27.87) |
| Austria | 1.65 (-0.05,3.36) | 1.29 (-0.04,2.7) | 35.81 (-1.87,70.56) | 26.9 (-0.92,53.87) | 0.72 (0.14,1.37) | 0.51 (0.11,0.98) | 18.49 (3.53,35.23) | 12.24 (2.65,23.1) | 1.03 (0.72,1.39) | 0.83 (0.57,1.14) | 25.21 (17.74,34.08) | 20.53 (14.17,28.45) |
| Azerbaijan | 0.91 (-0.05,1.75) | 1.12 (-0.04,2.22) | 19.93 (-2.7,39.78) | 27.4 (-1.69,56.07) | 0.22 (0.04,0.45) | 0.38 (0.09,0.77) | 7.18 (1.47,14.55) | 11.96 (2.79,24.8) | 1.03 (0.65,1.43) | 0.94 (0.59,1.42) | 30.96 (19.81,43.12) | 27.28 (17.06,40.36) |
| Bahamas | 1.97 (-0.08,3.96) | 2.84 (-0.1,5.54) | 43.08 (-4.04,87.49) | 58.92 (-4.8,117.45) | 0.68 (0.16,1.33) | 1.13 (0.3,2.07) | 22.17 (5.14,43.3) | 35.8 (9.6,65.14) | 1.37 (0.95,1.83) | 2.51 (1.71,3.46) | 41.29 (28.64,54.9) | 71.02 (47.87,97.18) |
| Bahrain | 1.85 (-0.06,3.76) | 2.72 (-0.1,5.25) | 44.09 (-2.34,90.54) | 62.67 (-3.4,122.27) | 0.73 (0.16,1.45) | 1.35 (0.41,2.54) | 21.03 (4.82,41.91) | 36.75 (11.28,67.96) | 0.86 (0.52,1.37) | 1.2 (0.76,1.75) | 23.24 (14.19,36.9) | 31.75 (19.69,47.53) |
| Bangladesh | 0.03 (-0.02,0.08) | 0.1 (-0.02,0.23) | 0.16 (-1.3,1.82) | 1.44 (-2.39,5.77) | 0.01 (-0.01,0.03) | 0.11 (0.01,0.24) | 0.26 (-0.37,1.12) | 3.65 (0.41,8.22) | 0.06 (0.04,0.12) | 0.12 (0.06,0.31) | 1.77 (1.02,3.27) | 3.6 (1.75,9.03) |
| Barbados | 1.89 (-0.07,3.84) | 3.21 (-0.12,6.47) | 39.91 (-3.6,81.68) | 70.37 (-3.98,140) | 0.59 (0.13,1.16) | 0.93 (0.26,1.71) | 18.4 (4.02,35.32) | 27.53 (8.23,49.29) | 1.66 (1.13,2.24) | 2.94 (1.95,4.15) | 46.72 (32.34,63.2) | 77.86 (51.79,109.88) |
| Barbuda | 1.43 (-0.05,2.85) | 2.67 (-0.09,5.24) | 31.32 (-2.31,61.92) | 61.4 (-2.4,119.07) | 0.37 (0.07,0.68) | 0.84 (0.2,1.51) | 11.4 (2.34,21.5) | 24.19 (5.92,44.27) | 0.88 (0.59,1.19) | 2.1 (1.51,2.75) | 24.93 (16.99,34.05) | 55.15 (40.02,71.93) |
| Belarus | 0.99 (-0.04,1.95) | 1.35 (-0.04,2.79) | 23.48 (-1.65,46.88) | 33.01 (-1.49,65.42) | 0.49 (0.11,0.93) | 0.77 (0.18,1.45) | 14.74 (3.31,27.93) | 22.8 (5.31,42.73) | 1.03 (0.72,1.39) | 1.76 (1.19,2.48) | 29.1 (20.35,39.35) | 50.02 (33.33,69.72) |
| Belgium | 1.91 (-0.06,3.92) | 1.56 (-0.04,3.09) | 41.99 (-2.35,83.47) | 34.63 (-1.13,68.35) | 0.65 (0.13,1.22) | 0.54 (0.12,0.99) | 17.42 (3.44,32.57) | 13.54 (2.96,24.34) | 0.74 (0.51,0.99) | 0.91 (0.63,1.22) | 19.04 (13.16,25.45) | 22.62 (15.81,30.23) |
| Belize | 0.65 (-0.02,1.3) | 1.14 (-0.04,2.24) | 14.2 (-1.22,28.29) | 26.02 (-1.82,50.45) | 0.21 (0.05,0.38) | 0.4 (0.12,0.7) | 6.72 (1.63,11.78) | 12.7 (3.77,21.65) | 1.36 (0.93,1.87) | 2.19 (1.55,2.83) | 37.93 (26.47,52.29) | 62.01 (44.51,79.96) |
| Benin | 0.56 (-0.02,1.19) | 0.75 (-0.03,1.55) | 13.6 (-0.86,28.61) | 17.21 (-0.92,35.79) | 0.1 (0.02,0.2) | 0.17 (0.04,0.32) | 3.11 (0.61,6.15) | 5.08 (1.16,10.09) | 0.51 (0.32,0.8) | 0.66 (0.39,1) | 14 (8.64,22.07) | 17.1 (10.08,26.67) |
| Bermuda | 2.66 (-0.09,5.36) | 1.91 (-0.08,3.92) | 57.99 (-2.61,114.6) | 41.15 (-2.26,81.27) | 0.87 (0.18,1.7) | 0.98 (0.28,1.78) | 24.8 (5.17,47.57) | 27.35 (8.23,49.1) | 1.48 (1.01,2.02) | 1.55 (1.08,2.21) | 38.67 (26.37,52.56) | 41 (28.42,57.93) |
| Bhutan | 0.25 (-0.02,0.54) | 0.38 (-0.02,0.82) | 5.42 (-0.94,12.63) | 8.06 (-0.89,18.49) | 0.12 (0.01,0.28) | 0.27 (0.05,0.63) | 4.01 (0.48,9.29) | 8.74 (1.6,20.81) | 0.23 (0.12,0.44) | 0.28 (0.14,0.67) | 6.74 (3.33,12.66) | 7.68 (3.71,18.68) |
| Bolivia | 0.71 (-0.02,1.72) | 1.25 (-0.04,2.9) | 15.52 (-1.23,40.02) | 28.19 (-1.77,65.82) | 0.14 (0.02,0.36) | 0.47 (0.12,0.94) | 4.52 (0.6,11.1) | 14.42 (3.54,28.76) | 1.24 (0.68,2.12) | 1.62 (0.84,2.58) | 35.04 (18.68,59.98) | 43.35 (23.29,70.48) |
| Bosnia and Herzegovina | 0.78 (-0.02,1.62) | 1.56 (-0.05,3.1) | 17.75 (-1.28,37.22) | 37.04 (-1.36,72.94) | 0.41 (0.09,0.78) | 0.72 (0.18,1.32) | 12.24 (2.66,23) | 20.3 (5.17,37.34) | 0.81 (0.52,1.18) | 1.07 (0.66,1.53) | 21.78 (13.94,31.45) | 28.19 (17.41,40.64) |
| Botswana | 0.83 (-0.03,1.88) | 1.63 (-0.05,3.21) | 17.55 (-1.33,41.45) | 33.66 (-1.23,65.7) | 0.25 (0.05,0.53) | 0.59 (0.14,1.1) | 7.2 (1.59,16.01) | 16.4 (3.78,31.67) | 0.71 (0.39,1.15) | 1.15 (0.7,1.74) | 18.09 (9.82,29.56) | 27.2 (16.31,43.33) |
| Brazil | 0.93 (-0.03,1.86) | 1.19 (-0.04,2.37) | 20.02 (-1.51,40) | 25.48 (-1.9,50.19) | 0.29 (0.06,0.55) | 0.46 (0.11,0.85) | 8.61 (1.8,16.73) | 13.58 (3.2,24.81) | 0.83 (0.58,1.14) | 0.91 (0.64,1.22) | 21.27 (14.98,29.11) | 23.89 (16.84,31.52) |
| Brunei | 0.63 (-0.02,1.32) | 1.08 (-0.03,2.21) | 19.3 (-0.56,40.95) | 33.09 (-0.86,68.07) | 0.21 (0.01,0.47) | 0.56 (0.13,1) | 7.54 (0.62,16.5) | 19.17 (4.56,34.83) | 0.51 (0.3,0.82) | 0.84 (0.51,1.25) | 15.67 (9,25.41) | 25.42 (15.71,38.17) |
| Bulgaria | 1.32 (-0.05,2.69) | 1.95 (-0.08,4.06) | 29.47 (-2.15,60.15) | 44.95 (-1.97,93.28) | 0.58 (0.14,1.05) | 0.98 (0.23,1.86) | 17.71 (4.22,31.77) | 29.08 (7.02,55.46) | 1.77 (1.16,2.52) | 2.39 (1.56,3.41) | 50.06 (32.97,71.14) | 66.95 (43.81,94.58) |
| Burkina Faso | 0.2 (-0.03,0.46) | 0.33 (-0.03,0.71) | 1.86 (-3.71,7.42) | 4.7 (-2.55,12.81) | 0.01 (-0.01,0.03) | 0.02 (-0.01,0.06) | 0.28 (-0.26,0.96) | 0.82 (-0.15,2.21) | 0.15 (0.09,0.23) | 0.2 (0.12,0.33) | 3.99 (2.47,6.32) | 5.33 (3.15,8.79) |
| Burundi | 0.28 (-0.01,0.63) | 0.38 (-0.01,0.8) | 5.85 (-0.79,14.19) | 7.66 (-0.72,16.69) | 0.04 (-0.02,0.12) | 0.11 (0.01,0.25) | 1.27 (-0.46,3.81) | 3.12 (0.18,7.32) | 0.24 (0.12,0.4) | 0.28 (0.15,0.46) | 6.6 (3.06,10.93) | 7.43 (4.04,12) |
| Cambodia | 0.27 (-0.01,0.65) | 0.58 (-0.01,1.25) | 7.95 (-0.22,19.32) | 16.64 (-0.36,36.17) | 0.03 (-0.01,0.08) | 0.13 (0,0.3) | 0.89 (-0.32,2.66) | 4.39 (0.12,10.25) | 0.25 (0.12,0.41) | 0.38 (0.21,0.6) | 7.81 (3.71,13.22) | 11.8 (6.35,19.02) |
| Cameroon | 0.91 (-0.03,1.94) | 1.49 (-0.04,3.29) | 19.91 (-1.32,43.29) | 32.2 (-1.66,69.42) | 0.15 (0.03,0.3) | 0.3 (0.07,0.61) | 4.44 (0.94,8.89) | 8.78 (2.01,17.74) | 0.8 (0.47,1.25) | 1.05 (0.58,1.71) | 20.5 (12.16,31.98) | 25.75 (14.32,42.35) |
| Canada | 1.93 (-0.06,3.79) | 1.32 (-0.04,2.59) | 44.71 (-1.63,88.28) | 29.4 (-1.08,56.7) | 0.62 (0.14,1.16) | 0.62 (0.16,1.12) | 17.21 (3.92,32.32) | 16.04 (4.19,28.76) | 0.99 (0.69,1.36) | 1.14 (0.8,1.53) | 25.29 (17.43,34.54) | 30.04 (21.22,40.16) |
| Cape Verde | 0.48 (-0.02,1) | 0.92 (-0.04,1.95) | 8.82 (-1.62,20.64) | 18.86 (-1.61,41.98) | 0.03 (0.01,0.06) | 0.2 (0.04,0.41) | 0.97 (0.18,2.02) | 5.95 (1.33,12.28) | 0.59 (0.35,0.9) | 0.99 (0.61,1.54) | 14.89 (9.07,22.99) | 22.84 (14.07,35.27) |
| Central African Republic | 0.34 (-0.01,0.75) | 0.64 (-0.03,1.36) | 7.74 (-0.92,17.67) | 14.8 (-1.13,31.63) | 0.05 (0,0.11) | 0.14 (0.02,0.3) | 1.48 (0.03,3.68) | 4.43 (0.78,9.57) | 0.29 (0.17,0.44) | 0.49 (0.27,0.81) | 8.48 (4.99,13.28) | 13.97 (7.57,23.33) |
| Chad | 0.31 (-0.01,0.65) | 0.55 (-0.02,1.2) | 7.22 (-0.35,15.6) | 12.79 (-0.67,27.78) | 0.04 (0.01,0.09) | 0.09 (0.01,0.2) | 1.22 (0.18,2.63) | 2.63 (0.41,5.78) | 0.31 (0.18,0.5) | 0.54 (0.31,0.86) | 8 (4.7,12.97) | 13.78 (8.05,21.72) |
| Chile | 1.32 (-0.04,2.61) | 1.19 (-0.04,2.31) | 28.16 (-1.29,56.4) | 25.3 (-1.27,48.95) | 0.44 (0.1,0.82) | 0.61 (0.16,1.06) | 12.76 (2.82,23.85) | 17.72 (4.65,30.7) | 0.76 (0.53,1.03) | 0.71 (0.5,0.94) | 19.14 (13.3,25.95) | 17.92 (12.55,23.62) |
| China | 0.26 (-0.01,0.53) | 0.49 (-0.02,1.07) | 7.15 (-0.19,14.93) | 14.39 (-0.47,30.83) | 0.03 (-0.02,0.08) | 0.16 (0.03,0.32) | 0.98 (-0.47,2.67) | 4.85 (0.96,9.92) | 0.27 (0.17,0.41) | 0.33 (0.2,0.51) | 8.44 (5.18,12.68) | 10.2 (6.1,15.89) |
| Colombia | 0.8 (-0.03,1.6) | 1.04 (-0.04,2.1) | 17.18 (-1.3,34.2) | 23.52 (-1.93,47.19) | 0.33 (0.06,0.64) | 0.59 (0.14,1.07) | 9.63 (1.86,18.75) | 17.7 (4.19,32.81) | 0.55 (0.37,0.75) | 0.71 (0.47,1.01) | 14.44 (9.73,19.9) | 19.26 (12.86,27.24) |
| Comoros | 0.41 (-0.02,0.9) | 0.98 (-0.03,2.1) | 7.29 (-1.17,17.23) | 17.98 (-2.08,40.39) | 0.09 (0,0.23) | 0.36 (0.06,0.78) | 2.88 (-0.09,6.96) | 11.05 (1.83,23.83) | 0.4 (0.2,0.64) | 0.65 (0.38,1.02) | 10.64 (5.33,17.39) | 17.19 (9.89,26.57) |
| Cook Islands | 3.66 (-0.1,7.6) | 4.33 (-0.14,8.56) | 93.84 (-2.35,195.65) | 108.58 (-3.72,214.33) | 0.15 (0.03,0.29) | 0.25 (0.07,0.44) | 4.88 (1.19,9.17) | 7.91 (2.3,14) | 0.79 (0.49,1.23) | 0.71 (0.46,1.02) | 23.4 (14.73,36.13) | 20.97 (13.61,30.5) |
| Costa Rica | 0.75 (-0.03,1.55) | 1.31 (-0.05,2.66) | 16.86 (-1.43,35.01) | 30.22 (-1.7,60.46) | 0.2 (0.04,0.39) | 0.46 (0.11,0.83) | 6.04 (1.32,11.58) | 14.29 (3.53,25.48) | 0.65 (0.45,0.9) | 1.1 (0.75,1.45) | 17.51 (11.92,24.11) | 30.16 (20.71,39.65) |
| Croatia | 1.64 (-0.06,3.26) | 1.81 (-0.06,3.64) | 34.82 (-1.82,67.61) | 39.57 (-1.63,77.88) | 0.71 (0.16,1.34) | 0.9 (0.23,1.61) | 19.49 (4.39,35.96) | 24.1 (5.94,43.4) | 1.2 (0.81,1.64) | 1.41 (0.97,1.93) | 31.39 (21.51,42.89) | 38.45 (26.9,51.68) |
| Cuba | 0.96 (-0.03,1.89) | 1.4 (-0.05,2.85) | 21.27 (-1.67,43.4) | 31.52 (-1.81,64.78) | 0.19 (0.04,0.37) | 0.47 (0.11,0.87) | 6.04 (1.3,11.51) | 14.64 (3.53,27.15) | 1.3 (0.9,1.76) | 2.41 (1.66,3.33) | 38.16 (26.13,51.66) | 66.3 (45.68,91.28) |
| Cyprus | 1.25 (-0.03,2.62) | 1.65 (-0.05,3.37) | 27.06 (-1,54.93) | 37.15 (-1.29,77.5) | 0.37 (0.06,0.78) | 0.56 (0.12,1.09) | 9.58 (1.71,19.8) | 14.18 (3.16,27.6) | 0.92 (0.54,1.57) | 0.95 (0.59,1.41) | 20.78 (12.73,33.5) | 22.76 (14,34.34) |
| Czech Republic | 2 (-0.06,3.94) | 1.58 (-0.06,3.25) | 44 (-1.74,86.77) | 33.64 (-1.45,68.7) | 0.8 (0.18,1.51) | 0.86 (0.21,1.63) | 22.91 (5.14,43.03) | 22.69 (5.47,42.86) | 2.04 (1.38,2.78) | 1.53 (1.01,2.17) | 52.14 (35.48,70.3) | 39.14 (25.73,55.78) |
| Democratic Republic of the Congo | 0.32 (-0.01,0.71) | 0.78 (-0.02,1.71) | 7.06 (-0.58,15.9) | 17.67 (-0.86,40.93) | 0.04 (0,0.09) | 0.17 (0.03,0.36) | 1.05 (-0.09,2.63) | 4.93 (0.81,10.45) | 0.24 (0.14,0.38) | 0.5 (0.27,0.86) | 6.45 (3.73,10.35) | 13.25 (7.27,22.9) |
| Denmark | 2.16 (-0.07,4.21) | 1.45 (-0.04,2.95) | 48.98 (-2.33,94.21) | 29.97 (-0.96,59.46) | 0.53 (0.1,1) | 0.59 (0.13,1.08) | 14.96 (2.77,28.67) | 14.59 (3.12,25.87) | 1.01 (0.7,1.37) | 0.83 (0.56,1.11) | 26.17 (18.1,35.46) | 21.26 (14.74,28.61) |
| Djibouti | 0.31 (-0.01,0.68) | 0.59 (-0.02,1.29) | 5.99 (-0.87,13.57) | 11.66 (-1.73,25.66) | 0.03 (-0.02,0.11) | 0.12 (0,0.3) | 1.14 (-0.61,3.48) | 4 (0.14,9.59) | 0.22 (0.11,0.4) | 0.35 (0.19,0.63) | 5.96 (3.1,11.08) | 9.61 (5.19,16.75) |
| Dominica | 2.15 (-0.08,4.05) | 3.06 (-0.11,6.08) | 46.76 (-2.88,90.02) | 65.64 (-2.77,131.82) | 0.37 (0.08,0.71) | 0.54 (0.15,0.99) | 10.17 (2.22,19.47) | 15.61 (4.28,29.11) | 1.4 (0.92,2.21) | 2.1 (1.41,3.14) | 36.61 (24.28,56.59) | 54.61 (36.48,81.62) |
| Dominican Republic | 0.4 (-0.03,0.8) | 0.75 (-0.04,1.58) | 7.18 (-1.49,14.97) | 15.07 (-1.72,32.14) | 0.08 (0.01,0.18) | 0.23 (0.04,0.44) | 2.88 (0.44,6.11) | 7.46 (1.58,14.29) | 0.76 (0.48,1.15) | 1.41 (0.86,2.2) | 22.92 (14.42,34.68) | 42.22 (25.61,66.22) |
| Ecuador | 0.46 (-0.03,0.93) | 1.04 (-0.03,2.06) | 8.98 (-0.88,19.24) | 22.77 (-1.25,45.18) | 0.05 (0.01,0.09) | 0.58 (0.14,1.05) | 1.57 (0.32,2.94) | 17.81 (4.36,31.92) | 1.74 (1.2,2.36) | 1.38 (0.91,1.93) | 48.97 (33.49,65.95) | 37.91 (25.72,52.87) |
| Egypt | 0.53 (-0.03,1.01) | 2.38 (-0.08,4.64) | 10.36 (-1.44,21.32) | 52.12 (-2.2,102.42) | 0.15 (0.04,0.44) | 0.59 (0.18,1.04) | 4.46 (1.08,12.19) | 16.45 (4.98,28.88) | 0.5 (0.33,0.73) | 0.73 (0.49,1.03) | 13.41 (8.56,19.49) | 18.91 (12.6,27.17) |
| El Salvador | 0.45 (-0.02,0.87) | 0.94 (-0.04,1.89) | 9.92 (-1.12,19.5) | 21.6 (-2,44.07) | 0.23 (0.05,0.45) | 0.58 (0.15,1.09) | 7.2 (1.61,14.15) | 18.13 (4.79,34.11) | 0.79 (0.52,1.1) | 1 (0.66,1.43) | 21.89 (14.35,30.28) | 27.72 (18.31,39.97) |
| Equatorial Guinea | 0.5 (-0.01,1.18) | 1.49 (-0.04,3.32) | 11.53 (-0.89,29.58) | 33.25 (-2.23,74.38) | 0.09 (0.01,0.2) | 0.36 (0.07,0.73) | 2.89 (0.38,6.43) | 11.11 (2.13,22.48) | 0.42 (0.24,0.7) | 0.76 (0.39,1.26) | 12.24 (6.8,19.78) | 20.74 (10.44,34.72) |
| Eritrea | 0.24 (-0.01,0.56) | 0.51 (-0.02,1.16) | 5.27 (-0.56,13.15) | 10.93 (-1.61,25.59) | 0.04 (0,0.1) | 0.13 (0.01,0.29) | 1.3 (-0.1,3.23) | 4.06 (0.29,8.86) | 0.24 (0.12,0.37) | 0.39 (0.22,0.61) | 6.7 (3.48,10.78) | 10.65 (5.95,17.06) |
| Estonia | 1.49 (-0.05,3) | 1.44 (-0.05,2.95) | 35.49 (-1.61,69.81) | 32.72 (-1.15,65.18) | 0.83 (0.19,1.54) | 0.79 (0.18,1.42) | 24.47 (5.71,45.23) | 21.08 (4.91,37.97) | 1.41 (0.97,1.9) | 1.22 (0.82,1.7) | 38.74 (26.4,52.4) | 33.92 (22.55,46.68) |
| Ethiopia | 0.31 (-0.02,0.68) | 0.42 (-0.02,0.88) | 5.62 (-1.25,13.83) | 7.6 (-1.01,15.98) | 0.05 (-0.01,0.14) | 0.1 (0,0.21) | 1.61 (-0.31,4.96) | 3.06 (0.05,6.42) | 0.21 (0.12,0.33) | 0.18 (0.1,0.29) | 5.99 (3.4,9.41) | 4.67 (2.71,7.95) |
| Fiji | 2.52 (-0.07,5.14) | 4.13 (-0.16,8.29) | 66.86 (-1.85,138.55) | 104.03 (-3.88,211.85) | 0.24 (0.06,0.48) | 0.4 (0.12,0.71) | 7.63 (1.82,15.4) | 12.15 (3.65,22.12) | 1.46 (0.87,2.31) | 2.24 (1.38,3.33) | 43.27 (26.08,67.75) | 64.29 (39.58,97.21) |
| Finland | 1.31 (-0.04,2.63) | 1.27 (-0.04,2.63) | 29.28 (-1.67,59.55) | 30 (-0.97,61.09) | 0.64 (0.12,1.21) | 0.57 (0.13,1.04) | 17.05 (3.29,32.44) | 14.44 (3.33,26.55) | 1.05 (0.73,1.42) | 1.16 (0.8,1.57) | 25.79 (17.85,35.04) | 28.12 (19.86,37.92) |
| France | 1.36 (-0.04,2.74) | 1.35 (-0.04,2.79) | 29.51 (-1.71,58.18) | 30.7 (-1.21,61.02) | 0.42 (0.07,0.77) | 0.5 (0.12,0.91) | 10.93 (1.82,19.79) | 12.3 (2.83,22.26) | 0.75 (0.52,1.01) | 0.87 (0.59,1.17) | 18.13 (12.67,24.52) | 21.74 (15.29,29.13) |
| Gabon | 1.19 (-0.04,2.7) | 2.23 (-0.06,4.72) | 27.12 (-1.99,64.16) | 49.65 (-2.88,105.04) | 0.25 (0.05,0.48) | 0.57 (0.14,1.13) | 7.59 (1.42,14.58) | 17.1 (4.4,33.65) | 0.7 (0.4,1.13) | 1.05 (0.58,1.58) | 19.37 (11.12,31.07) | 28.11 (15.63,43.12) |
| Gambia | 0.31 (-0.01,0.62) | 0.59 (-0.02,1.28) | 7.49 (-0.34,15.34) | 13.85 (-0.94,30.32) | 0.08 (0.01,0.16) | 0.18 (0.04,0.38) | 2.56 (0.46,5) | 5.57 (1.23,11.92) | 0.37 (0.22,0.57) | 0.61 (0.36,0.94) | 10.23 (5.98,15.64) | 16.5 (9.76,25.41) |
| Georgia | 1.48 (-0.06,2.9) | 2.27 (-0.08,4.66) | 33.57 (-3.26,66.79) | 54.31 (-2.28,111.6) | 0.19 (0.05,0.38) | 1.03 (0.24,1.97) | 6.09 (1.47,11.96) | 30.01 (6.89,57.51) | 2.2 (1.51,2.92) | 2.36 (1.59,3.27) | 64.56 (44.9,85.26) | 65.07 (44,90.82) |
| Germany | 1.83 (-0.06,3.74) | 1.59 (-0.05,3.25) | 40.26 (-2.43,81.76) | 35.16 (-1.23,70.94) | 0.84 (0.17,1.58) | 0.61 (0.14,1.14) | 22.47 (4.53,42.25) | 15.57 (3.61,28.99) | 0.99 (0.67,1.34) | 0.78 (0.53,1.06) | 24.35 (17.02,32.97) | 19.78 (13.74,26.77) |
| Ghana | 0.43 (-0.03,0.88) | 1.01 (-0.05,2.22) | 8.05 (-1.51,18.87) | 20.89 (-2.02,46.57) | 0.05 (0,0.1) | 0.22 (0.05,0.45) | 1.66 (0.19,3.44) | 6.96 (1.59,14.12) | 0.47 (0.26,0.76) | 1.21 (0.7,1.9) | 12.55 (7.21,20.67) | 29.91 (17.64,48.19) |
| Greece | 1.53 (-0.05,3.14) | 1.87 (-0.06,3.79) | 34.6 (-1.94,70.74) | 40.48 (-1.46,80.18) | 0.46 (0.09,0.9) | 0.7 (0.17,1.29) | 12.78 (2.52,24.79) | 18.63 (4.53,34.01) | 0.72 (0.5,0.97) | 1.11 (0.76,1.48) | 18.09 (12.65,24.34) | 29.26 (20.46,38.66) |
| Greenland | 2.31 (-0.07,4.75) | 1.34 (-0.04,2.76) | 52.49 (-2.42,108.66) | 31.6 (-1.18,66.28) | 1.31 (0.32,2.47) | 0.92 (0.23,1.7) | 37.54 (9.06,70.32) | 28.79 (7.15,53.83) | 0.63 (0.39,0.94) | 0.42 (0.26,0.65) | 16.2 (9.85,23.48) | 11.05 (6.7,17.06) |
| Grenada | 1.4 (-0.06,2.81) | 2.51 (-0.09,5.01) | 32.63 (-2.46,65.93) | 58.39 (-3.93,116.52) | 0.47 (0.09,1.01) | 1.05 (0.26,1.97) | 15.9 (2.94,34.29) | 32.98 (8.57,60.81) | 1.26 (0.86,1.71) | 2.07 (1.39,2.87) | 38.23 (26.06,52.15) | 58.59 (39.62,81.33) |
| Grenadines | 1.14 (-0.07,2.2) | 1.77 (-0.07,3.6) | 21.06 (-2.96,43.76) | 36.65 (-3.3,75.2) | 0.28 (0.04,0.57) | 0.59 (0.14,1.12) | 9.04 (1.44,18.37) | 19.45 (4.68,36.54) | 0.97 (0.66,1.39) | 1.52 (1.03,2.18) | 27.69 (18.8,39.22) | 43.92 (29.52,63.04) |
| Guam | 1.52 (-0.04,3.25) | 1.14 (-0.03,2.43) | 34.6 (-0.93,72.33) | 33.56 (-0.92,71.23) | 0.24 (0.05,0.49) | 0.4 (0.1,0.74) | 7.01 (1.5,14.73) | 14.18 (3.66,25.9) | 1.05 (0.68,1.51) | 0.94 (0.61,1.32) | 29.38 (19.14,42.26) | 30.95 (20.27,42.91) |
| Guatemala | 0.36 (-0.01,0.72) | 0.68 (-0.03,1.37) | 6.57 (-0.66,13) | 14.19 (-1.31,29.02) | 0.11 (0.03,0.2) | 0.31 (0.08,0.57) | 3.34 (0.8,6.21) | 9.62 (2.51,17.82) | 1.18 (0.82,1.6) | 1.2 (0.84,1.62) | 31.23 (21.84,42) | 33 (23.22,44.76) |
| Guinea | 0.48 (-0.02,0.99) | 0.81 (-0.02,1.67) | 11.49 (-0.65,23.76) | 19.14 (-0.88,39.83) | 0.07 (0.01,0.14) | 0.14 (0.03,0.28) | 2.19 (0.37,4.36) | 4.26 (0.9,8.74) | 0.37 (0.23,0.59) | 0.53 (0.33,0.85) | 10.27 (6.22,16.28) | 14.39 (8.63,23.01) |
| Guinea-Bissau | 0.48 (-0.02,1.12) | 0.85 (-0.04,1.97) | 10.09 (-1.23,25.34) | 18.16 (-1.76,43.86) | 0.07 (0.01,0.15) | 0.16 (0.03,0.32) | 2.14 (0.3,4.78) | 5.01 (0.87,10.28) | 0.49 (0.3,0.77) | 0.77 (0.46,1.2) | 13.49 (8.14,20.97) | 20.56 (12.31,32.31) |
| Guyana | 1.02 (-0.04,2.07) | 1.59 (-0.06,3.27) | 22.02 (-1.61,46.8) | 34.83 (-3.67,71.58) | 0.35 (0.06,0.69) | 0.8 (0.19,1.49) | 11.5 (2.06,22.39) | 25.72 (6.16,47.45) | 1.24 (0.82,1.78) | 2.26 (1.48,3.24) | 35.47 (23.8,51.24) | 65.22 (42.08,94.47) |
| Haiti | 0.37 (-0.03,0.94) | 0.74 (-0.05,1.77) | 6.29 (-2.65,18.54) | 14.08 (-2.34,34.22) | 0.07 (-0.01,0.22) | 0.23 (0.03,0.54) | 2.53 (-0.09,7.72) | 7.86 (0.92,17.77) | 0.68 (0.38,1.09) | 1.14 (0.61,1.97) | 20.96 (11.37,33.35) | 34.15 (18.37,58.71) |
| Honduras | 0.42 (-0.02,0.89) | 1.06 (-0.05,2.15) | 9.41 (-0.56,20.75) | 25.69 (-1.32,51.76) | 0.28 (0.05,0.56) | 0.68 (0.17,1.38) | 8.73 (1.56,17.55) | 21.07 (5.34,43.32) | 1.33 (0.8,2.1) | 2.69 (1.4,4.18) | 37.05 (22.46,58.18) | 72.13 (38.13,112.49) |
| Hungary | 1.87 (-0.06,3.65) | 1.92 (-0.06,3.87) | 41.5 (-2.29,80.44) | 44.66 (-1.61,88.12) | 0.88 (0.21,1.55) | 0.92 (0.25,1.66) | 25.54 (5.92,45.27) | 25.94 (7.06,46.62) | 1.86 (1.25,2.46) | 1.57 (1.06,2.16) | 48.04 (32.56,63.27) | 43.03 (28.98,58.88) |
| Iceland | 1.74 (-0.06,3.53) | 1.39 (-0.04,2.95) | 39.8 (-1.87,78.24) | 30.12 (-1.59,62.3) | 0.82 (0.17,1.57) | 0.65 (0.15,1.23) | 22.45 (4.78,42.45) | 16.69 (3.98,31.22) | 0.82 (0.57,1.13) | 0.89 (0.61,1.22) | 21.74 (15.17,29.65) | 22.42 (15.43,30.53) |
| India | 0.1 (-0.01,0.21) | 0.35 (-0.02,0.72) | 1.7 (-0.63,4.25) | 7.47 (-0.96,16.11) | 0.03 (-0.01,0.06) | 0.14 (0.02,0.27) | 0.85 (-0.14,1.98) | 4.34 (0.73,8.35) | 0.08 (0.05,0.11) | 0.17 (0.12,0.25) | 2.13 (1.3,3.1) | 4.74 (3.06,6.84) |
| Indonesia | 0.27 (-0.01,0.65) | 0.76 (-0.02,1.71) | 7.86 (-0.16,18.72) | 21.89 (-0.52,48.8) | 0.02 (-0.01,0.07) | 0.16 (0.02,0.35) | 0.95 (-0.36,2.74) | 5.86 (0.88,12.53) | 0.2 (0.12,0.3) | 0.48 (0.25,0.74) | 6.34 (3.83,9.47) | 15.27 (7.78,23.29) |
| Iran | 0.28 (-0.02,0.58) | 0.77 (-0.04,1.49) | 5.52 (-0.93,12.44) | 17.34 (-1.61,33.51) | 0.11 (0.02,0.23) | 0.35 (0.1,0.62) | 3.47 (0.67,6.99) | 10.21 (3.08,18.15) | 0.2 (0.12,0.29) | 0.35 (0.17,0.5) | 5.81 (3.39,8.36) | 10.04 (4.74,14.43) |
| Iraq | 0.67 (-0.03,1.4) | 1.22 (-0.05,2.69) | 15.42 (-1.5,33.33) | 27.64 (-3.03,61.08) | 0.27 (0.06,0.6) | 0.47 (0.12,0.89) | 8.46 (1.8,18.47) | 14.22 (3.65,27.48) | 0.4 (0.21,0.76) | 0.57 (0.36,0.88) | 11.49 (6.03,22.71) | 16.18 (10.02,25.09) |
| Ireland | 1.97 (-0.06,4.03) | 1.37 (-0.04,2.85) | 44.65 (-2.63,90.94) | 30.92 (-1.31,62.42) | 0.8 (0.17,1.53) | 0.72 (0.17,1.32) | 22.84 (4.79,43.51) | 18.49 (4.33,33.7) | 0.86 (0.59,1.17) | 0.91 (0.62,1.25) | 21.83 (15.34,29.94) | 23.38 (16.04,31.83) |
| Israel | 2 (-0.07,4.1) | 1.57 (-0.05,3.19) | 42.49 (-2.9,85.34) | 33.61 (-1.22,67.31) | 0.72 (0.15,1.36) | 0.58 (0.14,1.04) | 19.33 (4.21,36.73) | 14.5 (3.38,26.09) | 0.84 (0.56,1.14) | 0.94 (0.65,1.27) | 20.37 (13.62,28.01) | 22.14 (15.03,30.17) |
| Italy | 1.5 (-0.05,3.04) | 1.35 (-0.04,2.77) | 33.71 (-2.16,67.61) | 29.67 (-1.24,59.97) | 0.39 (0.08,0.74) | 0.49 (0.11,0.92) | 10.57 (1.98,20.37) | 12.26 (2.7,23.33) | 0.28 (0.2,0.37) | 0.77 (0.53,1.05) | 7.5 (5.23,10.03) | 21.43 (15.01,28.79) |
| Ivory Coast | 0.74 (-0.03,1.59) | 1.27 (-0.03,2.68) | 16.34 (-1.31,34.75) | 27.81 (-2.08,59.13) | 0.15 (0.03,0.29) | 0.31 (0.06,0.61) | 4.58 (0.88,8.98) | 9.22 (1.78,18.67) | 0.29 (0.18,0.44) | 0.44 (0.26,0.66) | 7.72 (4.94,11.26) | 11.37 (6.49,17.63) |
| Jamaica | 1.28 (-0.05,2.57) | 2.25 (-0.09,4.49) | 29.51 (-2.02,57.57) | 50.15 (-4.26,99.18) | 0.42 (0.09,0.77) | 0.79 (0.22,1.43) | 13.34 (2.89,23.96) | 24.48 (6.95,44) | 1.04 (0.72,1.42) | 2.71 (1.75,3.77) | 29.26 (20.21,39.54) | 74.99 (48.67,105.41) |
| Japan | 0.26 (-0.01,0.52) | 0.47 (-0.01,0.96) | 7.98 (-0.22,16.02) | 13.85 (-0.37,28.27) | 0.09 (-0.01,0.19) | 0.11 (0.01,0.23) | 2.65 (-0.24,5.97) | 3.38 (0.27,6.98) | 0.26 (0.2,0.35) | 0.37 (0.26,0.49) | 6.71 (5.03,8.94) | 10.78 (7.75,14.28) |
| Jordan | 1.15 (-0.04,2.34) | 1.91 (-0.07,3.7) | 27.48 (-1.32,55.53) | 45.15 (-2.09,87.68) | 0.36 (0.09,0.71) | 0.67 (0.2,1.22) | 10.66 (2.65,20.65) | 18.53 (5.48,33.87) | 0.78 (0.48,1.18) | 0.78 (0.48,1.18) | 21.22 (12.92,32.23) | 20.86 (12.69,31.61) |
| Kazakhstan | 1.41 (-0.05,2.78) | 1.16 (-0.04,2.28) | 33.01 (-2.7,64.11) | 27.8 (-1.45,53.73) | 0.48 (0.11,0.88) | 0.7 (0.18,1.25) | 14.71 (3.26,26.73) | 21.26 (5.3,38.01) | 1.58 (1.09,2.12) | 1.22 (0.85,1.62) | 43.92 (30.4,58.96) | 34.64 (24.23,45.42) |
| Kenya | 0.32 (-0.01,0.68) | 0.85 (-0.04,1.8) | 6.89 (-0.54,15.72) | 18.41 (-1.71,41.24) | 0.08 (0.01,0.17) | 0.31 (0.07,0.63) | 2.65 (0.27,5.73) | 10.11 (2.32,20.55) | 0.16 (0.09,0.3) | 0.36 (0.21,0.6) | 4.47 (2.57,8.53) | 10 (5.74,16.91) |
| Kiribati | 1.59 (-0.05,3.33) | 2.91 (-0.08,6.15) | 43.63 (-1.4,91.47) | 76.41 (-2.11,161.37) | 0.05 (0.01,0.1) | 0.12 (0.03,0.24) | 1.8 (0.4,3.52) | 4.04 (1.11,8) | 1.38 (0.53,2.25) | 2.02 (0.7,3.42) | 43.04 (16.44,69.18) | 60.29 (20.99,102.78) |
| Kuwait | 1.08 (-0.04,2.06) | 1.26 (-0.04,2.53) | 26.58 (-1.44,51.13) | 30.44 (-1.33,60.01) | 0.52 (0.14,0.93) | 0.55 (0.17,0.94) | 15.04 (4.02,26.71) | 15.58 (4.86,26.41) | 0.59 (0.42,0.78) | 1.3 (0.94,1.68) | 16.47 (11.63,21.59) | 37.96 (28.09,48.64) |
| Kyrgyzstan | 1.08 (-0.04,2.12) | 0.94 (-0.04,1.87) | 24.5 (-2.21,48.67) | 22.23 (-1.55,44.25) | 0.33 (0.07,0.63) | 0.79 (0.19,1.43) | 9.68 (2.18,18.3) | 23.92 (5.78,43.49) | 1.22 (0.82,1.67) | 1.16 (0.73,1.59) | 36.32 (24.85,49.06) | 33.83 (21.46,46.62) |
| Laos | 0.3 (-0.01,0.73) | 0.65 (-0.01,1.37) | 8.52 (-0.16,21.41) | 18.33 (-0.34,39.44) | 0.03 (-0.01,0.1) | 0.17 (0.02,0.37) | 1.04 (-0.21,3.39) | 5.82 (0.63,12.61) | 0.29 (0.15,0.52) | 0.45 (0.24,0.74) | 8.94 (4.25,15.98) | 14.05 (7.5,22.86) |
| Latvia | 1.48 (-0.05,2.94) | 1.8 (-0.06,3.62) | 35.39 (-1.86,69.29) | 41.87 (-1.53,82.17) | 0.76 (0.18,1.4) | 1.18 (0.29,2.2) | 22.86 (5.43,41.65) | 33.93 (8.25,64.75) | 1.47 (1.05,1.97) | 2.04 (1.41,2.79) | 41.25 (29.59,55.43) | 55.12 (37.79,75.27) |
| Lebanon | 1.41 (-0.04,3.02) | 2 (-0.07,3.83) | 31.27 (-1.82,68.7) | 45.61 (-2.09,85.8) | 0.4 (0.08,0.89) | 0.71 (0.17,1.27) | 11.26 (2.33,24.63) | 18.81 (4.7,33.92) | 0.66 (0.4,1.04) | 0.64 (0.41,0.97) | 17.34 (9.95,28.28) | 16.47 (10.51,25.27) |
| Lesotho | 0.85 (-0.03,1.92) | 2.22 (-0.07,5.06) | 19.17 (-0.77,44.01) | 50.05 (-2.92,115.55) | 0.31 (0.07,0.67) | 0.79 (0.18,1.68) | 8.82 (1.93,19.09) | 22.49 (5.13,47.89) | 0.69 (0.42,1.09) | 1.75 (0.95,2.85) | 17.15 (10.34,27.67) | 44.03 (23.4,72.24) |
| Liberia | 0.67 (-0.02,1.43) | 1.15 (-0.05,2.46) | 16.54 (-0.7,34.78) | 26.81 (-1.44,57.13) | 0.11 (0.02,0.21) | 0.25 (0.06,0.5) | 3.32 (0.58,6.58) | 7.65 (1.74,15.24) | 0.59 (0.38,0.88) | 0.92 (0.51,1.45) | 16.03 (10.25,23.9) | 24.1 (13.35,38.38) |
| Libya | 0.57 (-0.02,1.12) | 1.35 (-0.04,2.67) | 13.11 (-0.88,26.27) | 31.74 (-2.24,63.33) | 0.39 (0.08,0.8) | 1.06 (0.28,1.96) | 11.66 (2.36,24.21) | 30.83 (8.32,57.05) | 0.57 (0.33,0.88) | 1.03 (0.63,1.54) | 15.99 (9.49,24.39) | 28.72 (17.75,43.68) |
| Lithuania | 1.19 (-0.04,2.35) | 1.56 (-0.05,3.15) | 27.37 (-1.93,54.18) | 35.8 (-1.37,72.38) | 0.72 (0.17,1.36) | 0.99 (0.24,1.83) | 20.58 (4.73,39.44) | 27.08 (6.53,50.53) | 1.28 (0.89,1.73) | 1.79 (1.25,2.45) | 35.01 (24.35,47.42) | 48.34 (33.78,66.77) |
| Luxembourg | 1.96 (-0.06,3.88) | 1.44 (-0.04,2.98) | 43.76 (-1.79,86.97) | 31.11 (-1.08,63.41) | 0.84 (0.17,1.57) | 0.69 (0.17,1.28) | 22.07 (4.49,41.58) | 16.8 (4.23,31.29) | 1.41 (0.96,1.92) | 1.2 (0.82,1.64) | 34.79 (23.71,47.07) | 29.17 (20.16,39.6) |
| Madagascar | 0.32 (-0.01,0.67) | 0.58 (-0.03,1.27) | 6.6 (-0.95,14.53) | 12.03 (-1.14,27.99) | 0.07 (0,0.15) | 0.19 (0.03,0.42) | 1.87 (-0.05,4.34) | 5.53 (0.89,12.31) | 0.27 (0.14,0.43) | 0.45 (0.24,0.74) | 7.16 (3.63,11.57) | 11.76 (6.29,19.5) |
| Malawi | 0.19 (-0.01,0.4) | 0.48 (-0.02,1.02) | 3.3 (-1.12,7.97) | 9.12 (-1.62,20.27) | 0.02 (-0.02,0.06) | 0.11 (0.01,0.28) | 0.68 (-0.41,2.07) | 4.2 (0.45,9.71) | 0.13 (0.08,0.2) | 0.23 (0.13,0.37) | 3.67 (2.27,5.66) | 6.85 (3.75,11.2) |
| Malaysia | 0.93 (-0.02,1.96) | 1.97 (-0.05,3.92) | 27.44 (-0.66,57.82) | 56.26 (-1.49,110.73) | 0.14 (0.02,0.29) | 0.38 (0.09,0.72) | 4.69 (0.85,9.63) | 11.96 (2.89,23.29) | 0.48 (0.31,0.68) | 0.8 (0.52,1.14) | 14 (9.17,19.76) | 22.47 (14.55,31.46) |
| Maldives | 0.36 (-0.01,0.98) | 0.55 (-0.02,1.15) | 10.92 (-0.18,30.37) | 16.19 (-0.51,33.72) | 0.1 (0.01,0.33) | 0.31 (0.07,0.63) | 3.79 (0.32,12.49) | 10.94 (2.48,22.79) | 0.19 (0.09,0.32) | 0.15 (0.09,0.23) | 6.21 (2.74,10.48) | 4.79 (2.98,7.36) |
| Mali | 0.51 (-0.02,1.05) | 0.71 (-0.03,1.57) | 11.94 (-0.81,25.42) | 16.32 (-1.23,36.5) | 0.03 (0,0.07) | 0.06 (0.01,0.13) | 1.03 (0.12,2.08) | 1.89 (0.32,3.89) | 0.3 (0.18,0.44) | 0.35 (0.21,0.55) | 8.16 (4.88,12.39) | 9.72 (5.76,14.59) |
| Malta | 1.97 (-0.06,3.89) | 1.55 (-0.06,3.2) | 43.9 (-2.22,86.55) | 34.11 (-1.89,68.83) | 0.56 (0.1,1.05) | 0.75 (0.18,1.4) | 15.09 (2.7,28.56) | 20.05 (4.95,37.32) | 0.89 (0.6,1.22) | 1.18 (0.82,1.64) | 22.13 (15.19,30.5) | 30.15 (21.08,41.36) |
| Marshall Islands | 1.7 (-0.05,3.73) | 2.98 (-0.06,6.88) | 44.76 (-1.23,100.68) | 78.01 (-1.65,182.38) | 0.22 (0.05,0.47) | 0.5 (0.11,1.07) | 7.14 (1.81,14.85) | 15.97 (3.58,35.65) | 1.69 (0.9,2.72) | 2.71 (1.11,5.85) | 50.18 (27,81.64) | 81.57 (33.3,174.74) |
| Mauritania | 0.87 (-0.03,1.96) | 1.37 (-0.05,2.88) | 20.85 (-0.84,46.56) | 30.34 (-1.75,62.71) | 0.13 (0.03,0.24) | 0.27 (0.06,0.52) | 3.77 (0.78,7.29) | 7.85 (1.81,15.57) | 0.78 (0.45,1.21) | 1.05 (0.58,1.63) | 20.23 (11.49,31.47) | 25.71 (14.49,39.84) |
| Mauritius | 0.58 (-0.02,1.19) | 1.76 (-0.05,3.61) | 16.13 (-0.42,32.9) | 48.76 (-1.54,100.03) | 0.18 (0.03,0.35) | 0.57 (0.13,1.05) | 6.01 (1.04,11.71) | 19.03 (4.4,35.59) | 1.16 (0.79,1.56) | 1.46 (1.01,1.97) | 34.42 (23.47,46.78) | 44.26 (31.04,59.87) |
| Mexico | 0.72 (-0.03,1.43) | 1.12 (-0.04,2.21) | 14.2 (-1.32,28.83) | 25.59 (-1.98,51.24) | 0.4 (0.09,0.73) | 0.77 (0.22,1.41) | 11.95 (2.7,21.86) | 24.53 (6.95,44.33) | 0.55 (0.38,0.75) | 0.79 (0.53,1.07) | 14.04 (9.79,18.68) | 22.87 (15.46,31.16) |
| Micronesia | 2.05 (-0.06,4.55) | 3.32 (-0.09,7.05) | 56.9 (-1.68,123.61) | 90.33 (-2.25,193.83) | 0.22 (0.05,0.44) | 0.5 (0.11,1.05) | 7.57 (1.78,15.23) | 16.53 (3.87,34.73) | 1.67 (0.9,2.67) | 2.31 (1.19,3.79) | 51.23 (27.47,83.5) | 70.27 (35.81,115.75) |
| Moldova | 1.48 (-0.05,2.96) | 1.85 (-0.05,3.64) | 35.86 (-2.4,70.81) | 46.34 (-1.48,91.75) | 0.66 (0.16,1.2) | 0.63 (0.16,1.09) | 20.27 (4.84,36.59) | 18.87 (4.87,32.61) | 1.35 (0.96,1.77) | 1.48 (1.05,1.89) | 38.44 (27.3,50.46) | 43.09 (30.53,55.59) |
| Monaco | 2.19 (-0.08,4.71) | 2.79 (-0.09,5.83) | 48.4 (-2.41,104.31) | 60.95 (-2.9,116.9) | 0.61 (0.11,1.25) | 0.67 (0.15,1.45) | 17.17 (3.18,35.59) | 17.88 (4.02,38.56) | 0.43 (0.25,0.69) | 0.57 (0.34,0.87) | 10.98 (6.33,17.39) | 14.73 (8.84,22.12) |
| Mongolia | 0.27 (-0.01,0.58) | 0.34 (-0.01,0.69) | 6.07 (-0.52,13.08) | 7.22 (-0.52,14.99) | 0.26 (0.04,0.55) | 0.42 (0.08,0.83) | 8.48 (1.32,17.89) | 12.83 (2.61,25.88) | 0.68 (0.38,1.08) | 0.66 (0.4,1.04) | 20.4 (11.55,32.95) | 19.21 (11.24,29.69) |
| Montenegro | 1.53 (-0.05,3.19) | 2.62 (-0.09,5.35) | 35.05 (-1.66,72.11) | 59.07 (-2.25,121.83) | 0.49 (0.11,0.96) | 0.77 (0.2,1.42) | 14.46 (3.27,28.03) | 20.64 (5.39,38.29) | 0.97 (0.57,1.55) | 1.4 (0.88,2.1) | 26.47 (15.71,42.33) | 35.39 (22.16,52.87) |
| Morocco | 0.29 (-0.02,0.59) | 0.64 (-0.04,1.41) | 6.23 (-0.8,13.41) | 14.2 (-1.58,30.54) | 0.17 (0.03,0.34) | 0.4 (0.1,0.77) | 5.03 (0.93,10.3) | 12.01 (2.99,23.03) | 0.16 (0.09,0.25) | 0.26 (0.15,0.4) | 4.37 (2.5,6.99) | 7.39 (4.27,11.29) |
| Mozambique | 0.29 (-0.01,0.61) | 0.66 (-0.02,1.45) | 4.86 (-0.85,11.12) | 12.25 (-1.27,27.4) | 0.07 (0,0.17) | 0.26 (0.04,0.58) | 2.26 (-0.07,5.3) | 8.52 (1.39,18.55) | 0.29 (0.14,0.5) | 0.55 (0.27,1.02) | 8.21 (3.65,14.48) | 15.29 (7.47,28.64) |
| Myanmar | 0.41 (-0.01,0.91) | 0.65 (-0.02,1.4) | 12.52 (-0.29,27.88) | 19.2 (-0.49,41.66) | 0.06 (0,0.16) | 0.16 (0.02,0.34) | 2.38 (0.05,5.77) | 5.7 (0.76,11.91) | 0.36 (0.19,0.6) | 0.43 (0.26,0.67) | 11.87 (5.95,20.26) | 13.71 (7.79,21.86) |
| Namibia | 0.69 (-0.03,1.46) | 1.98 (-0.05,4.15) | 15.13 (-1.14,32.64) | 44.46 (-2.49,93.96) | 0.15 (0.03,0.29) | 0.38 (0.09,0.77) | 4.45 (0.9,8.76) | 10.98 (2.44,22.06) | 0.44 (0.27,0.72) | 0.78 (0.43,1.25) | 11.49 (7.04,18.29) | 19.65 (10.33,31.26) |
| Nauru | 2.86 (-0.06,6.89) | 4.08 (-0.09,9.53) | 78.58 (-1.62,189.19) | 110.24 (-2.45,261.3) | 0.29 (0.07,0.66) | 0.55 (0.12,1.19) | 10 (2.46,21.93) | 18.25 (4.11,40.3) | 2.21 (0.99,4.01) | 2.86 (1.33,4.82) | 67.29 (29.2,120.42) | 86.51 (39.4,149.28) |
| Nepal | 0.07 (-0.01,0.16) | 0.14 (-0.02,0.32) | 1.28 (-0.78,3.59) | 2.36 (-1.07,6.69) | 0.02 (-0.01,0.06) | 0.08 (0,0.2) | 0.64 (-0.29,1.96) | 3.02 (0.19,7.15) | 0.07 (0.04,0.14) | 0.11 (0.06,0.24) | 2.14 (1.03,4.36) | 3.35 (1.66,7.44) |
| Netherlands | 2.03 (-0.06,4.15) | 1.57 (-0.05,3.18) | 44.19 (-2.28,87.73) | 34.4 (-1.22,68.67) | 0.67 (0.13,1.28) | 0.6 (0.13,1.11) | 17.96 (3.37,33.79) | 14.9 (3.25,27.37) | 0.77 (0.53,1.06) | 0.82 (0.56,1.1) | 18.58 (12.95,25.71) | 19.39 (13.65,26.29) |
| Nevis | 2.13 (-0.08,4.31) | 2.5 (-0.09,4.91) | 43.23 (-3.72,88.67) | 55.9 (-2.34,110.99) | 0.51 (0.1,1.07) | 0.8 (0.21,1.45) | 15.96 (3.21,34.22) | 22.27 (6.25,40.27) | 1.81 (1.21,2.46) | 2.48 (1.67,3.32) | 51.57 (35.17,69.65) | 62.53 (42.64,83.82) |
| New Zealand | 2.02 (-0.06,4.06) | 1.38 (-0.05,2.74) | 47.77 (-2.52,95.34) | 32.18 (-2.06,63.14) | 0.73 (0.16,1.34) | 0.51 (0.12,0.92) | 20.75 (4.7,37.82) | 13.57 (3.25,24.14) | 1.07 (0.74,1.43) | 1.24 (0.88,1.65) | 27.79 (19.29,37.31) | 32.62 (23.17,43.02) |
| Nicaragua | 0.36 (-0.02,0.71) | 0.77 (-0.03,1.57) | 7.46 (-0.67,15.49) | 18.19 (-1.14,36.18) | 0.16 (0.03,0.32) | 0.37 (0.1,0.7) | 4.86 (1.1,10.02) | 11.25 (3.07,21.36) | 0.39 (0.26,0.57) | 0.53 (0.33,0.77) | 10.89 (7.22,15.69) | 14.48 (9.17,21.07) |
| Niger | 0.3 (-0.01,0.65) | 0.4 (-0.01,0.85) | 6.88 (-0.42,15.87) | 8.99 (-0.52,19.41) | 0.04 (0.01,0.09) | 0.08 (0.01,0.17) | 1.41 (0.17,2.99) | 2.34 (0.4,5.35) | 0.34 (0.19,0.54) | 0.43 (0.24,0.69) | 9.16 (5.2,14.32) | 11.12 (6.17,18.17) |
| Nigeria | 0.73 (-0.03,1.51) | 1.92 (-0.06,3.85) | 15.06 (-0.94,31.96) | 41.95 (-1.7,86.8) | 0.07 (0.01,0.15) | 0.26 (0.05,0.5) | 2.03 (0.23,4.17) | 6.96 (1.42,13.67) | 0.19 (0.11,0.31) | 0.36 (0.22,0.58) | 4.6 (2.76,7.54) | 8.65 (5.13,14.37) |
| Niue | 2.03 (-0.07,4.24) | 3.3 (-0.11,6.85) | 53.15 (-1.79,109.75) | 83.24 (-2.86,168.95) | 0.22 (0.05,0.43) | 0.5 (0.14,0.97) | 7.3 (1.69,14.31) | 16.55 (4.73,31.87) | 1.33 (0.75,2.07) | 1.97 (0.92,3.5) | 39.35 (22.54,62.03) | 58.25 (27.16,105.63) |
| North Korea | 0.2 (0,0.46) | 0.41 (-0.01,0.92) | 5.27 (-0.11,12) | 10.47 (-0.24,24.27) | 0.01 (-0.02,0.03) | 0.07 (0,0.18) | 0.1 (-0.64,0.99) | 1.6 (-0.23,4.49) | 0.15 (0.09,0.24) | 0.29 (0.17,0.48) | 4.36 (2.44,6.92) | 8.02 (4.63,13.3) |
| North Macedonia | 1.43 (-0.05,2.88) | 2.44 (-0.08,4.97) | 31.42 (-2.39,64.09) | 52.61 (-1.91,109.17) | 0.58 (0.13,1.14) | 0.93 (0.23,1.74) | 16.79 (3.82,32.74) | 25.07 (6.38,47.79) | 1.52 (0.98,2.19) | 1.94 (1.24,2.88) | 39.38 (25.73,57.53) | 47.98 (30.53,72.44) |
| Northern Mariana Islands | 2.32 (-0.07,4.81) | 3.18 (-0.09,6.24) | 60.57 (-1.81,125.86) | 80.66 (-2.33,155.19) | 0.24 (0.05,0.5) | 0.62 (0.16,1.07) | 7.79 (1.8,16.89) | 19.71 (5.46,33.67) | 2.11 (1.17,3.96) | 3.29 (2.14,4.68) | 63.65 (35.07,117.69) | 97.32 (62.3,139.13) |
| Norway | 1.32 (-0.04,2.65) | 0.95 (-0.03,1.92) | 28.76 (-1.75,58.5) | 20.96 (-1.06,40.89) | 0.62 (0.11,1.17) | 0.55 (0.12,1.04) | 17.3 (3.31,32.75) | 13.71 (3.03,25.57) | 0.89 (0.61,1.2) | 0.72 (0.5,0.96) | 22.63 (15.66,30.56) | 17.42 (12.28,23.21) |
| Oman | 0.22 (-0.01,0.46) | 0.49 (-0.02,0.98) | 5.43 (-0.3,11.65) | 12.87 (-0.52,25.21) | 0.11 (0.02,0.24) | 0.29 (0.08,0.51) | 3.48 (0.57,7.61) | 8.44 (2.43,15.01) | 0.14 (0.08,0.21) | 0.2 (0.13,0.29) | 3.91 (2.18,6.29) | 5.68 (3.7,8.37) |
| Pakistan | 0.5 (-0.03,1.06) | 1.29 (-0.08,2.66) | 9.98 (-1.69,22.18) | 26.69 (-2.78,55.81) | 0.08 (-0.01,0.2) | 0.45 (0.09,0.94) | 2.8 (-0.17,6.49) | 13.97 (2.75,28.75) | 0.42 (0.28,0.67) | 1.06 (0.64,1.58) | 11.45 (7.68,17.88) | 28.65 (17.61,42.54) |
| Palau | 3.63 (-0.1,7.42) | 4.94 (-0.14,10.2) | 88.25 (-2.26,180.52) | 109.74 (-3.3,227.69) | 0.07 (0.01,0.13) | 0.11 (0.03,0.2) | 2.02 (0.46,3.86) | 3.24 (0.9,6.05) | 0.24 (0.14,0.38) | 0.3 (0.18,0.44) | 7 (4.05,11.02) | 8.09 (5.01,12.04) |
| Palestine | 1.34 (-0.04,2.81) | 2.33 (-0.09,4.43) | 29.6 (-2.52,63.97) | 53.96 (-2.55,101.72) | 0.32 (0.06,0.69) | 0.64 (0.18,1.18) | 9.25 (1.84,20.48) | 18.12 (5.05,33.49) | 1.44 (0.88,2.21) | 1.75 (1.07,2.49) | 39.26 (23.91,60.32) | 47.24 (29.25,67.12) |
| Panama | 0.82 (-0.03,1.68) | 1.29 (-0.04,2.64) | 17.58 (-0.92,35.12) | 29.63 (-1.3,58.81) | 0.18 (0.04,0.33) | 0.53 (0.16,0.93) | 4.68 (1.01,8.71) | 15.35 (4.4,27.19) | 0.73 (0.5,1.01) | 1.33 (0.92,1.82) | 18.09 (12.39,24.88) | 35.03 (24,48.07) |
| Papua New Guinea | 0.7 (-0.02,1.61) | 0.99 (-0.03,2.1) | 21.3 (-0.56,48.58) | 29.77 (-0.84,62.98) | 0.05 (0,0.12) | 0.1 (0.02,0.23) | 1.69 (0.18,4.16) | 3.65 (0.65,8.05) | 0.58 (0.29,1.01) | 0.82 (0.38,1.45) | 17.8 (8.3,31.6) | 25.34 (11.5,45.83) |
| Paraguay | 0.71 (-0.03,1.47) | 1.42 (-0.06,3.09) | 14.89 (-1.55,31.29) | 31.62 (-1.96,68.4) | 0.16 (0.03,0.35) | 0.38 (0.08,0.75) | 4.91 (0.94,10.42) | 11.25 (2.55,21.94) | 1.03 (0.63,1.55) | 1.32 (0.79,2.04) | 27.14 (16.75,40.7) | 33.69 (20.41,53.86) |
| Peru | 0.59 (-0.02,1.2) | 0.83 (-0.03,1.77) | 12.42 (-1.42,26.43) | 18.91 (-1.5,41.5) | 0.22 (0.04,0.45) | 0.47 (0.11,0.93) | 7 (1.25,14.29) | 14.78 (3.48,29.47) | 0.92 (0.56,1.35) | 1.01 (0.62,1.63) | 26 (15.64,38.42) | 28.17 (17.19,45.35) |
| Philippines | 0.6 (-0.02,1.21) | 1.12 (-0.03,2.31) | 15.86 (-0.46,31.53) | 31.56 (-0.79,65.49) | 0.1 (0,0.2) | 0.3 (0.05,0.56) | 3.32 (0.23,6.87) | 10.13 (1.75,19.5) | 0.28 (0.18,0.41) | 0.55 (0.35,0.81) | 8.89 (5.63,12.67) | 17.19 (10.77,25.47) |
| Poland | 1.41 (-0.05,2.82) | 1.93 (-0.06,3.95) | 31.3 (-1.71,61.96) | 42.01 (-1.47,83.78) | 0.85 (0.19,1.55) | 1.08 (0.28,1.98) | 24.84 (5.31,45.09) | 28.45 (7.06,52.51) | 1.44 (1.02,1.9) | 2.05 (1.44,2.77) | 37.46 (26.63,49.3) | 48.42 (33.82,65.17) |
| Portugal | 1.3 (-0.05,2.55) | 1.17 (-0.04,2.43) | 26.85 (-1.99,52.21) | 25.46 (-1.64,51.47) | 0.33 (0.06,0.63) | 0.39 (0.09,0.71) | 9.25 (1.66,18.1) | 10.34 (2.43,18.64) | 0.96 (0.68,1.35) | 0.97 (0.68,1.35) | 24.83 (17.74,34.57) | 24.84 (17.52,33.96) |
| Puerto Rico | 1.29 (-0.04,2.59) | 1.45 (-0.05,2.85) | 28.46 (-2.01,55.39) | 33.82 (-1.71,66.49) | 0.27 (0.06,0.48) | 0.59 (0.16,1.05) | 7.58 (1.85,13.29) | 17.59 (5.08,31.02) | 0.87 (0.62,1.15) | 1.39 (0.98,1.89) | 22.99 (16.24,30.55) | 39.3 (27.85,53.1) |
| Qatar | 1.99 (-0.06,4.13) | 2.97 (-0.1,5.98) | 44.08 (-1.48,91.93) | 71.7 (-2.58,140.82) | 0.67 (0.15,1.36) | 1.23 (0.38,2.27) | 18.63 (4.15,37.22) | 31.93 (9.75,58.89) | 1.07 (0.59,1.94) | 1.39 (0.92,1.99) | 28.18 (15.73,49.82) | 36.16 (23.77,52.19) |
| Republic of Congo | 0.67 (-0.02,1.62) | 1.59 (-0.04,3.76) | 15.08 (-1.73,38.2) | 35.84 (-3.3,86.23) | 0.1 (0.01,0.23) | 0.38 (0.08,0.74) | 3.17 (0.27,7.56) | 11.82 (2.47,22.94) | 0.5 (0.29,0.79) | 0.99 (0.59,1.53) | 14.69 (8.57,23.72) | 27.55 (15.84,43.25) |
| Romania | 0.96 (-0.03,1.95) | 1.71 (-0.05,3.49) | 21.28 (-1.5,43.74) | 39.54 (-1.22,81.28) | 0.48 (0.1,0.87) | 0.8 (0.18,1.48) | 15.18 (3.32,28.39) | 22.96 (5.19,42.09) | 1.03 (0.68,1.38) | 1.26 (0.87,1.72) | 28.67 (19,38.87) | 34.87 (24.04,47.54) |
| Russia | 1.01 (-0.04,1.96) | 1.78 (-0.06,3.44) | 23.76 (-1.94,45.64) | 42.18 (-1.66,81.28) | 0.75 (0.18,1.35) | 0.94 (0.25,1.64) | 23.5 (5.58,42.28) | 27.59 (7.42,47.69) | 1.77 (1.27,2.34) | 2.2 (1.56,2.85) | 52.94 (37.79,69.43) | 63.38 (44.5,82.66) |
| Rwanda | 0.41 (-0.03,0.93) | 0.72 (-0.03,1.51) | 7.33 (-1.73,17.85) | 13.52 (-1.46,30.46) | 0.06 (-0.02,0.18) | 0.23 (0.03,0.53) | 1.99 (-0.56,5.7) | 7.19 (0.88,16.41) | 0.35 (0.17,0.56) | 0.41 (0.23,0.67) | 9.9 (4.59,15.71) | 10.91 (5.99,17.75) |
| Saint Lucia | 1.36 (-0.07,2.85) | 1.41 (-0.06,2.87) | 27.82 (-3.14,57.81) | 27.37 (-2.57,58.37) | 0.43 (0.08,0.86) | 0.75 (0.2,1.41) | 14.94 (3.34,29.57) | 24.42 (6.82,44.54) | 1.12 (0.77,1.55) | 1.6 (1.06,2.23) | 33.4 (23.03,46.03) | 45.54 (30.6,63.19) |
| Samoa | 1.65 (-0.06,3.32) | 2.42 (-0.06,4.98) | 45.6 (-1.55,92.12) | 66.46 (-1.69,137.12) | 0.37 (0.1,0.69) | 0.64 (0.16,1.2) | 12.06 (3.55,22.19) | 20.35 (5.41,39.33) | 1.5 (0.9,2.34) | 2.12 (1.32,3.28) | 42.64 (25.31,69.56) | 60.25 (37.39,95.72) |
| San Marino | 1.35 (-0.05,2.95) | 0.9 (-0.02,2.02) | 29.29 (-1.3,62.04) | 20.67 (-0.85,45.99) | 0.38 (0.08,0.76) | 0.27 (0.06,0.57) | 9.74 (2.08,19.11) | 7.23 (1.46,15.14) | 0.26 (0.15,0.4) | 0.2 (0.1,0.34) | 6.22 (3.68,9.85) | 5.11 (2.59,8.63) |
| Sao Tome and Principe | 0.58 (-0.02,1.17) | 1.25 (-0.05,2.69) | 13.25 (-0.77,27.24) | 28.42 (-1.52,62.43) | 0.21 (0.04,0.4) | 0.49 (0.12,1.04) | 6.44 (1.23,12.69) | 14.88 (3.61,31.55) | 0.82 (0.52,1.18) | 1.4 (0.84,2.12) | 20.94 (13.15,30.1) | 34.56 (20.93,52.31) |
| Saudi Arabia | 0.34 (-0.02,0.73) | 0.87 (-0.04,1.72) | 6.6 (-1.04,14.59) | 20.01 (-1.84,40.09) | 0.24 (0.05,0.51) | 0.61 (0.19,1.21) | 7.17 (1.55,15.46) | 17.92 (5.37,36.38) | 0.34 (0.19,0.53) | 0.64 (0.42,0.93) | 9.26 (5.23,14.98) | 18.24 (11.84,26.88) |
| Senegal | 0.45 (-0.02,0.9) | 0.87 (-0.03,1.86) | 9.84 (-0.75,20.55) | 19.56 (-1.35,42.87) | 0.08 (0.01,0.15) | 0.18 (0.04,0.37) | 2.48 (0.42,4.92) | 5.65 (1.14,11.54) | 0.44 (0.27,0.67) | 0.73 (0.44,1.12) | 12.12 (7.39,18.32) | 19.11 (11.24,29.29) |
| Serbia | 1.94 (-0.06,3.95) | 2.8 (-0.09,5.84) | 42.28 (-2.42,88.88) | 66.05 (-2.41,135.75) | 0.61 (0.13,1.18) | 1.08 (0.33,1.92) | 17.63 (3.84,34.01) | 30.12 (8.99,53.93) | 1.38 (0.81,2.16) | 1.65 (1.04,2.45) | 34.83 (20.26,54.85) | 43.02 (26.65,64.09) |
| Seychelles | 1.02 (-0.03,2.09) | 2.1 (-0.06,4.09) | 29.8 (-0.82,60.63) | 58.21 (-1.7,113.52) | 0.46 (0.1,0.89) | 1.03 (0.28,1.82) | 16.17 (3.51,30.83) | 34.3 (9.5,60.74) | 0.88 (0.57,1.3) | 1.05 (0.7,1.52) | 27.86 (17.79,41.03) | 31.62 (21.49,45.17) |
| Sierra Leone | 0.4 (-0.02,0.83) | 0.73 (-0.03,1.62) | 8.95 (-0.59,19.09) | 15.91 (-1.04,35.6) | 0.05 (0.01,0.11) | 0.13 (0.02,0.27) | 1.56 (0.19,3.29) | 3.91 (0.7,8.25) | 0.31 (0.18,0.5) | 0.57 (0.35,0.89) | 8.06 (4.83,12.84) | 14.68 (8.84,22.79) |
| Singapore | 0.44 (-0.01,0.89) | 0.67 (-0.02,1.34) | 13.02 (-0.33,26.34) | 19.66 (-0.5,39.52) | 0.06 (-0.03,0.17) | 0.25 (0.06,0.46) | 2.21 (-0.69,5.76) | 7.64 (1.76,13.89) | 0.25 (0.18,0.34) | 0.41 (0.27,0.55) | 7.34 (5.26,10.01) | 11.64 (7.68,15.67) |
| Slovakia | 1.67 (-0.05,3.42) | 2.01 (-0.06,4.19) | 39.73 (-1.43,80.65) | 47.54 (-1.59,97.24) | 0.9 (0.22,1.71) | 0.97 (0.27,1.76) | 25.98 (6.12,49.55) | 26.57 (7.38,47.68) | 2.42 (1.53,3.48) | 2.04 (1.28,3.07) | 63.04 (40.41,89.99) | 52.18 (32.88,81.1) |
| Slovenia | 1.61 (-0.06,3.2) | 1.62 (-0.05,3.41) | 36.08 (-1.54,71.97) | 35.01 (-1.1,73.72) | 0.74 (0.17,1.32) | 0.71 (0.18,1.3) | 20.77 (4.81,36.73) | 18.27 (4.56,33.97) | 1.38 (0.98,1.86) | 1.39 (0.96,1.9) | 35.69 (25.42,48.03) | 35.27 (24.29,48.34) |
| Solomon Islands | 0.79 (-0.02,1.81) | 1.44 (-0.04,3.15) | 21.78 (-0.48,50.54) | 40.56 (-1.15,89.67) | 0.08 (0.01,0.19) | 0.21 (0.04,0.47) | 2.77 (0.43,6.83) | 7.51 (1.57,16.99) | 0.88 (0.37,1.48) | 1.37 (0.67,2.22) | 27.51 (10.62,47.61) | 43.8 (21.28,71.85) |
| Somalia | 0.27 (-0.02,0.59) | 0.38 (-0.02,0.82) | 5.54 (-1.07,12.9) | 7.64 (-1.58,17.46) | 0.07 (0,0.18) | 0.16 (0.01,0.35) | 2.63 (0.1,6.51) | 5.7 (0.62,12.23) | 0.33 (0.16,0.56) | 0.44 (0.22,0.73) | 9.93 (4.61,16.87) | 12.89 (6.48,21.58) |
| South Africa | 1.09 (-0.05,2.13) | 2.23 (-0.09,4.4) | 20.1 (-2.24,41.9) | 47.16 (-3.21,90.9) | 0.39 (0.1,0.74) | 0.83 (0.23,1.47) | 11.67 (3.04,21.9) | 23.6 (6.6,41.6) | 0.62 (0.41,0.94) | 1.18 (0.77,1.58) | 16.19 (10.87,24.3) | 29.3 (19.53,38.81) |
| South Korea | 0.13 (0,0.28) | 0.27 (-0.01,0.55) | 3.96 (-0.11,8.23) | 8.26 (-0.23,16.48) | 0.02 (-0.01,0.06) | 0.12 (0.02,0.23) | 0.56 (-0.49,2.06) | 3.37 (0.42,6.47) | 0.26 (0.14,0.42) | 0.17 (0.1,0.27) | 7.41 (3.77,11.84) | 4.85 (2.7,7.64) |
| South Sudan | 0.25 (-0.01,0.56) | 0.33 (-0.01,0.71) | 5.04 (-0.7,11.88) | 6.65 (-0.87,15.22) | 0.03 (-0.02,0.09) | 0.06 (-0.02,0.17) | 1 (-0.5,2.91) | 2.03 (-0.44,5.47) | 0.22 (0.12,0.36) | 0.28 (0.15,0.46) | 6.11 (3.2,9.82) | 7.75 (3.91,12.92) |
| Spain | 1.49 (-0.05,2.95) | 1.14 (-0.04,2.27) | 32.42 (-2.2,62.74) | 26.4 (-1.02,51.49) | 0.48 (0.1,0.89) | 0.56 (0.14,1) | 13.65 (2.75,25.14) | 14.77 (3.8,26.79) | 1.06 (0.74,1.46) | 1.04 (0.71,1.38) | 27.29 (19.03,37.37) | 26.85 (18.97,35.46) |
| Sri Lanka | 0.35 (-0.01,0.73) | 0.63 (-0.02,1.41) | 9.35 (-0.25,19.73) | 16.8 (-0.46,37.46) | 0.07 (0.01,0.14) | 0.19 (0.04,0.39) | 2.14 (0.18,4.63) | 5.88 (1.25,12.32) | 0.24 (0.15,0.37) | 0.33 (0.19,0.54) | 6.95 (4.3,10.68) | 9.23 (5.15,15.45) |
| Sudan | 0.25 (-0.02,0.54) | 0.57 (-0.03,1.26) | 5.26 (-0.71,11.6) | 12.34 (-1.3,28.32) | 0.12 (0.02,0.33) | 0.27 (0.07,0.58) | 3.54 (0.52,10.02) | 8.27 (2.04,17.55) | 0.27 (0.15,0.52) | 0.43 (0.24,0.76) | 7.68 (4.06,14.64) | 12.58 (7.01,21.68) |
| Suriname | 0.69 (-0.03,1.42) | 1.02 (-0.04,2.06) | 14.39 (-1.06,31.19) | 21.93 (-2.13,45.61) | 0.24 (0.03,0.51) | 0.49 (0.11,0.96) | 7.59 (1.08,15.75) | 15.79 (3.57,30.18) | 0.48 (0.31,0.72) | 0.78 (0.48,1.18) | 13.64 (8.76,20.97) | 22.77 (13.85,34.61) |
| Swaziland | 1.39 (-0.06,2.91) | 2.79 (-0.07,6.12) | 29.09 (-1.7,61.56) | 58.33 (-2.94,132.2) | 0.56 (0.13,1.22) | 1.11 (0.25,2.25) | 15.43 (3.44,32.96) | 30.72 (6.82,62.88) | 1.25 (0.74,1.93) | 2.05 (1.08,3.34) | 30.05 (17.74,46.57) | 49.21 (25.43,83.67) |
| Sweden | 1.24 (-0.04,2.49) | 1.06 (-0.03,2.16) | 28.03 (-1.83,55.91) | 23.44 (-0.78,48.18) | 0.71 (0.14,1.36) | 0.53 (0.12,1) | 19.65 (4.02,37.81) | 13.03 (2.99,24.57) | 0.83 (0.56,1.13) | 0.85 (0.57,1.2) | 20.72 (14.12,28.32) | 20.1 (13.42,28.5) |
| Switzerland | 1.37 (-0.04,2.79) | 1.05 (-0.03,2.15) | 30.91 (-1.26,61.8) | 23.11 (-0.77,46.68) | 0.32 (0.06,0.59) | 0.37 (0.08,0.69) | 8.48 (1.53,15.87) | 8.54 (1.77,15.89) | 0.8 (0.54,1.08) | 0.66 (0.44,0.92) | 19.62 (13.77,26.43) | 16.4 (11.07,22.23) |
| Syria | 0.52 (-0.03,1.06) | 1.15 (-0.04,2.25) | 10.82 (-1.51,23.84) | 25.18 (-1.96,50.56) | 0.16 (0.03,0.34) | 0.38 (0.11,0.69) | 4.97 (1.01,10.05) | 10.9 (3.14,19.84) | 0.46 (0.28,0.71) | 0.6 (0.38,0.92) | 13.03 (8.24,19.73) | 16.57 (10.39,26.02) |
| Taiwan | 0.35 (-0.01,0.69) | 0.9 (-0.02,1.87) | 9.85 (-0.25,19.53) | 26.13 (-0.68,53.46) | 0.07 (0,0.14) | 0.26 (0.05,0.49) | 1.89 (-0.05,4.03) | 8.51 (1.65,15.85) | 0.12 (0.09,0.17) | 0.42 (0.29,0.58) | 3.57 (2.42,4.75) | 13.79 (9.48,19.02) |
| Tajikistan | 0.73 (-0.03,1.52) | 0.73 (-0.03,1.6) | 15.82 (-1.54,32.1) | 16.67 (-1.06,36.13) | 0.15 (0.03,0.31) | 0.2 (0.05,0.4) | 4.58 (0.97,9.23) | 6.2 (1.41,12.51) | 1.01 (0.61,1.48) | 0.94 (0.54,1.58) | 29.82 (17.68,43.79) | 27.4 (14.97,48.47) |
| Tanzania | 0.55 (-0.02,1.09) | 1.22 (-0.03,2.66) | 11.32 (-1.05,22.92) | 25.58 (-1.27,56.33) | 0.18 (0.02,0.35) | 0.52 (0.11,1) | 5.51 (0.81,10.77) | 15.58 (3.46,30.62) | 0.47 (0.23,0.73) | 0.77 (0.46,1.21) | 12.87 (6.35,19.93) | 20.51 (12.12,31.97) |
| Thailand | 0.33 (-0.01,0.67) | 0.99 (-0.03,2.12) | 9.73 (-0.27,19.47) | 28.36 (-0.86,61.12) | 0.08 (0,0.17) | 0.34 (0.08,0.67) | 2.67 (0.11,5.6) | 11.52 (2.7,22.74) | 0.22 (0.13,0.32) | 0.46 (0.24,0.71) | 6.68 (4.02,9.76) | 14.21 (7.41,22.11) |
| Timor-Leste | 0.13 (0,0.29) | 0.26 (-0.01,0.57) | 3.57 (-0.08,8.24) | 7.44 (-0.17,17.15) | 0 (-0.02,0.01) | 0.03 (-0.01,0.1) | -0.04 (-0.49,0.54) | 1.39 (-0.32,3.75) | 0.11 (0.06,0.18) | 0.18 (0.1,0.3) | 3.2 (1.71,5.36) | 5.9 (3.22,9.83) |
| Tobago | 1.65 (-0.06,3.18) | 2 (-0.08,4.1) | 37.73 (-2.92,74.01) | 45.63 (-3.68,92.17) | 0.64 (0.15,1.22) | 0.92 (0.25,1.66) | 20.53 (4.79,38) | 29.37 (8.2,54.33) | 1.66 (1.17,2.21) | 2.5 (1.66,3.52) | 46.96 (33.48,63.14) | 71.09 (47.04,101.34) |
| Tobago | 1.65 (-0.06,3.18) | 2 (-0.08,4.1) | 37.73 (-2.92,74.01) | 45.63 (-3.68,92.17) | 0.64 (0.15,1.22) | 0.92 (0.25,1.66) | 20.53 (4.79,38) | 29.37 (8.2,54.33) | 1.66 (1.17,2.21) | 2.5 (1.66,3.52) | 46.96 (33.48,63.14) | 71.09 (47.04,101.34) |
| Togo | 0.48 (-0.02,0.98) | 0.91 (-0.03,1.94) | 10.45 (-0.79,22.23) | 19.76 (-1.22,42.89) | 0.07 (0.01,0.14) | 0.17 (0.03,0.37) | 2.14 (0.37,4.52) | 5.33 (1.07,10.96) | 0.4 (0.25,0.63) | 0.71 (0.4,1.08) | 10.73 (6.82,16.52) | 17.95 (10.11,27.82) |
| Tokelau | 1.8 (-0.05,4.06) | 2.85 (-0.08,6.25) | 48.15 (-1.39,109.49) | 74.8 (-2.17,160.94) | 0.18 (0.04,0.39) | 0.42 (0.12,0.83) | 6.29 (1.36,13.14) | 14.17 (4.22,27.87) | 1.37 (0.77,2.21) | 1.79 (0.92,2.93) | 41.67 (23.26,67.69) | 54.94 (28.2,89.59) |
| Tonga | 3.25 (-0.11,6.4) | 4.51 (-0.15,9.39) | 91.89 (-2.99,182.27) | 120.66 (-4.12,247.97) | 0.22 (0.05,0.39) | 0.42 (0.12,0.78) | 7.18 (1.78,12.58) | 13.16 (3.94,24.36) | 1.35 (0.77,2.01) | 1.74 (0.86,2.66) | 41.63 (23.19,60.41) | 51.62 (25.55,78.36) |
| Tunisia | 0.48 (-0.02,0.95) | 0.85 (-0.04,1.8) | 10.52 (-0.91,21.64) | 19.52 (-1.67,41.21) | 0.19 (0.04,0.37) | 0.45 (0.12,0.88) | 5.69 (1.23,10.86) | 13.27 (3.54,25.3) | 0.26 (0.16,0.4) | 0.38 (0.23,0.59) | 7.13 (4.56,10.57) | 10.41 (6.32,16.75) |
| Turkey | 0.4 (-0.01,0.79) | 1.19 (-0.04,2.42) | 10.08 (-0.58,20.49) | 27.09 (-1.87,55.24) | 0.54 (0.12,1.12) | 0.77 (0.22,1.4) | 16.17 (3.46,33.96) | 22.07 (6.07,39.86) | 1.04 (0.62,1.56) | 1.02 (0.62,1.47) | 28.74 (17.19,42.92) | 27.88 (16.9,40.34) |
| Turkmenistan | 0.74 (-0.03,1.45) | 0.72 (-0.03,1.44) | 16.75 (-1.32,32.78) | 15.98 (-2.13,32.69) | 0.19 (0.04,0.36) | 0.42 (0.09,0.82) | 5.97 (1.2,11.27) | 13.47 (2.79,26.25) | 0.7 (0.48,0.97) | 0.55 (0.35,0.79) | 20.4 (13.98,28.44) | 16.56 (10.67,23.91) |
| Tuvalu | 1.34 (-0.03,3.05) | 2.5 (-0.07,5.37) | 36.07 (-0.85,83.91) | 65.64 (-1.98,141.1) | 0.14 (0.03,0.3) | 0.38 (0.1,0.74) | 4.77 (0.95,10.1) | 12.34 (3.24,24.38) | 1.35 (0.75,2.08) | 1.92 (0.95,3.08) | 41.1 (22.8,63.83) | 57.78 (28.52,93.08) |
| Uganda | 0.43 (-0.01,0.98) | 0.94 (-0.03,2) | 9.73 (-0.91,22.59) | 21.28 (-1.82,44.75) | 0.08 (-0.01,0.21) | 0.31 (0.04,0.65) | 2.71 (-0.19,6.4) | 9.97 (1.34,21.02) | 0.34 (0.2,0.53) | 0.69 (0.43,1.02) | 9.35 (5.48,14.6) | 18.95 (11.41,28.57) |
| UK | 2.49 (-0.08,5.09) | 1.71 (-0.06,3.43) | 55.95 (-2.73,113.52) | 36.56 (-1.55,71.04) | 0.95 (0.21,1.81) | 0.8 (0.2,1.47) | 26.74 (5.93,51.27) | 20.04 (4.89,36.73) | 0.84 (0.6,1.14) | 1.3 (0.91,1.73) | 21.16 (15.1,28.36) | 31.42 (22.21,41.55) |
| Ukraine | 1.47 (-0.06,2.94) | 1.62 (-0.05,3.47) | 34.85 (-2.44,69.65) | 39.93 (-1.48,83.81) | 0.54 (0.12,0.95) | 0.77 (0.18,1.52) | 16.91 (3.82,30.41) | 24.1 (5.51,48.03) | 1.46 (1.01,1.93) | 1.95 (1.16,2.97) | 40.85 (28.31,54.11) | 55.63 (32.7,85.1) |
| United Arab Emirates | 1.55 (-0.05,3.25) | 5.57 (-0.19,11.44) | 40.03 (-1.45,85.47) | 114.78 (-4.19,235.4) | 0.83 (0.14,2) | 3.73 (1.05,6.55) | 24.32 (4.07,60.28) | 80.08 (22.77,140.04) | 1.87 (0.93,3.24) | 5.35 (3.45,8.09) | 52.92 (25.36,90.82) | 112.77 (73.91,170.03) |
| Uruguay | 2.44 (-0.07,4.86) | 2.46 (-0.08,4.95) | 55.77 (-2.38,110.78) | 52.76 (-2.17,104.67) | 0.58 (0.13,1.1) | 0.74 (0.19,1.34) | 16.2 (3.66,30.99) | 20.97 (5.2,37.6) | 0.96 (0.67,1.3) | 0.88 (0.61,1.19) | 24.59 (17.05,33.55) | 22.76 (15.89,30.39) |
| USA | 2.05 (-0.06,4.04) | 1.64 (-0.06,3.18) | 50.42 (-2.17,98.26) | 38.88 (-1.85,74.77) | 0.86 (0.21,1.55) | 0.79 (0.22,1.37) | 24 (5.91,43.36) | 20.97 (5.83,36.19) | 1.06 (0.75,1.43) | 1.62 (1.16,2.06) | 28.44 (20.36,37.69) | 46.19 (33.84,58.22) |
| Uzbekistan | 0.83 (-0.03,1.7) | 0.88 (-0.03,1.81) | 18.83 (-1.41,38.93) | 21.12 (-1.47,43.65) | 0.17 (0.04,0.33) | 0.35 (0.08,0.68) | 5.31 (1.12,10.49) | 11.07 (2.53,21.64) | 0.8 (0.54,1.13) | 0.75 (0.49,1.08) | 23.48 (15.67,32.99) | 22.93 (15.05,32.55) |
| Vanuatu | 0.82 (-0.02,1.74) | 1.46 (-0.04,3.1) | 21 (-0.48,45.94) | 37.68 (-1.11,80.82) | 0.06 (0.01,0.15) | 0.16 (0.03,0.37) | 1.98 (0.28,5.11) | 5.03 (0.98,12.1) | 0.81 (0.39,1.33) | 1.29 (0.63,2.04) | 23.9 (11.47,41.4) | 38.42 (18.76,61.16) |
| Venezuela | 0.89 (-0.04,1.77) | 1.66 (-0.07,3.31) | 19.46 (-1.66,39.13) | 37.64 (-2.86,75.52) | 0.13 (0.03,0.24) | 0.62 (0.17,1.14) | 3.82 (0.86,7.11) | 19.1 (5.41,35.38) | 1.27 (0.89,1.72) | 1.21 (0.76,1.75) | 33.88 (23.92,45.12) | 32.69 (20.51,47.74) |
| Vietnam | 0.1 (0,0.23) | 0.28 (-0.01,0.65) | 2.93 (-0.08,6.43) | 8.04 (-0.19,18.4) | 0 (-0.01,0.01) | 0.05 (0,0.11) | 0.03 (-0.3,0.4) | 1.45 (0.02,3.43) | 0.05 (0.03,0.08) | 0.12 (0.07,0.19) | 1.37 (0.87,2.15) | 3.4 (1.98,5.49) |
| Virgin Islands | 2.25 (-0.08,4.52) | 1.63 (-0.08,3.36) | 47 (-3.36,99.29) | 32.2 (-2.72,69.02) | 0.71 (0.17,1.35) | 0.81 (0.23,1.56) | 21.12 (5.28,39.81) | 25.8 (7.26,49.39) | 1.56 (1.03,2.27) | 1.05 (0.62,1.78) | 42.13 (28.55,60.57) | 29.91 (17.32,52.4) |
| Yemen | 0.14 (-0.01,0.29) | 0.33 (-0.02,0.72) | 2.94 (-0.54,6.66) | 7.24 (-0.97,15.47) | 0.04 (0,0.13) | 0.13 (0.02,0.29) | 1.38 (0.13,3.94) | 3.88 (0.72,8.52) | 0.15 (0.08,0.31) | 0.26 (0.14,0.49) | 4.39 (2.17,8.77) | 7.43 (4.07,13.26) |
| Zambia | 0.37 (-0.03,0.79) | 1.27 (-0.05,2.87) | 5.9 (-1.79,15.4) | 24.88 (-2.09,57.06) | 0.09 (-0.01,0.22) | 0.44 (0.1,0.89) | 2.81 (-0.14,6.72) | 13.44 (2.93,26.94) | 0.39 (0.19,0.63) | 0.91 (0.44,1.91) | 10.81 (5.27,17.67) | 25.22 (11.62,55.39) |
| Zimbabwe | 0.64 (-0.03,1.42) | 1.85 (-0.05,3.88) | 14.52 (-1.1,32.71) | 41.87 (-3.51,88.74) | 0.2 (0.04,0.42) | 0.77 (0.18,1.49) | 6.37 (1.25,13.11) | 23.47 (5.52,46.01) | 0.65 (0.4,0.99) | 1.92 (1.15,2.92) | 17.33 (10.37,26.61) | 51.55 (30.36,77.38) |
