# Supplementary material for: High BMI-attributable female-specific cancers: a comprehensive analysis of the global disease burden and trends from 1990 to 2021 and projections to 2040
Source: Front Oncol. 2025 Oct 29;15:1704299. doi: 10.3389/fonc.2025.1704299 (PMC12605095; doi:10.3389/fonc.2025.1704299)
Supplement: Supplementary file 7 [file Table5.docx]

**Table S5**

ASDR and ASRDALY of breast cancer, ovarian cancer and uterine cancer in 21 SDI regions, 1990-2021.

|  | | | **Breast cancer** | | **Ovarian cancer** | | **Uterine cancer** | |
| --- | --- | --- | --- | --- | --- | --- | --- | --- |
| **location** | **year** | **SDI** | **ASDR** | **ASRDALY** | **ASDR** | **ASRDALY** | **ASDR** | **ASRDALY** |
| Andean Latin America | 1990 | 0.500011 | 0.577035 | 12.06519 | 0.162829 | 5.199767 | 1.179984 | 33.38528 |
| Andean Latin America | 1991 | 0.501652 | 0.597351 | 12.66112 | 0.166927 | 5.313262 | 1.188717 | 33.46641 |
| Andean Latin America | 1992 | 0.503656 | 0.627985 | 13.34257 | 0.177174 | 5.633173 | 1.237746 | 34.91415 |
| Andean Latin America | 1993 | 0.506388 | 0.627798 | 13.29241 | 0.183106 | 5.811384 | 1.21395 | 34.20074 |
| Andean Latin America | 1994 | 0.510306 | 0.661405 | 14.11903 | 0.193585 | 6.135838 | 1.19918 | 33.69217 |
| Andean Latin America | 1995 | 0.514896 | 0.695278 | 14.88602 | 0.206613 | 6.538723 | 1.122753 | 31.46124 |
| Andean Latin America | 1996 | 0.519376 | 0.703381 | 15.07043 | 0.235966 | 7.447031 | 0.987805 | 27.69532 |
| Andean Latin America | 1997 | 0.524036 | 0.713486 | 15.24513 | 0.259655 | 8.214571 | 1.117577 | 31.13131 |
| Andean Latin America | 1998 | 0.528444 | 0.712281 | 15.17163 | 0.276064 | 8.789266 | 1.162324 | 32.1373 |
| Andean Latin America | 1999 | 0.532744 | 0.717796 | 15.4087 | 0.297491 | 9.434036 | 1.176736 | 32.41722 |
| Andean Latin America | 2000 | 0.537561 | 0.730262 | 15.89638 | 0.313959 | 9.967199 | 1.160101 | 31.98638 |
| Andean Latin America | 2001 | 0.542572 | 0.739904 | 16.07884 | 0.324425 | 10.31462 | 1.193803 | 32.8811 |
| Andean Latin America | 2002 | 0.547911 | 0.752237 | 16.30915 | 0.339029 | 10.75236 | 1.267777 | 34.95013 |
| Andean Latin America | 2003 | 0.553109 | 0.770761 | 16.85095 | 0.350276 | 11.11257 | 1.291314 | 35.62371 |
| Andean Latin America | 2004 | 0.558235 | 0.76098 | 16.7963 | 0.359327 | 11.37264 | 1.315701 | 36.42498 |
| Andean Latin America | 2005 | 0.563092 | 0.767483 | 17.01355 | 0.369039 | 11.65546 | 1.244239 | 34.16513 |
| Andean Latin America | 2006 | 0.567987 | 0.780307 | 17.28235 | 0.375472 | 11.84251 | 1.231211 | 33.74446 |
| Andean Latin America | 2007 | 0.572999 | 0.766616 | 16.96175 | 0.381732 | 11.98746 | 1.186126 | 32.37987 |
| Andean Latin America | 2008 | 0.578564 | 0.785603 | 17.51504 | 0.39761 | 12.48143 | 1.177642 | 32.22301 |
| Andean Latin America | 2009 | 0.583999 | 0.830618 | 18.70054 | 0.413305 | 12.99524 | 1.201084 | 32.96677 |
| Andean Latin America | 2010 | 0.590201 | 0.837038 | 18.80643 | 0.42407 | 13.30255 | 1.190059 | 32.53132 |
| Andean Latin America | 2011 | 0.596945 | 0.822218 | 18.46068 | 0.427322 | 13.40206 | 1.153552 | 31.52507 |
| Andean Latin America | 2012 | 0.603839 | 0.851859 | 19.15364 | 0.435844 | 13.64652 | 1.152753 | 31.49988 |
| Andean Latin America | 2013 | 0.610705 | 0.844774 | 18.90659 | 0.446331 | 13.94275 | 1.136649 | 30.91362 |
| Andean Latin America | 2014 | 0.61706 | 0.832667 | 18.59965 | 0.453707 | 14.14922 | 1.12226 | 30.56686 |
| Andean Latin America | 2015 | 0.622761 | 0.83371 | 18.73108 | 0.464922 | 14.52064 | 1.090985 | 29.71497 |
| Andean Latin America | 2016 | 0.628161 | 0.855785 | 19.1884 | 0.476448 | 14.89859 | 1.108833 | 30.29146 |
| Andean Latin America | 2017 | 0.633538 | 0.893632 | 19.85434 | 0.492564 | 15.35091 | 1.166158 | 31.89709 |
| Andean Latin America | 2018 | 0.63886 | 0.93723 | 20.85696 | 0.499943 | 15.62412 | 1.196329 | 32.86125 |
| Andean Latin America | 2019 | 0.643921 | 0.966876 | 21.3847 | 0.509682 | 15.85674 | 1.25087 | 34.2729 |
| Andean Latin America | 2020 | 0.647807 | 0.949 | 21.41683 | 0.494853 | 15.33814 | 1.211069 | 33.23721 |
| Andean Latin America | 2021 | 0.651602 | 0.950226 | 21.44599 | 0.501234 | 15.54962 | 1.210232 | 33.28554 |
| Australasia | 1990 | 0.731234 | 1.649611 | 38.36231 | 0.849497 | 23.67639 | 0.65135 | 16.82033 |
| Australasia | 1991 | 0.734915 | 1.658229 | 38.88531 | 0.833959 | 23.16552 | 0.639929 | 16.59438 |
| Australasia | 1992 | 0.739006 | 1.630225 | 38.06776 | 0.821056 | 22.81313 | 0.632539 | 16.39645 |
| Australasia | 1993 | 0.743426 | 1.67984 | 39.49596 | 0.829198 | 22.67169 | 0.652673 | 16.90648 |
| Australasia | 1994 | 0.747726 | 1.698664 | 40.11499 | 0.833665 | 22.91698 | 0.650367 | 16.86837 |
| Australasia | 1995 | 0.752015 | 1.685339 | 40.03197 | 0.842321 | 22.94961 | 0.667063 | 17.44969 |
| Australasia | 1996 | 0.756387 | 1.641079 | 38.98837 | 0.871623 | 23.84952 | 0.653149 | 17.01437 |
| Australasia | 1997 | 0.76099 | 1.616649 | 38.63423 | 0.864055 | 23.57595 | 0.655527 | 17.16113 |
| Australasia | 1998 | 0.7653 | 1.597793 | 38.14273 | 0.878511 | 23.8961 | 0.667914 | 17.45042 |
| Australasia | 1999 | 0.769521 | 1.570879 | 37.57913 | 0.872739 | 23.60038 | 0.66931 | 17.44491 |
| Australasia | 2000 | 0.773791 | 1.577694 | 37.81304 | 0.877213 | 23.51727 | 0.682206 | 17.972 |
| Australasia | 2001 | 0.778074 | 1.593631 | 38.52966 | 0.88929 | 23.71791 | 0.688098 | 18.29368 |
| Australasia | 2002 | 0.78248 | 1.610007 | 38.9927 | 0.907969 | 24.24641 | 0.706287 | 18.68591 |
| Australasia | 2003 | 0.786285 | 1.577521 | 37.84149 | 0.889222 | 23.81332 | 0.669401 | 17.6869 |
| Australasia | 2004 | 0.789428 | 1.554181 | 37.21242 | 0.91972 | 24.31385 | 0.663912 | 17.47448 |
| Australasia | 2005 | 0.791722 | 1.522077 | 36.56168 | 0.91217 | 24.01161 | 0.675722 | 17.80639 |
| Australasia | 2006 | 0.792857 | 1.500854 | 36.09495 | 0.890383 | 23.47246 | 0.668993 | 17.6257 |
| Australasia | 2007 | 0.79395 | 1.502042 | 36.00335 | 0.888021 | 23.41165 | 0.669243 | 17.62601 |
| Australasia | 2008 | 0.796402 | 1.510018 | 36.05952 | 0.887517 | 23.21349 | 0.673199 | 17.71949 |
| Australasia | 2009 | 0.799932 | 1.507379 | 35.96324 | 0.883748 | 23.21864 | 0.689486 | 18.24166 |
| Australasia | 2010 | 0.804112 | 1.505359 | 35.67048 | 0.765203 | 20.12779 | 0.717208 | 18.85435 |
| Australasia | 2011 | 0.808161 | 1.49976 | 35.50072 | 0.851425 | 22.26614 | 0.706631 | 18.56953 |
| Australasia | 2012 | 0.812464 | 1.484176 | 35.36828 | 0.851389 | 22.07747 | 0.722598 | 19.04911 |
| Australasia | 2013 | 0.817349 | 1.492462 | 35.62233 | 0.856223 | 22.17998 | 0.729946 | 19.23091 |
| Australasia | 2014 | 0.821647 | 1.486759 | 35.45608 | 0.848152 | 21.80028 | 0.761686 | 19.96988 |
| Australasia | 2015 | 0.825611 | 1.480043 | 34.8953 | 0.697041 | 17.83433 | 0.860441 | 22.39724 |
| Australasia | 2016 | 0.829457 | 1.44794 | 33.729 | 0.651484 | 16.68911 | 0.904987 | 23.65729 |
| Australasia | 2017 | 0.832924 | 1.420194 | 33.1752 | 0.635022 | 16.26397 | 0.901193 | 23.64891 |
| Australasia | 2018 | 0.836619 | 1.41554 | 32.87853 | 0.625217 | 15.96882 | 0.899939 | 23.43018 |
| Australasia | 2019 | 0.840582 | 1.426664 | 32.9497 | 0.631573 | 16.15065 | 0.908374 | 23.66822 |
| Australasia | 2020 | 0.843333 | 1.349668 | 31.48377 | 0.597244 | 15.34129 | 0.855318 | 22.4358 |
| Australasia | 2021 | 0.845514 | 1.389318 | 32.15405 | 0.615067 | 15.6745 | 0.882417 | 22.96926 |
| Caribbean | 1990 | 0.518111 | 0.939636 | 20.0998 | 0.219802 | 6.809402 | 1.073877 | 30.86165 |
| Caribbean | 1991 | 0.522789 | 0.941844 | 20.10744 | 0.226401 | 7.02369 | 1.077244 | 30.91536 |
| Caribbean | 1992 | 0.526984 | 0.961015 | 20.32799 | 0.244878 | 7.61954 | 1.09964 | 31.56807 |
| Caribbean | 1993 | 0.530507 | 0.991228 | 21.30918 | 0.255792 | 7.965363 | 1.13162 | 32.53859 |
| Caribbean | 1994 | 0.533584 | 0.998926 | 21.63943 | 0.259171 | 8.079685 | 1.160577 | 33.57698 |
| Caribbean | 1995 | 0.536651 | 0.985084 | 21.02967 | 0.273996 | 8.558844 | 1.223201 | 35.46162 |
| Caribbean | 1996 | 0.539776 | 1.007282 | 21.26517 | 0.283176 | 8.850487 | 1.269047 | 36.89467 |
| Caribbean | 1997 | 0.543303 | 0.958924 | 20.15408 | 0.300484 | 9.385185 | 1.379534 | 40.11899 |
| Caribbean | 1998 | 0.547365 | 0.975852 | 20.80406 | 0.304909 | 9.56156 | 1.450141 | 42.2318 |
| Caribbean | 1999 | 0.55206 | 1.002619 | 21.45209 | 0.327642 | 10.25639 | 1.480781 | 43.20624 |
| Caribbean | 2000 | 0.557369 | 1.032935 | 22.33732 | 0.346698 | 10.80584 | 1.496967 | 43.48767 |
| Caribbean | 2001 | 0.56311 | 1.072446 | 23.46253 | 0.348315 | 10.86852 | 1.540901 | 44.82457 |
| Caribbean | 2002 | 0.569069 | 1.071116 | 23.42107 | 0.339037 | 10.56704 | 1.459016 | 42.14131 |
| Caribbean | 2003 | 0.574889 | 1.091681 | 23.7225 | 0.348575 | 10.83557 | 1.530703 | 44.03789 |
| Caribbean | 2004 | 0.580533 | 1.114409 | 24.19444 | 0.352189 | 10.94222 | 1.548119 | 44.36516 |
| Caribbean | 2005 | 0.585758 | 1.101829 | 23.78679 | 0.361689 | 11.18237 | 1.60129 | 45.784 |
| Caribbean | 2006 | 0.590602 | 1.102495 | 23.93956 | 0.367363 | 11.32023 | 1.585801 | 45.1617 |
| Caribbean | 2007 | 0.59471 | 1.162853 | 25.33016 | 0.379138 | 11.72889 | 1.643866 | 46.82364 |
| Caribbean | 2008 | 0.598297 | 1.197113 | 26.14597 | 0.387289 | 11.96812 | 1.687713 | 47.93267 |
| Caribbean | 2009 | 0.601655 | 1.22238 | 26.59346 | 0.394002 | 12.15251 | 1.704184 | 48.265 |
| Caribbean | 2010 | 0.60548 | 1.246029 | 27.24578 | 0.409284 | 12.64195 | 1.665084 | 47.17954 |
| Caribbean | 2011 | 0.609525 | 1.257466 | 27.578 | 0.418855 | 12.91014 | 1.607177 | 45.69822 |
| Caribbean | 2012 | 0.613255 | 1.269676 | 27.70923 | 0.414143 | 12.73764 | 1.617153 | 45.85386 |
| Caribbean | 2013 | 0.616877 | 1.268215 | 27.49266 | 0.422693 | 13.04286 | 1.718023 | 48.49037 |
| Caribbean | 2014 | 0.620443 | 1.283184 | 27.6443 | 0.42773 | 13.21464 | 1.761235 | 49.79623 |
| Caribbean | 2015 | 0.624075 | 1.282669 | 27.62968 | 0.439644 | 13.62824 | 1.787611 | 50.52189 |
| Caribbean | 2016 | 0.627582 | 1.283 | 27.64802 | 0.441823 | 13.71663 | 1.824698 | 51.25674 |
| Caribbean | 2017 | 0.630788 | 1.277327 | 27.35051 | 0.449239 | 13.92348 | 1.85059 | 51.99652 |
| Caribbean | 2018 | 0.633972 | 1.280669 | 27.46495 | 0.452915 | 14.05985 | 1.889092 | 53.12176 |
| Caribbean | 2019 | 0.637321 | 1.308704 | 28.17139 | 0.460782 | 14.29859 | 1.937209 | 54.44291 |
| Caribbean | 2020 | 0.639709 | 1.296788 | 27.9468 | 0.45692 | 14.12233 | 1.914099 | 53.53608 |
| Caribbean | 2021 | 0.642003 | 1.308754 | 28.164 | 0.461433 | 14.2295 | 1.923021 | 53.69196 |
| Central Asia | 1990 | 0.553362 | 1.163439 | 26.86293 | 0.293856 | 9.076609 | 1.309963 | 37.68992 |
| Central Asia | 1991 | 0.555148 | 1.059488 | 24.60953 | 0.306335 | 9.469142 | 1.376064 | 39.94064 |
| Central Asia | 1992 | 0.55727 | 1.045346 | 24.12666 | 0.325161 | 10.05141 | 1.274405 | 37.10587 |
| Central Asia | 1993 | 0.560083 | 1.048617 | 24.2317 | 0.337988 | 10.49095 | 1.221359 | 35.58906 |
| Central Asia | 1994 | 0.5627 | 1.046286 | 24.11666 | 0.346191 | 10.79711 | 1.191817 | 34.9992 |
| Central Asia | 1995 | 0.565586 | 1.051154 | 24.0706 | 0.367424 | 11.49837 | 1.187285 | 34.96403 |
| Central Asia | 1996 | 0.568761 | 1.080469 | 24.63728 | 0.361592 | 11.31843 | 1.21666 | 35.79687 |
| Central Asia | 1997 | 0.571766 | 1.101262 | 25.32701 | 0.356348 | 11.13639 | 1.256287 | 37.00055 |
| Central Asia | 1998 | 0.574659 | 1.122547 | 26.25572 | 0.353654 | 11.03637 | 1.267544 | 37.53415 |
| Central Asia | 1999 | 0.578121 | 1.150043 | 27.08268 | 0.348823 | 10.86556 | 1.249576 | 36.87147 |
| Central Asia | 2000 | 0.582156 | 1.173306 | 27.83861 | 0.364173 | 11.3422 | 1.242039 | 36.77663 |
| Central Asia | 2001 | 0.586646 | 1.176796 | 27.91538 | 0.374408 | 11.68142 | 1.21342 | 35.83699 |
| Central Asia | 2002 | 0.591457 | 1.184172 | 28.07554 | 0.377867 | 11.75545 | 1.184142 | 34.76825 |
| Central Asia | 2003 | 0.59653 | 1.208718 | 28.65042 | 0.399001 | 12.38352 | 1.179605 | 34.61481 |
| Central Asia | 2004 | 0.601737 | 1.195811 | 28.3135 | 0.416909 | 12.92582 | 1.152064 | 33.8161 |
| Central Asia | 2005 | 0.607294 | 1.18957 | 28.22568 | 0.425266 | 13.23312 | 1.089001 | 32.06041 |
| Central Asia | 2006 | 0.613078 | 1.162413 | 27.48175 | 0.430772 | 13.36459 | 0.974376 | 28.88936 |
| Central Asia | 2007 | 0.619105 | 1.162634 | 27.52527 | 0.441143 | 13.68521 | 0.950502 | 28.20024 |
| Central Asia | 2008 | 0.62493 | 1.199896 | 28.4127 | 0.461586 | 14.24304 | 0.953671 | 28.21585 |
| Central Asia | 2009 | 0.630103 | 1.210937 | 28.5453 | 0.481587 | 14.73545 | 0.979775 | 28.82133 |
| Central Asia | 2010 | 0.635296 | 1.227967 | 28.7829 | 0.489484 | 14.91856 | 1.049538 | 30.63516 |
| Central Asia | 2011 | 0.640314 | 1.235533 | 28.72307 | 0.499108 | 15.10016 | 1.110403 | 32.16754 |
| Central Asia | 2012 | 0.644674 | 1.242164 | 28.63299 | 0.510945 | 15.44005 | 1.143253 | 33.22256 |
| Central Asia | 2013 | 0.648888 | 1.188671 | 27.66996 | 0.492093 | 14.89054 | 1.136242 | 32.79878 |
| Central Asia | 2014 | 0.653 | 1.17316 | 27.38891 | 0.49986 | 15.12854 | 1.10316 | 31.82906 |
| Central Asia | 2015 | 0.656818 | 1.205017 | 28.07234 | 0.503561 | 15.21338 | 1.110924 | 32.00685 |
| Central Asia | 2016 | 0.660312 | 1.209827 | 28.10774 | 0.517771 | 15.62929 | 1.157851 | 33.02304 |
| Central Asia | 2017 | 0.663577 | 1.18565 | 27.60657 | 0.516376 | 15.60701 | 1.148959 | 32.63667 |
| Central Asia | 2018 | 0.666666 | 1.168018 | 27.20111 | 0.531265 | 16.01991 | 1.135099 | 32.24222 |
| Central Asia | 2019 | 0.669837 | 1.152583 | 26.84295 | 0.531185 | 15.94051 | 1.123373 | 31.73048 |
| Central Asia | 2020 | 0.672487 | 1.164457 | 27.25546 | 0.529927 | 15.80534 | 1.120916 | 31.46347 |
| Central Asia | 2021 | 0.675164 | 1.145624 | 26.91997 | 0.526808 | 15.76607 | 1.10563 | 31.19803 |
| Central Europe | 1990 | 0.637272 | 1.458843 | 32.22769 | 0.704509 | 20.58612 | 1.480941 | 38.88257 |
| Central Europe | 1991 | 0.643083 | 1.491252 | 32.95531 | 0.712387 | 20.81323 | 1.502019 | 39.67054 |
| Central Europe | 1992 | 0.648772 | 1.527967 | 33.813 | 0.722141 | 21.16877 | 1.517376 | 39.96349 |
| Central Europe | 1993 | 0.65447 | 1.554966 | 34.41244 | 0.737039 | 21.51181 | 1.488101 | 39.14158 |
| Central Europe | 1994 | 0.661219 | 1.586296 | 35.18591 | 0.746705 | 21.76034 | 1.476657 | 38.6947 |
| Central Europe | 1995 | 0.667955 | 1.627422 | 36.21096 | 0.770954 | 22.50887 | 1.474683 | 38.71506 |
| Central Europe | 1996 | 0.674121 | 1.645504 | 36.60242 | 0.777042 | 22.5592 | 1.486288 | 39.07488 |
| Central Europe | 1997 | 0.680037 | 1.681956 | 37.60819 | 0.786889 | 22.86627 | 1.51174 | 39.81489 |
| Central Europe | 1998 | 0.685968 | 1.702911 | 38.25632 | 0.807004 | 23.44123 | 1.518076 | 39.81431 |
| Central Europe | 1999 | 0.692056 | 1.747956 | 39.51893 | 0.827408 | 23.9638 | 1.530526 | 40.07134 |
| Central Europe | 2000 | 0.69878 | 1.743172 | 39.48953 | 0.853298 | 24.56672 | 1.507949 | 39.34711 |
| Central Europe | 2001 | 0.705657 | 1.755269 | 39.85542 | 0.875243 | 25.16046 | 1.458725 | 38.19331 |
| Central Europe | 2002 | 0.712001 | 1.768526 | 40.35554 | 0.883977 | 25.33917 | 1.443327 | 37.83611 |
| Central Europe | 2003 | 0.717824 | 1.801321 | 41.20946 | 0.913322 | 26.04615 | 1.458445 | 38.19227 |
| Central Europe | 2004 | 0.723494 | 1.803787 | 41.32794 | 0.922475 | 26.221 | 1.445279 | 37.84801 |
| Central Europe | 2005 | 0.728674 | 1.824196 | 41.97199 | 0.93258 | 26.50796 | 1.434132 | 37.87789 |
| Central Europe | 2006 | 0.733476 | 1.808676 | 41.73211 | 0.933937 | 26.56886 | 1.432602 | 37.88623 |
| Central Europe | 2007 | 0.738077 | 1.814632 | 42.01127 | 0.936201 | 26.60008 | 1.434476 | 37.93276 |
| Central Europe | 2008 | 0.743148 | 1.809979 | 41.86513 | 0.935004 | 26.45512 | 1.437297 | 37.96348 |
| Central Europe | 2009 | 0.748766 | 1.807186 | 41.93077 | 0.936689 | 26.51101 | 1.440027 | 37.91424 |
| Central Europe | 2010 | 0.754919 | 1.766354 | 40.8163 | 0.919692 | 25.97157 | 1.428547 | 37.55619 |
| Central Europe | 2011 | 0.760349 | 1.77333 | 40.95909 | 0.931557 | 26.21945 | 1.464063 | 38.56004 |
| Central Europe | 2012 | 0.764803 | 1.794521 | 41.4854 | 0.926282 | 26.04234 | 1.497687 | 39.33633 |
| Central Europe | 2013 | 0.768568 | 1.798756 | 41.46196 | 0.93981 | 26.43798 | 1.527601 | 40.05102 |
| Central Europe | 2014 | 0.771755 | 1.824168 | 41.81271 | 0.955361 | 26.78391 | 1.55867 | 40.60871 |
| Central Europe | 2015 | 0.774844 | 1.864392 | 42.49197 | 0.948317 | 26.4929 | 1.629707 | 42.18454 |
| Central Europe | 2016 | 0.777817 | 1.848884 | 41.84296 | 0.929885 | 25.84943 | 1.631888 | 42.20071 |
| Central Europe | 2017 | 0.781359 | 1.859103 | 41.82769 | 0.934059 | 25.83962 | 1.673516 | 43.21229 |
| Central Europe | 2018 | 0.785476 | 1.88286 | 42.3098 | 0.942761 | 25.94065 | 1.70054 | 43.74624 |
| Central Europe | 2019 | 0.789749 | 1.872725 | 41.90041 | 0.933556 | 25.64938 | 1.700256 | 43.58577 |
| Central Europe | 2020 | 0.793111 | 1.870164 | 41.90549 | 0.920216 | 25.02816 | 1.684527 | 42.78446 |
| Central Europe | 2021 | 0.796244 | 1.899653 | 42.70887 | 0.942396 | 25.65489 | 1.71598 | 43.70488 |
| Central Latin America | 1990 | 0.485787 | 0.715452 | 14.75314 | 0.315688 | 9.513592 | 0.686845 | 18.027 |
| Central Latin America | 1991 | 0.489559 | 0.726933 | 15.00184 | 0.320499 | 9.667769 | 0.662318 | 17.4345 |
| Central Latin America | 1992 | 0.49411 | 0.75797 | 15.92644 | 0.326542 | 9.865699 | 0.665768 | 17.63354 |
| Central Latin America | 1993 | 0.499418 | 0.76178 | 16.01208 | 0.329807 | 9.969782 | 0.650213 | 17.19928 |
| Central Latin America | 1994 | 0.505061 | 0.779259 | 16.47933 | 0.345405 | 10.48859 | 0.659321 | 17.37311 |
| Central Latin America | 1995 | 0.509874 | 0.794281 | 16.89494 | 0.368243 | 11.15654 | 0.641255 | 16.88216 |
| Central Latin America | 1996 | 0.514693 | 0.78216 | 16.50437 | 0.392287 | 11.81782 | 0.632387 | 16.62996 |
| Central Latin America | 1997 | 0.520171 | 0.77855 | 16.54642 | 0.400725 | 12.08844 | 0.666356 | 17.50752 |
| Central Latin America | 1998 | 0.526132 | 0.787914 | 16.93211 | 0.409569 | 12.3816 | 0.659431 | 17.47413 |
| Central Latin America | 1999 | 0.531807 | 0.787615 | 17.18265 | 0.408563 | 12.36465 | 0.642977 | 17.0457 |
| Central Latin America | 2000 | 0.5373 | 0.78897 | 17.30536 | 0.41661 | 12.57156 | 0.635391 | 16.73651 |
| Central Latin America | 2001 | 0.54244 | 0.794622 | 17.37775 | 0.427432 | 12.98847 | 0.627117 | 16.60356 |
| Central Latin America | 2002 | 0.547134 | 0.82566 | 18.21435 | 0.444345 | 13.48182 | 0.62741 | 16.63264 |
| Central Latin America | 2003 | 0.551446 | 0.842705 | 18.58226 | 0.45187 | 13.70098 | 0.622449 | 16.51527 |
| Central Latin America | 2004 | 0.556011 | 0.844553 | 18.75059 | 0.452902 | 13.72313 | 0.613536 | 16.2976 |
| Central Latin America | 2005 | 0.56075 | 0.855579 | 19.20319 | 0.472201 | 14.31544 | 0.609765 | 16.26574 |
| Central Latin America | 2006 | 0.565649 | 0.856328 | 19.18392 | 0.4867 | 14.77834 | 0.591556 | 15.71386 |
| Central Latin America | 2007 | 0.570707 | 0.861531 | 19.38232 | 0.491581 | 14.90634 | 0.57342 | 15.25255 |
| Central Latin America | 2008 | 0.575839 | 0.88681 | 20.06174 | 0.501262 | 15.26834 | 0.604576 | 16.211 |
| Central Latin America | 2009 | 0.57994 | 0.916394 | 20.66314 | 0.513305 | 15.66706 | 0.646994 | 17.4766 |
| Central Latin America | 2010 | 0.584253 | 0.922017 | 20.75807 | 0.511686 | 15.71825 | 0.658281 | 17.73995 |
| Central Latin America | 2011 | 0.589308 | 0.931884 | 21.11404 | 0.530027 | 16.33971 | 0.659651 | 17.84284 |
| Central Latin America | 2012 | 0.594827 | 0.957813 | 21.74391 | 0.538698 | 16.5915 | 0.683825 | 18.62719 |
| Central Latin America | 2013 | 0.600731 | 0.977182 | 22.1636 | 0.559345 | 17.25817 | 0.716939 | 19.62153 |
| Central Latin America | 2014 | 0.606509 | 0.997644 | 22.53176 | 0.576705 | 17.79944 | 0.75917 | 20.8589 |
| Central Latin America | 2015 | 0.611815 | 1.013289 | 22.91846 | 0.583711 | 18.01924 | 0.783908 | 21.5698 |
| Central Latin America | 2016 | 0.617086 | 1.056054 | 23.92219 | 0.605659 | 18.76707 | 0.816767 | 22.53381 |
| Central Latin America | 2017 | 0.622442 | 1.05835 | 23.90874 | 0.609613 | 18.89467 | 0.843716 | 23.34238 |
| Central Latin America | 2018 | 0.627765 | 1.078535 | 24.32163 | 0.618837 | 19.19321 | 0.861654 | 23.92234 |
| Central Latin America | 2019 | 0.632847 | 1.099898 | 24.79381 | 0.63893 | 19.88242 | 0.888641 | 24.6631 |
| Central Latin America | 2020 | 0.636996 | 1.115302 | 25.46985 | 0.651501 | 20.33109 | 0.893443 | 24.9456 |
| Central Latin America | 2021 | 0.640685 | 1.142939 | 26.15423 | 0.668125 | 20.87345 | 0.907051 | 25.35158 |
| Central Sub-Saharan Africa | 1990 | 0.302375 | 0.362978 | 7.987605 | 0.045271 | 1.348045 | 0.269334 | 7.400558 |
| Central Sub-Saharan Africa | 1991 | 0.305221 | 0.368351 | 8.105127 | 0.046939 | 1.400004 | 0.27335 | 7.51685 |
| Central Sub-Saharan Africa | 1992 | 0.307804 | 0.373249 | 8.228571 | 0.048408 | 1.443601 | 0.276004 | 7.581839 |
| Central Sub-Saharan Africa | 1993 | 0.309082 | 0.378929 | 8.368577 | 0.049916 | 1.48755 | 0.280104 | 7.689109 |
| Central Sub-Saharan Africa | 1994 | 0.309898 | 0.386102 | 8.537051 | 0.051772 | 1.543998 | 0.286259 | 7.857025 |
| Central Sub-Saharan Africa | 1995 | 0.311059 | 0.392994 | 8.700428 | 0.053705 | 1.602286 | 0.292341 | 8.024852 |
| Central Sub-Saharan Africa | 1996 | 0.312674 | 0.404456 | 9.007015 | 0.055641 | 1.658406 | 0.301861 | 8.281395 |
| Central Sub-Saharan Africa | 1997 | 0.314502 | 0.404192 | 9.019685 | 0.056951 | 1.696684 | 0.299758 | 8.204128 |
| Central Sub-Saharan Africa | 1998 | 0.316506 | 0.410398 | 9.14697 | 0.059116 | 1.763013 | 0.306132 | 8.381859 |
| Central Sub-Saharan Africa | 1999 | 0.318569 | 0.414895 | 9.211546 | 0.061508 | 1.838258 | 0.312072 | 8.553664 |
| Central Sub-Saharan Africa | 2000 | 0.320841 | 0.421125 | 9.352563 | 0.063673 | 1.903918 | 0.318385 | 8.720603 |
| Central Sub-Saharan Africa | 2001 | 0.323749 | 0.426486 | 9.489898 | 0.065664 | 1.963303 | 0.322939 | 8.830822 |
| Central Sub-Saharan Africa | 2002 | 0.327567 | 0.43406 | 9.669498 | 0.068414 | 2.046699 | 0.327757 | 8.950653 |
| Central Sub-Saharan Africa | 2003 | 0.331935 | 0.447772 | 9.993686 | 0.072019 | 2.158007 | 0.338024 | 9.233048 |
| Central Sub-Saharan Africa | 2004 | 0.33742 | 0.459673 | 10.2882 | 0.075354 | 2.258047 | 0.344628 | 9.403875 |
| Central Sub-Saharan Africa | 2005 | 0.344099 | 0.47107 | 10.57412 | 0.07878 | 2.360697 | 0.349621 | 9.527311 |
| Central Sub-Saharan Africa | 2006 | 0.35146 | 0.485563 | 10.91995 | 0.08278 | 2.48239 | 0.357799 | 9.742858 |
| Central Sub-Saharan Africa | 2007 | 0.359566 | 0.500707 | 11.29961 | 0.08688 | 2.606387 | 0.365141 | 9.934005 |
| Central Sub-Saharan Africa | 2008 | 0.368488 | 0.519081 | 11.73524 | 0.091495 | 2.74471 | 0.375248 | 10.20155 |
| Central Sub-Saharan Africa | 2009 | 0.376669 | 0.536607 | 12.15082 | 0.096386 | 2.892147 | 0.383786 | 10.42595 |
| Central Sub-Saharan Africa | 2010 | 0.385381 | 0.55703 | 12.62303 | 0.101879 | 3.056972 | 0.394169 | 10.70326 |
| Central Sub-Saharan Africa | 2011 | 0.394692 | 0.578026 | 13.11114 | 0.107472 | 3.225134 | 0.404502 | 10.98126 |
| Central Sub-Saharan Africa | 2012 | 0.404234 | 0.599931 | 13.62067 | 0.113361 | 3.402499 | 0.414726 | 11.25654 |
| Central Sub-Saharan Africa | 2013 | 0.413522 | 0.62398 | 14.16879 | 0.119871 | 3.596956 | 0.425703 | 11.55082 |
| Central Sub-Saharan Africa | 2014 | 0.422579 | 0.648489 | 14.7218 | 0.126733 | 3.804454 | 0.436451 | 11.84038 |
| Central Sub-Saharan Africa | 2015 | 0.430988 | 0.672767 | 15.25372 | 0.133498 | 4.005102 | 0.446842 | 12.11032 |
| Central Sub-Saharan Africa | 2016 | 0.438746 | 0.697907 | 15.82889 | 0.140733 | 4.22616 | 0.458207 | 12.42521 |
| Central Sub-Saharan Africa | 2017 | 0.446179 | 0.723775 | 16.40026 | 0.14852 | 4.463764 | 0.470263 | 12.75783 |
| Central Sub-Saharan Africa | 2018 | 0.45325 | 0.750847 | 17.00197 | 0.156624 | 4.711128 | 0.481955 | 13.07948 |
| Central Sub-Saharan Africa | 2019 | 0.46016 | 0.780298 | 17.65657 | 0.165267 | 4.972486 | 0.494271 | 13.41149 |
| Central Sub-Saharan Africa | 2020 | 0.466456 | 0.807605 | 18.24393 | 0.175244 | 5.276278 | 0.509239 | 13.82011 |
| Central Sub-Saharan Africa | 2021 | 0.472256 | 0.829415 | 18.71636 | 0.184041 | 5.547915 | 0.521345 | 14.15747 |
| East Asia | 1990 | 0.471179 | 0.255698 | 7.148393 | 0.030493 | 0.9714 | 0.268472 | 8.264593 |
| East Asia | 1991 | 0.479936 | 0.261139 | 7.280829 | 0.032624 | 1.036921 | 0.268597 | 8.25631 |
| East Asia | 1992 | 0.488431 | 0.268827 | 7.486576 | 0.035003 | 1.10932 | 0.271601 | 8.32985 |
| East Asia | 1993 | 0.496859 | 0.275833 | 7.674246 | 0.038057 | 1.202726 | 0.274526 | 8.412697 |
| East Asia | 1994 | 0.504886 | 0.284175 | 7.899099 | 0.040763 | 1.283852 | 0.278208 | 8.512132 |
| East Asia | 1995 | 0.51353 | 0.292669 | 8.126755 | 0.043801 | 1.376417 | 0.282002 | 8.609498 |
| East Asia | 1996 | 0.523758 | 0.299928 | 8.326817 | 0.046921 | 1.470037 | 0.288823 | 8.810591 |
| East Asia | 1997 | 0.533816 | 0.307443 | 8.53458 | 0.05026 | 1.567538 | 0.293193 | 8.930722 |
| East Asia | 1998 | 0.542653 | 0.316908 | 8.817413 | 0.053229 | 1.653111 | 0.300009 | 9.134484 |
| East Asia | 1999 | 0.551367 | 0.328475 | 9.15033 | 0.056437 | 1.749262 | 0.308108 | 9.382903 |
| East Asia | 2000 | 0.558816 | 0.337512 | 9.412545 | 0.059113 | 1.829577 | 0.314677 | 9.564554 |
| East Asia | 2001 | 0.564235 | 0.344619 | 9.63113 | 0.061451 | 1.899262 | 0.322358 | 9.764129 |
| East Asia | 2002 | 0.570798 | 0.346036 | 9.731118 | 0.062931 | 1.951194 | 0.326875 | 9.929231 |
| East Asia | 2003 | 0.578905 | 0.349393 | 9.859568 | 0.064606 | 2.010753 | 0.335639 | 10.2321 |
| East Asia | 2004 | 0.587683 | 0.357394 | 10.13099 | 0.067196 | 2.089207 | 0.345233 | 10.53665 |
| East Asia | 2005 | 0.597096 | 0.367155 | 10.43987 | 0.070682 | 2.194111 | 0.346905 | 10.61819 |
| East Asia | 2006 | 0.607273 | 0.36978 | 10.58348 | 0.073904 | 2.292795 | 0.338846 | 10.46325 |
| East Asia | 2007 | 0.617524 | 0.377056 | 10.81742 | 0.077941 | 2.416355 | 0.3379 | 10.47929 |
| East Asia | 2008 | 0.626956 | 0.382655 | 10.96817 | 0.08183 | 2.537832 | 0.340323 | 10.54854 |
| East Asia | 2009 | 0.636271 | 0.385532 | 11.02325 | 0.085903 | 2.66721 | 0.339071 | 10.51179 |
| East Asia | 2010 | 0.647985 | 0.39481 | 11.26598 | 0.091195 | 2.829974 | 0.336748 | 10.42919 |
| East Asia | 2011 | 0.657502 | 0.401496 | 11.42502 | 0.094932 | 2.941366 | 0.330228 | 10.22491 |
| East Asia | 2012 | 0.66293 | 0.402919 | 11.52652 | 0.097969 | 3.04354 | 0.317843 | 9.859419 |
| East Asia | 2013 | 0.668682 | 0.400663 | 11.52818 | 0.101071 | 3.150542 | 0.302741 | 9.411582 |
| East Asia | 2014 | 0.674063 | 0.402307 | 11.64073 | 0.105072 | 3.289812 | 0.286343 | 8.915597 |
| East Asia | 2015 | 0.677307 | 0.409056 | 11.82445 | 0.109737 | 3.426664 | 0.27972 | 8.688982 |
| East Asia | 2016 | 0.681528 | 0.421222 | 12.15598 | 0.11636 | 3.627879 | 0.280746 | 8.704143 |
| East Asia | 2017 | 0.689748 | 0.439036 | 12.73741 | 0.125771 | 3.923088 | 0.287369 | 8.937337 |
| East Asia | 2018 | 0.698626 | 0.453902 | 13.18411 | 0.132871 | 4.13911 | 0.296653 | 9.23231 |
| East Asia | 2019 | 0.708328 | 0.472877 | 13.72468 | 0.140627 | 4.372355 | 0.306654 | 9.542439 |
| East Asia | 2020 | 0.717709 | 0.489012 | 14.19163 | 0.149811 | 4.643312 | 0.317873 | 9.887396 |
| East Asia | 2021 | 0.725705 | 0.500839 | 14.54376 | 0.15666 | 4.858467 | 0.328417 | 10.2318 |
| Eastern Europe | 1990 | 0.66425 | 1.146191 | 27.00794 | 0.681508 | 21.362 | 1.64204 | 48.11663 |
| Eastern Europe | 1991 | 0.671452 | 1.190084 | 28.07564 | 0.697934 | 21.86306 | 1.672754 | 48.81661 |
| Eastern Europe | 1992 | 0.678753 | 1.252225 | 29.43403 | 0.717626 | 22.4476 | 1.706123 | 49.86661 |
| Eastern Europe | 1993 | 0.683359 | 1.360191 | 32.01668 | 0.755723 | 23.67724 | 1.829973 | 53.93384 |
| Eastern Europe | 1994 | 0.686012 | 1.425897 | 33.92466 | 0.774735 | 24.31611 | 1.898951 | 56.16707 |
| Eastern Europe | 1995 | 0.689459 | 1.430699 | 34.09855 | 0.796997 | 25.02005 | 1.87317 | 55.24894 |
| Eastern Europe | 1996 | 0.692791 | 1.431144 | 34.24035 | 0.77724 | 24.32802 | 1.839681 | 54.03526 |
| Eastern Europe | 1997 | 0.695203 | 1.45992 | 35.02021 | 0.765348 | 23.87241 | 1.81005 | 52.58787 |
| Eastern Europe | 1998 | 0.69694 | 1.496747 | 36.08327 | 0.75075 | 23.26812 | 1.790564 | 51.78375 |
| Eastern Europe | 1999 | 0.698814 | 1.598357 | 38.75501 | 0.742381 | 23.03048 | 1.839863 | 53.44605 |
| Eastern Europe | 2000 | 0.701011 | 1.653269 | 40.18752 | 0.806426 | 24.94028 | 1.808139 | 52.24575 |
| Eastern Europe | 2001 | 0.703506 | 1.676005 | 40.85719 | 0.820112 | 25.28309 | 1.791193 | 51.85215 |
| Eastern Europe | 2002 | 0.706727 | 1.703975 | 41.68728 | 0.840562 | 25.99144 | 1.800122 | 52.07433 |
| Eastern Europe | 2003 | 0.712025 | 1.743053 | 42.78643 | 0.84732 | 26.17448 | 1.811249 | 52.51082 |
| Eastern Europe | 2004 | 0.719215 | 1.73918 | 42.76356 | 0.848017 | 26.11708 | 1.771354 | 51.77376 |
| Eastern Europe | 2005 | 0.726266 | 1.745331 | 42.8905 | 0.898832 | 27.67606 | 1.707659 | 49.95678 |
| Eastern Europe | 2006 | 0.732617 | 1.684554 | 41.19585 | 0.887718 | 27.05146 | 1.585881 | 46.05281 |
| Eastern Europe | 2007 | 0.738787 | 1.660398 | 40.2366 | 0.894538 | 26.89904 | 1.447809 | 41.43518 |
| Eastern Europe | 2008 | 0.74545 | 1.698022 | 41.06165 | 0.918607 | 27.59337 | 1.463306 | 41.78301 |
| Eastern Europe | 2009 | 0.75107 | 1.697529 | 41.04188 | 0.913531 | 27.29255 | 1.45413 | 41.28162 |
| Eastern Europe | 2010 | 0.756606 | 1.732377 | 41.80746 | 0.940521 | 28.08454 | 1.502299 | 42.75664 |
| Eastern Europe | 2011 | 0.761025 | 1.697788 | 40.89956 | 0.912682 | 27.38367 | 1.563562 | 44.84223 |
| Eastern Europe | 2012 | 0.765423 | 1.690947 | 40.6956 | 0.912128 | 27.39791 | 1.643947 | 47.43523 |
| Eastern Europe | 2013 | 0.770338 | 1.696049 | 40.6726 | 0.914207 | 27.39786 | 1.793692 | 51.763 |
| Eastern Europe | 2014 | 0.775092 | 1.688491 | 40.32224 | 0.914219 | 27.2988 | 1.74416 | 50.22842 |
| Eastern Europe | 2015 | 0.779663 | 1.681557 | 39.99537 | 0.909922 | 27.0419 | 1.870578 | 53.66031 |
| Eastern Europe | 2016 | 0.784805 | 1.671104 | 39.7669 | 0.895923 | 26.61246 | 1.908752 | 54.53896 |
| Eastern Europe | 2017 | 0.790015 | 1.633728 | 38.99067 | 0.872331 | 26.0394 | 1.94468 | 55.90899 |
| Eastern Europe | 2018 | 0.794486 | 1.659145 | 39.59159 | 0.870303 | 25.96449 | 2.017316 | 57.99364 |
| Eastern Europe | 2019 | 0.798138 | 1.655915 | 39.3581 | 0.870633 | 25.94888 | 2.038678 | 58.68558 |
| Eastern Europe | 2020 | 0.800505 | 1.64084 | 39.09109 | 0.863145 | 25.68821 | 2.01557 | 58.01059 |
| Eastern Europe | 2021 | 0.802851 | 1.717464 | 41.12157 | 0.892477 | 26.4754 | 2.095328 | 60.21525 |
| Eastern Sub-Saharan Africa | 1990 | 0.233622 | 0.349778 | 6.927769 | 0.077406 | 2.48347 | 0.281711 | 7.817894 |
| Eastern Sub-Saharan Africa | 1991 | 0.23634 | 0.355337 | 7.03318 | 0.080391 | 2.5808 | 0.285296 | 7.926991 |
| Eastern Sub-Saharan Africa | 1992 | 0.238615 | 0.361085 | 7.15268 | 0.083212 | 2.670077 | 0.288847 | 8.026336 |
| Eastern Sub-Saharan Africa | 1993 | 0.240817 | 0.368118 | 7.293009 | 0.086504 | 2.775684 | 0.29358 | 8.163112 |
| Eastern Sub-Saharan Africa | 1994 | 0.242902 | 0.374743 | 7.427687 | 0.089905 | 2.885181 | 0.298367 | 8.302093 |
| Eastern Sub-Saharan Africa | 1995 | 0.245424 | 0.379602 | 7.516279 | 0.092926 | 2.982966 | 0.302199 | 8.408965 |
| Eastern Sub-Saharan Africa | 1996 | 0.248585 | 0.385946 | 7.645525 | 0.096092 | 3.080815 | 0.306097 | 8.512172 |
| Eastern Sub-Saharan Africa | 1997 | 0.252145 | 0.394334 | 7.83634 | 0.099419 | 3.18364 | 0.310479 | 8.62844 |
| Eastern Sub-Saharan Africa | 1998 | 0.255891 | 0.403324 | 8.03595 | 0.103191 | 3.30046 | 0.315248 | 8.757572 |
| Eastern Sub-Saharan Africa | 1999 | 0.259957 | 0.410112 | 8.19658 | 0.106537 | 3.400058 | 0.317694 | 8.809693 |
| Eastern Sub-Saharan Africa | 2000 | 0.264165 | 0.418428 | 8.380539 | 0.110543 | 3.521873 | 0.320828 | 8.879583 |
| Eastern Sub-Saharan Africa | 2001 | 0.268772 | 0.423789 | 8.504438 | 0.113797 | 3.62059 | 0.31989 | 8.841929 |
| Eastern Sub-Saharan Africa | 2002 | 0.273484 | 0.432543 | 8.683962 | 0.118164 | 3.749689 | 0.322493 | 8.887962 |
| Eastern Sub-Saharan Africa | 2003 | 0.278434 | 0.442955 | 8.891496 | 0.123585 | 3.915604 | 0.327305 | 9.015084 |
| Eastern Sub-Saharan Africa | 2004 | 0.283937 | 0.454332 | 9.125436 | 0.128977 | 4.078335 | 0.332371 | 9.141517 |
| Eastern Sub-Saharan Africa | 2005 | 0.28997 | 0.464551 | 9.34255 | 0.134006 | 4.229632 | 0.336187 | 9.230609 |
| Eastern Sub-Saharan Africa | 2006 | 0.296455 | 0.473443 | 9.514594 | 0.138638 | 4.370423 | 0.339635 | 9.307625 |
| Eastern Sub-Saharan Africa | 2007 | 0.303576 | 0.481263 | 9.662623 | 0.143725 | 4.530013 | 0.343468 | 9.409466 |
| Eastern Sub-Saharan Africa | 2008 | 0.310918 | 0.492215 | 9.890064 | 0.148749 | 4.683906 | 0.347616 | 9.507335 |
| Eastern Sub-Saharan Africa | 2009 | 0.318457 | 0.505551 | 10.17273 | 0.154521 | 4.862909 | 0.353862 | 9.668483 |
| Eastern Sub-Saharan Africa | 2010 | 0.326296 | 0.52479 | 10.59065 | 0.161145 | 5.061057 | 0.362067 | 9.87455 |
| Eastern Sub-Saharan Africa | 2011 | 0.334253 | 0.539075 | 10.87895 | 0.167283 | 5.253125 | 0.368129 | 10.0319 |
| Eastern Sub-Saharan Africa | 2012 | 0.341628 | 0.555857 | 11.23272 | 0.173604 | 5.447206 | 0.374667 | 10.2015 |
| Eastern Sub-Saharan Africa | 2013 | 0.349207 | 0.575043 | 11.61769 | 0.180447 | 5.658899 | 0.382854 | 10.41595 |
| Eastern Sub-Saharan Africa | 2014 | 0.35701 | 0.594102 | 11.99993 | 0.188181 | 5.898621 | 0.391618 | 10.65093 |
| Eastern Sub-Saharan Africa | 2015 | 0.364907 | 0.614016 | 12.41403 | 0.196402 | 6.145864 | 0.400435 | 10.87584 |
| Eastern Sub-Saharan Africa | 2016 | 0.372759 | 0.633887 | 12.84316 | 0.204564 | 6.40274 | 0.408305 | 11.09617 |
| Eastern Sub-Saharan Africa | 2017 | 0.3805 | 0.655223 | 13.29179 | 0.212898 | 6.663181 | 0.416401 | 11.31389 |
| Eastern Sub-Saharan Africa | 2018 | 0.388355 | 0.676778 | 13.74487 | 0.221961 | 6.948886 | 0.423858 | 11.51456 |
| Eastern Sub-Saharan Africa | 2019 | 0.396345 | 0.698877 | 14.20401 | 0.231171 | 7.234805 | 0.431315 | 11.70989 |
| Eastern Sub-Saharan Africa | 2020 | 0.40348 | 0.718011 | 14.60202 | 0.241139 | 7.54216 | 0.439936 | 11.93807 |
| Eastern Sub-Saharan Africa | 2021 | 0.409721 | 0.733245 | 14.96838 | 0.250461 | 7.841994 | 0.447041 | 12.14956 |
| Global | 1990 | 0.525529 | 0.91385 | 20.55137 | 0.322893 | 8.72275 | 0.660644 | 17.26387 |
| Global | 1991 | 0.530272 | 0.921705 | 20.73263 | 0.326471 | 8.809162 | 0.661637 | 17.29493 |
| Global | 1992 | 0.534637 | 0.930661 | 20.92245 | 0.330584 | 8.910769 | 0.663519 | 17.34919 |
| Global | 1993 | 0.538598 | 0.945984 | 21.29261 | 0.335527 | 9.045891 | 0.667658 | 17.50911 |
| Global | 1994 | 0.542389 | 0.951773 | 21.47373 | 0.338998 | 9.14166 | 0.670354 | 17.61143 |
| Global | 1995 | 0.546328 | 0.950975 | 21.47718 | 0.344149 | 9.287902 | 0.664586 | 17.45533 |
| Global | 1996 | 0.550703 | 0.947024 | 21.37703 | 0.343979 | 9.266957 | 0.663259 | 17.41508 |
| Global | 1997 | 0.555139 | 0.943051 | 21.34919 | 0.345331 | 9.293251 | 0.662679 | 17.38487 |
| Global | 1998 | 0.559381 | 0.943358 | 21.40355 | 0.345581 | 9.286481 | 0.66147 | 17.35069 |
| Global | 1999 | 0.563738 | 0.951087 | 21.63441 | 0.347041 | 9.32663 | 0.66401 | 17.46379 |
| Global | 2000 | 0.568003 | 0.953946 | 21.78304 | 0.355239 | 9.550402 | 0.660992 | 17.40224 |
| Global | 2001 | 0.571778 | 0.953144 | 21.80346 | 0.35884 | 9.654963 | 0.659955 | 17.40806 |
| Global | 2002 | 0.575688 | 0.954276 | 21.85486 | 0.362445 | 9.767298 | 0.661623 | 17.4705 |
| Global | 2003 | 0.579907 | 0.956034 | 21.95368 | 0.364934 | 9.837616 | 0.66793 | 17.67445 |
| Global | 2004 | 0.584379 | 0.948149 | 21.85829 | 0.365398 | 9.845304 | 0.663475 | 17.61735 |
| Global | 2005 | 0.588979 | 0.94522 | 21.8577 | 0.366374 | 9.908299 | 0.657208 | 17.51817 |
| Global | 2006 | 0.593757 | 0.938275 | 21.73243 | 0.366453 | 9.899998 | 0.645654 | 17.2146 |
| Global | 2007 | 0.598641 | 0.935766 | 21.70443 | 0.366358 | 9.881783 | 0.636256 | 16.95962 |
| Global | 2008 | 0.60337 | 0.940033 | 21.83499 | 0.367723 | 9.9367 | 0.639723 | 17.0613 |
| Global | 2009 | 0.607759 | 0.938955 | 21.85068 | 0.368893 | 9.989267 | 0.640709 | 17.11234 |
| Global | 2010 | 0.612952 | 0.940485 | 21.93322 | 0.364592 | 9.918163 | 0.647901 | 17.31684 |
| Global | 2011 | 0.617759 | 0.938698 | 21.90251 | 0.364971 | 9.948282 | 0.653771 | 17.49924 |
| Global | 2012 | 0.621845 | 0.936681 | 21.82812 | 0.364261 | 9.947511 | 0.659232 | 17.65364 |
| Global | 2013 | 0.626343 | 0.933351 | 21.70154 | 0.364972 | 9.984196 | 0.668622 | 17.91264 |
| Global | 2014 | 0.630982 | 0.932453 | 21.63984 | 0.366194 | 10.04017 | 0.667342 | 17.86078 |
| Global | 2015 | 0.635441 | 0.938868 | 21.73117 | 0.363701 | 10.0027 | 0.681691 | 18.21055 |
| Global | 2016 | 0.640154 | 0.942391 | 21.74393 | 0.367146 | 10.12284 | 0.692755 | 18.51056 |
| Global | 2017 | 0.645564 | 0.94217 | 21.70381 | 0.368759 | 10.19948 | 0.701798 | 18.77105 |
| Global | 2018 | 0.651048 | 0.947437 | 21.7968 | 0.370328 | 10.27448 | 0.710199 | 19.00425 |
| Global | 2019 | 0.656576 | 0.947494 | 21.79401 | 0.371725 | 10.3386 | 0.714103 | 19.14003 |
| Global | 2020 | 0.661344 | 0.941056 | 21.63061 | 0.371988 | 10.36079 | 0.708689 | 18.99795 |
| Global | 2021 | 0.665821 | 0.946927 | 21.82608 | 0.377922 | 10.56047 | 0.715446 | 19.23463 |
| High-income Asia Pacific | 1990 | 0.767804 | 0.240322 | 7.370859 | 0.074941 | 2.288321 | 0.264475 | 6.898589 |
| High-income Asia Pacific | 1991 | 0.773333 | 0.249343 | 7.622995 | 0.07613 | 2.317767 | 0.262341 | 6.869538 |
| High-income Asia Pacific | 1992 | 0.778834 | 0.254921 | 7.791256 | 0.077472 | 2.361222 | 0.258131 | 6.812814 |
| High-income Asia Pacific | 1993 | 0.783967 | 0.261635 | 7.978593 | 0.078253 | 2.383134 | 0.248308 | 6.553863 |
| High-income Asia Pacific | 1994 | 0.789028 | 0.270271 | 8.239885 | 0.08106 | 2.446456 | 0.250652 | 6.63686 |
| High-income Asia Pacific | 1995 | 0.794099 | 0.281802 | 8.629101 | 0.083669 | 2.507918 | 0.251799 | 6.668663 |
| High-income Asia Pacific | 1996 | 0.798863 | 0.286512 | 8.787342 | 0.084459 | 2.53185 | 0.251718 | 6.72209 |
| High-income Asia Pacific | 1997 | 0.803128 | 0.291644 | 8.967567 | 0.085383 | 2.559304 | 0.247433 | 6.59607 |
| High-income Asia Pacific | 1998 | 0.806831 | 0.297868 | 9.143119 | 0.084295 | 2.512495 | 0.246546 | 6.568118 |
| High-income Asia Pacific | 1999 | 0.810469 | 0.306938 | 9.393943 | 0.082707 | 2.462081 | 0.248349 | 6.63881 |
| High-income Asia Pacific | 2000 | 0.814089 | 0.314157 | 9.629373 | 0.082817 | 2.470987 | 0.245276 | 6.640662 |
| High-income Asia Pacific | 2001 | 0.817545 | 0.322387 | 9.903455 | 0.083335 | 2.492871 | 0.24474 | 6.655904 |
| High-income Asia Pacific | 2002 | 0.82111 | 0.323421 | 9.938009 | 0.083446 | 2.48869 | 0.242905 | 6.609311 |
| High-income Asia Pacific | 2003 | 0.824636 | 0.328445 | 10.11095 | 0.08496 | 2.53769 | 0.24365 | 6.672244 |
| High-income Asia Pacific | 2004 | 0.828074 | 0.342069 | 10.49722 | 0.087578 | 2.623035 | 0.247444 | 6.848505 |
| High-income Asia Pacific | 2005 | 0.831062 | 0.347239 | 10.64137 | 0.087963 | 2.630359 | 0.246061 | 6.884081 |
| High-income Asia Pacific | 2006 | 0.833766 | 0.355585 | 10.89464 | 0.088414 | 2.642572 | 0.247167 | 6.939422 |
| High-income Asia Pacific | 2007 | 0.836546 | 0.358173 | 10.97271 | 0.089238 | 2.668094 | 0.250573 | 7.003832 |
| High-income Asia Pacific | 2008 | 0.83928 | 0.364506 | 11.14601 | 0.090494 | 2.709435 | 0.251298 | 7.050835 |
| High-income Asia Pacific | 2009 | 0.841771 | 0.367909 | 11.21445 | 0.092213 | 2.768171 | 0.247784 | 7.007643 |
| High-income Asia Pacific | 2010 | 0.844682 | 0.377391 | 11.46218 | 0.092704 | 2.779261 | 0.258542 | 7.322388 |
| High-income Asia Pacific | 2011 | 0.847722 | 0.385999 | 11.66487 | 0.09523 | 2.851854 | 0.270357 | 7.672759 |
| High-income Asia Pacific | 2012 | 0.850693 | 0.382559 | 11.51735 | 0.096362 | 2.890938 | 0.272884 | 7.773794 |
| High-income Asia Pacific | 2013 | 0.853671 | 0.390205 | 11.74098 | 0.098038 | 2.925206 | 0.275402 | 7.883057 |
| High-income Asia Pacific | 2014 | 0.856612 | 0.394801 | 11.84293 | 0.100702 | 2.997531 | 0.284784 | 8.198309 |
| High-income Asia Pacific | 2015 | 0.859603 | 0.398222 | 11.89703 | 0.100475 | 2.989861 | 0.290753 | 8.355541 |
| High-income Asia Pacific | 2016 | 0.862733 | 0.409959 | 12.19457 | 0.103769 | 3.088055 | 0.296425 | 8.495649 |
| High-income Asia Pacific | 2017 | 0.86587 | 0.412805 | 12.24232 | 0.105877 | 3.153995 | 0.302315 | 8.710131 |
| High-income Asia Pacific | 2018 | 0.869073 | 0.420119 | 12.4256 | 0.109475 | 3.249427 | 0.309013 | 8.879364 |
| High-income Asia Pacific | 2019 | 0.872219 | 0.424493 | 12.53067 | 0.11285 | 3.347245 | 0.312819 | 9.031003 |
| High-income Asia Pacific | 2020 | 0.874437 | 0.414716 | 12.30134 | 0.113803 | 3.397531 | 0.307498 | 8.939206 |
| High-income Asia Pacific | 2021 | 0.876767 | 0.422815 | 12.46707 | 0.118279 | 3.50219 | 0.3147 | 9.072241 |
| High-income North America | 1990 | 0.76566 | 2.034169 | 49.85758 | 0.833799 | 23.35711 | 1.052565 | 28.14386 |
| High-income North America | 1991 | 0.76805 | 2.046753 | 50.15313 | 0.851293 | 23.77192 | 1.047064 | 27.99574 |
| High-income North America | 1992 | 0.77155 | 2.038932 | 49.95411 | 0.857723 | 23.8343 | 1.052967 | 28.09429 |
| High-income North America | 1993 | 0.774978 | 2.063066 | 50.53019 | 0.859413 | 23.79113 | 1.071469 | 28.65261 |
| High-income North America | 1994 | 0.778207 | 2.067311 | 50.60152 | 0.877366 | 24.20404 | 1.085448 | 29.08037 |
| High-income North America | 1995 | 0.781348 | 2.057532 | 50.49908 | 0.885504 | 24.38402 | 1.090676 | 29.31577 |
| High-income North America | 1996 | 0.784105 | 2.023669 | 49.65904 | 0.880409 | 24.14977 | 1.090921 | 29.39716 |
| High-income North America | 1997 | 0.786348 | 1.981 | 48.56658 | 0.887486 | 24.24845 | 1.087589 | 29.41027 |
| High-income North America | 1998 | 0.788569 | 1.955125 | 47.80686 | 0.891801 | 24.27404 | 1.100488 | 29.78389 |
| High-income North America | 1999 | 0.791196 | 1.965169 | 47.95607 | 0.919386 | 24.96927 | 1.124497 | 30.61522 |
| High-income North America | 2000 | 0.794997 | 1.951502 | 47.54105 | 0.939475 | 25.41418 | 1.143361 | 31.3884 |
| High-income North America | 2001 | 0.799275 | 1.939541 | 47.24969 | 0.958393 | 25.9054 | 1.163695 | 32.01771 |
| High-income North America | 2002 | 0.802961 | 1.929714 | 46.91156 | 0.971071 | 26.21531 | 1.177243 | 32.45355 |
| High-income North America | 2003 | 0.805821 | 1.915494 | 46.56791 | 0.97689 | 26.24003 | 1.180293 | 32.56367 |
| High-income North America | 2004 | 0.807991 | 1.881717 | 45.82871 | 0.981585 | 26.25324 | 1.176363 | 32.4744 |
| High-income North America | 2005 | 0.808887 | 1.85601 | 45.17467 | 0.960352 | 25.64491 | 1.187789 | 33.00862 |
| High-income North America | 2006 | 0.809721 | 1.83568 | 44.63687 | 0.958632 | 25.49348 | 1.207884 | 33.60637 |
| High-income North America | 2007 | 0.812486 | 1.806127 | 43.87115 | 0.93758 | 24.7875 | 1.211857 | 33.86085 |
| High-income North America | 2008 | 0.817084 | 1.775512 | 43.041 | 0.922758 | 24.46205 | 1.217603 | 34.15251 |
| High-income North America | 2009 | 0.82247 | 1.759476 | 42.62167 | 0.925054 | 24.54269 | 1.234828 | 34.78267 |
| High-income North America | 2010 | 0.827814 | 1.740577 | 42.32108 | 0.884916 | 23.4341 | 1.274193 | 35.94342 |
| High-income North America | 2011 | 0.832061 | 1.729192 | 41.93702 | 0.878524 | 23.23655 | 1.304629 | 36.92576 |
| High-income North America | 2012 | 0.835669 | 1.700924 | 41.0351 | 0.86672 | 22.88393 | 1.323389 | 37.384 |
| High-income North America | 2013 | 0.839059 | 1.671248 | 40.252 | 0.851851 | 22.45603 | 1.348914 | 38.27336 |
| High-income North America | 2014 | 0.842341 | 1.667244 | 39.94695 | 0.848543 | 22.39154 | 1.388746 | 39.36706 |
| High-income North America | 2015 | 0.845877 | 1.651071 | 39.51758 | 0.819173 | 21.65986 | 1.427764 | 40.60117 |
| High-income North America | 2016 | 0.849423 | 1.646312 | 39.08574 | 0.825772 | 21.87097 | 1.507981 | 43.04724 |
| High-income North America | 2017 | 0.85296 | 1.637774 | 38.71929 | 0.815144 | 21.59757 | 1.538765 | 43.88255 |
| High-income North America | 2018 | 0.856316 | 1.618714 | 38.07182 | 0.790814 | 21.02894 | 1.54841 | 44.03283 |
| High-income North America | 2019 | 0.859749 | 1.593687 | 37.37723 | 0.770975 | 20.41404 | 1.562194 | 44.38679 |
| High-income North America | 2020 | 0.861874 | 1.5808 | 37.07922 | 0.765926 | 20.27864 | 1.548449 | 44.0093 |
| High-income North America | 2021 | 0.863465 | 1.610024 | 37.8732 | 0.775689 | 20.4547 | 1.570366 | 44.47584 |
| North Africa and Middle East | 1990 | 0.437421 | 0.41217 | 9.002148 | 0.244118 | 7.266033 | 0.492262 | 13.51944 |
| North Africa and Middle East | 1990 | 0.437421 | 0.41217 | 9.002148 | 0.244118 | 7.266033 | 0.492262 | 13.51944 |
| North Africa and Middle East | 1990 | 0.437421 | 0.41217 | 9.002148 | 0.244118 | 7.266033 | 0.492262 | 13.51944 |
| North Africa and Middle East | 1990 | 0.437421 | 0.41217 | 9.002148 | 0.244118 | 7.266033 | 0.492262 | 13.51944 |
| North Africa and Middle East | 1991 | 0.445921 | 0.424176 | 9.374134 | 0.25086 | 7.494755 | 0.491748 | 13.67772 |
| North Africa and Middle East | 1991 | 0.445921 | 0.424176 | 9.374134 | 0.25086 | 7.494755 | 0.491748 | 13.67772 |
| North Africa and Middle East | 1991 | 0.445921 | 0.424176 | 9.374134 | 0.25086 | 7.494755 | 0.491748 | 13.67772 |
| North Africa and Middle East | 1991 | 0.445921 | 0.424176 | 9.374134 | 0.25086 | 7.494755 | 0.491748 | 13.67772 |
| North Africa and Middle East | 1992 | 0.453971 | 0.433376 | 9.610672 | 0.257063 | 7.682021 | 0.490783 | 13.69962 |
| North Africa and Middle East | 1992 | 0.453971 | 0.433376 | 9.610672 | 0.257063 | 7.682021 | 0.490783 | 13.69962 |
| North Africa and Middle East | 1992 | 0.453971 | 0.433376 | 9.610672 | 0.257063 | 7.682021 | 0.490783 | 13.69962 |
| North Africa and Middle East | 1992 | 0.453971 | 0.433376 | 9.610672 | 0.257063 | 7.682021 | 0.490783 | 13.69962 |
| North Africa and Middle East | 1993 | 0.462102 | 0.444415 | 9.871649 | 0.263449 | 7.873697 | 0.493282 | 13.78923 |
| North Africa and Middle East | 1993 | 0.462102 | 0.444415 | 9.871649 | 0.263449 | 7.873697 | 0.493282 | 13.78923 |
| North Africa and Middle East | 1993 | 0.462102 | 0.444415 | 9.871649 | 0.263449 | 7.873697 | 0.493282 | 13.78923 |
| North Africa and Middle East | 1993 | 0.462102 | 0.444415 | 9.871649 | 0.263449 | 7.873697 | 0.493282 | 13.78923 |
| North Africa and Middle East | 1994 | 0.470394 | 0.453275 | 10.08632 | 0.269148 | 8.046919 | 0.494647 | 13.83802 |
| North Africa and Middle East | 1994 | 0.470394 | 0.453275 | 10.08632 | 0.269148 | 8.046919 | 0.494647 | 13.83802 |
| North Africa and Middle East | 1994 | 0.470394 | 0.453275 | 10.08632 | 0.269148 | 8.046919 | 0.494647 | 13.83802 |
| North Africa and Middle East | 1994 | 0.470394 | 0.453275 | 10.08632 | 0.269148 | 8.046919 | 0.494647 | 13.83802 |
| North Africa and Middle East | 1995 | 0.478553 | 0.462301 | 10.30815 | 0.275516 | 8.228471 | 0.493661 | 13.80019 |
| North Africa and Middle East | 1995 | 0.478553 | 0.462301 | 10.30815 | 0.275516 | 8.228471 | 0.493661 | 13.80019 |
| North Africa and Middle East | 1995 | 0.478553 | 0.462301 | 10.30815 | 0.275516 | 8.228471 | 0.493661 | 13.80019 |
| North Africa and Middle East | 1995 | 0.478553 | 0.462301 | 10.30815 | 0.275516 | 8.228471 | 0.493661 | 13.80019 |
| North Africa and Middle East | 1996 | 0.486463 | 0.470882 | 10.50996 | 0.280605 | 8.378548 | 0.494147 | 13.82129 |
| North Africa and Middle East | 1996 | 0.486463 | 0.470882 | 10.50996 | 0.280605 | 8.378548 | 0.494147 | 13.82129 |
| North Africa and Middle East | 1996 | 0.486463 | 0.470882 | 10.50996 | 0.280605 | 8.378548 | 0.494147 | 13.82129 |
| North Africa and Middle East | 1996 | 0.486463 | 0.470882 | 10.50996 | 0.280605 | 8.378548 | 0.494147 | 13.82129 |
| North Africa and Middle East | 1997 | 0.49398 | 0.479548 | 10.72657 | 0.285683 | 8.52871 | 0.494551 | 13.83635 |
| North Africa and Middle East | 1997 | 0.49398 | 0.479548 | 10.72657 | 0.285683 | 8.52871 | 0.494551 | 13.83635 |
| North Africa and Middle East | 1997 | 0.49398 | 0.479548 | 10.72657 | 0.285683 | 8.52871 | 0.494551 | 13.83635 |
| North Africa and Middle East | 1997 | 0.49398 | 0.479548 | 10.72657 | 0.285683 | 8.52871 | 0.494551 | 13.83635 |
| North Africa and Middle East | 1998 | 0.501445 | 0.488357 | 10.96604 | 0.29056 | 8.678812 | 0.493799 | 13.82125 |
| North Africa and Middle East | 1998 | 0.501445 | 0.488357 | 10.96604 | 0.29056 | 8.678812 | 0.493799 | 13.82125 |
| North Africa and Middle East | 1998 | 0.501445 | 0.488357 | 10.96604 | 0.29056 | 8.678812 | 0.493799 | 13.82125 |
| North Africa and Middle East | 1998 | 0.501445 | 0.488357 | 10.96604 | 0.29056 | 8.678812 | 0.493799 | 13.82125 |
| North Africa and Middle East | 1999 | 0.508956 | 0.494878 | 11.13674 | 0.295121 | 8.827371 | 0.489936 | 13.7392 |
| North Africa and Middle East | 1999 | 0.508956 | 0.494878 | 11.13674 | 0.295121 | 8.827371 | 0.489936 | 13.7392 |
| North Africa and Middle East | 1999 | 0.508956 | 0.494878 | 11.13674 | 0.295121 | 8.827371 | 0.489936 | 13.7392 |
| North Africa and Middle East | 1999 | 0.508956 | 0.494878 | 11.13674 | 0.295121 | 8.827371 | 0.489936 | 13.7392 |
| North Africa and Middle East | 2000 | 0.516824 | 0.502343 | 11.35567 | 0.301237 | 8.983937 | 0.481693 | 13.46137 |
| North Africa and Middle East | 2000 | 0.516824 | 0.502343 | 11.35567 | 0.301237 | 8.983937 | 0.481693 | 13.46137 |
| North Africa and Middle East | 2000 | 0.516824 | 0.502343 | 11.35567 | 0.301237 | 8.983937 | 0.481693 | 13.46137 |
| North Africa and Middle East | 2000 | 0.516824 | 0.502343 | 11.35567 | 0.301237 | 8.983937 | 0.481693 | 13.46137 |
| North Africa and Middle East | 2001 | 0.524091 | 0.516061 | 11.72774 | 0.307124 | 9.14469 | 0.48164 | 13.45582 |
| North Africa and Middle East | 2001 | 0.524091 | 0.516061 | 11.72774 | 0.307124 | 9.14469 | 0.48164 | 13.45582 |
| North Africa and Middle East | 2001 | 0.524091 | 0.516061 | 11.72774 | 0.307124 | 9.14469 | 0.48164 | 13.45582 |
| North Africa and Middle East | 2001 | 0.524091 | 0.516061 | 11.72774 | 0.307124 | 9.14469 | 0.48164 | 13.45582 |
| North Africa and Middle East | 2002 | 0.531149 | 0.546207 | 12.50257 | 0.317136 | 9.42883 | 0.480159 | 13.42182 |
| North Africa and Middle East | 2002 | 0.531149 | 0.546207 | 12.50257 | 0.317136 | 9.42883 | 0.480159 | 13.42182 |
| North Africa and Middle East | 2002 | 0.531149 | 0.546207 | 12.50257 | 0.317136 | 9.42883 | 0.480159 | 13.42182 |
| North Africa and Middle East | 2002 | 0.531149 | 0.546207 | 12.50257 | 0.317136 | 9.42883 | 0.480159 | 13.42182 |
| North Africa and Middle East | 2003 | 0.538292 | 0.611318 | 14.09311 | 0.327235 | 9.708164 | 0.477349 | 13.35193 |
| North Africa and Middle East | 2003 | 0.538292 | 0.611318 | 14.09311 | 0.327235 | 9.708164 | 0.477349 | 13.35193 |
| North Africa and Middle East | 2003 | 0.538292 | 0.611318 | 14.09311 | 0.327235 | 9.708164 | 0.477349 | 13.35193 |
| North Africa and Middle East | 2003 | 0.538292 | 0.611318 | 14.09311 | 0.327235 | 9.708164 | 0.477349 | 13.35193 |
| North Africa and Middle East | 2004 | 0.545678 | 0.667448 | 15.39966 | 0.337966 | 9.994159 | 0.474992 | 13.26552 |
| North Africa and Middle East | 2004 | 0.545678 | 0.667448 | 15.39966 | 0.337966 | 9.994159 | 0.474992 | 13.26552 |
| North Africa and Middle East | 2004 | 0.545678 | 0.667448 | 15.39966 | 0.337966 | 9.994159 | 0.474992 | 13.26552 |
| North Africa and Middle East | 2004 | 0.545678 | 0.667448 | 15.39966 | 0.337966 | 9.994159 | 0.474992 | 13.26552 |
| North Africa and Middle East | 2005 | 0.553047 | 0.722659 | 16.67418 | 0.350232 | 10.32403 | 0.477295 | 13.30572 |
| North Africa and Middle East | 2005 | 0.553047 | 0.722659 | 16.67418 | 0.350232 | 10.32403 | 0.477295 | 13.30572 |
| North Africa and Middle East | 2005 | 0.553047 | 0.722659 | 16.67418 | 0.350232 | 10.32403 | 0.477295 | 13.30572 |
| North Africa and Middle East | 2005 | 0.553047 | 0.722659 | 16.67418 | 0.350232 | 10.32403 | 0.477295 | 13.30572 |
| North Africa and Middle East | 2006 | 0.560089 | 0.76708 | 17.7006 | 0.358933 | 10.5755 | 0.478462 | 13.33762 |
| North Africa and Middle East | 2006 | 0.560089 | 0.76708 | 17.7006 | 0.358933 | 10.5755 | 0.478462 | 13.33762 |
| North Africa and Middle East | 2006 | 0.560089 | 0.76708 | 17.7006 | 0.358933 | 10.5755 | 0.478462 | 13.33762 |
| North Africa and Middle East | 2006 | 0.560089 | 0.76708 | 17.7006 | 0.358933 | 10.5755 | 0.478462 | 13.33762 |
| North Africa and Middle East | 2007 | 0.566516 | 0.802857 | 18.49778 | 0.368796 | 10.8414 | 0.483621 | 13.42548 |
| North Africa and Middle East | 2007 | 0.566516 | 0.802857 | 18.49778 | 0.368796 | 10.8414 | 0.483621 | 13.42548 |
| North Africa and Middle East | 2007 | 0.566516 | 0.802857 | 18.49778 | 0.368796 | 10.8414 | 0.483621 | 13.42548 |
| North Africa and Middle East | 2007 | 0.566516 | 0.802857 | 18.49778 | 0.368796 | 10.8414 | 0.483621 | 13.42548 |
| North Africa and Middle East | 2008 | 0.572339 | 0.839447 | 19.28597 | 0.380852 | 11.15553 | 0.500383 | 13.81289 |
| North Africa and Middle East | 2008 | 0.572339 | 0.839447 | 19.28597 | 0.380852 | 11.15553 | 0.500383 | 13.81289 |
| North Africa and Middle East | 2008 | 0.572339 | 0.839447 | 19.28597 | 0.380852 | 11.15553 | 0.500383 | 13.81289 |
| North Africa and Middle East | 2008 | 0.572339 | 0.839447 | 19.28597 | 0.380852 | 11.15553 | 0.500383 | 13.81289 |
| North Africa and Middle East | 2009 | 0.577062 | 0.875464 | 20.06003 | 0.394409 | 11.49819 | 0.521614 | 14.34016 |
| North Africa and Middle East | 2009 | 0.577062 | 0.875464 | 20.06003 | 0.394409 | 11.49819 | 0.521614 | 14.34016 |
| North Africa and Middle East | 2009 | 0.577062 | 0.875464 | 20.06003 | 0.394409 | 11.49819 | 0.521614 | 14.34016 |
| North Africa and Middle East | 2009 | 0.577062 | 0.875464 | 20.06003 | 0.394409 | 11.49819 | 0.521614 | 14.34016 |
| North Africa and Middle East | 2010 | 0.581849 | 0.900863 | 20.67172 | 0.404993 | 11.80715 | 0.532684 | 14.67856 |
| North Africa and Middle East | 2010 | 0.581849 | 0.900863 | 20.67172 | 0.404993 | 11.80715 | 0.532684 | 14.67856 |
| North Africa and Middle East | 2010 | 0.581849 | 0.900863 | 20.67172 | 0.404993 | 11.80715 | 0.532684 | 14.67856 |
| North Africa and Middle East | 2010 | 0.581849 | 0.900863 | 20.67172 | 0.404993 | 11.80715 | 0.532684 | 14.67856 |
| North Africa and Middle East | 2011 | 0.587593 | 0.916452 | 21.02513 | 0.413316 | 12.06567 | 0.541293 | 14.93546 |
| North Africa and Middle East | 2011 | 0.587593 | 0.916452 | 21.02513 | 0.413316 | 12.06567 | 0.541293 | 14.93546 |
| North Africa and Middle East | 2011 | 0.587593 | 0.916452 | 21.02513 | 0.413316 | 12.06567 | 0.541293 | 14.93546 |
| North Africa and Middle East | 2011 | 0.587593 | 0.916452 | 21.02513 | 0.413316 | 12.06567 | 0.541293 | 14.93546 |
| North Africa and Middle East | 2012 | 0.594112 | 0.941734 | 21.60058 | 0.422934 | 12.34387 | 0.555516 | 15.35482 |
| North Africa and Middle East | 2012 | 0.594112 | 0.941734 | 21.60058 | 0.422934 | 12.34387 | 0.555516 | 15.35482 |
| North Africa and Middle East | 2012 | 0.594112 | 0.941734 | 21.60058 | 0.422934 | 12.34387 | 0.555516 | 15.35482 |
| North Africa and Middle East | 2012 | 0.594112 | 0.941734 | 21.60058 | 0.422934 | 12.34387 | 0.555516 | 15.35482 |
| North Africa and Middle East | 2013 | 0.601127 | 0.964966 | 22.03864 | 0.433365 | 12.60536 | 0.572221 | 15.77232 |
| North Africa and Middle East | 2013 | 0.601127 | 0.964966 | 22.03864 | 0.433365 | 12.60536 | 0.572221 | 15.77232 |
| North Africa and Middle East | 2013 | 0.601127 | 0.964966 | 22.03864 | 0.433365 | 12.60536 | 0.572221 | 15.77232 |
| North Africa and Middle East | 2013 | 0.601127 | 0.964966 | 22.03864 | 0.433365 | 12.60536 | 0.572221 | 15.77232 |
| North Africa and Middle East | 2014 | 0.608419 | 1.007245 | 22.93395 | 0.447579 | 12.95123 | 0.594809 | 16.30223 |
| North Africa and Middle East | 2014 | 0.608419 | 1.007245 | 22.93395 | 0.447579 | 12.95123 | 0.594809 | 16.30223 |
| North Africa and Middle East | 2014 | 0.608419 | 1.007245 | 22.93395 | 0.447579 | 12.95123 | 0.594809 | 16.30223 |
| North Africa and Middle East | 2014 | 0.608419 | 1.007245 | 22.93395 | 0.447579 | 12.95123 | 0.594809 | 16.30223 |
| North Africa and Middle East | 2015 | 0.615777 | 1.041361 | 23.67015 | 0.459125 | 13.22803 | 0.611339 | 16.65881 |
| North Africa and Middle East | 2015 | 0.615777 | 1.041361 | 23.67015 | 0.459125 | 13.22803 | 0.611339 | 16.65881 |
| North Africa and Middle East | 2015 | 0.615777 | 1.041361 | 23.67015 | 0.459125 | 13.22803 | 0.611339 | 16.65881 |
| North Africa and Middle East | 2015 | 0.615777 | 1.041361 | 23.67015 | 0.459125 | 13.22803 | 0.611339 | 16.65881 |
| North Africa and Middle East | 2016 | 0.623142 | 1.062522 | 24.13036 | 0.47049 | 13.51919 | 0.619849 | 16.85374 |
| North Africa and Middle East | 2016 | 0.623142 | 1.062522 | 24.13036 | 0.47049 | 13.51919 | 0.619849 | 16.85374 |
| North Africa and Middle East | 2016 | 0.623142 | 1.062522 | 24.13036 | 0.47049 | 13.51919 | 0.619849 | 16.85374 |
| North Africa and Middle East | 2016 | 0.623142 | 1.062522 | 24.13036 | 0.47049 | 13.51919 | 0.619849 | 16.85374 |
| North Africa and Middle East | 2017 | 0.630561 | 1.068608 | 24.24883 | 0.477584 | 13.71833 | 0.615415 | 16.74322 |
| North Africa and Middle East | 2017 | 0.630561 | 1.068608 | 24.24883 | 0.477584 | 13.71833 | 0.615415 | 16.74322 |
| North Africa and Middle East | 2017 | 0.630561 | 1.068608 | 24.24883 | 0.477584 | 13.71833 | 0.615415 | 16.74322 |
| North Africa and Middle East | 2017 | 0.630561 | 1.068608 | 24.24883 | 0.477584 | 13.71833 | 0.615415 | 16.74322 |
| North Africa and Middle East | 2018 | 0.637918 | 1.080941 | 24.60008 | 0.483719 | 13.92207 | 0.610895 | 16.70808 |
| North Africa and Middle East | 2018 | 0.637918 | 1.080941 | 24.60008 | 0.483719 | 13.92207 | 0.610895 | 16.70808 |
| North Africa and Middle East | 2018 | 0.637918 | 1.080941 | 24.60008 | 0.483719 | 13.92207 | 0.610895 | 16.70808 |
| North Africa and Middle East | 2018 | 0.637918 | 1.080941 | 24.60008 | 0.483719 | 13.92207 | 0.610895 | 16.70808 |
| North Africa and Middle East | 2019 | 0.645095 | 1.103224 | 25.07787 | 0.49351 | 14.17184 | 0.614283 | 16.76772 |
| North Africa and Middle East | 2019 | 0.645095 | 1.103224 | 25.07787 | 0.49351 | 14.17184 | 0.614283 | 16.76772 |
| North Africa and Middle East | 2019 | 0.645095 | 1.103224 | 25.07787 | 0.49351 | 14.17184 | 0.614283 | 16.76772 |
| North Africa and Middle East | 2019 | 0.645095 | 1.103224 | 25.07787 | 0.49351 | 14.17184 | 0.614283 | 16.76772 |
| North Africa and Middle East | 2020 | 0.651673 | 1.110607 | 25.18664 | 0.500373 | 14.31412 | 0.618558 | 16.82127 |
| North Africa and Middle East | 2020 | 0.651673 | 1.110607 | 25.18664 | 0.500373 | 14.31412 | 0.618558 | 16.82127 |
| North Africa and Middle East | 2020 | 0.651673 | 1.110607 | 25.18664 | 0.500373 | 14.31412 | 0.618558 | 16.82127 |
| North Africa and Middle East | 2020 | 0.651673 | 1.110607 | 25.18664 | 0.500373 | 14.31412 | 0.618558 | 16.82127 |
| North Africa and Middle East | 2021 | 0.658225 | 1.122011 | 25.48563 | 0.51097 | 14.62257 | 0.624428 | 17.03691 |
| North Africa and Middle East | 2021 | 0.658225 | 1.122011 | 25.48563 | 0.51097 | 14.62257 | 0.624428 | 17.03691 |
| North Africa and Middle East | 2021 | 0.658225 | 1.122011 | 25.48563 | 0.51097 | 14.62257 | 0.624428 | 17.03691 |
| North Africa and Middle East | 2021 | 0.658225 | 1.122011 | 25.48563 | 0.51097 | 14.62257 | 0.624428 | 17.03691 |
| Oceania | 1990 | 0.391195 | 1.205699 | 32.90791 | 0.10537 | 3.407392 | 0.846546 | 25.26054 |
| Oceania | 1991 | 0.394419 | 1.236526 | 33.77159 | 0.108525 | 3.508372 | 0.866428 | 25.8678 |
| Oceania | 1992 | 0.397572 | 1.251214 | 34.24342 | 0.110456 | 3.575387 | 0.876954 | 26.19966 |
| Oceania | 1993 | 0.401113 | 1.2648 | 34.69003 | 0.112693 | 3.655026 | 0.88696 | 26.52684 |
| Oceania | 1994 | 0.404892 | 1.276421 | 35.07408 | 0.114914 | 3.734091 | 0.894686 | 26.78792 |
| Oceania | 1995 | 0.408507 | 1.294861 | 35.56712 | 0.120607 | 3.919312 | 0.905616 | 27.12321 |
| Oceania | 1996 | 0.412437 | 1.323383 | 36.34142 | 0.124928 | 4.053233 | 0.924408 | 27.65247 |
| Oceania | 1997 | 0.415893 | 1.330252 | 36.50991 | 0.127997 | 4.149823 | 0.929889 | 27.80275 |
| Oceania | 1998 | 0.419225 | 1.337083 | 36.71074 | 0.130624 | 4.234519 | 0.930825 | 27.84324 |
| Oceania | 1999 | 0.422521 | 1.357917 | 37.3541 | 0.132359 | 4.295304 | 0.941098 | 28.179 |
| Oceania | 2000 | 0.42507 | 1.37631 | 37.96201 | 0.140935 | 4.569386 | 0.955275 | 28.61761 |
| Oceania | 2001 | 0.427004 | 1.370819 | 37.7832 | 0.144899 | 4.686457 | 0.958348 | 28.62835 |
| Oceania | 2002 | 0.428535 | 1.369073 | 37.8185 | 0.147766 | 4.785029 | 0.97027 | 29.02289 |
| Oceania | 2003 | 0.43006 | 1.381646 | 38.1588 | 0.149684 | 4.848479 | 0.980046 | 29.30214 |
| Oceania | 2004 | 0.431581 | 1.377023 | 38.02536 | 0.151094 | 4.892429 | 0.981331 | 29.34013 |
| Oceania | 2005 | 0.433152 | 1.395457 | 38.56618 | 0.154051 | 4.992941 | 0.997897 | 29.86727 |
| Oceania | 2006 | 0.434584 | 1.419953 | 39.12775 | 0.15709 | 5.081929 | 1.031543 | 30.7924 |
| Oceania | 2007 | 0.436102 | 1.441377 | 39.67752 | 0.159146 | 5.157202 | 1.049405 | 31.36059 |
| Oceania | 2008 | 0.437392 | 1.465606 | 40.20335 | 0.162287 | 5.223062 | 1.060096 | 31.61079 |
| Oceania | 2009 | 0.438808 | 1.48378 | 40.62045 | 0.163975 | 5.257302 | 1.073937 | 31.95085 |
| Oceania | 2010 | 0.440752 | 1.517703 | 41.52822 | 0.167477 | 5.370124 | 1.091255 | 32.48257 |
| Oceania | 2011 | 0.442618 | 1.553004 | 42.38357 | 0.170088 | 5.449014 | 1.112689 | 33.06352 |
| Oceania | 2012 | 0.444524 | 1.569228 | 42.84205 | 0.172305 | 5.517657 | 1.116731 | 33.21197 |
| Oceania | 2013 | 0.446594 | 1.579008 | 43.02617 | 0.173494 | 5.533164 | 1.113522 | 33.04251 |
| Oceania | 2014 | 0.449364 | 1.600438 | 43.54974 | 0.17604 | 5.605631 | 1.123559 | 33.28945 |
| Oceania | 2015 | 0.452397 | 1.625082 | 44.21823 | 0.180717 | 5.773028 | 1.138443 | 33.73866 |
| Oceania | 2016 | 0.455454 | 1.635151 | 44.31198 | 0.180536 | 5.747621 | 1.13935 | 33.66621 |
| Oceania | 2017 | 0.458378 | 1.655214 | 44.87793 | 0.182681 | 5.815294 | 1.148189 | 33.97395 |
| Oceania | 2018 | 0.460818 | 1.666035 | 45.12955 | 0.18427 | 5.862199 | 1.152602 | 34.12076 |
| Oceania | 2019 | 0.463334 | 1.684811 | 45.65081 | 0.186629 | 5.951602 | 1.166346 | 34.56564 |
| Oceania | 2020 | 0.465535 | 1.676083 | 45.49465 | 0.184239 | 5.920344 | 1.165391 | 34.57499 |
| Oceania | 2021 | 0.467445 | 1.674645 | 45.28549 | 0.185115 | 5.931305 | 1.164091 | 34.4755 |
| South Asia | 1990 | 0.319797 | 0.130445 | 2.32255 | 0.029056 | 0.977678 | 0.106708 | 2.957561 |
| South Asia | 1990 | 0.319797 | 0.130445 | 2.32255 | 0.029056 | 0.977678 | 0.106708 | 2.957561 |
| South Asia | 1990 | 0.319797 | 0.130445 | 2.32255 | 0.029056 | 0.977678 | 0.106708 | 2.957561 |
| South Asia | 1990 | 0.319797 | 0.130445 | 2.32255 | 0.029056 | 0.977678 | 0.106708 | 2.957561 |
| South Asia | 1991 | 0.325854 | 0.133527 | 2.386642 | 0.030673 | 1.034358 | 0.10888 | 3.02322 |
| South Asia | 1991 | 0.325854 | 0.133527 | 2.386642 | 0.030673 | 1.034358 | 0.10888 | 3.02322 |
| South Asia | 1991 | 0.325854 | 0.133527 | 2.386642 | 0.030673 | 1.034358 | 0.10888 | 3.02322 |
| South Asia | 1991 | 0.325854 | 0.133527 | 2.386642 | 0.030673 | 1.034358 | 0.10888 | 3.02322 |
| South Asia | 1992 | 0.331941 | 0.140222 | 2.518931 | 0.032621 | 1.09791 | 0.112775 | 3.124916 |
| South Asia | 1992 | 0.331941 | 0.140222 | 2.518931 | 0.032621 | 1.09791 | 0.112775 | 3.124916 |
| South Asia | 1992 | 0.331941 | 0.140222 | 2.518931 | 0.032621 | 1.09791 | 0.112775 | 3.124916 |
| South Asia | 1992 | 0.331941 | 0.140222 | 2.518931 | 0.032621 | 1.09791 | 0.112775 | 3.124916 |
| South Asia | 1993 | 0.338013 | 0.145718 | 2.638175 | 0.034578 | 1.160769 | 0.115646 | 3.20372 |
| South Asia | 1993 | 0.338013 | 0.145718 | 2.638175 | 0.034578 | 1.160769 | 0.115646 | 3.20372 |
| South Asia | 1993 | 0.338013 | 0.145718 | 2.638175 | 0.034578 | 1.160769 | 0.115646 | 3.20372 |
| South Asia | 1993 | 0.338013 | 0.145718 | 2.638175 | 0.034578 | 1.160769 | 0.115646 | 3.20372 |
| South Asia | 1994 | 0.344323 | 0.159595 | 2.942973 | 0.037448 | 1.234532 | 0.121198 | 3.329859 |
| South Asia | 1994 | 0.344323 | 0.159595 | 2.942973 | 0.037448 | 1.234532 | 0.121198 | 3.329859 |
| South Asia | 1994 | 0.344323 | 0.159595 | 2.942973 | 0.037448 | 1.234532 | 0.121198 | 3.329859 |
| South Asia | 1994 | 0.344323 | 0.159595 | 2.942973 | 0.037448 | 1.234532 | 0.121198 | 3.329859 |
| South Asia | 1995 | 0.350726 | 0.168619 | 3.090399 | 0.039786 | 1.303769 | 0.125803 | 3.432585 |
| South Asia | 1995 | 0.350726 | 0.168619 | 3.090399 | 0.039786 | 1.303769 | 0.125803 | 3.432585 |
| South Asia | 1995 | 0.350726 | 0.168619 | 3.090399 | 0.039786 | 1.303769 | 0.125803 | 3.432585 |
| South Asia | 1995 | 0.350726 | 0.168619 | 3.090399 | 0.039786 | 1.303769 | 0.125803 | 3.432585 |
| South Asia | 1996 | 0.357287 | 0.171948 | 3.140864 | 0.042032 | 1.378627 | 0.127804 | 3.496195 |
| South Asia | 1996 | 0.357287 | 0.171948 | 3.140864 | 0.042032 | 1.378627 | 0.127804 | 3.496195 |
| South Asia | 1996 | 0.357287 | 0.171948 | 3.140864 | 0.042032 | 1.378627 | 0.127804 | 3.496195 |
| South Asia | 1996 | 0.357287 | 0.171948 | 3.140864 | 0.042032 | 1.378627 | 0.127804 | 3.496195 |
| South Asia | 1997 | 0.363719 | 0.175466 | 3.230181 | 0.044757 | 1.468773 | 0.130175 | 3.583996 |
| South Asia | 1997 | 0.363719 | 0.175466 | 3.230181 | 0.044757 | 1.468773 | 0.130175 | 3.583996 |
| South Asia | 1997 | 0.363719 | 0.175466 | 3.230181 | 0.044757 | 1.468773 | 0.130175 | 3.583996 |
| South Asia | 1997 | 0.363719 | 0.175466 | 3.230181 | 0.044757 | 1.468773 | 0.130175 | 3.583996 |
| South Asia | 1998 | 0.370288 | 0.181853 | 3.381021 | 0.048065 | 1.573006 | 0.133435 | 3.680311 |
| South Asia | 1998 | 0.370288 | 0.181853 | 3.381021 | 0.048065 | 1.573006 | 0.133435 | 3.680311 |
| South Asia | 1998 | 0.370288 | 0.181853 | 3.381021 | 0.048065 | 1.573006 | 0.133435 | 3.680311 |
| South Asia | 1998 | 0.370288 | 0.181853 | 3.381021 | 0.048065 | 1.573006 | 0.133435 | 3.680311 |
| South Asia | 1999 | 0.376979 | 0.185893 | 3.473467 | 0.051355 | 1.677008 | 0.134895 | 3.733227 |
| South Asia | 1999 | 0.376979 | 0.185893 | 3.473467 | 0.051355 | 1.677008 | 0.134895 | 3.733227 |
| South Asia | 1999 | 0.376979 | 0.185893 | 3.473467 | 0.051355 | 1.677008 | 0.134895 | 3.733227 |
| South Asia | 1999 | 0.376979 | 0.185893 | 3.473467 | 0.051355 | 1.677008 | 0.134895 | 3.733227 |
| South Asia | 2000 | 0.383388 | 0.192115 | 3.640235 | 0.055002 | 1.791076 | 0.137278 | 3.81007 |
| South Asia | 2000 | 0.383388 | 0.192115 | 3.640235 | 0.055002 | 1.791076 | 0.137278 | 3.81007 |
| South Asia | 2000 | 0.383388 | 0.192115 | 3.640235 | 0.055002 | 1.791076 | 0.137278 | 3.81007 |
| South Asia | 2000 | 0.383388 | 0.192115 | 3.640235 | 0.055002 | 1.791076 | 0.137278 | 3.81007 |
| South Asia | 2001 | 0.389533 | 0.198271 | 3.781537 | 0.058431 | 1.895728 | 0.140009 | 3.886811 |
| South Asia | 2001 | 0.389533 | 0.198271 | 3.781537 | 0.058431 | 1.895728 | 0.140009 | 3.886811 |
| South Asia | 2001 | 0.389533 | 0.198271 | 3.781537 | 0.058431 | 1.895728 | 0.140009 | 3.886811 |
| South Asia | 2001 | 0.389533 | 0.198271 | 3.781537 | 0.058431 | 1.895728 | 0.140009 | 3.886811 |
| South Asia | 2002 | 0.395194 | 0.203284 | 3.868087 | 0.062289 | 2.01182 | 0.143876 | 3.990678 |
| South Asia | 2002 | 0.395194 | 0.203284 | 3.868087 | 0.062289 | 2.01182 | 0.143876 | 3.990678 |
| South Asia | 2002 | 0.395194 | 0.203284 | 3.868087 | 0.062289 | 2.01182 | 0.143876 | 3.990678 |
| South Asia | 2002 | 0.395194 | 0.203284 | 3.868087 | 0.062289 | 2.01182 | 0.143876 | 3.990678 |
| South Asia | 2003 | 0.400983 | 0.209838 | 4.013782 | 0.06644 | 2.136282 | 0.147559 | 4.086159 |
| South Asia | 2003 | 0.400983 | 0.209838 | 4.013782 | 0.06644 | 2.136282 | 0.147559 | 4.086159 |
| South Asia | 2003 | 0.400983 | 0.209838 | 4.013782 | 0.06644 | 2.136282 | 0.147559 | 4.086159 |
| South Asia | 2003 | 0.400983 | 0.209838 | 4.013782 | 0.06644 | 2.136282 | 0.147559 | 4.086159 |
| South Asia | 2004 | 0.407181 | 0.214342 | 4.114894 | 0.070376 | 2.25507 | 0.14956 | 4.139562 |
| South Asia | 2004 | 0.407181 | 0.214342 | 4.114894 | 0.070376 | 2.25507 | 0.14956 | 4.139562 |
| South Asia | 2004 | 0.407181 | 0.214342 | 4.114894 | 0.070376 | 2.25507 | 0.14956 | 4.139562 |
| South Asia | 2004 | 0.407181 | 0.214342 | 4.114894 | 0.070376 | 2.25507 | 0.14956 | 4.139562 |
| South Asia | 2005 | 0.413987 | 0.222091 | 4.28543 | 0.075101 | 2.395852 | 0.153553 | 4.243304 |
| South Asia | 2005 | 0.413987 | 0.222091 | 4.28543 | 0.075101 | 2.395852 | 0.153553 | 4.243304 |
| South Asia | 2005 | 0.413987 | 0.222091 | 4.28543 | 0.075101 | 2.395852 | 0.153553 | 4.243304 |
| South Asia | 2005 | 0.413987 | 0.222091 | 4.28543 | 0.075101 | 2.395852 | 0.153553 | 4.243304 |
| South Asia | 2006 | 0.421367 | 0.232805 | 4.533713 | 0.079555 | 2.526704 | 0.159504 | 4.393376 |
| South Asia | 2006 | 0.421367 | 0.232805 | 4.533713 | 0.079555 | 2.526704 | 0.159504 | 4.393376 |
| South Asia | 2006 | 0.421367 | 0.232805 | 4.533713 | 0.079555 | 2.526704 | 0.159504 | 4.393376 |
| South Asia | 2006 | 0.421367 | 0.232805 | 4.533713 | 0.079555 | 2.526704 | 0.159504 | 4.393376 |
| South Asia | 2007 | 0.429262 | 0.244135 | 4.794629 | 0.08447 | 2.674355 | 0.164606 | 4.526095 |
| South Asia | 2007 | 0.429262 | 0.244135 | 4.794629 | 0.08447 | 2.674355 | 0.164606 | 4.526095 |
| South Asia | 2007 | 0.429262 | 0.244135 | 4.794629 | 0.08447 | 2.674355 | 0.164606 | 4.526095 |
| South Asia | 2007 | 0.429262 | 0.244135 | 4.794629 | 0.08447 | 2.674355 | 0.164606 | 4.526095 |
| South Asia | 2008 | 0.437012 | 0.251705 | 4.944436 | 0.089622 | 2.833899 | 0.170175 | 4.681523 |
| South Asia | 2008 | 0.437012 | 0.251705 | 4.944436 | 0.089622 | 2.833899 | 0.170175 | 4.681523 |
| South Asia | 2008 | 0.437012 | 0.251705 | 4.944436 | 0.089622 | 2.833899 | 0.170175 | 4.681523 |
| South Asia | 2008 | 0.437012 | 0.251705 | 4.944436 | 0.089622 | 2.833899 | 0.170175 | 4.681523 |
| South Asia | 2009 | 0.445197 | 0.260158 | 5.17182 | 0.094134 | 2.975716 | 0.172875 | 4.772663 |
| South Asia | 2009 | 0.445197 | 0.260158 | 5.17182 | 0.094134 | 2.975716 | 0.172875 | 4.772663 |
| South Asia | 2009 | 0.445197 | 0.260158 | 5.17182 | 0.094134 | 2.975716 | 0.172875 | 4.772663 |
| South Asia | 2009 | 0.445197 | 0.260158 | 5.17182 | 0.094134 | 2.975716 | 0.172875 | 4.772663 |
| South Asia | 2010 | 0.454076 | 0.270526 | 5.45965 | 0.099015 | 3.127213 | 0.177018 | 4.900974 |
| South Asia | 2010 | 0.454076 | 0.270526 | 5.45965 | 0.099015 | 3.127213 | 0.177018 | 4.900974 |
| South Asia | 2010 | 0.454076 | 0.270526 | 5.45965 | 0.099015 | 3.127213 | 0.177018 | 4.900974 |
| South Asia | 2010 | 0.454076 | 0.270526 | 5.45965 | 0.099015 | 3.127213 | 0.177018 | 4.900974 |
| South Asia | 2011 | 0.463339 | 0.282761 | 5.793193 | 0.103936 | 3.278585 | 0.181717 | 5.034569 |
| South Asia | 2011 | 0.463339 | 0.282761 | 5.793193 | 0.103936 | 3.278585 | 0.181717 | 5.034569 |
| South Asia | 2011 | 0.463339 | 0.282761 | 5.793193 | 0.103936 | 3.278585 | 0.181717 | 5.034569 |
| South Asia | 2011 | 0.463339 | 0.282761 | 5.793193 | 0.103936 | 3.278585 | 0.181717 | 5.034569 |
| South Asia | 2012 | 0.473081 | 0.290923 | 5.946672 | 0.108468 | 3.42236 | 0.187153 | 5.193255 |
| South Asia | 2012 | 0.473081 | 0.290923 | 5.946672 | 0.108468 | 3.42236 | 0.187153 | 5.193255 |
| South Asia | 2012 | 0.473081 | 0.290923 | 5.946672 | 0.108468 | 3.42236 | 0.187153 | 5.193255 |
| South Asia | 2012 | 0.473081 | 0.290923 | 5.946672 | 0.108468 | 3.42236 | 0.187153 | 5.193255 |
| South Asia | 2013 | 0.483434 | 0.301034 | 6.11948 | 0.113519 | 3.588461 | 0.191189 | 5.314484 |
| South Asia | 2013 | 0.483434 | 0.301034 | 6.11948 | 0.113519 | 3.588461 | 0.191189 | 5.314484 |
| South Asia | 2013 | 0.483434 | 0.301034 | 6.11948 | 0.113519 | 3.588461 | 0.191189 | 5.314484 |
| South Asia | 2013 | 0.483434 | 0.301034 | 6.11948 | 0.113519 | 3.588461 | 0.191189 | 5.314484 |
| South Asia | 2014 | 0.494084 | 0.313788 | 6.46442 | 0.119057 | 3.764068 | 0.195494 | 5.441891 |
| South Asia | 2014 | 0.494084 | 0.313788 | 6.46442 | 0.119057 | 3.764068 | 0.195494 | 5.441891 |
| South Asia | 2014 | 0.494084 | 0.313788 | 6.46442 | 0.119057 | 3.764068 | 0.195494 | 5.441891 |
| South Asia | 2014 | 0.494084 | 0.313788 | 6.46442 | 0.119057 | 3.764068 | 0.195494 | 5.441891 |
| South Asia | 2015 | 0.504891 | 0.332943 | 6.96994 | 0.126238 | 3.985427 | 0.203461 | 5.678611 |
| South Asia | 2015 | 0.504891 | 0.332943 | 6.96994 | 0.126238 | 3.985427 | 0.203461 | 5.678611 |
| South Asia | 2015 | 0.504891 | 0.332943 | 6.96994 | 0.126238 | 3.985427 | 0.203461 | 5.678611 |
| South Asia | 2015 | 0.504891 | 0.332943 | 6.96994 | 0.126238 | 3.985427 | 0.203461 | 5.678611 |
| South Asia | 2016 | 0.515311 | 0.34231 | 7.131252 | 0.132095 | 4.165772 | 0.209062 | 5.838587 |
| South Asia | 2016 | 0.515311 | 0.34231 | 7.131252 | 0.132095 | 4.165772 | 0.209062 | 5.838587 |
| South Asia | 2016 | 0.515311 | 0.34231 | 7.131252 | 0.132095 | 4.165772 | 0.209062 | 5.838587 |
| South Asia | 2016 | 0.515311 | 0.34231 | 7.131252 | 0.132095 | 4.165772 | 0.209062 | 5.838587 |
| South Asia | 2017 | 0.525128 | 0.355562 | 7.356627 | 0.139561 | 4.39467 | 0.217034 | 6.054675 |
| South Asia | 2017 | 0.525128 | 0.355562 | 7.356627 | 0.139561 | 4.39467 | 0.217034 | 6.054675 |
| South Asia | 2017 | 0.525128 | 0.355562 | 7.356627 | 0.139561 | 4.39467 | 0.217034 | 6.054675 |
| South Asia | 2017 | 0.525128 | 0.355562 | 7.356627 | 0.139561 | 4.39467 | 0.217034 | 6.054675 |
| South Asia | 2018 | 0.534477 | 0.370503 | 7.755233 | 0.146305 | 4.599839 | 0.224039 | 6.250827 |
| South Asia | 2018 | 0.534477 | 0.370503 | 7.755233 | 0.146305 | 4.599839 | 0.224039 | 6.250827 |
| South Asia | 2018 | 0.534477 | 0.370503 | 7.755233 | 0.146305 | 4.599839 | 0.224039 | 6.250827 |
| South Asia | 2018 | 0.534477 | 0.370503 | 7.755233 | 0.146305 | 4.599839 | 0.224039 | 6.250827 |
| South Asia | 2019 | 0.543246 | 0.379205 | 7.944122 | 0.152027 | 4.773729 | 0.229509 | 6.397601 |
| South Asia | 2019 | 0.543246 | 0.379205 | 7.944122 | 0.152027 | 4.773729 | 0.229509 | 6.397601 |
| South Asia | 2019 | 0.543246 | 0.379205 | 7.944122 | 0.152027 | 4.773729 | 0.229509 | 6.397601 |
| South Asia | 2019 | 0.543246 | 0.379205 | 7.944122 | 0.152027 | 4.773729 | 0.229509 | 6.397601 |
| South Asia | 2020 | 0.550586 | 0.378292 | 7.822401 | 0.157048 | 4.942695 | 0.232042 | 6.480293 |
| South Asia | 2020 | 0.550586 | 0.378292 | 7.822401 | 0.157048 | 4.942695 | 0.232042 | 6.480293 |
| South Asia | 2020 | 0.550586 | 0.378292 | 7.822401 | 0.157048 | 4.942695 | 0.232042 | 6.480293 |
| South Asia | 2020 | 0.550586 | 0.378292 | 7.822401 | 0.157048 | 4.942695 | 0.232042 | 6.480293 |
| South Asia | 2021 | 0.557865 | 0.382787 | 7.92589 | 0.162751 | 5.114796 | 0.234534 | 6.558374 |
| South Asia | 2021 | 0.557865 | 0.382787 | 7.92589 | 0.162751 | 5.114796 | 0.234534 | 6.558374 |
| South Asia | 2021 | 0.557865 | 0.382787 | 7.92589 | 0.162751 | 5.114796 | 0.234534 | 6.558374 |
| South Asia | 2021 | 0.557865 | 0.382787 | 7.92589 | 0.162751 | 5.114796 | 0.234534 | 6.558374 |
| Southeast Asia | 1990 | 0.464104 | 0.325754 | 9.389985 | 0.045996 | 1.665069 | 0.216802 | 6.839926 |
| Southeast Asia | 1991 | 0.47175 | 0.336016 | 9.683056 | 0.048691 | 1.761279 | 0.221729 | 6.99663 |
| Southeast Asia | 1992 | 0.47949 | 0.349066 | 10.06498 | 0.052106 | 1.883316 | 0.228774 | 7.224368 |
| Southeast Asia | 1993 | 0.487349 | 0.362069 | 10.45072 | 0.055437 | 2.002932 | 0.236193 | 7.465704 |
| Southeast Asia | 1994 | 0.495348 | 0.375277 | 10.82597 | 0.059133 | 2.133904 | 0.243568 | 7.69846 |
| Southeast Asia | 1995 | 0.503351 | 0.388816 | 11.21199 | 0.063075 | 2.273432 | 0.250739 | 7.927975 |
| Southeast Asia | 1996 | 0.511233 | 0.403736 | 11.64442 | 0.067215 | 2.419926 | 0.259163 | 8.201769 |
| Southeast Asia | 1997 | 0.518664 | 0.416538 | 12.01775 | 0.071332 | 2.565925 | 0.265567 | 8.414519 |
| Southeast Asia | 1998 | 0.524439 | 0.430864 | 12.4368 | 0.075735 | 2.723308 | 0.273165 | 8.669874 |
| Southeast Asia | 1999 | 0.529668 | 0.451665 | 13.05619 | 0.081155 | 2.917168 | 0.284947 | 9.059928 |
| Southeast Asia | 2000 | 0.534567 | 0.468716 | 13.57563 | 0.086359 | 3.103234 | 0.294238 | 9.374496 |
| Southeast Asia | 2001 | 0.53901 | 0.481602 | 13.95875 | 0.091065 | 3.2702 | 0.300466 | 9.584104 |
| Southeast Asia | 2002 | 0.543479 | 0.502221 | 14.57247 | 0.09689 | 3.473741 | 0.309506 | 9.875539 |
| Southeast Asia | 2003 | 0.548051 | 0.522346 | 15.17247 | 0.103006 | 3.683311 | 0.317487 | 10.12462 |
| Southeast Asia | 2004 | 0.552707 | 0.54146 | 15.74183 | 0.109076 | 3.887219 | 0.325548 | 10.37189 |
| Southeast Asia | 2005 | 0.557391 | 0.55786 | 16.21788 | 0.115141 | 4.086022 | 0.33316 | 10.58968 |
| Southeast Asia | 2006 | 0.562302 | 0.575795 | 16.7332 | 0.120984 | 4.278063 | 0.340066 | 10.79585 |
| Southeast Asia | 2007 | 0.567654 | 0.59207 | 17.19246 | 0.127012 | 4.478227 | 0.34593 | 10.97152 |
| Southeast Asia | 2008 | 0.573208 | 0.609084 | 17.65827 | 0.132888 | 4.670139 | 0.35199 | 11.15225 |
| Southeast Asia | 2009 | 0.578497 | 0.626865 | 18.15325 | 0.138659 | 4.858622 | 0.358141 | 11.33265 |
| Southeast Asia | 2010 | 0.584268 | 0.642587 | 18.58305 | 0.144708 | 5.055254 | 0.363535 | 11.49326 |
| Southeast Asia | 2011 | 0.590415 | 0.658375 | 19.02481 | 0.150409 | 5.242494 | 0.36825 | 11.63587 |
| Southeast Asia | 2012 | 0.59678 | 0.669228 | 19.319 | 0.154873 | 5.384689 | 0.372003 | 11.73939 |
| Southeast Asia | 2013 | 0.603145 | 0.682296 | 19.6821 | 0.159667 | 5.541135 | 0.377784 | 11.90791 |
| Southeast Asia | 2014 | 0.609439 | 0.696736 | 20.08062 | 0.164535 | 5.698009 | 0.385135 | 12.11614 |
| Southeast Asia | 2015 | 0.615689 | 0.71549 | 20.60041 | 0.170239 | 5.882408 | 0.394105 | 12.37956 |
| Southeast Asia | 2016 | 0.62184 | 0.732844 | 21.08531 | 0.175229 | 6.041415 | 0.401182 | 12.58905 |
| Southeast Asia | 2017 | 0.627978 | 0.749284 | 21.52058 | 0.180462 | 6.200945 | 0.408479 | 12.78978 |
| Southeast Asia | 2018 | 0.634096 | 0.766772 | 21.97396 | 0.185927 | 6.364155 | 0.415317 | 12.97901 |
| Southeast Asia | 2019 | 0.640107 | 0.787191 | 22.53066 | 0.191735 | 6.547743 | 0.424691 | 13.2527 |
| Southeast Asia | 2020 | 0.64507 | 0.787081 | 22.52894 | 0.194394 | 6.638044 | 0.42523 | 13.27735 |
| Southeast Asia | 2021 | 0.649777 | 0.802983 | 22.90952 | 0.200537 | 6.815117 | 0.434707 | 13.5208 |
| Southern Latin America | 1990 | 0.587308 | 2.027398 | 43.8336 | 0.59202 | 16.81486 | 1.030748 | 26.84779 |
| Southern Latin America | 1991 | 0.592119 | 2.046266 | 44.14437 | 0.593563 | 16.8689 | 1.020232 | 26.44346 |
| Southern Latin America | 1992 | 0.59808 | 2.059586 | 44.22859 | 0.60339 | 17.15653 | 1.050738 | 27.15961 |
| Southern Latin America | 1993 | 0.603589 | 2.095261 | 45.13616 | 0.602049 | 17.13057 | 1.050934 | 27.11938 |
| Southern Latin America | 1994 | 0.609392 | 2.09567 | 45.0468 | 0.608977 | 17.39454 | 1.048562 | 27.20541 |
| Southern Latin America | 1995 | 0.614373 | 2.12593 | 45.85629 | 0.614556 | 17.50912 | 1.032927 | 26.74535 |
| Southern Latin America | 1996 | 0.61926 | 2.201425 | 47.75942 | 0.624447 | 17.74961 | 1.053505 | 27.01544 |
| Southern Latin America | 1997 | 0.624756 | 2.237998 | 48.90506 | 0.647883 | 18.51419 | 1.087687 | 27.29501 |
| Southern Latin America | 1998 | 0.630349 | 2.273958 | 49.63642 | 0.65904 | 18.73145 | 1.075231 | 26.69186 |
| Southern Latin America | 1999 | 0.635748 | 2.257354 | 49.3968 | 0.666665 | 19.04796 | 1.038506 | 25.56285 |
| Southern Latin America | 2000 | 0.641237 | 2.25774 | 49.54197 | 0.688491 | 19.71223 | 1.01578 | 25.10626 |
| Southern Latin America | 2001 | 0.64604 | 2.265541 | 49.69468 | 0.695657 | 19.85132 | 0.994127 | 24.69169 |
| Southern Latin America | 2002 | 0.649826 | 2.295024 | 50.5989 | 0.705981 | 20.07824 | 0.977126 | 24.11231 |
| Southern Latin America | 2003 | 0.652552 | 2.363105 | 52.33728 | 0.712383 | 20.25744 | 0.951809 | 23.3934 |
| Southern Latin America | 2004 | 0.655719 | 2.338367 | 51.72563 | 0.713003 | 20.25782 | 0.956155 | 23.64755 |
| Southern Latin America | 2005 | 0.660519 | 2.312422 | 50.78232 | 0.727817 | 20.65271 | 0.93034 | 23.03232 |
| Southern Latin America | 2006 | 0.664386 | 2.307152 | 50.80976 | 0.743027 | 21.05341 | 0.921508 | 22.82284 |
| Southern Latin America | 2007 | 0.667393 | 2.299786 | 50.75743 | 0.73503 | 20.87695 | 0.912979 | 22.7082 |
| Southern Latin America | 2008 | 0.670689 | 2.248987 | 49.68253 | 0.719808 | 20.41278 | 0.916814 | 22.82841 |
| Southern Latin America | 2009 | 0.674152 | 2.275394 | 50.07769 | 0.736177 | 20.97188 | 0.936792 | 23.61018 |
| Southern Latin America | 2010 | 0.678761 | 2.258607 | 49.71475 | 0.738286 | 21.08196 | 0.94967 | 24.02131 |
| Southern Latin America | 2011 | 0.684134 | 2.24552 | 49.32232 | 0.731342 | 20.93691 | 0.952614 | 24.04021 |
| Southern Latin America | 2012 | 0.688428 | 2.219924 | 48.6311 | 0.721096 | 20.57822 | 0.959008 | 24.19211 |
| Southern Latin America | 2013 | 0.691716 | 2.204619 | 47.93609 | 0.734875 | 20.92024 | 0.9466 | 23.83209 |
| Southern Latin America | 2014 | 0.69604 | 2.18624 | 47.5135 | 0.709117 | 20.15482 | 0.929865 | 23.42183 |
| Southern Latin America | 2015 | 0.703287 | 2.193891 | 47.51903 | 0.710297 | 20.28451 | 0.940114 | 23.64302 |
| Southern Latin America | 2016 | 0.710788 | 2.186195 | 47.35999 | 0.736135 | 20.95898 | 0.952305 | 23.98888 |
| Southern Latin America | 2017 | 0.717548 | 2.154639 | 46.35237 | 0.737205 | 21.03157 | 0.931244 | 23.52547 |
| Southern Latin America | 2018 | 0.724952 | 2.085995 | 44.45674 | 0.725536 | 20.75273 | 0.930548 | 23.58102 |
| Southern Latin America | 2019 | 0.731134 | 2.090098 | 44.51884 | 0.729477 | 20.87005 | 0.929665 | 23.58445 |
| Southern Latin America | 2020 | 0.733963 | 2.024638 | 43.56322 | 0.704276 | 20.1334 | 0.897485 | 22.83101 |
| Southern Latin America | 2021 | 0.735985 | 1.946166 | 41.98777 | 0.680521 | 19.48075 | 0.86369 | 21.98947 |
| Southern Sub-Saharan Africa | 1990 | 0.506947 | 1.01963 | 19.50659 | 0.356276 | 10.57688 | 0.630293 | 16.44705 |
| Southern Sub-Saharan Africa | 1991 | 0.512378 | 1.029617 | 19.75437 | 0.363694 | 10.78952 | 0.628184 | 16.354 |
| Southern Sub-Saharan Africa | 1992 | 0.517569 | 1.079435 | 20.58075 | 0.380333 | 11.28197 | 0.66177 | 17.21194 |
| Southern Sub-Saharan Africa | 1993 | 0.522773 | 1.085848 | 21.11674 | 0.382989 | 11.28875 | 0.645314 | 16.57965 |
| Southern Sub-Saharan Africa | 1994 | 0.528063 | 1.147272 | 22.5462 | 0.398484 | 11.71543 | 0.678545 | 17.35496 |
| Southern Sub-Saharan Africa | 1995 | 0.53338 | 1.152783 | 23.0592 | 0.398842 | 11.64016 | 0.663987 | 16.7634 |
| Southern Sub-Saharan Africa | 1996 | 0.53878 | 1.249439 | 25.92339 | 0.411209 | 11.87652 | 0.706849 | 17.60975 |
| Southern Sub-Saharan Africa | 1997 | 0.544134 | 1.373932 | 28.7554 | 0.433665 | 12.49928 | 0.788745 | 19.66545 |
| Southern Sub-Saharan Africa | 1998 | 0.549142 | 1.406019 | 29.44598 | 0.439123 | 12.60692 | 0.816834 | 20.25422 |
| Southern Sub-Saharan Africa | 1999 | 0.553885 | 1.428955 | 29.72128 | 0.448865 | 12.86124 | 0.838206 | 20.71796 |
| Southern Sub-Saharan Africa | 2000 | 0.558491 | 1.470982 | 30.15524 | 0.463303 | 13.33269 | 0.892451 | 22.18104 |
| Southern Sub-Saharan Africa | 2001 | 0.562511 | 1.481255 | 29.84854 | 0.472687 | 13.59778 | 0.917331 | 22.73792 |
| Southern Sub-Saharan Africa | 2002 | 0.56605 | 1.510312 | 29.76284 | 0.492568 | 14.18888 | 0.964589 | 23.97549 |
| Southern Sub-Saharan Africa | 2003 | 0.569215 | 1.554215 | 30.08946 | 0.513758 | 14.83486 | 1.014845 | 25.348 |
| Southern Sub-Saharan Africa | 2004 | 0.57239 | 1.594344 | 30.69311 | 0.537353 | 15.5328 | 1.054906 | 26.53514 |
| Southern Sub-Saharan Africa | 2005 | 0.576116 | 1.6621 | 32.66409 | 0.556571 | 16.0456 | 1.086009 | 27.39894 |
| Southern Sub-Saharan Africa | 2006 | 0.580685 | 1.735651 | 34.79529 | 0.578294 | 16.64688 | 1.128568 | 28.48163 |
| Southern Sub-Saharan Africa | 2007 | 0.585673 | 1.803154 | 36.8554 | 0.596625 | 17.10528 | 1.149741 | 28.91478 |
| Southern Sub-Saharan Africa | 2008 | 0.590405 | 1.871649 | 38.8836 | 0.61867 | 17.74486 | 1.187523 | 29.95317 |
| Southern Sub-Saharan Africa | 2009 | 0.594585 | 1.938832 | 40.80638 | 0.640081 | 18.34005 | 1.223842 | 30.8893 |
| Southern Sub-Saharan Africa | 2010 | 0.598836 | 1.99529 | 42.43795 | 0.655818 | 18.77004 | 1.244326 | 31.42044 |
| Southern Sub-Saharan Africa | 2011 | 0.603347 | 2.036046 | 43.60851 | 0.671946 | 19.23312 | 1.25272 | 31.61014 |
| Southern Sub-Saharan Africa | 2012 | 0.608038 | 2.056346 | 44.10191 | 0.686384 | 19.65756 | 1.252686 | 31.58131 |
| Southern Sub-Saharan Africa | 2013 | 0.612844 | 2.081817 | 44.62921 | 0.702912 | 20.14602 | 1.265605 | 31.9026 |
| Southern Sub-Saharan Africa | 2014 | 0.617453 | 2.136822 | 45.85584 | 0.722202 | 20.7039 | 1.299477 | 32.76307 |
| Southern Sub-Saharan Africa | 2015 | 0.621838 | 2.187211 | 47.04383 | 0.740045 | 21.17526 | 1.325681 | 33.36096 |
| Southern Sub-Saharan Africa | 2016 | 0.625919 | 2.198657 | 47.43238 | 0.753047 | 21.54639 | 1.324671 | 33.3505 |
| Southern Sub-Saharan Africa | 2017 | 0.629762 | 2.175997 | 46.87182 | 0.760133 | 21.7356 | 1.301043 | 32.71811 |
| Southern Sub-Saharan Africa | 2018 | 0.633411 | 2.156141 | 46.18144 | 0.765185 | 21.89439 | 1.284131 | 32.28498 |
| Southern Sub-Saharan Africa | 2019 | 0.63694 | 2.098121 | 44.16435 | 0.765351 | 21.91605 | 1.251851 | 31.44244 |
| Southern Sub-Saharan Africa | 2020 | 0.639882 | 2.203249 | 46.80798 | 0.802812 | 22.90246 | 1.296151 | 32.52122 |
| Southern Sub-Saharan Africa | 2021 | 0.6422 | 2.192555 | 46.54314 | 0.810954 | 23.20125 | 1.288007 | 32.41318 |
| Tropical Latin America | 1990 | 0.499588 | 0.924455 | 19.89796 | 0.284257 | 8.523368 | 0.839224 | 21.40354 |
| Tropical Latin America | 1991 | 0.50429 | 0.93019 | 20.11815 | 0.2923 | 8.726743 | 0.853422 | 21.79875 |
| Tropical Latin America | 1992 | 0.508057 | 0.957293 | 20.7239 | 0.305958 | 9.1583 | 0.870893 | 22.12236 |
| Tropical Latin America | 1993 | 0.511622 | 0.994595 | 21.52213 | 0.3171 | 9.502333 | 0.875013 | 22.04199 |
| Tropical Latin America | 1994 | 0.51533 | 1.013982 | 21.98607 | 0.321778 | 9.579289 | 0.896529 | 22.68266 |
| Tropical Latin America | 1995 | 0.519291 | 1.015446 | 22.01974 | 0.332084 | 9.778644 | 0.874066 | 22.26136 |
| Tropical Latin America | 1996 | 0.523499 | 1.017935 | 22.07704 | 0.335318 | 9.925833 | 0.898701 | 22.9587 |
| Tropical Latin America | 1997 | 0.527841 | 1.018008 | 22.06934 | 0.346408 | 10.23292 | 0.883031 | 22.45781 |
| Tropical Latin America | 1998 | 0.532165 | 1.039959 | 22.48737 | 0.35554 | 10.49328 | 0.867936 | 22.05333 |
| Tropical Latin America | 1999 | 0.536659 | 1.046349 | 22.55459 | 0.36225 | 10.61731 | 0.870568 | 21.95463 |
| Tropical Latin America | 2000 | 0.541716 | 1.050028 | 22.71916 | 0.366768 | 10.72497 | 0.863693 | 21.75337 |
| Tropical Latin America | 2001 | 0.546932 | 1.059161 | 22.99674 | 0.368978 | 10.78189 | 0.851315 | 21.39114 |
| Tropical Latin America | 2002 | 0.552365 | 1.06799 | 23.18867 | 0.376011 | 10.95754 | 0.841888 | 21.30612 |
| Tropical Latin America | 2003 | 0.557691 | 1.075645 | 23.28299 | 0.380604 | 11.02817 | 0.860593 | 21.66778 |
| Tropical Latin America | 2004 | 0.563269 | 1.081399 | 23.46927 | 0.386484 | 11.14622 | 0.835624 | 21.0089 |
| Tropical Latin America | 2005 | 0.568897 | 1.071474 | 23.23203 | 0.383258 | 11.09029 | 0.828292 | 20.78304 |
| Tropical Latin America | 2006 | 0.57472 | 1.082983 | 23.47096 | 0.384912 | 11.15256 | 0.830942 | 20.77047 |
| Tropical Latin America | 2007 | 0.580915 | 1.070913 | 23.10286 | 0.383991 | 11.10079 | 0.839163 | 20.99831 |
| Tropical Latin America | 2008 | 0.587354 | 1.077527 | 23.30692 | 0.395007 | 11.4398 | 0.833625 | 20.88171 |
| Tropical Latin America | 2009 | 0.593303 | 1.076171 | 23.24442 | 0.398927 | 11.60024 | 0.821836 | 20.64719 |
| Tropical Latin America | 2010 | 0.599646 | 1.090757 | 23.6358 | 0.397762 | 11.56454 | 0.817822 | 20.54237 |
| Tropical Latin America | 2011 | 0.605957 | 1.09967 | 23.75038 | 0.396311 | 11.48434 | 0.836781 | 21.11395 |
| Tropical Latin America | 2012 | 0.611787 | 1.096157 | 23.67334 | 0.397015 | 11.54821 | 0.843857 | 21.23685 |
| Tropical Latin America | 2013 | 0.617402 | 1.092922 | 23.56376 | 0.400107 | 11.6134 | 0.843236 | 21.40681 |
| Tropical Latin America | 2014 | 0.622646 | 1.093552 | 23.47111 | 0.400238 | 11.69946 | 0.848424 | 21.56047 |
| Tropical Latin America | 2015 | 0.6274 | 1.118161 | 23.97254 | 0.412723 | 12.09836 | 0.863574 | 21.94414 |
| Tropical Latin America | 2016 | 0.631533 | 1.135968 | 24.2366 | 0.426038 | 12.46809 | 0.889673 | 22.75498 |
| Tropical Latin America | 2017 | 0.635765 | 1.136741 | 24.243 | 0.424465 | 12.44036 | 0.901203 | 23.00057 |
| Tropical Latin America | 2018 | 0.64018 | 1.153851 | 24.47436 | 0.428758 | 12.5779 | 0.888619 | 22.6798 |
| Tropical Latin America | 2019 | 0.644694 | 1.17357 | 24.99346 | 0.437713 | 12.76272 | 0.906608 | 23.26078 |
| Tropical Latin America | 2020 | 0.648603 | 1.187257 | 25.37795 | 0.446501 | 13.08537 | 0.918107 | 23.68644 |
| Tropical Latin America | 2021 | 0.652442 | 1.192551 | 25.61447 | 0.455649 | 13.51995 | 0.922892 | 24.10286 |
| Western Europe | 1990 | 0.7464 | 1.735983 | 38.30976 | 0.634472 | 17.17904 | 0.801839 | 19.98887 |
| Western Europe | 1991 | 0.75167 | 1.751909 | 38.65171 | 0.63841 | 17.25353 | 0.798381 | 19.89439 |
| Western Europe | 1992 | 0.757022 | 1.765761 | 38.98045 | 0.6442 | 17.34151 | 0.794932 | 19.80468 |
| Western Europe | 1993 | 0.762057 | 1.782434 | 39.46665 | 0.652465 | 17.54489 | 0.778215 | 19.39658 |
| Western Europe | 1994 | 0.766693 | 1.77621 | 39.48335 | 0.652898 | 17.54792 | 0.763984 | 19.07149 |
| Western Europe | 1995 | 0.770622 | 1.774531 | 39.67237 | 0.661153 | 17.80273 | 0.753653 | 18.83233 |
| Western Europe | 1996 | 0.77408 | 1.766512 | 39.60359 | 0.66581 | 17.89404 | 0.759413 | 19.05079 |
| Western Europe | 1997 | 0.777578 | 1.745521 | 39.23363 | 0.66736 | 17.86854 | 0.756542 | 19.00722 |
| Western Europe | 1998 | 0.780867 | 1.727977 | 38.78831 | 0.66488 | 17.74148 | 0.754605 | 18.92518 |
| Western Europe | 1999 | 0.784019 | 1.716316 | 38.54326 | 0.658423 | 17.49954 | 0.747979 | 18.75528 |
| Western Europe | 2000 | 0.7874 | 1.714096 | 38.62383 | 0.661242 | 17.56834 | 0.756424 | 18.9993 |
| Western Europe | 2001 | 0.790904 | 1.709042 | 38.6079 | 0.663515 | 17.60765 | 0.771462 | 19.45793 |
| Western Europe | 2002 | 0.794165 | 1.70399 | 38.4583 | 0.665917 | 17.64621 | 0.786058 | 19.80549 |
| Western Europe | 2003 | 0.796962 | 1.687716 | 38.16753 | 0.665711 | 17.58541 | 0.823714 | 20.87318 |
| Western Europe | 2004 | 0.799716 | 1.656923 | 37.59711 | 0.65795 | 17.32289 | 0.832968 | 21.18646 |
| Western Europe | 2005 | 0.802414 | 1.633283 | 37.19266 | 0.644509 | 16.96816 | 0.839379 | 21.48793 |
| Western Europe | 2006 | 0.805067 | 1.62004 | 36.98289 | 0.643469 | 16.89124 | 0.837587 | 21.53016 |
| Western Europe | 2007 | 0.807723 | 1.608429 | 36.73096 | 0.642627 | 16.82828 | 0.842035 | 21.66849 |
| Western Europe | 2008 | 0.810484 | 1.615008 | 36.85463 | 0.639952 | 16.66658 | 0.850861 | 21.83636 |
| Western Europe | 2009 | 0.81311 | 1.591505 | 36.30313 | 0.637411 | 16.57795 | 0.855493 | 21.96101 |
| Western Europe | 2010 | 0.816142 | 1.567229 | 35.61297 | 0.617674 | 16.06014 | 0.871092 | 22.38673 |
| Western Europe | 2011 | 0.819375 | 1.545059 | 35.01425 | 0.619757 | 16.07348 | 0.877836 | 22.50144 |
| Western Europe | 2012 | 0.822634 | 1.531786 | 34.54876 | 0.614157 | 15.88361 | 0.885111 | 22.64984 |
| Western Europe | 2013 | 0.825787 | 1.522172 | 34.16944 | 0.610076 | 15.69635 | 0.898888 | 22.97658 |
| Western Europe | 2014 | 0.82861 | 1.497557 | 33.44256 | 0.604379 | 15.51445 | 0.903047 | 23.05695 |
| Western Europe | 2015 | 0.831322 | 1.506747 | 33.48454 | 0.592029 | 15.12797 | 0.919954 | 23.43211 |
| Western Europe | 2016 | 0.834216 | 1.51005 | 33.45074 | 0.599023 | 15.31363 | 0.925753 | 23.56753 |
| Western Europe | 2017 | 0.837522 | 1.501197 | 33.19133 | 0.599873 | 15.28448 | 0.93946 | 23.86242 |
| Western Europe | 2018 | 0.840834 | 1.502842 | 33.14759 | 0.604362 | 15.41297 | 0.954755 | 24.31706 |
| Western Europe | 2019 | 0.844212 | 1.482944 | 32.77956 | 0.599168 | 15.27582 | 0.950106 | 24.23087 |
| Western Europe | 2020 | 0.84655 | 1.442126 | 31.66128 | 0.578865 | 14.5938 | 0.920086 | 23.18331 |
| Western Europe | 2021 | 0.848726 | 1.433656 | 31.7006 | 0.58027 | 14.70316 | 0.917399 | 23.28399 |
| Western Sub-Saharan Africa | 1990 | 0.2737 | 0.622187 | 13.10552 | 0.073763 | 2.183053 | 0.304287 | 8.003824 |
| Western Sub-Saharan Africa | 1991 | 0.277411 | 0.640094 | 13.48544 | 0.077393 | 2.291088 | 0.310296 | 8.16728 |
| Western Sub-Saharan Africa | 1992 | 0.281025 | 0.65202 | 13.73663 | 0.079953 | 2.363183 | 0.31625 | 8.310957 |
| Western Sub-Saharan Africa | 1993 | 0.284432 | 0.669736 | 14.10749 | 0.083263 | 2.458139 | 0.321732 | 8.440767 |
| Western Sub-Saharan Africa | 1994 | 0.2877 | 0.682332 | 14.39041 | 0.085734 | 2.528676 | 0.327472 | 8.580067 |
| Western Sub-Saharan Africa | 1995 | 0.290983 | 0.696057 | 14.66907 | 0.088226 | 2.599307 | 0.333258 | 8.718158 |
| Western Sub-Saharan Africa | 1996 | 0.294618 | 0.70944 | 14.93614 | 0.09134 | 2.69048 | 0.338928 | 8.856749 |
| Western Sub-Saharan Africa | 1997 | 0.298302 | 0.717306 | 15.08991 | 0.09383 | 2.762801 | 0.344454 | 8.989107 |
| Western Sub-Saharan Africa | 1998 | 0.302026 | 0.731943 | 15.41524 | 0.096712 | 2.848301 | 0.350546 | 9.135865 |
| Western Sub-Saharan Africa | 1999 | 0.30581 | 0.743754 | 15.6597 | 0.099649 | 2.936574 | 0.356843 | 9.287557 |
| Western Sub-Saharan Africa | 2000 | 0.309781 | 0.762303 | 16.02509 | 0.102665 | 3.018325 | 0.364851 | 9.453006 |
| Western Sub-Saharan Africa | 2001 | 0.314028 | 0.780558 | 16.45275 | 0.106918 | 3.151869 | 0.375514 | 9.742466 |
| Western Sub-Saharan Africa | 2002 | 0.318881 | 0.797385 | 16.79789 | 0.110089 | 3.233988 | 0.384295 | 9.916538 |
| Western Sub-Saharan Africa | 2003 | 0.324233 | 0.814888 | 17.22695 | 0.113483 | 3.334176 | 0.390947 | 10.07631 |
| Western Sub-Saharan Africa | 2004 | 0.330228 | 0.836644 | 17.73755 | 0.11735 | 3.443193 | 0.39903 | 10.25392 |
| Western Sub-Saharan Africa | 2005 | 0.33678 | 0.854587 | 18.13476 | 0.120382 | 3.527508 | 0.40636 | 10.4066 |
| Western Sub-Saharan Africa | 2006 | 0.343248 | 0.877167 | 18.60786 | 0.123882 | 3.613947 | 0.415252 | 10.57047 |
| Western Sub-Saharan Africa | 2007 | 0.35004 | 0.894129 | 19.04411 | 0.127422 | 3.722313 | 0.421524 | 10.73466 |
| Western Sub-Saharan Africa | 2008 | 0.356716 | 0.915821 | 19.49369 | 0.130738 | 3.801095 | 0.428504 | 10.85103 |
| Western Sub-Saharan Africa | 2009 | 0.363492 | 0.948417 | 20.25631 | 0.136206 | 3.96085 | 0.438618 | 11.09787 |
| Western Sub-Saharan Africa | 2010 | 0.370609 | 0.981979 | 21.0336 | 0.141342 | 4.109322 | 0.44757 | 11.29985 |
| Western Sub-Saharan Africa | 2011 | 0.377609 | 1.029445 | 22.13171 | 0.148557 | 4.316458 | 0.458788 | 11.55906 |
| Western Sub-Saharan Africa | 2012 | 0.384753 | 1.067398 | 23.02971 | 0.155124 | 4.505908 | 0.469932 | 11.82695 |
| Western Sub-Saharan Africa | 2013 | 0.39189 | 1.11404 | 24.11421 | 0.162924 | 4.731793 | 0.484052 | 12.169 |
| Western Sub-Saharan Africa | 2014 | 0.399162 | 1.149257 | 24.99913 | 0.169417 | 4.928996 | 0.494808 | 12.458 |
| Western Sub-Saharan Africa | 2015 | 0.406238 | 1.174221 | 25.54725 | 0.173328 | 5.029241 | 0.497272 | 12.47449 |
| Western Sub-Saharan Africa | 2016 | 0.412927 | 1.211409 | 26.4058 | 0.180377 | 5.230303 | 0.506526 | 12.70285 |
| Western Sub-Saharan Africa | 2017 | 0.419615 | 1.248924 | 27.27561 | 0.187123 | 5.417931 | 0.513511 | 12.8548 |
| Western Sub-Saharan Africa | 2018 | 0.426437 | 1.279994 | 28.01854 | 0.192281 | 5.553274 | 0.518348 | 12.94069 |
| Western Sub-Saharan Africa | 2019 | 0.433384 | 1.316603 | 28.84136 | 0.19858 | 5.724391 | 0.524008 | 13.05513 |
| Western Sub-Saharan Africa | 2020 | 0.439822 | 1.347302 | 29.52098 | 0.205385 | 5.913979 | 0.530668 | 13.2112 |
| Western Sub-Saharan Africa | 2021 | 0.446023 | 1.383595 | 30.29426 | 0.212866 | 6.118493 | 0.539623 | 13.41384 |
