# Supplementary material for: High BMI-attributable female-specific cancers: a comprehensive analysis of the global disease burden and trends from 1990 to 2021 and projections to 2040
Source: Front Oncol. 2025 Oct 29;15:1704299. doi: 10.3389/fonc.2025.1704299 (PMC12605095; doi:10.3389/fonc.2025.1704299)
Supplement: Supplementary file 8 [file Table7.docx]

| **Table S7**  High BMI-attributable breast, ovarian and uterine cancer ASDR and ASDALYR by age group across different SDI regions, 2021. | | | | | | | |
| --- | --- | --- | --- | --- | --- | --- | --- |
|  | | Breast cancer | | Ovarian cancer | | Uterine cancer | |
| Location | Age | Deaths Number  (Per 100,000) | DALYs Number  (Per 100,000) | Deaths Number  (Per 100,000) | DALYs Number  (Per 100,000) | Deaths Number  (Per 100,000) | DALYs Number  (Per 100,000) |
| Global | 20-24 | -0.01 (-0.03, 0) | -0.94 (-2.15, 0) | 0.01 (0, 0.02) | 0.74 (0.1, 1.43) | 0.01 (0, 0.01) | 0.49 (0.34, 0.67) |
| Global | 25-29 | -0.05 (-0.1, 0) | -3.06 (-6.73, 0) | 0.02 (0, 0.04) | 1.52 (0.28, 2.88) | 0.02 (0.01, 0.03) | 1.35 (0.92, 1.81) |
| Global | 30-34 | -0.14 (-0.3, 0) | -8.24 (-17.98, -0.01) | 0.05 (0.01, 0.08) | 2.79 (0.59, 5.07) | 0.05 (0.03, 0.06) | 2.95 (1.99, 3.96) |
| Global | 35-39 | -0.31 (-0.68, 0) | -17.16 (-37.6, -0.01) | 0.1 (0.02, 0.17) | 5.23 (1.18, 9.46) | 0.1 (0.07, 0.14) | 6.04 (4.14, 8.06) |
| Global | 40-44 | -0.57 (-1.28, 0) | -29.13 (-64.63, -0.02) | 0.21 (0.05, 0.38) | 10.58 (2.46, 18.86) | 0.23 (0.16, 0.31) | 12.03 (8.57, 16.18) |
| Global | 45-49 | -0.86 (-1.93, 0) | -39.58 (-87.97, -0.03) | 0.42 (0.1, 0.75) | 18.77 (4.42, 33.45) | 0.48 (0.33, 0.63) | 22.66 (15.93, 30) |
| Global | 50-54 | 2.87 (-0.09, 5.78) | 117.12 (-3.76, 236.41) | 0.73 (0.17, 1.28) | 28.58 (6.85, 50.51) | 0.89 (0.63, 1.19) | 38.74 (27.57, 51.42) |
| Global | 55-59 | 3.72 (-0.11, 7.53) | 133.63 (-4.09, 271.22) | 1.05 (0.27, 1.88) | 36.15 (9.21, 64.48) | 1.55 (1.1, 2.05) | 59.1 (41.87, 77.78) |
| Global | 60-64 | 4.28 (-0.13, 8.46) | 134.85 (-4.1, 266.67) | 1.47 (0.35, 2.57) | 43.71 (10.28, 76.28) | 2.87 (2.05, 3.72) | 92.65 (66.39, 120.19) |
| Global | 65-69 | 4.9 (-0.14, 9.78) | 131.55 (-3.89, 261.91) | 1.8 (0.44, 3.16) | 45.06 (11.1, 79.17) | 3.91 (2.81, 5.11) | 105.34 (76.07, 137.26) |
| Global | 70-74 | 5.84 (-0.18, 11.82) | 129.73 (-3.92, 262.08) | 2.16 (0.53, 3.94) | 44.37 (10.89, 81.15) | 4.83 (3.5, 6.33) | 106.56 (76.54, 139.97) |
| Global | 75-79 | 6.78 (-0.2, 13.84) | 120.96 (-3.52, 246.32) | 2.37 (0.55, 4.23) | 38.75 (9.04, 69.31) | 5.24 (3.78, 6.89) | 92.07 (66.58, 121.74) |
| Global | 80-84 | 8.12 (-0.22, 16.76) | 113.45 (-3.08, 233.03) | 2.35 (0.53, 4.34) | 30.12 (6.75, 55.54) | 5.92 (4.01, 7.95) | 79.59 (53.91, 106.52) |
| Global | 85-89 | 11.19 (-0.29, 23.02) | 124.7 (-3.23, 255.86) | 2.69 (0.59, 5.03) | 27.25 (5.92, 50.92) | 6.68 (4.3, 9.11) | 70.71 (45.48, 96.49) |
| Global | 90-94 | 17.23 (-0.46, 35.95) | 159.96 (-4.24, 332.6) | 3.27 (0.74, 6.12) | 28.83 (6.49, 53.92) | 9.03 (5.66, 12.35) | 80.68 (50.73, 110.08) |
| Global | 95+ | 23.99 (-0.61, 50.88) | 203.96 (-5.21, 430.72) | 3.37 (0.77, 6.36) | 27.64 (6.32, 52.11) | 10.05 (6.12, 13.91) | 83.41 (51.01, 115.74) |
| High SDI | 20-24 | -0.01 (-0.01, 0) | -0.43 (-0.94, 0) | 0.01 (0, 0.02) | 0.95 (0.19, 1.73) | 0 (0, 0.01) | 0.37 (0.26, 0.5) |
| High SDI | 25-29 | -0.04 (-0.08, 0) | -2.55 (-5.54, 0) | 0.03 (0.01, 0.05) | 1.95 (0.44, 3.53) | 0.02 (0.01, 0.02) | 1.19 (0.85, 1.57) |
| High SDI | 30-34 | -0.15 (-0.32, 0) | -9.36 (-20.55, -0.01) | 0.06 (0.01, 0.1) | 3.59 (0.88, 6.35) | 0.06 (0.04, 0.07) | 3.74 (2.7, 4.82) |
| High SDI | 35-39 | -0.32 (-0.71, 0) | -19.03 (-41.38, -0.01) | 0.11 (0.03, 0.2) | 6.37 (1.61, 11.18) | 0.13 (0.09, 0.16) | 7.83 (5.61, 10.22) |
| High SDI | 40-44 | -0.59 (-1.29, 0) | -31.55 (-68.67, -0.02) | 0.25 (0.06, 0.44) | 12.5 (2.99, 21.93) | 0.25 (0.18, 0.32) | 14.33 (10.31, 18.5) |
| High SDI | 45-49 | -0.93 (-2.08, 0) | -45.27 (-100.46, -0.03) | 0.5 (0.12, 0.88) | 22.38 (5.48, 39.44) | 0.55 (0.4, 0.7) | 27.87 (20.25, 35.76) |
| High SDI | 50-54 | 3.05 (-0.1, 6.02) | 130.82 (-4.13, 258.42) | 0.93 (0.23, 1.66) | 36.52 (8.87, 65.74) | 1.09 (0.8, 1.41) | 51.31 (37.52, 65.97) |
| High SDI | 55-59 | 4.2 (-0.12, 8.29) | 158.25 (-4.5, 313.67) | 1.45 (0.39, 2.52) | 49.96 (13.33, 86.78) | 2.01 (1.46, 2.56) | 82.28 (59.59, 105.35) |
| High SDI | 60-64 | 5.25 (-0.17, 10.3) | 173.75 (-5.65, 335.53) | 2.13 (0.55, 3.73) | 63.51 (16.46, 111.09) | 3.98 (2.9, 5.07) | 135.51 (99.43, 173.06) |
| High SDI | 65-69 | 6.54 (-0.19, 12.73) | 184.99 (-5.48, 360.68) | 2.86 (0.73, 5.14) | 71.72 (18.35, 129) | 5.83 (4.21, 7.51) | 163.8 (119.96, 210.68) |
| High SDI | 70-74 | 8.09 (-0.25, 16.14) | 188.02 (-5.86, 369.95) | 3.56 (0.9, 6.34) | 73.02 (18.68, 130.56) | 7.33 (5.26, 9.5) | 167.05 (120.36, 215.2) |
| High SDI | 75-79 | 10.35 (-0.3, 20.9) | 192.71 (-5.56, 389) | 4.31 (1.05, 7.8) | 70.51 (17.11, 127.57) | 8.6 (6.09, 11.43) | 155.47 (109.8, 203.99) |
| High SDI | 80-84 | 11.95 (-0.31, 24.56) | 173.35 (-4.5, 354.08) | 4.08 (0.91, 7.61) | 52.37 (11.64, 97.73) | 8.79 (5.77, 12.01) | 120.25 (78.56, 164.42) |
| High SDI | 85-89 | 16.12 (-0.4, 32.63) | 185.47 (-4.58, 378.94) | 4.44 (0.96, 8.35) | 44.93 (9.73, 84.67) | 9.91 (6.27, 13.51) | 106.07 (67.4, 145.09) |
| High SDI | 90-94 | 23.99 (-0.62, 49.47) | 225.78 (-5.8, 467.31) | 5 (1.12, 9.45) | 44.05 (9.91, 83.28) | 12.5 (7.7, 17.15) | 112.08 (69.14, 154.08) |
| High SDI | 95+ | 31.33 (-0.78, 66.23) | 267.65 (-6.62, 562.27) | 4.67 (1.06, 8.89) | 38.29 (8.73, 72.82) | 13.19 (8.09, 18.43) | 109.5 (67.18, 153.1) |
| High-middle SDI | 20-24 | 0 (-0.01, 0) | -0.33 (-0.72, 0) | 0.01 (0, 0.02) | 0.72 (0.1, 1.37) | 0.01 (0.01, 0.01) | 0.59 (0.39, 0.81) |
| High-middle SDI | 25-29 | -0.02 (-0.05, 0) | -1.45 (-3.21, 0) | 0.02 (0, 0.04) | 1.54 (0.29, 2.83) | 0.02 (0.02, 0.03) | 1.59 (1.04, 2.16) |
| High-middle SDI | 30-34 | -0.08 (-0.18, 0) | -5.16 (-11.23, 0) | 0.05 (0.01, 0.09) | 2.93 (0.6, 5.35) | 0.06 (0.04, 0.09) | 3.9 (2.65, 5.32) |
| High-middle SDI | 35-39 | -0.22 (-0.48, 0) | -12.41 (-27.55, -0.01) | 0.11 (0.02, 0.2) | 6.06 (1.37, 10.94) | 0.15 (0.1, 0.2) | 8.63 (5.91, 11.68) |
| High-middle SDI | 40-44 | -0.46 (-1.04, 0) | -24 (-54.27, -0.02) | 0.27 (0.06, 0.5) | 13.54 (3.14, 24.64) | 0.32 (0.22, 0.43) | 17.07 (11.93, 23.3) |
| High-middle SDI | 45-49 | -0.72 (-1.57, 0) | -33.7 (-73.11, -0.02) | 0.55 (0.13, 1.01) | 24.57 (5.71, 44.7) | 0.61 (0.43, 0.82) | 29.76 (21.01, 39.77) |
| High-middle SDI | 50-54 | 2.98 (-0.09, 5.8) | 123.18 (-3.77, 239.71) | 0.95 (0.23, 1.7) | 37.4 (8.92, 67.11) | 1.14 (0.78, 1.55) | 50.68 (35.1, 68.37) |
| High-middle SDI | 55-59 | 4.1 (-0.13, 8.19) | 148.23 (-4.55, 291.8) | 1.38 (0.35, 2.48) | 47.45 (11.89, 85.51) | 2.05 (1.43, 2.74) | 79.69 (56.36, 106.47) |
| High-middle SDI | 60-64 | 5.31 (-0.15, 10.43) | 166.89 (-4.86, 325.77) | 2.05 (0.49, 3.61) | 60.83 (14.69, 107.05) | 3.98 (2.79, 5.17) | 129.55 (91.66, 168.44) |
| High-middle SDI | 65-69 | 5.94 (-0.18, 11.73) | 158.57 (-4.74, 310.58) | 2.41 (0.63, 4.19) | 60.08 (15.73, 104.92) | 5.21 (3.69, 6.8) | 140.82 (100.26, 184.11) |
| High-middle SDI | 70-74 | 7.1 (-0.21, 14.1) | 156.12 (-4.66, 310.33) | 2.75 (0.7, 4.93) | 56.44 (14.43, 101.1) | 6.41 (4.52, 8.42) | 141.53 (99.71, 186.73) |
| High-middle SDI | 75-79 | 8.09 (-0.24, 16.49) | 141.98 (-4.2, 289.6) | 2.77 (0.65, 4.91) | 45.04 (10.48, 80) | 6.44 (4.58, 8.52) | 112.62 (80.47, 148.87) |
| High-middle SDI | 80-84 | 9.68 (-0.27, 19.97) | 132.57 (-3.68, 275.75) | 2.68 (0.6, 4.96) | 34.21 (7.7, 63.34) | 7.81 (5.36, 10.47) | 104.65 (72.58, 140.57) |
| High-middle SDI | 85-89 | 12.32 (-0.33, 26.02) | 134.14 (-3.63, 283.28) | 2.68 (0.59, 5.05) | 27.15 (5.97, 51.21) | 7.56 (4.93, 10.38) | 80.1 (52.38, 109.8) |
| High-middle SDI | 90-94 | 17.53 (-0.49, 37.19) | 160.4 (-4.46, 340.09) | 2.95 (0.67, 5.59) | 26.02 (5.94, 49.26) | 9.61 (6.15, 13.24) | 85.82 (54.98, 118.25) |
| High-middle SDI | 95+ | 24.3 (-0.66, 51.4) | 205.97 (-5.61, 436.19) | 3.11 (0.71, 5.9) | 25.63 (5.85, 48.69) | 10.17 (6.18, 14.24) | 84.87 (51.55, 119.08) |
| Low SDI | 20-24 | -0.02 (-0.04, 0) | -1.24 (-2.91, 0) | 0 (0, 0.01) | 0.33 (-0.02, 0.78) | 0 (0, 0.01) | 0.32 (0.2, 0.48) |
| Low SDI | 25-29 | -0.06 (-0.13, 0) | -3.87 (-8.69, 0) | 0.01 (0, 0.02) | 0.76 (0.06, 1.6) | 0.02 (0.01, 0.02) | 0.97 (0.62, 1.43) |
| Low SDI | 30-34 | -0.16 (-0.37, 0) | -9.43 (-21.76, -0.01) | 0.03 (0, 0.05) | 1.55 (0.21, 3.12) | 0.03 (0.02, 0.05) | 1.97 (1.21, 2.98) |
| Low SDI | 35-39 | -0.36 (-0.85, 0) | -19.75 (-46.33, -0.01) | 0.05 (0.01, 0.1) | 2.69 (0.44, 5.25) | 0.06 (0.04, 0.09) | 3.24 (2.04, 4.81) |
| Low SDI | 40-44 | -0.63 (-1.42, 0) | -31.17 (-70.24, -0.02) | 0.11 (0.02, 0.21) | 5.34 (0.8, 10.56) | 0.15 (0.09, 0.22) | 7.28 (4.46, 10.98) |
| Low SDI | 45-49 | -1.04 (-2.31, 0) | -45.92 (-102.24, -0.03) | 0.22 (0.04, 0.42) | 9.58 (1.58, 18.59) | 0.3 (0.19, 0.43) | 13.28 (8.49, 19.06) |
| Low SDI | 50-54 | 2.46 (-0.07, 5.13) | 96.57 (-2.9, 201.27) | 0.42 (0.07, 0.81) | 16.45 (2.86, 31.66) | 0.58 (0.37, 0.85) | 23.19 (14.9, 34.15) |
| Low SDI | 55-59 | 2.89 (-0.08, 6.05) | 99.6 (-2.82, 208.35) | 0.56 (0.1, 1.09) | 19.31 (3.39, 37.53) | 1.02 (0.65, 1.49) | 35.95 (22.78, 52.24) |
| Low SDI | 60-64 | 2.85 (-0.08, 5.85) | 84.87 (-2.5, 174.01) | 0.68 (0.1, 1.31) | 20.13 (3.08, 38.65) | 1.72 (1.11, 2.47) | 51.57 (33.33, 74.38) |
| Low SDI | 65-69 | 3.12 (-0.09, 6.44) | 78.62 (-2.15, 162.41) | 0.69 (0.11, 1.36) | 17.11 (2.63, 33.92) | 2.23 (1.41, 3.2) | 56.17 (35.64, 80.68) |
| Low SDI | 70-74 | 3.49 (-0.1, 7.09) | 72.38 (-2.14, 147.38) | 0.65 (0.07, 1.29) | 13.4 (1.45, 26.39) | 2.12 (1.38, 3.1) | 43.92 (28.69, 64.43) |
| Low SDI | 75-79 | 3.23 (-0.09, 6.69) | 53.66 (-1.54, 111.31) | 0.58 (0.05, 1.18) | 9.5 (0.87, 19.24) | 2.16 (1.38, 3.15) | 35.72 (22.78, 52.21) |
| Low SDI | 80-84 | 4.33 (-0.11, 8.91) | 56.24 (-1.5, 115.79) | 0.4 (-0.02, 0.92) | 5.15 (-0.31, 11.91) | 1.99 (1.24, 2.94) | 25.63 (16.02, 37.81) |
| Low SDI | 85-89 | 4.68 (-0.12, 9.56) | 48.28 (-1.23, 98.41) | 0.4 (-0.01, 0.88) | 4.03 (-0.06, 8.93) | 1.86 (1.18, 2.77) | 19.02 (12.04, 28.4) |
| Low SDI | 90-94 | 6.36 (-0.16, 13.09) | 56.37 (-1.46, 116.21) | 0.37 (0, 0.79) | 3.23 (0.01, 6.95) | 2.27 (1.38, 3.39) | 19.99 (12.11, 29.83) |
| Low SDI | 95+ | 11.7 (-0.29, 25.01) | 98.26 (-2.44, 209.95) | 0.25 (0.01, 0.53) | 2.09 (0.08, 4.47) | 1.57 (0.91, 2.55) | 13.19 (7.66, 21.55) |
| Low-middle SDI | 20-24 | -0.02 (-0.05, 0) | -1.6 (-3.6, 0) | 0.01 (0, 0.02) | 0.64 (0.05, 1.39) | 0.01 (0, 0.01) | 0.45 (0.29, 0.69) |
| Low-middle SDI | 25-29 | -0.07 (-0.15, 0) | -4.61 (-10.03, 0) | 0.02 (0, 0.04) | 1.35 (0.2, 2.58) | 0.02 (0.01, 0.03) | 1.28 (0.83, 1.87) |
| Low-middle SDI | 30-34 | -0.19 (-0.41, 0) | -11.25 (-24.55, -0.01) | 0.04 (0.01, 0.08) | 2.52 (0.47, 4.79) | 0.04 (0.03, 0.06) | 2.32 (1.53, 3.34) |
| Low-middle SDI | 35-39 | -0.4 (-0.89, 0) | -21.7 (-48.71, -0.02) | 0.08 (0.02, 0.15) | 4.5 (0.88, 8.32) | 0.08 (0.05, 0.11) | 4.36 (2.86, 6.11) |
| Low-middle SDI | 40-44 | -0.73 (-1.58, 0) | -36.18 (-78.5, -0.03) | 0.17 (0.04, 0.31) | 8.49 (1.84, 15.49) | 0.18 (0.12, 0.24) | 8.81 (5.88, 12.09) |
| Low-middle SDI | 45-49 | -1.11 (-2.48, 0) | -49.74 (-110.67, -0.03) | 0.33 (0.07, 0.6) | 14.76 (3.28, 26.68) | 0.37 (0.25, 0.49) | 16.71 (11.43, 22.34) |
| Low-middle SDI | 50-54 | 2.93 (-0.1, 6.01) | 116.1 (-3.95, 238.25) | 0.56 (0.13, 1.01) | 21.92 (4.96, 39.45) | 0.67 (0.45, 0.93) | 27.23 (18.19, 37.55) |
| Low-middle SDI | 55-59 | 3.57 (-0.12, 7.21) | 124.4 (-4.19, 249.94) | 0.79 (0.18, 1.46) | 26.99 (6.1, 50.33) | 1.14 (0.76, 1.57) | 40.79 (27.4, 55.89) |
| Low-middle SDI | 60-64 | 3.36 (-0.1, 6.75) | 101.43 (-2.9, 204.3) | 0.91 (0.18, 1.7) | 26.85 (5.45, 50.11) | 1.85 (1.25, 2.49) | 56.3 (37.78, 75.43) |
| Low-middle SDI | 65-69 | 3.84 (-0.12, 7.93) | 98.2 (-3.03, 202.94) | 1.07 (0.22, 1.98) | 26.63 (5.56, 49.41) | 2.49 (1.69, 3.45) | 63.42 (43.11, 87.48) |
| Low-middle SDI | 70-74 | 4.11 (-0.13, 8.34) | 86.57 (-2.68, 176.89) | 1.09 (0.22, 2.05) | 22.26 (4.44, 42.11) | 2.62 (1.78, 3.62) | 54.8 (37.18, 75.39) |
| Low-middle SDI | 75-79 | 3.99 (-0.11, 8.23) | 67.2 (-1.93, 138.42) | 0.99 (0.21, 1.84) | 16.23 (3.35, 30.15) | 2.6 (1.72, 3.67) | 43.28 (28.69, 61.1) |
| Low-middle SDI | 80-84 | 4.07 (-0.12, 8.48) | 53.67 (-1.62, 111.87) | 0.78 (0.12, 1.52) | 10.03 (1.49, 19.54) | 2.48 (1.65, 3.45) | 31.98 (21.32, 44.73) |
| Low-middle SDI | 85-89 | 4.9 (-0.13, 10.3) | 51.09 (-1.4, 107.24) | 0.8 (0.12, 1.57) | 8.13 (1.19, 16.04) | 2.55 (1.64, 3.58) | 26 (16.79, 36.66) |
| Low-middle SDI | 90-94 | 6.24 (-0.18, 13.25) | 55.55 (-1.59, 117.77) | 0.81 (0.12, 1.61) | 7.18 (1.05, 14.21) | 3.15 (2, 4.41) | 27.74 (17.65, 38.92) |
| Low-middle SDI | 95+ | 7.89 (-0.2, 16.73) | 64.86 (-1.69, 137.5) | 0.77 (0.14, 1.47) | 6.24 (1.1, 11.9) | 3.03 (1.77, 4.45) | 24.67 (14.48, 36.06) |
| Middle SDI | 20-24 | -0.01 (-0.02, 0) | -0.55 (-1.23, 0) | 0.01 (0, 0.03) | 1.03 (0.18, 2) | 0.01 (0.01, 0.01) | 0.62 (0.4, 0.85) |
| Middle SDI | 25-29 | -0.03 (-0.07, 0) | -2.18 (-4.84, 0) | 0.03 (0.01, 0.05) | 1.85 (0.39, 3.51) | 0.02 (0.02, 0.03) | 1.55 (1.01, 2.09) |
| Middle SDI | 30-34 | -0.11 (-0.24, 0) | -6.7 (-14.95, -0.01) | 0.05 (0.01, 0.09) | 3.06 (0.66, 5.63) | 0.05 (0.03, 0.07) | 2.98 (1.92, 4.11) |
| Middle SDI | 35-39 | -0.26 (-0.58, 0) | -14.75 (-32.44, -0.01) | 0.1 (0.02, 0.19) | 5.72 (1.28, 10.55) | 0.11 (0.07, 0.15) | 6.07 (3.99, 8.38) |
| Middle SDI | 40-44 | -0.5 (-1.14, 0) | -25.26 (-57.52, -0.01) | 0.23 (0.05, 0.42) | 11.2 (2.58, 20.92) | 0.23 (0.15, 0.32) | 11.94 (7.9, 16.39) |
| Middle SDI | 45-49 | -0.72 (-1.59, 0) | -32.71 (-72.37, -0.03) | 0.42 (0.1, 0.76) | 18.49 (4.39, 33.54) | 0.48 (0.33, 0.65) | 22.09 (15.07, 30.05) |
| Middle SDI | 50-54 | 2.76 (-0.09, 5.66) | 111.7 (-3.64, 229.5) | 0.65 (0.16, 1.17) | 25.5 (6.26, 45.78) | 0.83 (0.56, 1.15) | 34.84 (23.55, 48.1) |
| Middle SDI | 55-59 | 3.44 (-0.11, 7.2) | 122 (-3.77, 255.62) | 0.85 (0.21, 1.58) | 29.21 (7.36, 54.3) | 1.28 (0.88, 1.79) | 46.92 (32.66, 64.79) |
| Middle SDI | 60-64 | 3.69 (-0.11, 7.48) | 113.74 (-3.5, 230.7) | 1.08 (0.26, 1.93) | 31.94 (7.6, 56.99) | 2.11 (1.46, 2.88) | 65.27 (45.54, 89.08) |
| Middle SDI | 65-69 | 3.8 (-0.11, 7.86) | 99.41 (-2.87, 205.5) | 1.16 (0.28, 2.11) | 29.02 (6.95, 52.52) | 2.59 (1.78, 3.58) | 66.87 (45.85, 92.26) |
| Middle SDI | 70-74 | 4.08 (-0.12, 8.39) | 87.84 (-2.53, 180.04) | 1.24 (0.29, 2.29) | 25.46 (5.91, 46.94) | 2.87 (1.97, 3.98) | 60.83 (42.16, 84.28) |
| Middle SDI | 75-79 | 4.33 (-0.13, 9) | 74.38 (-2.17, 153.8) | 1.21 (0.28, 2.21) | 19.81 (4.57, 36.15) | 2.94 (2.02, 4.04) | 49.4 (33.9, 67.58) |
| Middle SDI | 80-84 | 4.57 (-0.13, 9.63) | 61.24 (-1.75, 128.55) | 1.03 (0.19, 1.96) | 13.21 (2.39, 25.23) | 2.88 (1.89, 3.93) | 37.42 (24.54, 50.99) |
| Middle SDI | 85-89 | 5.98 (-0.17, 12.82) | 63.42 (-1.78, 135.07) | 1.15 (0.22, 2.16) | 11.61 (2.21, 21.93) | 3.16 (1.98, 4.38) | 32.35 (20.21, 44.71) |
| Middle SDI | 90-94 | 7.84 (-0.22, 17.01) | 70.39 (-1.95, 152.06) | 1.24 (0.24, 2.27) | 10.89 (2.08, 20) | 3.84 (2.47, 5.31) | 33.84 (21.77, 46.86) |
| Middle SDI | 95+ | 9.61 (-0.27, 20.45) | 79.91 (-2.22, 170.14) | 1.15 (0.23, 2.15) | 9.44 (1.88, 17.68) | 4.14 (2.57, 5.86) | 34.24 (21.24, 48.56) |
