# Supplementary material for: High BMI-attributable female-specific cancers: a comprehensive analysis of the global disease burden and trends from 1990 to 2021 and projections to 2040
Source: Front Oncol. 2025 Oct 29;15:1704299. doi: 10.3389/fonc.2025.1704299 (PMC12605095; doi:10.3389/fonc.2025.1704299)
Supplement: Supplementary file 9 [file Table8.docx]

| **Table S8**  Decomposition analysis of the global high BMI-attributable breast, ovarian and uterine cancer burden from 1990 to 2021. | | | | | | | | | | | | | | |
| --- | --- | --- | --- | --- | --- | --- | --- | --- | --- | --- | --- | --- | --- | --- |
| Breast cancer | | | | | | | | | | | | | | |
|  | Deaths | | | | | | | DALYs | | | | | | |
| location name | Overall difference | Aging | Population | Epidemiological change | Aging percent | Population percent | r_percent | Overall difference | Aging | Population | Epidemiological change | Aging percent | Population percent | r_percent |
| Global | 105367.99 | -1127.305 | 94605.709 | 11889.583 | -1.07 | 89.79 | 11.28 | 1935107.69 | 32291.545 | 1594750.47 | 308065.671 | 1.67 | 82.41 | 15.92 |
| High SDI | 2769.29 | -533.701 | 5252.65 | -1949.662 | -19.27 | 189.67 | -70.4 | 2258.47 | -39987.88 | 100642.805 | -58396.452 | -1770.57 | 4456.24 | -2585.66 |
| High-middle SDI | 24950.27 | 6293.843 | 9229.852 | 9426.577 | 25.23 | 36.99 | 37.78 | 164785.38 | -133355.673 | 176044.895 | 122096.162 | -80.93 | 106.83 | 74.09 |
| Middle SDI | 17832.41 | -2153.046 | 7415.581 | 12569.874 | -12.07 | 41.58 | 70.49 | 375526.87 | 95877.365 | 104778.088 | 174871.414 | 25.53 | 27.9 | 46.57 |
| Low SDI | 17580.39 | 309.642 | 9338.799 | 7931.948 | 1.76 | 53.12 | 45.12 | 301174.14 | -5114.631 | 169382.339 | 136906.43 | -1.7 | 56.24 | 45.46 |
| Low-middle SDI | 29637.63 | 523.817 | 11132.093 | 17981.722 | 1.77 | 37.56 | 60.67 | 622162.7 | -7982.989 | 233436.478 | 396709.208 | -1.28 | 37.52 | 63.76 |
| Ovarian cancer | | | | | | | | | | | | | | |
|  | Deaths | | | | | | | DALYs | | | | | | |
| location name | Overall difference | Aging | Population | Epidemiological change | Aging percent | Population percent | r_percent | Overall difference | Aging | Population | Epidemiological change | Aging percent | Population percent | r_percent |
| Global | 35021.91 | -1052.472 | 25757.418 | 10316.961 | -3.01 | 73.55 | 29.46 | 779357.45 | -495.721 | 549658.46 | 230194.707 | -0.06 | 70.53 | 29.54 |
| High SDI | 890.66 | -468.548 | 1726.069 | -366.863 | -52.61 | 193.8 | -41.19 | 8062.57 | -16026.432 | 40302.664 | -16213.66 | -198.78 | 499.87 | -201.1 |
| High-middle SDI | 4353.69 | -1433.519 | 2699.14 | 3088.065 | -32.93 | 62 | 70.93 | 84164.17 | -38694.536 | 65746.488 | 57112.222 | -45.98 | 78.12 | 67.86 |
| Middle SDI | 5586.89 | 776.178 | 1331.513 | 3479.203 | 13.89 | 23.83 | 62.27 | 146620.37 | 8800.232 | 38195.251 | 99624.883 | 6 | 26.05 | 67.95 |
| Low SDI | 3043.23 | 2.392 | 1320.63 | 1720.212 | 0.08 | 43.4 | 56.53 | 85423.8 | -420.021 | 37596.161 | 48247.657 | -0.49 | 44.01 | 56.48 |
| Low-middle SDI | 8636.22 | 45.786 | 2565.742 | 6024.69 | 0.53 | 29.71 | 69.76 | 231012.55 | -1274.479 | 69085.005 | 163202.022 | -0.55 | 29.91 | 70.65 |
| Uterine cancer | | | | | | | | | | | | | | |
|  | Deaths | | | | | | | DALYs | | | | | | |
| location name | Overall difference | Aging | Population | Epidemiological change | Aging percent | Population percent | r_percent | Overall difference | Aging | Population | Epidemiological change | Aging percent | Population percent | r_percent |
| Global | 67088.66 | 2135.959 | 51516.162 | 13436.544 | 3.18 | 76.79 | 20.03 | 1480043.11 | 31926.679 | 1123830.58 | 324285.85 | 2.16 | 75.93 | 21.91 |
| High SDI | 5313.44 | -507.306 | 3080.857 | 2739.886 | -9.55 | 57.98 | 51.57 | 120456.25 | -24433.531 | 68349.613 | 76540.17 | -20.28 | 56.74 | 63.54 |
| High-middle SDI | 3792.28 | -3030.715 | 6589.857 | 233.136 | -79.92 | 173.77 | 6.15 | 52543.96 | -88009.88 | 150950.317 | -10396.478 | -167.5 | 287.28 | -19.79 |
| Middle SDI | 9236.98 | 2757.549 | 3313.029 | 3166.399 | 29.85 | 35.87 | 34.28 | 201644.48 | 39762.51 | 85371.774 | 76510.193 | 19.72 | 42.34 | 37.94 |
| Low SDI | 7014.94 | 89.121 | 4415.08 | 2510.736 | 1.27 | 62.94 | 35.79 | 175148.7 | 258.48 | 112611.907 | 62278.312 | 0.15 | 64.3 | 35.56 |
| Low-middle SDI | 14546.92 | 356.171 | 6944.443 | 7246.31 | 2.45 | 47.74 | 49.81 | 344580.22 | -657.925 | 166052.752 | 179185.392 | -0.19 | 48.19 | 52 |
