# Supplementary material for: High BMI-attributable female-specific cancers: a comprehensive analysis of the global disease burden and trends from 1990 to 2021 and projections to 2040
Source: Front Oncol. 2025 Oct 29;15:1704299. doi: 10.3389/fonc.2025.1704299 (PMC12605095; doi:10.3389/fonc.2025.1704299)
Supplement: Supplementary file 10 [file Table9.docx]

| **Table S9**  Slope index of inequality and 95% confidence interval for health inequality in breast, ovarian and uterine cancer, 1990-2021. | | | |
| --- | --- | --- | --- |
|  | Breast cancer | Ovarian cancer | Uterine cancer |
| year | SII 95%CI | SII 95%CI | SII 95%CI |
| 1990 | 42.71 (33.93, 51.48) | 14.87 (11.67, 18.06) | 28.26 (22.19, 34.34) |
| 1991 | 42.93 (33.91, 51.95) | 15.29 (12.03, 18.56) | 28.52 (22.36, 34.67) |
| 1992 | 43.73 (34.86, 52.6) | 15.72 (12.44, 19.01) | 28.79 (22.39, 35.19) |
| 1993 | 44.69 (35.7, 53.68) | 16.24 (12.87, 19.61) | 29.24 (22.94, 35.54) |
| 1994 | 44.97 (36.09, 53.84) | 16.66 (13.26, 20.06) | 29.33 (22.85, 35.81) |
| 1995 | 45.46 (36.55, 54.38) | 17.63 (14.13, 21.14) | 29.56 (23.15, 35.98) |
| 1996 | 46.08 (37.03, 55.13) | 18.33 (14.66, 22) | 29.97 (23.44, 36.5) |
| 1997 | 46.64 (37.34, 55.94) | 18.84 (15.15, 22.54) | 30.52 (23.81, 37.23) |
| 1998 | 47.14 (37.71, 56.57) | 19.3 (15.56, 23.05) | 30.74 (24.1, 37.38) |
| 1999 | 49.29 (39.79, 58.79) | 20.07 (16.25, 23.89) | 31.08 (24.4, 37.75) |
| 2000 | 51.25 (41.48, 61.02) | 21.14 (17.27, 25.01) | 31.79 (24.88, 38.69) |
| 2001 | 52.53 (42.3, 62.77) | 21.59 (17.83, 25.35) | 32.44 (25.35, 39.54) |
| 2002 | 54.2 (43.96, 64.44) | 21.89 (18.06, 25.71) | 33.07 (25.96, 40.19) |
| 2003 | 55.43 (45.12, 65.74) | 22.7 (18.91, 26.5) | 33.75 (26.48, 41.01) |
| 2004 | 55.27 (45.09, 65.44) | 23.14 (19.26, 27.02) | 33.95 (26.48, 41.41) |
| 2005 | 55.84 (45.98, 65.71) | 23.53 (19.66, 27.39) | 34.59 (26.89, 42.28) |
| 2006 | 56.92 (47.06, 66.78) | 24.15 (20.31, 28) | 35.1 (27.25, 42.96) |
| 2007 | 58.74 (48.55, 68.93) | 24.91 (21.1, 28.73) | 35.83 (27.85, 43.8) |
| 2008 | 60.78 (50.45, 71.11) | 26.24 (22.42, 30.06) | 36.05 (27.96, 44.14) |
| 2009 | 63.42 (53.09, 73.75) | 27.19 (23.27, 31.11) | 37.29 (29.04, 45.54) |
| 2010 | 66.06 (55.88, 76.24) | 27.51 (23.77, 31.26) | 38.79 (30.38, 47.21) |
| 2011 | 68.14 (57.75, 78.53) | 28.27 (24.34, 32.21) | 39.06 (30.53, 47.6) |
| 2012 | 69.38 (58.93, 79.83) | 28.44 (24.65, 32.24) | 39.77 (31.11, 48.43) |
| 2013 | 70.54 (60.11, 80.97) | 28.65 (24.98, 32.32) | 41.01 (31.8, 50.22) |
| 2014 | 71.18 (60.99, 81.37) | 28.85 (25.15, 32.55) | 42.39 (32.92, 51.86) |
| 2015 | 72.61 (62.23, 82.99) | 28.68 (24.94, 32.42) | 43.76 (34.04, 53.49) |
| 2016 | 73.41 (62.94, 83.89) | 28.93 (25.3, 32.57) | 45.13 (34.9, 55.36) |
| 2017 | 74.13 (63.68, 84.58) | 29.16 (25.44, 32.88) | 46.27 (35.94, 56.61) |
| 2018 | 75.17 (64.84, 85.49) | 29.33 (25.4, 33.25) | 47.03 (36.39, 57.67) |
| 2019 | 75.95 (65.12, 86.77) | 29.56 (25.64, 33.47) | 48 (36.88, 59.12) |
| 2020 | 72.91 (62.32, 83.5) | 28.67 (24.71, 32.64) | 46.37 (35.41, 57.33) |
| 2021 | 74.12 (62.55, 85.7) | 29.04 (24.8, 33.29) | 47.06 (35.89, 58.23) |
