# Supplementary material for: High BMI-attributable female-specific cancers: a comprehensive analysis of the global disease burden and trends from 1990 to 2021 and projections to 2040
Source: Front Oncol. 2025 Oct 29;15:1704299. doi: 10.3389/fonc.2025.1704299 (PMC12605095; doi:10.3389/fonc.2025.1704299)
Supplement: Supplementary file 11 [file Table10.docx]

| **Table S10**  Global projections of ASDR and ASDALYR for high BMI-attributable breast, ovarian and uterine cancer from 2022 to 2040. | | | | | | |
| --- | --- | --- | --- | --- | --- | --- |
|  | **Breast cancer** | | **Ovarian cancer** | | **Uterine cancer** | |
| Year | ASDR 95%CI  (Per 100,000) | ASDALYR 95%CI  (Per 100,000) | ASDR 95%CI  (Per 100,000) | ASDALYR 95%CI  (Per 100,000) | ASDR 95%CI  (Per 100,000) | ASDALYR 95%CI  (Per 100,000) |
| 2022 | 0.9492 (0.9401, 0.9582) | 21.9795 (21.7562, 22.2028) | 0.3797 (0.3748, 0.3846) | 10.6197 (10.49, 10.7495) | 0.7187 (0.708, 0.7295) | 19.3441 (19.0413, 19.6469) |
| 2023 | 0.9488 (0.9335, 0.9642) | 22.026 (21.6295, 22.4225) | 0.3815 (0.3746, 0.3883) | 10.679 (10.4955, 10.8626) | 0.7204 (0.7011, 0.7396) | 19.3948 (18.8583, 19.9313) |
| 2024 | 0.9471 (0.9278, 0.9664) | 22.0724 (21.5581, 22.5868) | 0.3832 (0.3748, 0.3917) | 10.7383 (10.5135, 10.9631) | 0.7211 (0.6944, 0.7479) | 19.4183 (18.6796, 20.157) |
| 2025 | 0.9449 (0.9236, 0.9662) | 22.1189 (21.5091, 22.7287) | 0.385 (0.3753, 0.3947) | 10.7976 (10.538, 11.0572) | 0.7215 (0.6882, 0.7548) | 19.4291 (18.5154, 20.3428) |
| 2026 | 0.9429 (0.9209, 0.9649) | 22.1654 (21.4731, 22.8576) | 0.3868 (0.3759, 0.3977) | 10.8569 (10.5666, 11.1471) | 0.7217 (0.6826, 0.7608) | 19.4341 (18.3668, 20.5015) |
| 2027 | 0.9414 (0.9193, 0.9635) | 22.2118 (21.4459, 22.9777) | 0.3886 (0.3767, 0.4005) | 10.9162 (10.5982, 11.2341) | 0.7218 (0.6776, 0.766) | 19.4365 (18.2321, 20.6409) |
| 2028 | 0.9405 (0.9184, 0.9627) | 22.2583 (21.4253, 23.0913) | 0.3903 (0.3775, 0.4032) | 10.9754 (10.632, 11.3188) | 0.7218 (0.6729, 0.7708) | 19.4375 (18.1089, 20.7662) |
| 2029 | 0.9402 (0.9181, 0.9624) | 22.3048 (21.4096, 23.1999) | 0.3921 (0.3784, 0.4059) | 11.0347 (10.6676, 11.4018) | 0.7219 (0.6686, 0.7751) | 19.438 (17.9952, 20.8808) |
| 2030 | 0.9403 (0.918, 0.9625) | 22.3512 (21.398, 23.3044) | 0.3939 (0.3793, 0.4085) | 11.094 (10.7046, 11.4834) | 0.7219 (0.6647, 0.7791) | 19.4383 (17.8895, 20.987) |
| 2031 | 0.9405 (0.9182, 0.9628) | 22.3977 (21.3897, 23.4056) | 0.3957 (0.3803, 0.411) | 11.1533 (10.7428, 11.5637) | 0.7219 (0.6609, 0.7828) | 19.4384 (17.7903, 21.0864) |
| 2032 | 0.9408 (0.9185, 0.9631) | 22.4441 (21.3843, 23.504) | 0.3974 (0.3813, 0.4136) | 11.2126 (10.7821, 11.643) | 0.7219 (0.6574, 0.7863) | 19.4384 (17.6967, 21.1801) |
| 2033 | 0.9411 (0.9188, 0.9634) | 22.4906 (21.3812, 23.6) | 0.3992 (0.3824, 0.4161) | 11.2718 (10.8222, 11.7215) | 0.7219 (0.6541, 0.7897) | 19.4384 (17.6078, 21.269) |
| 2034 | 0.9413 (0.919, 0.9637) | 22.5371 (21.3803, 23.6938) | 0.401 (0.3835, 0.4185) | 11.3311 (10.8632, 11.7991) | 0.7219 (0.6509, 0.7929) | 19.4384 (17.5231, 21.3538) |
| 2035 | 0.9415 (0.9191, 0.9638) | 22.5835 (21.3813, 23.7858) | 0.4028 (0.3846, 0.421) | 11.3904 (10.9048, 11.876) | 0.7219 (0.6479, 0.7959) | 19.4385 (17.4419, 21.435) |
| 2036 | 0.9415 (0.9192, 0.9638) | 22.63 (21.3839, 23.8761) | 0.4045 (0.3857, 0.4234) | 11.4497 (10.947, 11.9524) | 0.7219 (0.6449, 0.7988) | 19.4385 (17.3639, 21.5131) |
| 2037 | 0.9415 (0.9192, 0.9638) | 22.6764 (21.388, 23.9649) | 0.4063 (0.3869, 0.4258) | 11.509 (10.9898, 12.0281) | 0.7219 (0.6421, 0.8017) | 19.4385 (17.2887, 21.5882) |
| 2038 | 0.9415 (0.9191, 0.9638) | 22.7229 (21.3935, 24.0524) | 0.4081 (0.3881, 0.4281) | 11.5682 (11.0331, 12.1034) | 0.7219 (0.6394, 0.8044) | 19.4385 (17.216, 21.6609) |
| 2039 | 0.9414 (0.9191, 0.9638) | 22.7694 (21.4001, 24.1386) | 0.4099 (0.3893, 0.4305) | 11.6275 (11.0769, 12.1782) | 0.7219 (0.6368, 0.807) | 19.4385 (17.1457, 21.7312) |
| 2040 | 0.9414 (0.919, 0.9637) | 22.8158 (21.4079, 24.2237) | 0.4116 (0.3905, 0.4328) | 11.6868 (11.1211, 12.2526) | 0.7219 (0.6342, 0.8096) | 19.4385 (17.0774, 21.7995) |
